# Distribution and shared evolutionary history of the Fumonisin and AAL toxin biosynthetic gene clusters

**DOI:** 10.1186/s12864-025-12037-3

**Published:** 2026-01-21

**Authors:** Robert H. Proctor, Mark Busman, Hye-Seon Kim, Jon Menke, Alessandra Villani, Jessica M. Lohmar, Daren W. Brown, B. Gillian Turgeon, Antonia Susca, Antonio Moretti, Kathryn E. Bushley

**Affiliations:** 1 Agricultural Research Service, Mycotoxin Prevention and Applied Mycology Research Unit, USDA, National Center for Agricultural Utilization Research, 1815 N University St, Peoria, IL 61604 USA; 2 LabCorp Oncology, Minneapolis, MN USA; 3 Institute of Sciences of Food Production, National Council of Research (ISPA-CNR), Bari, 70126 Italy; 4 Agriculture Research Service, Southern Regional Research Center, Food and Feed Safety Research, USDA, 1100 Robert E. Lee Blvd, New Orleans, LA 70124 USA; 5 Section of Plant Pathology & Plant-Microbe Biology, School of Integrative Plant Science, Cornell University, Ithaca, NY USA; 6 Agricultural Research Service, Emerging Pests and Pathogens Unit, USDA, Robert Holley Center, Cornell University, Ithaca, NY 14853 USA

**Keywords:** Fumonisin, AAL toxin, Secondary metabolite biosynthetic gene cluster, Fungi, Mycotoxins, Horizontal gene transfer

## Abstract

**Background:**

Fumonisins are among the mycotoxins of most concern to food safety and are structurally similar to AAL toxins, a family of host selective toxins. Together, these two toxin families are produced by ecologically diverse species in three fungal classes: AAL toxins by *Alternaria arborescens* in class Dothideomycetes and fumonisins by *Aspergillus* species in class Eurotiomycetes and by *Fusarium* and *Tolypocladium* species in class Sordariomycetes. Although structural similarities suggest that AAL toxins and fumonisins have a common biogenic origin, the evolutionary origins and relationships of their biosynthetic genes are not clear.

**Results:**

Here, we used BLAST, comparative genomic, phylogenetic, and functional analyses to identify and characterize homologs of the fumonisin biosynthetic gene (*FUM*) cluster in fungi. Our analyses identified *FUM* cluster homologs in *A. arborescens* and in species of *Aspergillus*, *Bipolaris*, *Fusarium*, and *Tolypocladium*. The results also suggest that the *FUM* cluster likely evolved from an ancestral cluster with 11 *FUM* genes through multiple mechanisms, including (1) vertical transmission, (2) acquisition of additional genes by some cluster lineages, (3) duplication of individual *FUM* genes, and (4) either horizontal transfer of the cluster from the Sordariomycetes to the Dothideomycetes or duplication and differential loss. Overall, our results suggest that the AAL toxin and *FUM* clusters share a common evolutionary origin and indicate that structural variation of the chemical products of AAL toxins and fumonisins has resulted from variation in *FUM* gene content and function.

**Conclusions:**

The presence of *FUM* clusters in relatively few classes of fungi with distinct lifestyles (plant versus insect/animal pathogens) suggests an important role of *FUM* metabolites in diverse fungal-host interactions. This study advances our understanding of the role of specific *FUM* genes in toxin biosynthesis and will improve our ability to detect and predict the ability of fungi found in food and animal feed to synthesize these mycotoxins.

**Supplementary Information:**

The online version contains supplementary material available at 10.1186/s12864-025-12037-3.

## Background

**Fig. 1:**
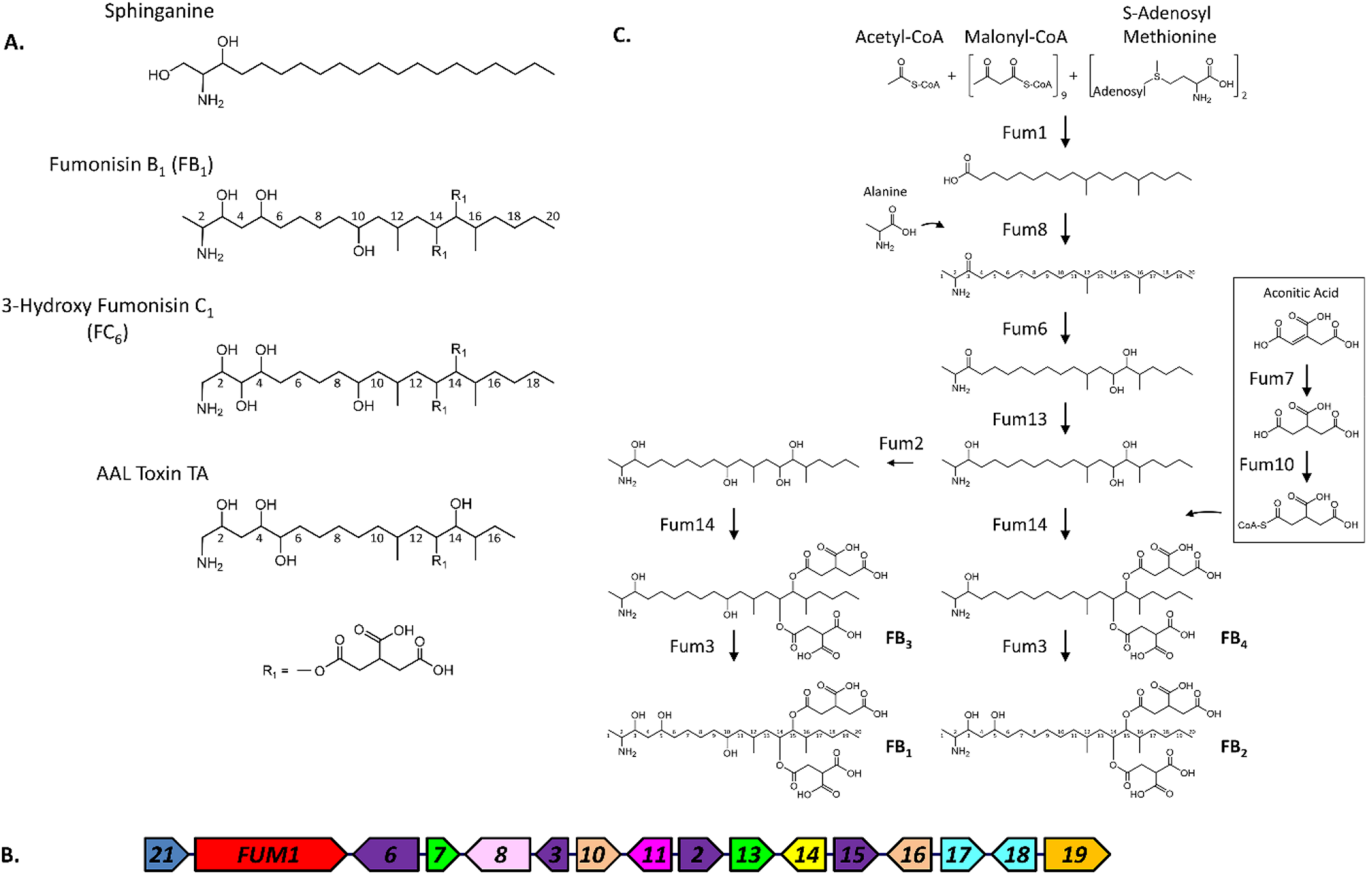
Sphinganine, sphinganine-analog metabolites (SAMs), and fumonisin biosynthesis. **A** Chemical structures of sphinganine and representative analogs of B fumonisins (FBs), C fumonisins (FCs), and AAL toxins. The numbers above or below the backbone structure indicate carbon atom number. R_1_ indicates tricarboxylate substituent esterified to the fumonisin and AAL toxin backbones. **B** The 16-gene fumonisin biosynthetic gene cluster (*FUM* cluster) in *Fusarium verticillioides*. **C** FB biosynthetic pathway. Fumonisin biosynthetic enzymes are indicated using the Fum designation followed by a number (e.g., Fum8 is the enzyme encoded by the *FUM8* gene)

The fungal secondary metabolites fumonisins and AAL toxins are considered sphinganine-analog metabolites (SAMs) because of their structural similarities to the sphingolipid precursor sphinganine (Fig. 1) and their ability to inhibit the enzyme ceramide synthase, thus disrupting sphingolipid metabolism [1–3]. Due to this, fumonisins are mycotoxins that pose health risks to humans and livestock because of their occurrence in food and feed crops, particularly maize (*Zea mays*) [4–7]. Production of fumonisins has been reported in several species of the fungus *Fusarium* (Class Sordariomycetes) [8–11], including the maize pathogens *F. proliferatum* and *F. verticillioides* and the rice pathogen *F. fujikuroi* [12, 13]. Although isolates of *Fusarium oxysporum* do not typically produce fumonisins, at least two producing isolates have been reported [10, 11, 14]. Fumonisin production has also been reported in multiple isolates of the black *Aspergillus* species *A. niger* and *A. welwitschiae* (Class Eurotiomycetes) [15–17], and three species of the entomopathogenic fungus *Tolypocladium* (Class Sordariomycetes) [18]. To our knowledge, production of AAL toxins has been reported only in *Alternaria arborescens* (Class Dothideomycetes), where they act as host-specific toxins against tomato (*Solanum lycopersicum*) [19].

Fumonisins consist of a linear polyketide backbone with one amine, two methyl, two tricarboxylate esters, and 1–4 hydroxyl groups along the backbone (Fig. 1A). The major analog groups, B- and C-series fumonisins (FBs and FCs) differ from one another in length of the backbone (20 carbon atoms in FBs and 19 in FCs) due to presence of a terminal methyl group next to the amine in FBs but not FCs [11]. Within the FB and FC series, analogs differ in the presence and absence of hydroxyl groups at carbon atoms along the backbone (Fig. 1A). Most fumonisin-producing *Fusarium* species produce predominantly FBs, typically the analogs FB_1_, FB_2_, FB_3_ and FB_4_, while fewer species produce predominantly FCs (Fig. 1A) [20, 21]. Fumonisin-producing *Aspergillus* and *Tolypocladium* species synthesize predominantly FB_2_ and FB_4_ but not FB_1_, FB_3_ or FCs [15–18]. AAL toxins differ in structure from fumonisins in that they have a 17-carbon-long backbone and only one tricarboxylate ester (Fig. 1A) [19]. Like FCs, AAL toxins lack a terminal methyl group next to the amine.

In fungi, secondary metabolite biosynthetic genes are typically located adjacent to one another in secondary metabolite biosynthetic gene clusters (SMBCs) [22]. These clusters generally contain a core gene encoding a synthase enzyme that catalyzes formation of a parent compound (e.g., a polyketide) and genes encoding modifying enzymes (cytochrome P450 monooxygenases, dioxygenases, short-chain dehydrogenases, esterases, methylases, among others) that catalyze modification of the parent compound and/or biosynthetic intermediates to form the final secondary metabolite product(s) [22, 23]. SMBCs can also include genes encoding transcription factors, proteins that provide protection from toxicity of pathway products, and proteins that transport the metabolites and/or their precursors within or out of cells in which they are produced.

In *Fusarium* species, the fumonisin biosynthetic gene cluster (*FUM* cluster) typically consists of 16 genes: *FUM1* – *FUM3*, *FUM6* – *FUM8*, *FUM10*, *FUM11*, *FUM13* – *FUM19* and *FUM21* [3, 24, 25]. *FUM1* is a core synthase gene that encodes a polyketide synthase (PKS), and *FUM2*, *FUM3*, *FUM6* – *FUM8*, *FUM10*, *FUM13* – *FUM16* encode modifying enzymes (Fig. 1B) [3, 24, 25]. Functional analyses of these genes in *F. verticillioides* have led to a proposed FB biosynthetic pathway (Fig. 1B, C, STable 3). In the pathway, the *FUM1*-encoded PKS (Fum1) catalyzes synthesis of an 18-carbon-long polyketide, and then the *FUM8*-encoded aminotransferase (Fum8) catalyzes condensation of the polyketide with alanine to form the fumonisin B backbone with an amine at C2 [11]. Subsequently, enzymes encoded by *FUM2*, *FUM3*, *FUM6*, *FUM7*, *FUM10*, *FUM13*, and *FUM14* catalyze modification of the backbone to yield FB_1_ – FB_4_ (Fig. 1C). The different hydroxylation patterns of the FB_1_ – FB_4_ backbones results from the activity of the *FUM2* and *FUM3*-encoded oxygenases (Fig. 1C, STable 3). The *Fusarium FUM* cluster also includes a Zn_2_Cys_6_ transcription factor gene (*FUM21*) that positively regulates transcription of the cluster genes, two transporter genes (*FUM11* and *FUM19*), and two ceramide synthase genes (*FUM17* and *FUM18*) [26]. Although, *FUM15* and *FUM16* likely encode modifying enzymes based on their sequence-based predicted functions (STable 3), functional analysis in *F. verticillioides* indicates that neither gene is required for production of FB_1_ – FB_4_ [13, 24, 27, 28].

Among fumonisin-producing *Fusarium* species, the *FUM* cluster is largely conserved with respect to gene content and syntenic arrangement [13, 21, 29]. However, distribution of the cluster and, therefore, fumonisin production is highly discontinuous among *Fusarium* species, and phylogenetic relationships of homologous *FUM* genes are not concordant with the species phylogeny, suggesting a complex evolutionary history involving possible horizontal transfer and/or duplication events, and extensive loss of the cluster [21, 27]. Homologs of the *FUM* cluster have also been described in other FB-producing ascomycetes, including *A. niger* and *A. welwitschiae* [30–32], and *T. inflatum* [33], but these cluster homologs contain only 11 of the 16 *Fusarium* genes (*FUM1*, *FUM3*, *FUM6* – *FUM8*, *FUM10*, *FUM13* – *FUM15*, *FUM19*, and *FUM21*).

To our knowledge, there is no formal published report of an AAL toxin biosynthetic gene cluster. However, preliminary reports of the cluster exist [34, 35], and a 124 kb region containing a putative AAL cluster in *A. arborescens* strain Asp-27 has been deposited into the GenBank database (Accession AB969680.1). *FUM* cluster homologs have also been identified in three *Bipolaris* species *(B. maydis*, *B. zeicola*, and *B. sorokiniana)* [36]. To our knowledge, however, production of fumonisins, AAL toxins, or structurally similar SAMs has not been reported in any *Bipolaris* species.

The objective of the current study was to identify homologs of fumonisin and AAL toxin biosynthetic gene clusters from fungal genome sequences in public and in-house databases. We sought to trace the evolutionary history of the AAL and *FUM* clusters and to investigate how changes in gene content, sequence, and function impact the chemical structures of fumonisins and AAL toxins. The results support a common evolutionary origin of the fumonisin and AAL toxin clusters and provide evidence for a potential horizontal transfer of the cluster from the Sordariomycetes to the Dothideomycetes. The results also indicate that structural differences between and within the two SAM families have arisen through variation in *FUM* gene content and function.

## Materials and methods

### Genome assembly, annotation, and synteny analysis

NCBI accessions of genome sequence data for fungi with *FUM* genes examined in this study are listed in STable 1. Genome sequence data sequenced for two *A. arborescens* strains (NRRL 20593 and NRRL 66744) and *T. cylindrosporum* strain IBT 41712 were generated during the current study as these species are known to produce a SAM analog, but high-quality genomes were not publicly available. Illumina reads were generated for these genomes using a MiSeq platform (Illumina, Inc., U.S.A.) at USDA ARS NCAUR in Peoria, Illinois, U.S.A. To prepare genomic DNA for sequencing, strains were grown in liquid GYP medium (2% glucose, 1% peptone, and 0.3% yeast extract) for 2–3 days. The resulting mycelia were harvested by filtration, lyophilized, and ground to a powder. Genomic DNA was extracted from ground mycelia using the Genomic-Tip 20/G protocol (QIAGEN, Germany). The resulting DNA was used to prepare sequencing libraries with the Nextera XT DNA Library Preparation Kit. MiSeq-generated sequence reads were imported into CLC Genomics Workbench version 8.0 or 12.0 (CLC Bio-QIAGEN, Denmark), screened against genome sequences of 84 bacterial species to remove contaminating bacterial DNA, trimmed to remove low-quality data at the ends, and then subjected to *de novo* assembly using default parameters in CLC Genomics Workbench. For automated gene predictions, we used the program Augustus [37].

A long-read chromosomal scale assembly using a combined PacBio and Hi-C approach was generated for *A. arborescens* strain NRRL 66744 to examine the possible genomic location of the AAL cluster on a conditional dispensable chromosome (CDC). PacBio sequencing was performed at the Mayo Clinic Core Sequencing Facility (Rochester, MN, U.S.A.) using a 20 kb insert library prepped with the SMRTBell Express Template kit 2.0 and sequenced with the Sequel Sequencing kit 3.0 on a single SMRT cell. Sequence reads were imported into Canu [38] for *de-novo* assembly followed by polishing twice with PacBio reads using *arrow* implemented in SMRTlink6.0 (Pacific Biosciences of California Inc, U.S.A.) and once with Illumina reads generated as above using pilon2 [39]. The resulting assembly consisted of 34.32 Mb in 25 contigs, with an N50 of 3.12 Mb. This PacBio assembly was further scaffolded using Hi-C Chromatin conformation capture data generated using a Proximo Hi-C Fungal Kit (Phase Genomics, Seattle, WA, U.S.A.) [40]. Molecules were pulled down with streptavidin beads, processed into an Illumina-compatible sequencing library, and sequenced on an Illumina HiSeq 4000, generating a total of 28,943,907 PE150 read pairs. Reads were aligned to the draft PacBio assembly following the manufacturer’s recommendations using BWA-MEM with the − 5SP and -t 8 options specified, and all other options default [41]. SAMBLASTER [42] was used to flag PCR duplicates, which were later excluded from analysis. Alignments were then filtered with SAMtools [43] using the -F 2304 filtering flag to remove non-primary and secondary alignments. The Phase Genomics Proximo Hi-C genome scaffolding platform was used to create chromosome-scale scaffolds from the corrected assembly as previously described [44]. Approximately 22,000 separate Proximo runs were performed to optimize the number of scaffolds and scaffold construction in order to make the scaffolds as concordant with the observed Hi-C data as possible. Finally, Juicebox [45] was used to correct scaffolding errors, resulting in a final set of 11 scaffolds with a total genome size of 32.078 Mbp, a scaffold N50 of 3.12 Mbp and a scaffold N90 of 2.36 Mbp. The genome was annotated using Funannotate 1.8.15 [46] using both Swissport proteome database and the protein and transcriptome of the closely related *Alternaria alternata* strain SRC1_rK2f genome (GenBank Accession LXPP00000000) as evidence and the pre-trained Augustus model for *Aspergillus fumigatus*.

Genome sequence data for *A. arborescens* strain EGS 39–128, *Bipolaris* species, *T. inflatum* strain NRRL 8044 were downloaded from NCBI and uploaded into an in-house database in CLC Genomics Workbench. Genome sequence data for *A. welwitschiae* strain ITEM 11945 were obtained as previously described [31] and also uploaded to the in-house database. For other *Aspergillus* species, individual contigs with *FUM* genes were obtained from the JGI Mycocosm database. Genome sequence data for other fungi included in this study were examined using the NCBI and Joint Genome Institute (JGI) Mycocosm DNA/protein sequence databases. The arrangement of genes within *FUM* cluster were determined by aligning contig sequences with *FUM* gene sequences in the program Sequencher (Gene Codes Corporation, U.S.A.). An all-versus-all BLASTN search was conducted between the genome sequences of *A. arborescens* strain EGS 39–128 and the NRRL 20593 and NRRL 66744 genomes to identify contigs with hits with e-value = 0 and length > 1000 bp to the *FUM* cluster in NRRL 66744. These contigs were then aligned to contig 1908 of strain NRRL 66744 and the alignments visualized using Progressive Mauve [47] and Geneious Prime 2023.2.1 (https://www.geneious.com).

### Identification of *FUM* cluster homologs in public databases

We used the basic local alignment search tool functions BLASTp and BLASTx using each of the 16 genes from the *FUM* cluster in *F. verticillioides* as query against the NCBI nr database in order to identify homologs in other fungi. The top 50 BLAST hits to each FUM gene were aligned using MAFFT with the FFT-NS-2 method and default parameters [48] and a phylogeny was constructed using RAxML [49] with an optimally determined number of bootstrap values and the best fit model for each gene determined by PROTEST [50]. Midpoint rooting was used to root the trees. Both BLASTp and BLASTn were also used to search for *FUM* cluster homologues in the genomes sequenced for this study (*A. arborescens* strains 66744 and 20593, *A. welwitschiae* (ITEM 11945), and *T. cylindrosporum* (IBT 41712), as well as previously sequenced strains of *A. arborescens* (EGS 39–128) and *T. inflatum* (NRRL 8044).

### Gene and species tree phylogenetic analyses

Predicted amino acid sequences of *FUM* and housekeeping genes were aligned using the program MUSCLE [51] with default parameters as implemented in MEGA7 [52] and the FFT-NS-2 progressive method in MAFFT [48], respectively. Phylogenetic trees were inferred from the aligned amino acid sequences using the program IQ-Tree which combines a hill-climbing approach and a stochastic perturbation method to infer maximum likelihood trees and included the ultra-fast bootstrapping method [53, 54]. For all trees, 1000 bootstrap replicates were used. The species tree was inferred from concatenated alignments of 16 housekeeping genes (STable 5). In addition to the ultrafast bootstrap analysis, branch support was assessed by gene concordance analysis as implemented in IQ-Tree [55] and Extended Majority Rule analysis as implemented in RAxML [56, 57]. Housekeeping gene sequences were identified by BLASTn or BLASTx using default parameters and *Fusarium* housekeeping gene sequences as queries against genome sequence data in CLC Genomics Workbench, the NCBI nr database, or the JGI database. Coding region sequences of some orthologs of housekeeping genes differed markedly from most other orthologs. In such cases, the divergent coding regions were manually annotated.

Reconciliation analysis employed the program NOTUNG version 2.9 [58]. In this analysis, individual *FUM* gene trees were reconciled to the species tree described above. Constraint analysis employed the Shimodaira-Hasegawa and Approximately Unbiased tests to assess whether constrained trees were less well supported than unconstrained trees [59, 60]. In the analysis, individual *FUM* gene trees were constrained so that they included a *Fusarium*-*Tolypocladium* clade (i.e., Sordariomycetes clade) that was absent in most *FUM* gene trees but was well supported in the species tree. For *FUM1*, we tested two constraints: (1) a *Fusarium*-*Tolypocladium* clade, which was well supported in the species tree; and (2) a *Tolypocladium*-*Alternaria*-*Bipolaris* clade, which did not exist in the *FUM1* tree but was well supported in most other *FUM* gene trees.

### Deletion of *FUM8* in *T. inflatum*

A genetic knockout of the homolog of *FUM8* (TINF_02668) in the *T. inflatum* strain NRRL8044 was created using an established split-marker approach for protoplast mediated transformation for filamentous fungi [61] (SFile 1). Flanking regions of ~ 800 bp on either side of the coding gene were amplified using Takara EX-Taq (Takara Bio USA Inc., U.S.A.) and the gene expressing hygromycin resistance was amplified from the pCX62 plasmid for use in the split-marker protocol. After transformation, plates were allowed to grow overnight in regeneration media and were then overlaid with regeneration media containing 250 ug/mL of HYG for a final concentration of 125 ug/mL for selection. Two transformants were identified by PCR screening of the left and right integration sites to have the HYG resistance construct replacing the Fum8 gene (SFile 1). These two transformants as well as one ectopic strain were screened for production of fumonisins using mycotoxin production assay described below.

### Deletion of *FUM15* in *F. oxysporum*

A genetic knockout of Fum15 was generated in a highly hydroxy-FC_1_-producing strain of *Fusarium oxysporum* (NRRL 39464; FRC O-1890, CAR1) [11]. Strains were routinely maintained on V8 juice agar medium and grown at 28 °C. Deletion of the *FUM15* ortholog in *F. oxysporum* was done using a previously described protoplast-mediated transformation method using fusion PCR (SFile 2) [27]. Glycerol stocks of fungal strains were maintained at −80 °C. For fumonisin analysis, the fungus was grown on V8 medium for 7 days. A ~ 0.5 cm plug taken from the resulting culture was used to inoculate 4 g of cracked maize kernel medium in a 4-dram vial. The cultures were allowed to incubate at room temperature in the dark for 10 days, and three replicate cultures of each strain were analyzed. Fumonisins were extracted using previously described methods [62] with minor modifications. Briefly, fumonisins were extracted by adding acetonitrile: water (1:1, v/v) into the 4 mL dram vials containing cracked maize kernel medium cultures. The solvent-culture was allowed to incubate for 3 h at room temperature with mild shaking. After this incubation, 1 mL of extract was collected from each sample and analyzed via LC-MS using the methods previously described [63].

### Site-directed mutagenesis of Fum8 position 580

Site-directed mutagenesis of *FUM8* was performed in vitro by making a single-nucleotide change in the codon of the *FUM8* coding region corresponding to Fum8 position 580 (SFile2). For the mutagenesis we used two previously described plasmids, one carrying the *FUM8* gene from *F. oxysporum* (*FoFUM8*) strain NRRL 39464 (= FRC O-1890), and the other carrying the *FUM8* gene from *F. verticillioides* (*FvFUM8*) strain NRRL 20956 (= FRC M-3125 = FGSC 7600=) [34, 95]. The cloned *FoFUM8* gene was mutated by GenScript Biotech Corporation (China) using a custom mutagenesis method. The cloned *FvFUM8* was mutated using the In-Fusion HD Cloning Plus Kit (Takara Bio USA Inc., U.S.A.) following protocols described by the manufacturer, except that Q5 High-Fidelity DNA Polymerase (New England Biolabs Inc., U.S.A.) was used as the polymerase in PCR amplifications. For *FoFUM8* mutagenesis, the nucleotide sequence of the codon was changed from GTC, which specifies valine, to GCC, which specifies alanine (V580A mutation). For *FvFUM8* mutagenesis, the nucleotide sequence of the codon was changed from GCC, which specifies alanine, to GTC, which specifies valine (A580V mutation). The presence of the single nucleotide changes in the *FoFUM8* V580A and *FVFUM8* A580V clones was confirmed by Sanger sequencing using the BigDye Terminator version 3.1 method [20].

The plasmids carrying mutagenized *FoFUM8* or *FvFUM8* were introduced separately into a previously described strain of *F. verticillioides* in which the *FUM8* gene had been inactivated by additive gene disruption (i.e., *fum8* mutant strain GfA3245) [96]. Each plasmid was introduced into the mutant separately using a previously described protoplast-mediated transformation method [97]. The presence of individual *FUM8* plasmids in the resulting *F. verticillioides* transformants was confirmed by PCR analysis, and amplification products were subjected to Sanger sequencing (see above) to confirm the presence of the mutated *FUM8* codon corresponding to Fum8 position 580. Five transformants with the plasmid carrying the *FoFUM8* V580A mutation and five transformants with the plasmid carrying the *FvFUM8* A580V mutation were assessed for fumonisin production. As controls, we also assessed fumonisin production in the original *fum8* mutant, the *fum8* mutant complemented with a plasmid carrying the wild-type *FoFUM8* gene, and the *fum8* mutant complemented with a plasmid carrying the wild-type *FvFUM8* gene [34]. For comparison, we also assessed fumonisin production in the wild-type strains of *F. oxysporum* and *F. verticillioides*.

##  AAL toxin and fumonisin production assays

AAL toxin production in *A. arborescens* and fumonisin production in other fungi were assessed by growing strains on rice kernel (4.4-g polished rice and 1.8-ml water) and cracked maize kernel (2.5-g crack maize kernels and 1.2-mL water) media, respectively. The ingredients for both media were placed in 8-dram vials, which were then autoclaved for 20 min at 120 °C and subsequently cooled to room temperature prior to inoculation with an approximately 3-mm^2^ piece of agar-media cultures of the strains. After incubation of the cultures in the dark at room temperature for 12 days, 12-mL of 1:1 (v/v) acetonitrile: water was added to each culture and mixed with a plastic implement to break up the fungal growth and culture medium. The resulting mixture was then shaken at 150 rpm for at least 2 hours. An aliquot of the acetonitrile: water extract was centrifuged at 16,000 × g and then stored at 4 °C until LC-MS analysis. The LC-MS system consisted of a Thermo Dionex Ultimate 3000 chromatography system coupled to a Thermo QExactive high resolution tandem mass spectrometer (ThermoScientific). LC analysis employed a 50 mm x 2 mm Luna C18 column (Phenomenex) and the following solvent system: a 40 to 95% aqueous methanol gradient over 5 min and a flow rate of 0.6 mL/min. MS analysis employed an electrospray ionization interface operated in positive ionization mode. Ten-µL aliquots of the acetonitrile: water extract were injected into the LC. Detection of AAL toxin and fumonisin analogs was based on presence of the following [M + H] + ions: AAL toxin TA, m/z 522; AAL toxin TB, m/z 506; FB_1_ m/z 722, FB_2_ and FB_3_, 706; and FB_4_, m/z 690. Quantification of these analogs was based on purified standards. Thermo Xcalibur LC-MS software was used to control the LC-MS system and evaluate data.

## Results

### Distribution of *FUM* genes

To identify *FUM* cluster homologs in fungi, we used *F. verticillioides FUM* gene and protein sequences as queries in BLASTn, BLASTp and/or BLASTx searches against public and in-house fungal genome sequence databases. We then conducted phylogenetic analysis of sequences of the resulting BLAST hits to identify genes that were most closely related to and formed a monophyletic group with *F. verticillioides* and *A. niger FUM* gene homologs. The databases used in the BLAST analyses were (1) the NCBI nr fungal database, (2) the MycoCosm database at the Joint Genome Institute, and (3) an in-house database consisting of genome sequence data downloaded from NCBI or MycoCosm and newly generated genome sequences for the current study (STable 1). Strains included in the latter database were sequenced because they were shown here and/or previously to produce fumonisins or AAL toxins [18, 31] or to contain a gene with sequence homology to the AAL toxin synthase gene, *ALT1* [64, 65].

**Fig. 2:**
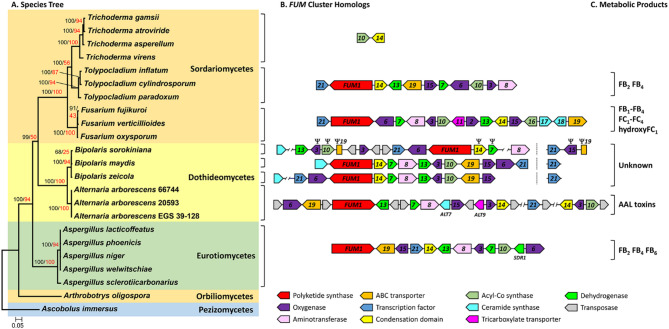
Gene content and syntenic relationships of *FUM* cluster homologs detected in fungi. **A** Species tree for taxa in which a full or partial *FUM* cluster was detected. The tree was inferred by maximum likelihood analysis of a concatenated alignment of predicted amino acid sequences of 16 housekeeping genes. Individual gene trees of the 16 housekeeping genes and an extended majority rule tree generated from these trees are shown in SFigure 5. The labels on branches are bootstrap values based on 1000 replications (black) and gene concordance values (red). The tree was rooted with representative species of two early diverging lineages of the subphylum Pezizomycotina: *Arthrobotrys oligospora* in Class Orbiliomycetes and *Ascobolus immersus* in Class Pezizomycetes. **B** Content and arrangement of genes in *FUM* cluster homologs. **C** Metabolic products of *FUM* cluster homologs

We detected a *FUM* cluster homolog consisting of the same subset of 11 *FUM* genes homologs (*FUM1*, *FUM3*, *FUM6*, *FUM7*, *FUM8*, *FUM10*, *FUM13*, *FUM14*, *FUM15*, *FUM19* and *FUM21*) in multiple fungal genera in three classes of the subphylum Pezizomycotina (Table 1, SFigure 1): *A. arborescens*, *B. maydis* and *B. zeicola* in the Dothideomycetes; *Aspergillus niger*, *A. lacticoffeatus*,* A. phoenicis*, *A. sclerotiicarbonarius* and *A. welwitschiae* in the Eurotiomycetes; and *Tolypocladium cylindrosporum*, *T. inflatum* and *T. paradoxum* in the Sordariomycetes. BLASTn analysis of the *B. sorokiniana* genome revealed the presence of sequences homologous to the 11 *FUM* genes, but subsequent manual annotation revealed that some of these genes (*FUM3*, *FUM7*, *FUM10*, *FUM14*, *FUM15*, and *FUM19)* contained deletions, insertions, and/or base substitutions that would most likely result in nonfunctional proteins (Table 1). In most of these * FUM *cluster homologs, only one copy of each gene was typically detected. However, the three *Bipolaris* species had two *FUM21* paralogs; *B. sorokiniana* had one apparently functional and one pseudogenized copy of *FUM3*; and *A. arborescens* had two copies of *FUM3* and *FUM14* (Table 1; Fig. 2, SFigure 1). As previously observed among *Fusarium* species, these *FUM* cluster homologs were discontinuously distributed in *Alternaria* and *Tolypocladium* species. They were detected in *T. inflatum*, *T. cylindrosporum*, and *T. paradoxum*, for example, but not in the closely related species *T. ophioglossoides.* Similarly, all four strains of *A. arborescens* analyzed contained a putative *FUM* cluster homolog, which was absent from closely related *Alternaria* species. In previous studies [34, 66, 67], putative AAL toxin biosynthetic genes were referred to as *ALT* genes. Due to their sequence homology to *FUM* genes, we will hereafter refer to them using the corresponding *FUM* designations. Two exceptions are *ALT7* and *ALT9*, which despite belonging to the same gene families as *FUM17/FUM18* and *FUM11*, respectively, were relatively distantly related to the *FUM *genes (see below). Homologs of the 16 *F. verticillioides FUM* genes were found in multiple species of *Fusarium*, but because these are well characterized, we included genes from only a single strain of each of three species (*F. fujikuroi* IMI 58289, *F. oxysporum* FRC O-1879, and *F. verticillioides* FGSC 7600) in subsequent analyses.

We detected one or a few *FUM* gene homologs in additional fungi. Homologs of *FUM10* and *FUM14* were located adjacent to one another in *Trichoderma asperellum*, *T. atroviride*,* T. gamsii*, and *T. virens* (Table 1, SFigure 1). Homologs of only *FUM1* were detected in multiple *Aspergillus* species in section *Nigri*, and both *FUM1* and *FUM15* were detected in *A. neoniger* (Table 1, SFigure 1). The presence of partial *FUM* clusters in some *Aspergillus* spp. has been reported previously [31], but as far as we are aware, this is the first report of a partial *FUM* cluster in *A. costaricaensis*, *A. eucalypticola*, *A. neoniger*, and *A. sclerotioniger*. A homolog of *FUM21* was detected in *Bipolaris victoriae* 28538 and *B. oryzae* 105204. Although a BLAST hit to *FUM1* was detected in the fungus *Glarea lozoyensis* (Class Leotiomycetes), this PKS gene is part of the pneumocandin biosynthetic cluster [68] and our phylogenetic analyses revealed that it fell outside the clade containing the other *FUM1* homologs (SFigure 1) [68]. Thus, the *G. lozoyensis FUM1* homolog most likely belong to a closely related PKS family not involved in fumonisin biosynthesis.

**Table 1:** *FUM*-gene content in fungi containing a cluster with homologs of 11 *F. verticillioides FUM* genes. Although they do not have a demonstrated role in AAL toxin or Fumonisin biosynthesis, the distribution of the genus/class-specific, *FUM* cluster-associated genes *ALT7*, *ALT9* and *SDR1* are also shown

Species	*FUM* Gene																		
	1	2	3	6	7	8	10	11	13	14	15	16	17	18	19	21	ALT7	*ALT9*	*SDR1*
*Alternaria arborescens*	X		**2 **X	X	X	X	X		X	**2 **X	X				X	X	X	X	
*Aspergillus lacticoffeatus*	X		X	X	X	X	X		X	X	X				X	X			X
*Aspergillus niger*	X		X	X	X	X	X		X	X	X				X	X			X
*Aspergillus phoenicis*	X		X	X	X	X	X		X	X	X				X	X			X
*Aspergillus sclerotiicarbonarius*	X		X	X	X	X	X		X	X	X				X	X			X
*Aspergillus welwitschiae*	X		X	X	X	X	X		X	X	X				X	X			X
*Bipolaris maydis*	X		X	X	X	X	X		X	X	X				X	**2 **X	X		
*Bipolaris sorokiniana*	X		**2 **X	X	**Ψ**	X	**Ψ**		X	**Ψ**	**Ψ**				**Ψ**	**2 **X	X		
*Bipolaris zeicola*	X		X	X	X	X	X		X	X	X				X	**2 **X	X		
*Fusarium fujikuroi*	X	X	X	X	X	X	X	X	X	X	X	X	**Ψ**	X	X	X			
*Fusarium oxysporum*	X	X	X	X	X	X	X	X	X	X	X	X	X	X	X	X			
*Fusarium verticillioides*	X	X	X	X	X	X	X	X	X	X	X	X	X	X	X	X			
*Tolypocladium cylindrosporum*	X		X	X	X	X	X		X	X	X				X	X			
*Tolypocladium inflatum*	X		X	X	X	X	X		X	X	X				X	X			
*Tolypocladium paradoxum*	X		X	X	X	X	X		X	X	X				X	X			

X indicates an apparently functional homolog of the corresponding gene was detected. A white cell indicates that the gene was not detected. A number in a cell indicates the number of paralogs of a gene that were detected in a genome. However, the *B. sorokiniana* sequence data indicated that one of the *FUM3* paralogs was a pseudogene. The Greek letter Psi (Ψ) indicates that DNA sequence of the corresponding gene was detected, but the gene was pseudogenized as evidenced by nucleotide insertions and/or deletions

### *FUM* cluster homologs in relation to species phylogeny

To assess how gene content and organization in *FUM* cluster homologs varied with species phylogeny, we inferred a species tree using maximum likelihood analysis of sixteen housekeeping genes from taxa in which two or more *FUM* genes were detected (Fig. 2A). Because all the *Aspergillus* species with *FUM* genes were closely related members of section *Nigri*, we excluded *Aspergillus* species with a partial *FUM* cluster from this analysis. The species tree was rooted with two early diverging species of the subphylum Pezizomycotina, *Ascobolus immersus* (Class Pezizomycetes) and *Arthrobotrys oligospora* (Class Orbiliomycetes). The topology of the resulting species tree generally reflected currently accepted phylogenetic relationships among classes of the Pezizomycotina, except that it failed to support a sister relationship between Dothideomycetes and Eurotiomycetes, a relationship that has historically lacked strong bootstrap support in multigene phylogenies [69, 70].

The content and arrangement of genes within *FUM* cluster homologs differed markedly among genera and were not necessarily correlated with phylogenetic relationships of species (Fig. 2B). For example, the *Tolypocladium FUM* clusters had only the core set of 11 *FUM* genes noted above, whereas *FUM* clusters in other genera included at least one additional gene not shared with other genera. The consensus *Fusarium FUM* cluster included five genes (*FUM2*, *FUM11*, *FUM16*, *FUM17* and *FUM18*) that were absent in other genera (Table 1; Fig. 2B). The *Aspergillus FUM* clusters included the short-chain dehydrogenase/reductase gene *SDR1* (Fig. 2B) [31], while the *Bipolaris* species and *A. arborescens FUM* clusters included *ALT7*, a putative ceramide synthase gene [34]. Phylogenetic analysis revealed that *ALT7* was not closely related to the ceramide synthase genes (*FUM17* and *FUM18*) in the *Fusarium FUM* cluster (SFigure 2). The *A. arborescens* cluster also included *ALT9*, which is predicted to encode a mitochondrial tricarboxylic acid (TCA) transporter that is relatively distantly related to the TCA transporter gene, *FUM11*, in the *Fusarium FUM* cluster (Fig. 2B, SFigure 3). Thus, the gene content of *FUM* cluster homologs in *Fusarium* and *Tolypocladium*, which are in the same fungal class, differed by five *FUM* genes, whereas the gene content of *FUM*-cluster homologs in *Tolypocladium* and *Aspergillus*, which are in different classes, differed only by the presence or absence of *SDR1* (Fig. 2B).

Although the gene content and arrangement of *FUM* cluster homologs differed among genera, content and arrangement within genera of the Sordariomycetes (*Fusarium*, *Tolypocladium*) and within genera of the Eurotiomycetes (*Aspergillus*) were mostly conserved (Fig. 2B). In contrast, *FUM* cluster homologs among Dothideomycetes (*Bipolaris and Alternaria)* were more variable and rearranged (Fig. 2B). For example, most *FUM* genes were arranged contiguously, or nearly so, in a 40–43 kb region in *B. maydis* and *B. zeicola*, but in *B. sorokiniana* the genes were dispersed across an 80–112 kb region, which included transposon sequences. In addition, the *B. sorokiniana* homologs of *FUM7*, *FUM10*, *FUM14*, *FUM15*, *FUM19*, and one *FUM3* paralog were pseudogenized. All three *Bipolaris* species also had two paralogs of the transcription factor gene *FUM21*. In *B. maydis* and *B. zeicola*, one copy was contiguous with the *FUM* cluster and the other was not. In *B. sorokiniana*, by contrast, the *FUM21* paralog that was not contiguous with the *FUM* cluster and was located 1.4 Mb away on the same scaffold and adjacent to *FUM15* and *FUM19* pseudogenes. Finally, while all three *Bipolaris* species had a homolog of the putative ceramide synthase gene *ALT7*, the gene was contiguous with the cluster in *B. maydis* but was 47–67 kb from the nearest *FUM* gene in *B. sorokiniana* and *B. zeicola*.

**Fig. 3:**
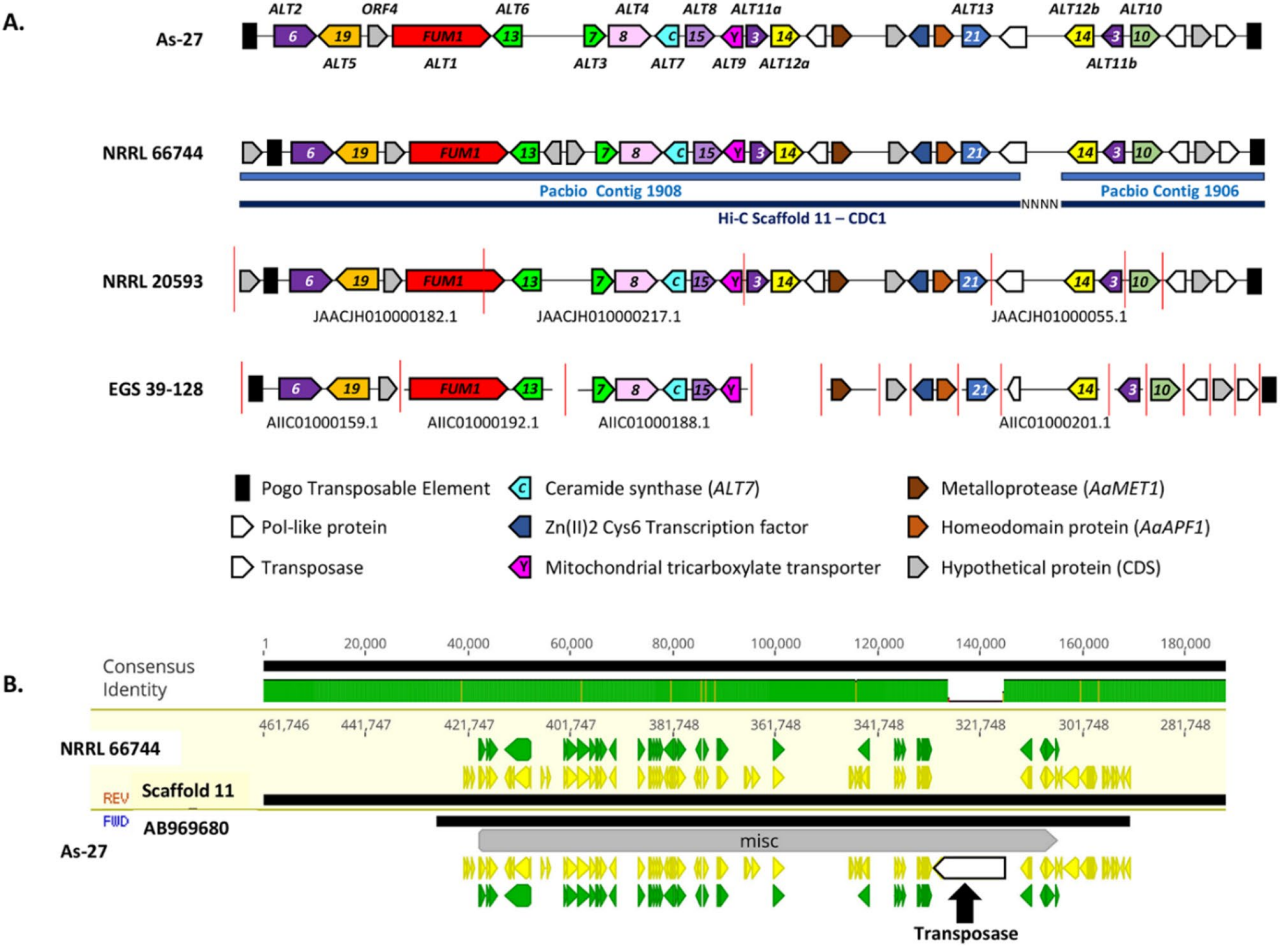
Synteny of AAL toxin clusters. **A** Content and arrangement of genes in the *FUM* cluster in four *Alternaria arborescens* strains: As-27, NRRL 66744, EGS 39-128, and NRRL 20593. Red vertical lines indicate contig boundaries in assemblies from Illumina MiSeq or GA II sequence data. Contig numbers are indicated below those contigs containing more than one *FUM* homolog. Contigs 1906 and 1908 from the PacBio sequence of NRRL 66744 were assembled into the same scaffold (Scaffold 11) using Hi-C analysis. For strains NRRL 20593 and NRRL 66744, gaps between contigs were filled in using the Map Read to Reference function in CLC Genomics Workbench to map reads to the As-27 reference cluster (SFigure 4). **B** Genious alignment of the *FUM* cluster sequence from strain As-27 (NCBI Accession AB969680.1) and the Hi-C scaffold 11 (CDC1) from strain NRRL 66744 showed high sequence identity and conservation of gene order. Areas in green above the alignment indicate high sequence similarity (100%), punctuated by variant positions or SNPs shown as vertical yellow lines. The unassembled region between PacBio contigs 1906 and 1908 in scaffold 11 of NRRL 66744 maps to a transposon sequence in the As-27 cluster, but raw reads of 66744 map to this region to fill this gap (SFigure 4). Additional transposable element sequences, including a Pogo transposable element flanks both ends of the cluster in most stains, as well as several Pol-like genes downstream of *FUM21* in the cluster-flanking region

In the genomes of *A. arborescens* NRRL 66744 and NRRL 20593, as well as the GenBank accession (AB969680.1) of the AAL cluster amplified from strain *A. arborescens* strain As-27, 10 of the 11 conserved *FUM* genes were clustered within a 91.5 kb region (Fig. 3A). However, 7 Kb away from this cluster was a 20 kb region that included *FUM10* and a second paralog of both *FUM3* and *FUM14* arranged contiguously (Figs. 2B and 3A, SFigure 4). The sequence of this region in As-27, NRRL 20593 and NRRL 66744 shared high sequence similarity and gene content, except that NRRL 66744 had two genes of unknown function between *FUM13* and *FUM7* that both As-27 and NRRL 20593 lacked (Fig. 3B).

### Conflict and reconciliation between *FUM* gene trees and species tree

**Fig. 4:**
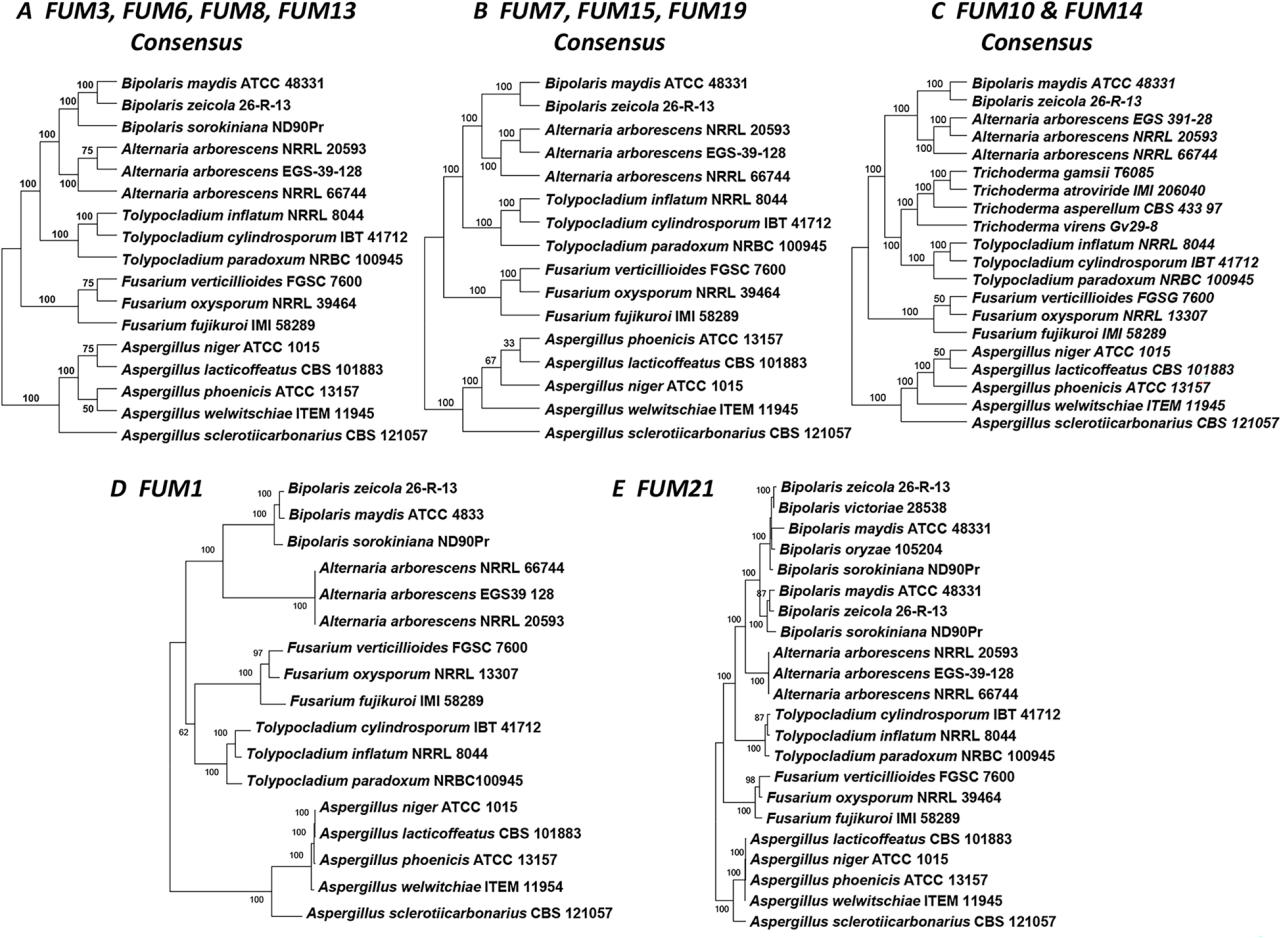
*FUM* gene trees inferred from predicted amino acid sequences. Individual *FUM* gene trees were inferred by maximum likelihood analysis using IQ-Tree (shown in **SFigure 6**). Individual trees were grouped into five groups (A – E) based on similar topology and/or taxon content to show consensus trees. For groups with more than one tree, the consensus tree was generated using the Constructing Consensus Tree process in IQ-Tree (-con command). A) Consensus tree for *FUM3*, *FUM6*, *FUM8* and *FUM13*; B) Consensus tree for *FUM7*, *FUM15* and *FUM19*; C) Consensus tree for *FUM10* and *FUM14*; D) *FUM1* tree; and E) *FUM21* tree. In consensus trees, values near branches are percent of trees with the branch indicated. In *FUM1* and *FUM21* trees, values near branches are bootstrap values generated with 1000 replications. In the *FUM1* tree, *Tolypocladium* and *Fusarium* (Sordariomycete) resolved as an exclusive clade with 62% bootstrap support. In all other *FUM* gene trees, *Tolypocladium* (Sordariomycete) resolved into a clade with *Alternaria* and *Bipolaris* (Dothideomycetes) sequences with 70 – 100% bootstrap support.

Comparison of topologies of the species tree (Fig. 2A, SFigure 5) to individual *FUM* gene trees revealed multiple conflicts (Fig. 4, SFigure 6). The species tree and individual housekeeping gene trees strongly supported the monophyly of the Sordariomycete genera *Tolypocladium*, *Trichoderma*, and *Fusarium* (Fig. 2A, SFigure 5), but in most *FUM* gene trees *Tolypocladium* formed an exclusive and well-supported clade together with the Dothideomycete genera *Bipolaris* and *Alternaria* (Fig. 4A, B, E, SFigure 6). The *FUM10* and *FUM14* trees differed in that they included *FUM* gene homologs from *Trichoderma*, which grouped together with those from *Tolypocladium* to form a strongly supported clade sister to the *Bipolaris* and *Alternaria* clade (Fig. 4D, SFigure 6). The *FUM1* tree was the only tree with a topology that mirrored the species phylogeny by grouping *Tolypocladium* and *Fusarium* together, although with only 62% bootstrap support (Fig. 4A, SFigure 6). In the *FUM21* tree, two distinct *Bipolaris* clades corresponded to the two paralogs (Fig. 4E, SFigure 6), suggesting a duplication of *FUM21* prior to divergence of *B. maydis*, *B. sorokiniana* and *B. zeicola* but after divergence from *A. arborescens.* The two paralogs of *FUM3* and *FUM14* in *A. arborescens* were identical in sequence and, thus, are likely products of a recent duplication event, as supported by NOTUNG analyses (Fig. 4, SFigs. 6 and 7).

To further examine the relationships of *Tolypocladium FUM* genes with those in *Alternaria* and *Bipolaris*, we performed Shimodaira-Hasegawa and Approximately Unbiased tests to compare the unconstrained *FUM* gene trees to gene trees in which the relationship between Sordariomycetes *Fusarium* and *Tolypocladium* was constrained to mirror the species tree [59, 60]. For all the genes except *FUM1* and *FUM7*, the original unconstrained tree received significantly higher support than the constrained tree at *p* < 0.05 (STable 4). Thus, the results of the constraint analyses provide additional evidence that most *Tolypocladium* (Sordariomycetes) *FUM* genes were more closely related to their homologs in *Alternaria* and *Bipolaris* (Dothideomycetes) than to those in *Fusarium* (Sordariomycetes). In contrast, the unconstrained *FUM1* tree mirrored the species tree and did not have significantly higher support than a tree constrained to include a *Tolypocladium*-*Fusarium* clade (STable 4).

**Fig. 5:**
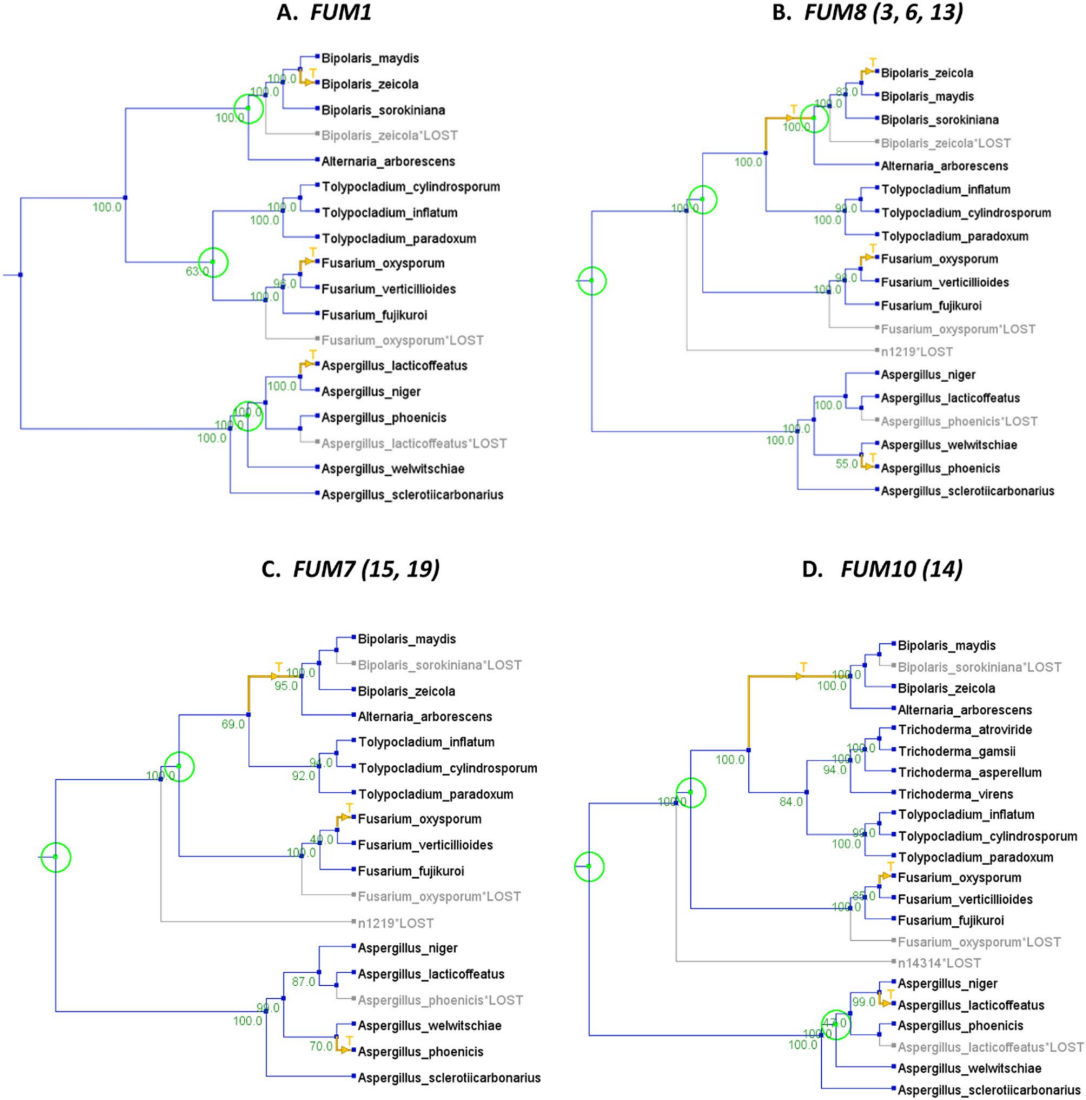
Results of NOTUNG Reconciliation analysis of individual *FUM* gene trees (SFigure 6) and the species tree (Fig. 2A) inferred from 16 housekeeping genes (SFigure 5). All individual *FUM* gene trees were subjected to this analysis, but only four scenarios are shown here: (**A**) *FUM1* tree; (**B**) F*UM8* which showed the same reconciliation as *FUM3*, *FUM6*, and *FUM13* (**C**) *FUM7* which showed the same reconciliation as *FUM15* and *FUM19*; and (**D**) *FUM10* which was similar to *FUM14*. NOTUNG trees for all individual *FUM* genes are shown in SFigure 7. In the trees, blue branches indicate vertical inheritance, yellow branches indicate horizontal transfer with arrowhead pointing in direction of transfer, gray branches indicate gene loss event, and green circles indicate alternative events were inferred to the right of the circle. NOTUNG analyses for all *FUM* genes except *FUM1* and *FUM21* support a horizontal transfer (yellow arrow) from an ancestor of *Tolypocladium* (Sordariomycetes) to an ancestor of *Alternaria* and *Bipolaris* (Dothideomycetes)

We also performed gene tree-species tree reconciliation analyses using the program NOTUNG [58]. This analysis inferred multiple and variable horizontal gene transfer (HGT) and gene duplication events of *FUM* genes (Fig. 5, SFigure 7). However, one inference that was consistent for most genes (*FUM3*, *FUM6* – *FUM8*, *FUM13*, *FUM15* and *FUM19)* was HGT from an ancestor of *Tolypocladium* in Sordariomycetes to an ancestor of *Alternaria* and *Bipolaris* in Dothideomycetes (Fig. 5, SFigure 7). Reconciliation analysis of *FUM10* and *FUM14* trees, which included *Trichoderma* species, also inferred HGT from an ancestor of *Tolypocladium* and *Trichoderma* in Sordariomycetes to an ancestor of *Alternaria* and *Bipolaris* in Dothideomycetes (Fig. 5D, SFigure 7). It is noteworthy that HGT of the core polyketide synthase *FUM1* from a *Tolypocladium* ancestor to an ancestor of *Alternaria* and *Bipolaris* was not inferred in the reconciliation analysis (Fig. 5, SFigure 7), while *FUM21* showed a horizontal transfer in the opposite direction from Dothideomycetes to Sordariomycetes (SFigure 7). Duplications of *FUM3* and *FUM14* were inferred in the ancestor of *Alternaria* and for *FUM21* in the ancestor of *Bipolaris* species (SFigure 7).

### Deletion of *FUM8* confirms a role in fumonisin production in *Tolypocladium*

While a putative *FUM* cluster homolog was previously identified in *T. inflatum* [33], its role in fumonisin production has never been functionally verified. We first used LC-MS to confirm production of fumonisins in the *T. inflatum* and *T. cylindrosporum* strains used in this study (STable 4). To assess the role of the *Tolypocladium FUM* cluster homolog in fumonisin production and to generate a mutant strain to investigate the role of *FUM8* in conferring FB versus FC production in an insect pathogenic fungus, we deleted the *FUM8* gene in *T. inflatum* strain NRRL 8044 using a split-marker approach [61]. We identified two *fum8* deletion mutants, i.e., transformants in which the *FUM8* coding region was replaced by the hygromycin resistance cassette (SFile 1). In LC-MS analysis of culture extracts, the two *fum8* deletion mutants did not produce detectable levels of fumonisins (Table 2). By contrast, the wild-type progenitor strain and an ectopic transformant produced relatively high levels of FB_2_ and FB_4_. These results provide evidence that *FUM8* is required for FB production in *T. inflatum* and, therefore, evidence that the *Tolypocladium FUM* cluster confers fumonisin production.

**Table 2:** Production of fumonisins in wild-type, *fum8* deletion mutant, and ectopic transformant strains of *Tolypocladium inflatum*

**Strain**	Fumonisin production (ng/mL)^a^			
	**FB**_1_	**FB**_2_	**FB**_3_	**FB**_4_
Control	0.0	0.0	0.0	0.0
Wild type (NRRL 8044)	0.0	20.3–44.4	0.0	73.4-101.4
*fum8* mutant 1	0.0	0.0	0.0	0.0
*fum8* mutant 2	0.0	0.0	0.0	0.0
Ectopic transformant	0.0	26.4–33.1	0.0	87.3–74.6

^a^Strains were grown in cracked maize kernel medium. The range of values from 2 replicate cultures are shown. No fumonisins were detected in either of two *fum8* mutant strains

### Sequence variation in *FUM8* determines production of B versus C-series fumonisins

Previous studies revealed a difference in the amino acid at position 580 of Fum8 (hereafter, Fum8 580) in FB versus FC-producing *Fusarium* species [11]. FB-producing species had an alanine at Fum8 580, while FC-producing species had a valine at this position [21]. In the current study, we examined the predicted amino acid residue at Fum8 580 in all fungi that had a *FUM* cluster. An alanine was present at Fum8 580 in all FB-producing species examined, whereas a valine was present at this position in all FC and AAL-toxin-producing species (Table 3). We also used LC-MS to confirm production of AAL toxin by the specific *A. arborescens* strains used in this study (STable 4). Among the fungi examined for which a chemical product of the *FUM* cluster has not been identified, as far as we are aware, some had an alanine at Fum8 580, and others had a valine (Table 3).

**Table 3:** Fumonisin/AAL toxin production phenotypes, amino acid at Fum8 position 580, and predictions of the amino acid substrate of Fum8 homologs

		Predicted Amino Acid^b^	
Species	Production^**a**^	Fum8 580	Substrate
*Aspergillus niger*	FB	Alanine	Alanine
*Aspergillus welwitschiae*	FB	Alanine	Alanine
*Fusarium fujikuroi*	FB	Alanine	Alanine
*Fusarium verticillioides*	FB	Alanine	Alanine
*Tolypocladium cylindrosporum*	FB	Alanine	Alanine
*Tolypocladium inflatum*	FB	Alanine	Alanine
*Alternaria arborescens*	AAL	Valine	Glycine
*Fusarium oxysporum*	FC	Valine	Glycine
*Fusarium anthophilum* ^c^	FC	Valine	Glycine
*Fusarium bulbicola* ^c^	FC	Valine	Glycine
*Aspergillus phoenicis*	ND	Alanine	Alanine
*Aspergillus sclerotiicarbonarius*	ND	Alanine	Alanine
*Aspergillus lacticoffeatus*	ND	Alanine	Alanine
*Bipolaris maydis*	ND	Valine	Glycine
*Bipolaris sorokiniana*	ND	Valine	Glycine
*Bipolaris zeicola*	ND	Valine	Glycine
*Tolypocladium paradoxum*	ND	Alanine	Alanine

^a^The fumonisin or AAL toxin production phenotypes of *Alternaria arborescens*, *Aspergillus niger*, *A. welwitschiae*, *Fusarium fujikuroi*, *F. oxysporum*, *F. verticillioides*, *Tolypocladium cylindrosporum* and *T. inflatum* were determined in previous studies as described in the Introduction and/or the Results of the current study (Table 2). To our knowledge the production phenotypes of the other species (listed in the lower half of this table) have not been determined. FB indicates B-series fumonisins are produced in greatest abundance; FC indicates C-series fumonisin are produced in greatest abundance; AAL indicates AAL toxin production; and ND indicates that the fumonisin/AAL toxin production phenotype has not been reported

^**b**^Fum8 580 indicates the deduced amino acid at residue 580 of the Fum8 protein. Position 580 is based on the *F. verticillioides* Fum8 homolog. Substrate indicates the Fum8 amino acid substrate. For species in which the fumonisin or AAL toxin production phenotype has been determined, predictions of the amino acid substrate of Fum8 was based on precursor feeding experiments and/or chemical structures of fumonisins and AAL toxins (Fig. 1). For species in which fumonisin/AAL toxin has not been determined, the amino acid substrate of Fum8 was predicted based on the amino acid (alanine or valine) at Fum8 residue 580

^**c**^Information for *F. anthophilum* and *F. bulbicola* is from a previous study [21]

**Fig. 6:**
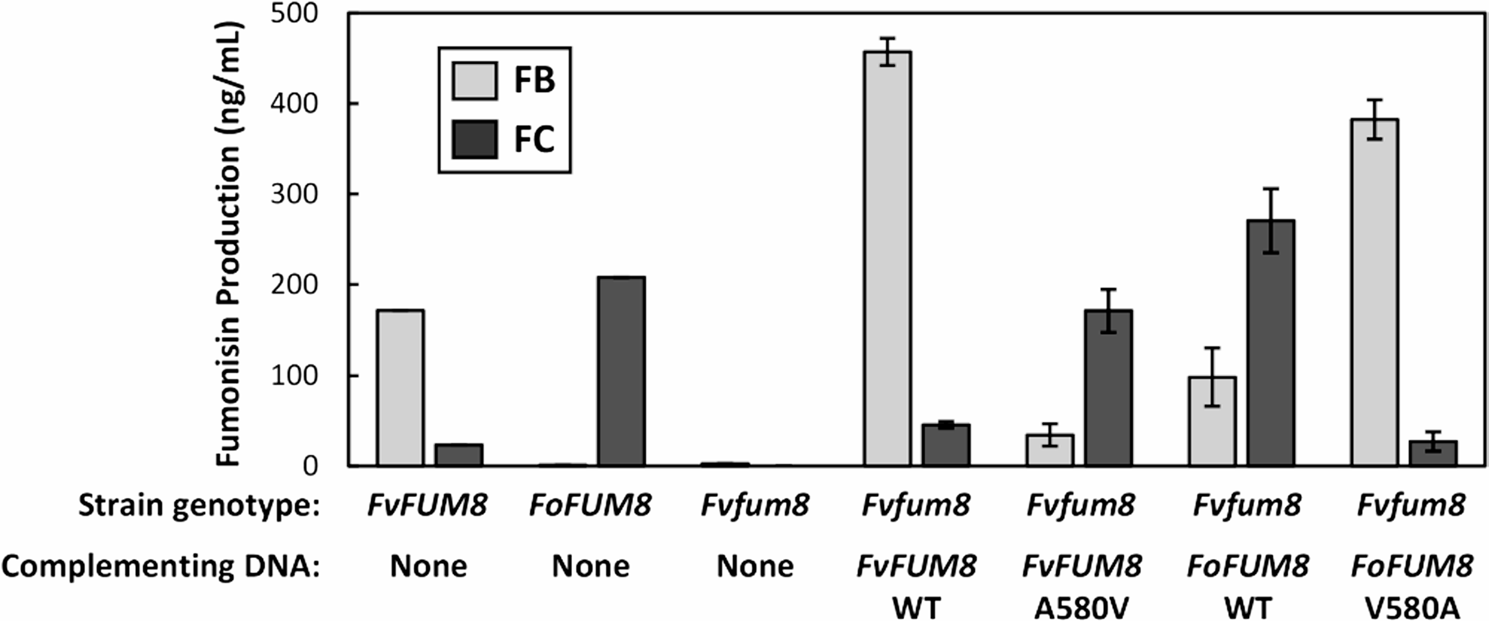
Effects of changing the amino acid at position 580 of *Fusarium* Fum8 proteins on production of B (FB) versus C (FC) fumonisins. Site-directed mutagenesis was used to change a single nucleotide in the coding region of the *Fusarium verticillioides* (*Fv*) and *F. oxysporum* (*Fo*) *FUM8* genes. In *FvFUM8*, the single-nucleotide change switched the specificity of the codon corresponding to Fum8 position 580 (Fum8 580) from alanine to valine (A580V); whereas in *FoFUM8*, the single-nucleotide change switched the codon specificity from valine to alanine (V580A). Plasmids with the single-nucleotide changes of each *FUM8* gene were introduced by protoplast-mediated transformation into a *F. verticillioides fum8* deletion mutant, and selected transformants were then analyzed for fumonisin production. For comparison, fumonisin production was also assessed in the *fum8* mutant, the fum8 mutant complemented with wild-type (WT) *FvFUM8* or *FoFUM8*, as well as wild-type strains of *F. verticillioides* and *F. oxysporum*. Fumonisin production values are the combined concentrations of the FB analogs FB_1_, FB_2_, FB_3_ and FB_4_, or the FC analogs FC_1_, FC_2_, FC_3_, FC_4_ and hydroxy FC_1_. Production values for complementation with WT *FvFUM8* or *FoFUM8* are means based on single cultures of two independent transformants. Production values for complementation with modified *FvFUM8* or *FoFUM8* are means based on single cultures of five independent transformants

To determine whether these differences at Fum8 580 do indeed confer FB versus FC production, we performed in vitro site-directed mutagenesis of *FUM8* from an FB-producing (*F. verticillioides* FGSG 7600) and an FC-producing (*F. oxysporum* NRRL 39464) strain of *Fusarium* (SFile 2). The sequence of the *F. verticillioides FUM8* coding region was modified so that Fum8 580 was changed from alanine to valine (A580V). Likewise, the sequence of the *F. oxysporum FUM8* coding region was modified so that Fum8 580 was changed from valine to alanine (V580A). Protoplast-mediated transformation was then used to introduce plasmids carrying wild-type, modified *F. verticillioides*, or modified *F. oxysporum FUM8* coding regions into a previously described *fum8* deletion mutant of *F. verticillioides* [71]. LC-MS analysis of culture filtrates confirmed that the wild-type strains of *F. verticillioides* and *F. oxysporum* produced predominantly FBs and FCs, respectively, and that the *F. verticillioides fum8* mutant produced little or no fumonisins (Fig. 6). Transformation of the *fum8* mutant with the wild-type *F. verticillioides FUM8* (*FvFUM8*) restored FB production. In contrast, transformation of the mutant with the *FvFUM8* A580V construct resulted in production of predominantly FCs. However, the levels of FCs produced by transformants carrying the *FvFUM8* A580V construct were less than the levels of FBs produced by transformants with the wild-type *FvFUM8* (Fig. 6). Transformation of the *F. verticillioides fum8* mutant with the wild-type *F. oxysporum FUM8 (FoFUM8*) resulted in production of predominantly FCs, whereas transformation with the *FoFUM8* V580A construct resulted in production of predominantly FBs (Fig. 6). These findings support the hypothesis that Fum8 580 plays a critical role in substrate specificity of Fum8.

### Deletion of *FUM15* in *F. oxysporum*

Functional analyses of *F. verticillioides* have demonstrated that *FUM2*, *FUM3*, and *FUM6* are required for formation of hydroxyl groups along the backbone of FB_1_ – FB_4_ (Fig. 1), yet the *FUM* gene required for formation of the 3-hydroxyl group of hydroxyl-FC_1_ and the hydroxyl group at the equivalent position (4-hydroxy) in FB_6_ [72], has not been identified. The functions of only three genes *(FUM15*,* FUM16*,* FUM17*) in fumonisin biosynthesis in *Fusarium* remain unknown (STable 3). Given that *FUM15* is the only one of these three genes that is likely encode a hydroxylation enzyme (i.e., a cytochrome P450 monooxygenase or dioxygenase) (STable 3), we hypothesized that *FUM15* could be required for formation of the 3-hydroxyl group of hydroxy-FC_1_. To test this hypothesis, we deleted *FUM15* in the hydroxy-FC1-producing *F. oxysporum* strain NRRL 39464 using the same strategy used to delete the *AOD1* gene in *F. babinda* and *F. tricinctum* [27] (SFile 3). LC-MS analysis indicated that both wild-type strain NRRL 39464 and the *fum15* deletion mutant derived from it both still produced hydroxy-FC_1_ (SFile 3).

## Discussion

### Distribution of Fumonisin and AAL toxin biosynthetic clusters

The results of the current study provide insights into the distribution of the *FUM* cluster and fumonisin and AAL toxin production in fungi. Detection of the *FUM* cluster only in certain species of *Alternaria*, *Aspergillus*, *Bipolaris*, *Fusarium* and *Tolypocladium* indicates the cluster has a limited and discontinuous distribution (Table 1; Fig. 2B). This finding is consistent with reports of production of fumonisins or AAL toxins only in certain species of these same genera, except for *Bipolaris* species. However, the presence of only 1–3 *FUM* genes in some *Aspergillus* and *Trichoderma* species, as well as in *B. oryzae* and *B. victoriae* (Fig. 2) [31, 73] raises the possibility that an intact *FUM* cluster and fumonisin or AAL toxin production occurred previously in these fungi as well. In individual *FUM* gene trees (Fig. 4), nesting of *A. arborescens* within clades of known fumonisin-producing species of *Fusarium* and *Tolypocladium* suggests that the *FUM* and AAL toxin clusters share a single common evolutionary origin and that the AAL cluster constitutes a branch within the evolutionary history of the *FUM* cluster. We demonstrate that the *FUM* cluster in an insect pathogenic fungus (*Tolypocladium*) is responsible for synthesis of fumonisins, and although we did not conduct functional analyses of the *A. arborescens FUM* cluster, four lines of evidence support that the cluster confers AAL toxin production. First, the chemical structures of AAL toxins and fumonisins are very similar (Fig. 1A); second, the *FUM* cluster is the only biosynthetic gene cluster in *A. arborescens* with characteristics of a SAM cluster [27]; third, chemical analyses indicate that *A. arborescens* produces AAL toxins but not fumonisins [19, 74]; and fourth, a previous study demonstrated that the *A. arborescens FUM1* homolog restored fumonisin production in a *fum1* mutant of *F. verticillioides* [67].

### Role of *FUM8* in fumonisin and AAL toxin biosynthesis

Previous analyses of *Fusarium* spp. indicated that *FUM8* encodes a class II aminotransferase that catalyzes condensation of the polyketide product of Fum1 with alanine during FB biosynthesis (Fig. 1) and with glycine during FC biosynthesis [11]. The results of the *FUM8* deletion analysis in *T. inflatum* were consistent with *FUM8* having the same role in fumonisin biosynthesis in *Tolypocladium* spp. To our knowledge, *T. inflatum* is the only fungus other than *F. oxysporum* and *F. verticillioides* in which the role of *FUM8* in fumonisin biosynthesis has been examined by functional analysis. The results of site-directed mutagenesis of *Fusarium FUM8* homologs demonstrated that the amino acid residue at Fum8 580 determines whether *Fusarium* spp. produce predominantly FBs (position 580 = alanine) or FCs (position 580 = valine). These results along with previous studies examining substrate specificity of class II aminotransferases [11] are consistent with a role for Fum8 in AAL toxin biosynthesis in that the *A. arborescens* Fum8 homolog has a valine at Fum8 580 (Table 3), and AAL toxins are structurally analogous to FCs in that they lack a terminal methyl group adjacent to the amine. Thus, we propose that during AAL toxin biosynthesis, Fum8 catalyzes condensation of glycine and the polyketide product of Fum1. This proposal is consistent with previous reports indicating that glycine is a precursor of AAL toxins [74, 75].

Based on phylogenetic analyses, *FUM* cluster homologs can be divided into three lineages: (1) an *Aspergillus* lineage, (2) a *Fusarium* lineage, and (3) a *Tolypocladium*-*Alternaria*-*Bipolaris* lineage (Figs. 2 and 4, SFigure 6). The *Fusarium* and *Tolypocladium*-*Alternaria*-*Bipolaris* lineages contain the two alternative residues at Fum8 580 (alanine or valine), whereas the *Aspergillus* lineage has only the alanine residue (Table 3). The topology of the *FUM8* tree (Fig. 4A, SFigure 6) and the distribution of the two residues at Fum8 580 suggest that a change from one residue to the other has occurred multiple times during the evolutionary history of Fum8.

### Impact of variation in *FUM* gene content on structural variation of metabolites

Variation in gene content of *FUM* cluster homologs from different fungal genera has potential to affect AAL toxin/fumonisin production by impacting chemical structure of the SAMs or other aspects of biosynthesis. For example, the occurrence of the fumonisin 9- or 10-hydroxylase gene, *FUM2*, in the *Fusarium FUM* cluster is consistent with production of 9- or 10-hydroxylated fumonisin analogs (e.g., FC_1_, FC_3_ or FB_1_, FB_3_) by *Fusarium* species [62], whereas the absence of *FUM2* in *A. arborescens*, *Aspergillus* spp. and *Tolypocladium* spp. is consistent with the lack of production of 9- or 10-hydroxylated analogs of AAL toxins or fumonisins by these fungi [18, 19, 31, 76]. Similarly, the *Fum11* gene is predicted to encode a mitochondrial TCA transporter required for transport of aconitate from mitochondria to the cytoplasm where aconitate is used to form the tricarboxylate esters of fumonisins (Fig. 1C) [28]. Although *FUM11* was detected only in *Fusarium*, a distantly related TCA transporter gene, *ALT9*, was present in the *A. arborescens FUM* cluster (Fig. 2B). We hypothesize that *ALT9* has a role in AAL toxin biosynthesis that is analogous to the role of *FUM11* in fumonisin biosynthesis (Fig. 1C).

SMBCs often include a gene(s) that confers self-protection (resistance) to toxic metabolite products of the clusters. These self-protection genes can encode resistant variants of the proteins targeted by the toxic metabolites [77]. Fumonisin and AAL toxin both target the enzyme ceramide synthase, and *Fusarium FUM* clusters have two ceramide synthase genes, *FUM17* and *FUM18*. Functional analyses indicated that *FUM18*, but not *FUM17*, confers resistance to FB_1_ [78]. Although non-*Fusarium* fungi with a *FUM* cluster lacked closely related homologs of *FUM17* or *FUM18*, the *A. arborescens* and *Bipolaris FUM* clusters had a distantly related ceramide synthase gene (*ALT7*) (Figs. 2B and 3A, SFigure 2). However, previous functional analyses indicate *ALT7* does not confer resistance to AAL toxins [34]. Thus, the roles of *ALT7* and *FUM17* in AAL toxin/fumonisin biosynthesis remain to be determined.

## Proposed AAL toxin biosynthetic pathway

**Fig. 7:**
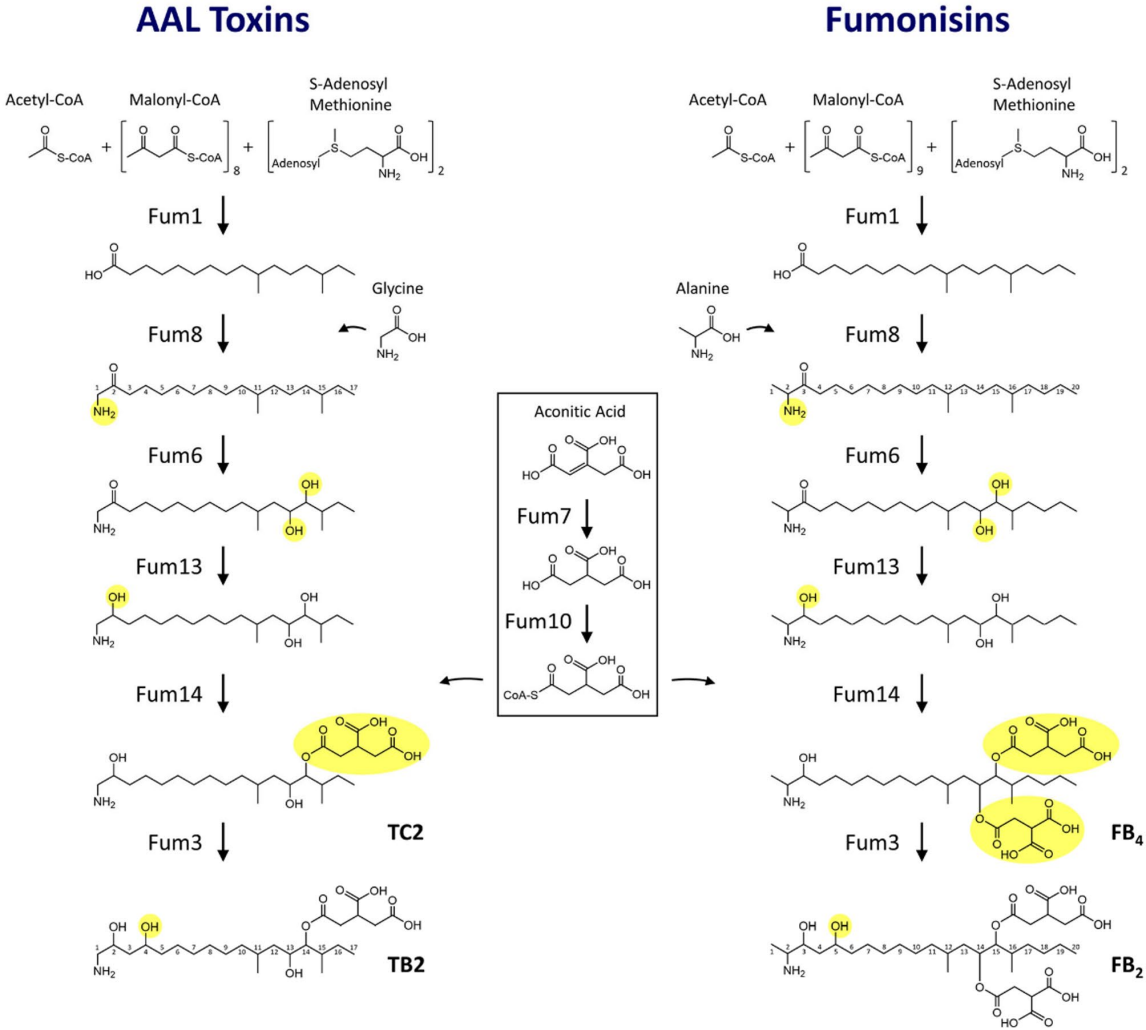
Comparison of proposed fumonisin and AAL toxin biosynthetic pathways inferred from information on functions of *FUM* genes/Fum enzymes in fumonisin biosynthesis as determined by molecular genetic and biochemical analyses of *FUM* genes in *Fusarium verticillioides* and *F. oxysporum* (STable 3)

AAL toxins differ in structure from fumonisins by having a shorter backbone and one rather than two TCA esters (Fig. 1A). Given the predicted function of *ALT7*, *ALT9* or other genes flanking the *A. arborescens FUM* cluster (Fig. 3, STable 3), we argue that these genes are unlikely to be responsible for the structural differences between AAL toxins and fumonisins. Instead, we posit that the differences in chemical structure of AAL toxins relative to fumonisins results from functional variation of one or more of the 11 *FUM* genes that *A. arborescens* shares with fumonisin-producing fungi. That the Fum1 PKS could synthesize a 16-carbon-long polyketide during AAL toxin biosynthesis rather than the 18-carbon-long polyketide synthesized during fumonisin biosynthesis is consistent with evidence that domains within PKSs can control polyketide chain length [79]. The finding that the *A. arborescens FUM1* restored fumonisin production to a *fum1* mutant of *F. verticillioides* suggests that Fum1 activity could be sufficiently flexible to produce polyketides of different chain lengths and/or that other factors may influence polyketide chain length [67]. The proposal that Fum8 is critical for release of the fumonisin polyketide from Fum1 raises the possibility that Fum8 impacts polyketide chain length and, therefore, length of the AAL toxin backbone [3]. Fum14 catalyzes esterification of TCA to two positions along the fumonisin backbone. Given this, differences in the activity of the *A. arborescens* Fum14 could be responsible for the presence of only one TCA ester in the AAL toxin structure. Using the above conjectures and our current understanding of the roles of Fum enzymes in fumonisin biosynthesis, we have proposed an AAL toxin biosynthetic pathway (Fig. 7). Further functional analyses of *A. arborescens FUM* genes should provide evidence for or against this proposed pathway.

### Inference of chemical structure of putative *Bipolaris* SAMs

Although the *FUM* cluster was previously reported in *Bipolaris* species [36], the results of the current study clarify the content and arrangement of genes in *FUM* clusters in three *Bipolaris* species (Fig. 2B). To our knowledge, production of AAL toxins, fumonisins, or a structurally similar SAM has not been reported in any *Bipolaris* species. Nevertheless, the presence of apparently functional *FUM* clusters in *B. maydis* and *B. zeicola* suggests these species have the genetic potential to produce a SAM, and the functions of *FUM* genes in fumonisin biosynthesis allow for inferences to be made about the chemical structure of this putative SAM. First, the presence of apparently functional homologs of 11 *FUM* genes in *B. maydis* and *B. zeicola* suggests these species could produce metabolites that are structurally analogous to FB_2_ and FB_4_ or the corresponding AAL toxin analogs [74, 75]. Further, the valine at Fum8 580 in *B. maydis* and *B. zeicola* suggests that a putative *Bipolaris* SAM would likely be structurally analogous to AAL toxins and FCs in lacking a terminal methyl adjacent to the amine (Fig. 1A; Table 3).

### Origin and evolutionary history of the *FUM* Cluster

Previous studies proposed hypotheses to explain the origin and evolution of the *FUM* cluster in *Aspergillus* and *Fusarium* [21, 80]. The current study provides additional insights into *FUM* cluster evolution. Below, we consider the strengths and weaknesses of scenarios for both the origin of the cluster and subsequent evolutionary processes that may have led to its current distribution and the observed variation in sequences and gene content.

#### *FUM* cluster origins

**Fig. 8:**
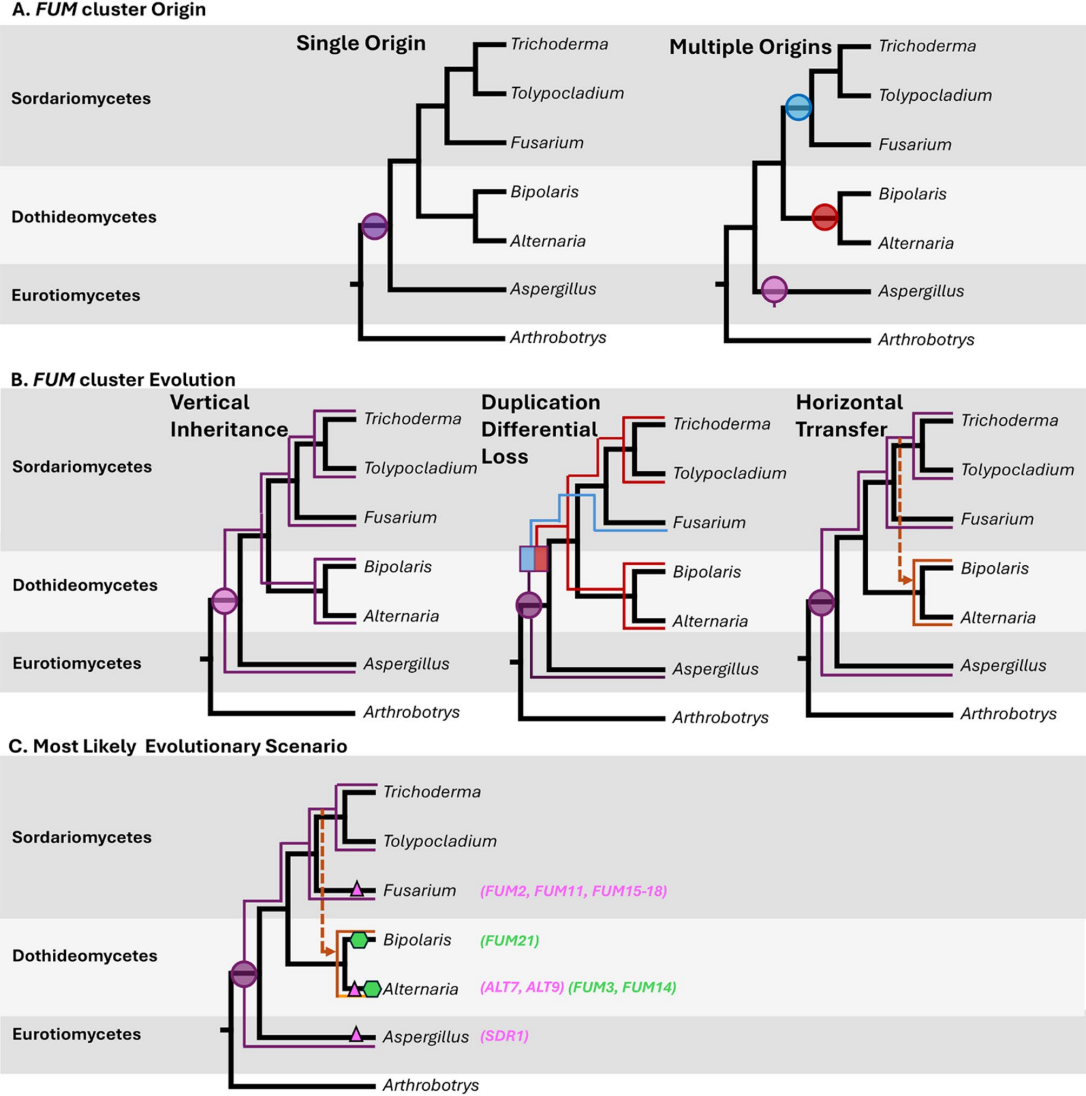
A single ancestral origin of the *FUM* cluster is a more parsimonious explanation than independent assembly in two or all three of these classes. Evolutionary scenarios for (**A**) Origin of the *FUM* cluster, (**B**) Evolutionary processes contributing to dispersal of the *FUM* cluster, and (**C**) Proposed most likely evolutionary scenario for the *FUM* cluster that hypothesizes a single origin in the common ancestor of the Pezizomycotina and that multiple evolutionary processes are responsible for its current discontinuous distribution in three classes of ascomycetes. Alternating dark and light gray backgrounds demarcate the three ascomycete classes (Dothideomycetes, Eurotiomycetes and Sordariomycetes); black solid lines denote a simplified phylogenetic tree based on the species tree in Fig. 2; colored circles indicate *FUM* cluster assembly/origin; bicolored blue/red square indicates duplication of *FUM* cluster; solid colored lines indicate vertical transmission of *FUM* cluster (with blue/red indicating one of the duplicate copies in **B**); dashed orange lines indicate horizontal transfer of the *FUM* cluster with colored arrow head indicates direction of horizontal transfer; Pink triangles indicate acquisition of lineage specific *FUM* genes; and green hexagons indicate possible duplication of individual *FUM* genes

In the Single Ancestral Origin scenario, the *FUM* cluster originated in a common ancestor of subphylum Pezizomycotina (Fig. 8A, left), while in the alternative Multiple Independent Origins hypothesis (Fig. 8A, right), first proposed by Khaldi and Wolfe [80] to explain the presence of a *FUM* cluster in *Aspergillus* and *Fusarium*, the occurrence of the *FUM* cluster in Dothideomycetes, Eurotiomycetes, and Sordariomycetes resulted from independent *de novo* assembly of the cluster in each fungal class. We argue that available evidence provides more support for the Single Ancestral Origin scenario based on conservation of the same 11 genes found in all species with a complete *FUM* cluster (Fig. 2). It seems unlikely that the same set of 11 genes would have assembled independently in three distinct classes of filamentous fungi. 

Given the current limited distribution of the cluster among fungi, the Single Ancestral Origin scenario must account for extensive loss of the cluster from many lineages within Pezizomycotina. However, previous studies in both *Fusarium* and *Aspergillus* indicate that the *FUM* cluster has been lost repeatedly by gene deletion or degeneration [21, 31, 73, 81]. In our study, each of the 5 genera in which the *FUM* cluster was detected also included species that lacked the cluster or contained partial (*Aspergillus*,* Trichoderma)* and/or pseudogenized (*B. sorokiniana) FUM* clusters (Fig. 2B). These findings suggest that loss or degeneration of the *FUM* cluster is not uncommon and that there may be limited selection for its retention. It should be noted that the Single Ancestral Origin scenario does not preclude acquisition of additional genus or lineage-specific *FUM* cluster genes, such as *SDR1* in *Aspergillus*, by some *FUM* cluster homologs as they diverged from the ancestor or other evolutionary mechanisms discussed below from acting on the ancestral cluster during its subsequent evolution.

### *FUM* cluster evolution and dispersal

If a single ancestral origin of the *FUM* cluster in the ancestor of Pezizomycotina is assumed, it raises the question of what evolutionary mechanisms were responsible for dispersal of the cluster to relatively few species and genera in the Dothideomycetes, Eurotiomycetes, and Sordariomycetes. Both this and previous studies [21, 31] support that vertical transmission has played a role in the evolution of the *FUM* cluster. However, vertical transmission alone (Fig. 8B, left) of a single ancestral cluster cannot fully explain the closer than expected relationship observed between *Tolypocladium FUM* genes and those of *Alternaria* and *Bipolaris* in a majority of *FUM* gene trees, which significantly conflict with the species phylogeny (Fig. 4, STable 4). We evaluate the support for two possible evolutionary mechanisms, Duplication and Differential Loss and Horizontal Transfer to explain this conflict.

In the Duplication and Loss scenario, an ancestral *FUM* cluster underwent an ancient duplication after it diverged from the *Aspergillus FUM* cluster, and the resulting paralogs were subsequently differentially retained or lost as they were dispersed by vertical inheritance to the Sordariomycetes and Dothideomycetes (Fig. 8B, middle). Duplication and differential loss of cluster paralogs in different lineages could explain the close relationship between the *Tolypocladium* and *Alternaria/Bipolaris FUM* clusters, as well as the presence of *FUM* gene paralogs in some species [80]. However, in our study, duplicate paralogs of *FUM* genes were only observed in *A. arborescens* (*FUM3* and *FUM14*) and *Bipolaris (FUM21)* species. These paralogs were nearly identical in sequence and grouped within the Dothideomycete clade in gene trees (Fig. 4, SFigure 6), supporting recent duplications of these genes within Dothideomycetes rather than an ancestral duplication (Fig. 4, SFigs. 6 and 7). Previous studies in the genus *Fusarium* [21] also found no direct evidence for duplication of the *FUM* cluster and did not observe two or more paralogs of *FUM* genes in a single species.

The Horizontal Transfer scenario also assumes a single origin of the *FUM* cluster in a Pezizomycotina ancestor that was subsequently vertically transmitted to the Eurotiomycetes and Sordariomycetes, but either not transmitted to or lost from the Dothideomycetes (Fig. 8B, right). This scenario invokes horizontal transfer of the *FUM* cluster from an ancestor of *Tolypocladium* in the Sordariomycetes to an ancestor of *Alternaria/Bipolaris* in the Dothideomycetes to explain the closer than expected relationship between *Tolypocladium* and *Alternaria*/*Bipolaris FUM* genes relative to species relationships (Fig. 4, SFigure 6). This scenario is also consistent with the results of NOTUNG reconciliation and constraint analyses (Fig. 5, SFigure 7, STable 4). That horizontal transfer has likely played a role in dispersing other SMBCs across classes of the Pezizomycotina is supported by multiple examples of horizontal transfer of SMBCs from one fungal class to another [82–85]. A previous study in *Fusarium* based on similar evidence also led to the conclusion that the *FUM* cluster was horizontally transferred from *Fusarium bulbicola*, or a recent ancestor, to *F. oxysporum* [21].

The genomic location of the AAL cluster is also consistent with HGT. Other authors [Nishimura et al. (1983)] previously proposed that the *Alternaria FUM* cluster (e.g. AAL toxin cluster) was horizontally transferred to *A. arborescens* from another fungus [86]. Based on patterns of GC content and codon bias, the first genome of *A. arborescens* (strain EGS 39–128) showed that the *FUM1* (*ALT1*) gene likely resided on a 1 Mb conditional dispensable chromosome (CDC) [64]. BLAST similarity searches showed that many genes on this CDC were more closely related to genes from other fungi than to the closely related species *Alternaria brassicicola.* While most genes were more closely related to genes from other Dothideomycetes (e.g., *Pyrenophora*,* Leptosphaeria*,* Phaeospharia*), some were more closely related to those in Sordariomycetes (e.g. *Fusarium vanettenii* (formerly *Nectria haematococca*). Genome sequencing of the *A. arborescens* NRRL 66744 strain further supports location of the complete AAL/*FUM* cluster on a CDC as both the NRRL 66744 scaffold 11 containing the *FUM* cluster and the previously sequenced As-27 *FUM* cluster sequence aligned to the EGS 39–128 contigs identified as being part of the CDC (Fig. 3A). The length of the NRRL 66744 scaffold 11 (0.46 Mb) is also consistent with the size of CDCs in ascomycetes. As CDCs do not follow mendelian patterns of inheritance and have been shown to transfer between fungal species [87], evidence that the *A. arborescens FUM* cluster is likely located on a CDC, as well as the presence of multiple transposable elements flanking the *A. arborescens FUM* cluster (Fig. 3), suggest possible mechanism(s) for horizontal transfer of the cluster. Additionally, previous studies have noted accelerated evolution and degeneration of SMBCs after horizontal transfer [88, 89], similar to the higher levels of rearrangement and pseudogenes in *FUM* clusters we observed among the Dothideomycete genera (*Alternaria* and *Bipolaris* spp.) compared to *FUM* clusters in other genera.

It is noteworthy that *FUM1* was the only *FUM* gene that NOTUNG and constraint analyses did not provide support for a putative horizontal transfer. The reason for this is not clear. However, the node grouping *Tolypocladium* with other Sordariomycetes (*Fusarium*), received only 62% bootstrap support, while *FUM* gene trees supporting the alternative topology grouping *Tolypocladium* with *Alternaria/Bipolaris *together, all had greater than 70% bootstrap support, with all but two having 100% bootstrap support (SFigure 6). Given that *FUM1* is essential for formation of fumonisins and AAL toxin, it seems likely that a putative horizontal transfer would have also included *FUM1*. If *FUM1* was horizontally transferred along with the rest the cluster, a possible explanation of the difference in topology of the *FUM1* tree versus other *FUM* gene trees is that the *A. arborescens* and *Bipolaris* Fum1 homologs underwent selection after the putative horizontal transfer. The driver of such selection is unknown, but one possibility is suggested by the differences in structure of the polyketide products of Fum1. In *A. arborescens* and presumably *Bipolaris*, Fum1 synthesizes a 16-carbon-long polyketide, whereas in *Aspergillus*, *Fusarium* and *Tolypocladium*, Fum1 synthesizes an 18-carbon-long polyketide. Thus, we speculate that selection driven by a change in polyketide structure provides a possible explanation for the difference in topology of the *FUM1* tree versus other *FUM* gene trees.

### Most likely evolutionary scenario for the *FUM* cluster

We present what we consider the most likely evolutionary scenario in Fig. 8C. Overall, our results provide strong support for a single and shared evolutionary origin of the *FUM* and AAL toxin producing clusters containing at least the 11 *FUM* genes conserved in *FUM* clusters among extant lineages. Sequence based phylogenetic analysis alone has limitations and the rapid rate of evolution, frequent duplication, loss, and recombination of fungal SMBCs [90], including the FUM cluster [21, 31], poses additional challenges to reconstructing a definitive evolutionary history of the *FUM* cluster. Available evidence supports that subsequent evolution of this ancestral *FUM* cluster likely involved multiple evolutionary processes including (1) duplication of at least some *FUM* genes, (2) acquisition of additional cluster genes in some lineages, (3) vertical transmission within genera and classes, and (4) either duplication and differential loss or horizontal transfer. Considering all evidence, including the lack of paralogs of ancient origin of *FUM* gene, the genomic location of the *Alternaria FUM* cluster on a CDC, and multiple transposable elements flanking this cluster, we favor horizontal transfer from the Sordariomycetes to the Dothideomycetes as the most likely scenario to explain the unusually close phylogenetic relationships between a majority of *FUM* genes in *Tolypocladium* and *Alternaria/Bipolaris*, but acknowledge that duplication and differential loss can also explain this pattern.

## Conclusions

The results of the current study demonstrate for the first time that the clusters that confer production of fumonisins and AAL toxins share a single ancestral origin. Our results suggest that multiple evolutionary processes shaped the subsequent evolution of this ancestral cluster to explain its current distribution limited to a few genera in three classes within the Pezizomycotina and the closer than expected relationship between *Tolypocladium* and *Alternaria/Bipolaris FUM* genes that conflicts with species phylogeny. Further, we provide evidence that changes in gene content and function, including mutation of a single amino acid in *Fum8*, is responsible for observed variation in structures of fumonisin analogs. The scarcity of the *FUM* cluster in fungi and evidence that it has been lost numerous times suggests limited or highly specific selection for AAL toxins and fumonisins. Despite this, out results indicate that AAL toxin or fumonisin production occurs in fungi with diverse lifestyles (e.g., insect pathogens, plant pathogens, endophytes, and saprophytes). This suggests these SAM metabolites can provide a selective advantage in multiple environments. Understanding the varied roles of AAL toxin/fumonisin production in the ecology of these diverse fungi should provide further insights into the evolution of AAL toxins and fumonisins. This in turn has potential to aid the discovery of beneficial uses for the metabolites and/or methods to reduce their occurrence in food and feed crops.

## Supplementary Information

Supplementary Material 1.

Supplementary Material 2.

Supplementary Material 3.

## Data Availability

The genomes and sequencing datasets generated for this study are available in the NCBI genomes database. Accession numbers are listed in STable 1.

## Abbreviations

AAL toxin: Alternaria alternata host–specific toxin
CDC: Conditional dispensable chromosome
FUM: Fumonisin
HGT: Horizontal gene transfer
LC: MS–Liquid chromatography mass spectrometry
SMBC: Secondary Metabolite Biosynthetic Cluster
