# Supplementary material for: Distribution and shared evolutionary history of the Fumonisin and AAL toxin biosynthetic gene clusters
Source: BMC Genomics. 2026 Jan 21;27:71. doi: 10.1186/s12864-025-12037-3 (PMC12821873; doi:10.1186/s12864-025-12037-3)
Supplement: Supplementary file 1 — Supplementary Material 1. [file 12864_2025_12037_MOESM1_ESM.pdf]

Supplemental Files (Methods)

Supplemental File 1. Methods for Split-Marker PCR knockout of FUM8 homolog (TINF2088) in *T. inflatum*.

A. Amplification of left-flank (1422 bp) and right flank (1615) of gene TINF2088.

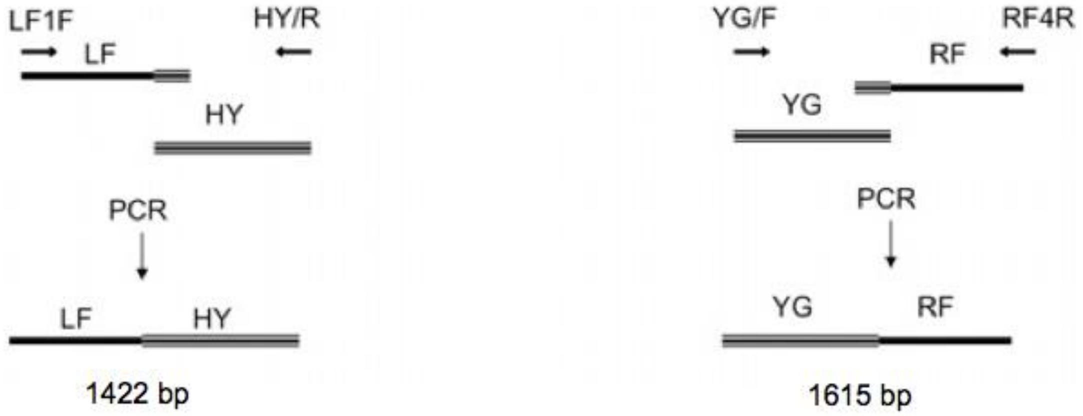

B. Transformation of two flanks and integration by homologous recombination.

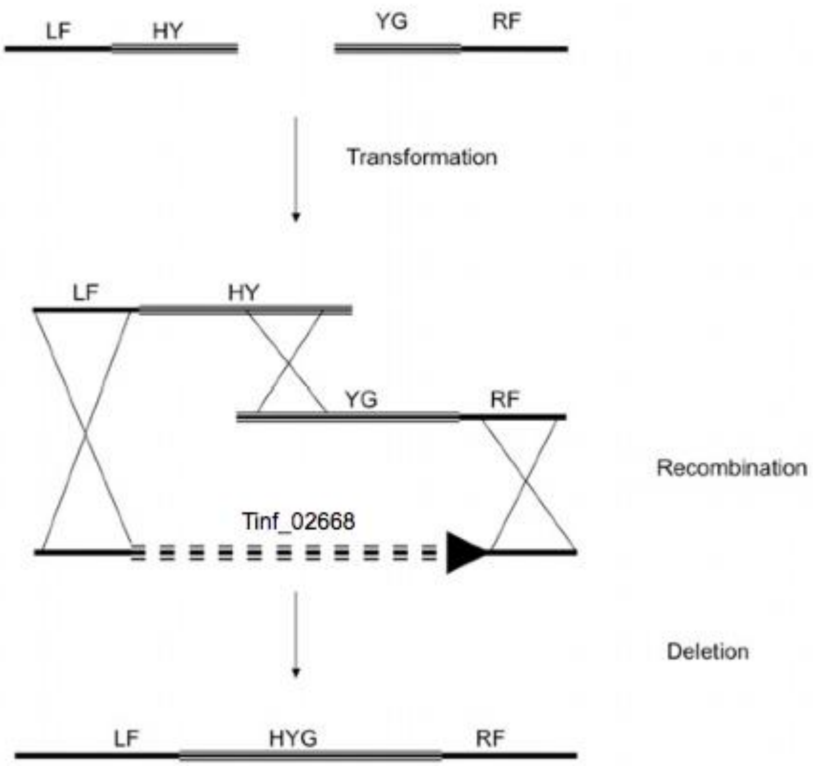

C. PCR screening for correct integration

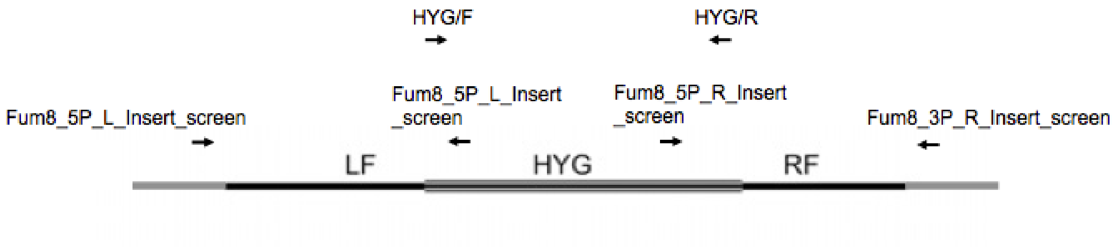

D. Primers used in transformation and screening

Primer Name	Sequence
FUM_8_LF1F	ACTTGTCCTCCCGTCCTTTT
FUM_8_LF2R	TTGACCTCCACTAGCTCCAGCGGCGCGTAGGGCGGTCTTGAAAATGA
FUM_8_RF3F	ATAGAGTAGATGCCGACCGGGCCGGCTTTTTGGCTCCATTTTCACC
FUM_8_RF4R	CTGGGTCTTGGCTGATGTTG
FUM_8_LF1F	ACTTGTCCTCCCGTCCTTTT
HY/R	GTATTGACCGATTCCTTGCGGTCCGAA
FUM_8_RF4R	CTGGGTCTTGGCTGATGTTG
YG/F	GATGTAGGAGGGCGTGGATATGTCCT
HYG/F	CGCGCCGCTGGAGCTAGTGGAGGTCAA
HYG/R	GCCGGCCCGGTCGGCATCTACTCTAT
Fum8_5P_L_Insert_screen	AAGCCCGTCAATACAGATGC
Fum8_3P_L_Insert_screen	GCACCAAGCAGCAGATGATA
Fum8_5P_R_Insert_screen	ATTTCGGCTCCAACAATGTC
Fum8_3P_R_Insert_screen	AGCCGTCTCTGTTGGTCTGT

E) Screening of wildtype and FUM8 transformants for correct insertion of HYG resistance construct into FUM8 gene. From left to right: lanes 1-6: is screening of left flank (primers Fum8_5P_L_Insert_screen and Fum8_3P_L_Insert_screen; expected size 1498bp): 1 Kb ladder, wild-type, knockout #2, knockout #3, ectopic #1, no DNA control, lanes 7-12 is screening of right flank (primers Fum8_5P_R_Insert_screen and Fum8_3P_R_Insert_screen; expected size 1718 bp): 1 Kb ladder, wild-type, knockout #2, knockout #3, ectopic #1, no DNA control. Transformants 2 and 3 show correct insertion on both flanks.

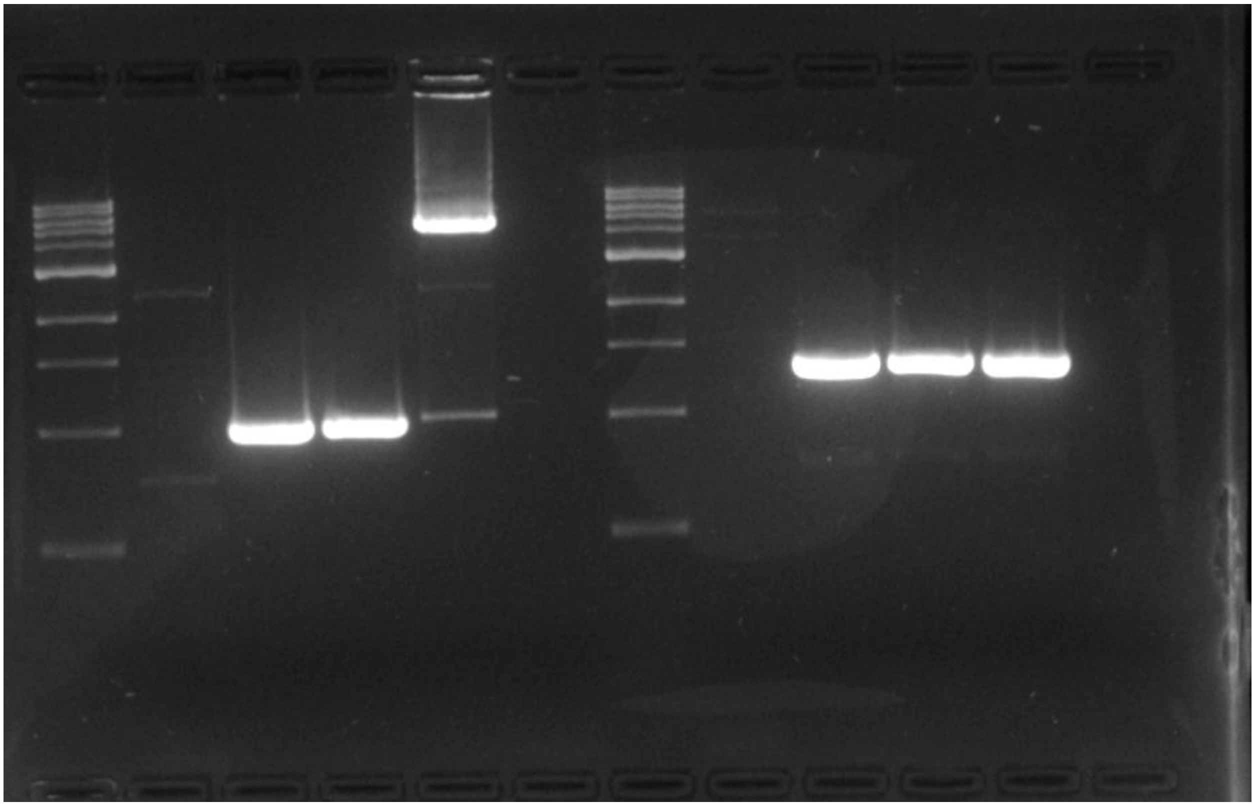

Supplementary File 2. Deletion of the *FUM15* gene in *Fusarium oxysporum* NRRL 39464

Strains and growth conditions

The hydroxy-FC_1-producing strain of *Fusarium oxysporum* used in this study was strain NRRL 39464 (FRC O-1890 = CAR1) (Seo et al. 1996; Seo and Lee 1999). Strains were

routinely maintained on V8 juice agar medium (Tuite 1969) and grown at 28°C. For fumonisin analysis, the fungus was grown on cracked maize kernel medium as previously described (Proctor et al. 2006). Glycerol stocks of fungal strains were maintained at -80°C.

Generation of *FUM15* deletion mutant

Deletion of the *FUM15* ortholog in *F. oxysporum* was done using a previously described protoplast-mediated transformation method (Kim et al. 2020). Briefly, two 1.6-kb fragments corresponding to the *FUM15* 5' and 3' flanking regions were amplified from genomic DNA of *F. oxysporum* NRRL 39464 by PCR with primer pairs 2857/2858 and 2861/2862, respectively. The 1.5 kb hygromycin resistance gene (*hygB*) was then amplified from plasmid pJML31.1 using the primers 2859 and 2860 (Lohmar et al. 2022). Fusion PCR of the three PCR products was carried out with the primer pair 2863/2864 to generate a single 4.0 kb fusion PCR product, which was transformed into *F. oxysporum* NRRL 39464 to delete the *FUM15* coding region. Primers used in this study are listed in SFile 2 **Table S1**.

Protoplast-mediated transformation was carried out as previously described (Lohmar et al. 2022). Initially, transformants were screened for deletion of *FUM15* based on resistance to hygromycin B and subsequently by a diagnostic PCR analysis (SFile 2 **Figure S1A)**. The latter analysis identified one transformant, strain tJML44.9, in which the *FUM15* coding region was deleted and replaced by the *hygB* gene (SFile 2 **Figure S1B)**. This transformant was then subjected to whole genome sequence analysis to verify *FUM15* deletion (see below).

Whole genome sequencing

Whole genome sequencing was carried out using previously described methods (Lohmar et al. 2022) but with minor modifications. Briefly, genomic DNA was isolated with the Quick-DNA Fungal/Bacterial Miniprep Kit (Zymo Research, D6005). The Illumina Nextera XT DNA Library Preparation Kit was used to prepare DNA libraries for sequencing from 1 ng of genomic DNA. Sequencing of the resulting library was performed with MiSeq Reagent Kit Version 3 and samples were run on a MiSeq Illumina sequencer. Analysis of the whole genome sequencing reads was carried out using Geneious Prime (version 2023.0.1).

Raw reads were trimmed, and low-quality reads were removed using the BBDuk Adapter/Quality Trimming (version 38.84, Brian Bushnell). BBDuk Adapter/Quality Trimming was carried out with all standard settings except the trim low-quality reads and discard short read settings were changed to a minimum quality/length of 30. Mapping sequencing reads to select reference sequences was carried out using Bowtie2and an end-to-end alignment type (Version 2.5.1, Langmead and Salzberg, 2012).

To verify that the *FUM15* coding region was deleted in the putative *FUM15* deletion mutant (strain tJML44.9), we used the program Bowtie2 and an end-to-end alignment type (Version 2.5.1) (Langmead and Salzberg 2012) to map reads from the genome sequence of strain tJML44.9 to the following two sequences: 1) a 14.8-kb region of the *FUM* cluster of *F. oxysporum* NRRL 39464 that included the wild-type *FUM15* gene; and 2) an equivalent 14.5-kb region of the *FUM* cluster of *F. oxysporum* in which the *FUM15* coding region was removed and replaced by the *hygB* gene (SFile 2 **Figure S1**). The results of this analysis confirmed deletion of *FUM15* in strain tJML44.9. That is, none of the sequence reads from strain tJML44.9 mapped to the segment of the first reference sequence corresponding to the *FUM15* coding region, whereas hundreds of reads mapped to the *hygB* segment of the second reference sequence (**SFile 2 Figure S2**).

Fumonisin analysis

Fumonisin production of *F. oxysporum* strains was determined first growing strains on V8 juice agar medium for 7 days. A ~0.5 cm plug taken from the resulting culture was used to inoculate 4 g of cracked maize kernel medium in 4-dram vial. The cultures were allowed to incubate at room temperature in the dark for 10 days, and three replicate cultures of each strain were analyzed. Fumonisins were extracted using previously described methods (Brown et al. 2007) with minor modifications. Briefly, fumonisins were extracted by adding acetonitrile:water (1:1, v/v) into the 4 dram vials containing cracked maize kernel medium cultures. The solvent-culture was allowed to incubate for 3 hours at room temperature with mild shaking. After this incubation, 1 mL of extract was collected from each sample and analyzed via liquid chromatography coupled with mass

spectrometry (LC-MS) using the methods previously described in (Brown et al. 2007). The results of this analysis indicated that the wild-type progenitor strain (NRRL 39464) and the *FUM15* deletion mutant (Δ*FoFUM15*) produced similar levels of hydroxy-FC_1 as well as other fumonisin analogs (**SFile 2 Table S2**). These results indicate that the *FUM15*-encoded monooxygenase does not catalyze formation of the 3-hydroxyl group of hydroxy-FC_1.

References

Brown DW, Butchko RAE, Busman M, Proctor RH. 2007. The *Fusarium verticillioides FUM* gene cluster encodes a Zn(II)2Cys6 protein that affects *FUM* gene expression and fumonisin production. Eukaryot Cell 6:1210-1218.

Kim HS, Lohmar JM, Busman M, Brown DW, Naumann TA, Divon HH, Lysoe E, Uhlig S, Proctor RH. 2020. Identification and distribution of gene clusters required for synthesis of sphingolipid metabolism inhibitors in diverse species of the filamentous fungus *Fusarium*. BMC Genomics 21:510. 10.1186/s12864-020-06896-1

Langmead B, Salzberg SL. 2012. Fast gapped-read alignment with Bowtie 2. Nat Methods 9:357-359. 10.1038/nmeth.1923

Lohmar JM, Rhoades NA, Patel TN, Proctor RH, Hammond TM, Brown DW. 2022. A-to-I mRNA editing controls spore death induced by a fungal meiotic drive gene in homologous and heterologous expression systems. Genetics 221. 10.1093/genetics/iyac029

Proctor RH, Plattner RD, Desjardins AE, Busman M, Butchko RAE. 2006. Fumonisin production in the maize pathogen *Fusarium verticillioides*: genetic basis of naturally occurring chemical variation. J Agric Food Chem 54:2424-2430.

Seo J-A, Kim J-C, Lee Y-W. 1996. Isolation and characterization of two new type C fumonisins produced by *Fusarium oxysporum*. J Nat Prod 59:1003-1005.

Seo J-A, Lee Y-W. 1999. Natural occurrence of the C series of fumonisins in moldy corn. Appl Environ Microbiol 65:1331-1334.

Tuite J. 1969. Plant Pathological Methods: Fungi and Bacteria. Minneapolis, MN: Burgess Publishing Company. p. 1-239.

SFile 2 Table S1. Primer sequences

Primer name	Sequence (5’-3’)
739	CGCCAGGGTTTTCCCAGTCACGAC
740	AGCGGATAACAATTTCACACAGGA
2757	CCGATAGTGGAAACCGACGCC
2758	CCAGCCAAGCCCAAAAAATGCTCC
2857	CACCATACCAAGGACGCTCAGGAT
2858	ACTGCCCGTTCTCGGATTGTCAAA
2859	TTTGACAATCCGAGAACGGGCAGTGAATTCCTGCAGCCCAACTGATATTG
2860	GAGATATCTTGAATTTACCACTATCAGTGTGGCCGCTCTAGAACTAGTGGATCCA
2861	ACACTGATAGTGGTAAATTCAAGATATCTC
2862	CGTCAAGCCGTCTGATCTCTAT
2863	CTTCCAGGTGTTTGACTAGCCGAGC
2864	CCGAAGACCCTTACTGATGCGTCTC
2865	ATTTGGCTGAACCGATCCCCG
2866	GCGTTGTCGGCTATCTGGTCTTCT
2867	GTGAGTGGAATAGGAGCGAAGTACCG

SFile 2, Table S2: Fumonisin content (ng/μL) in extracts of cracked maize kernel cultures of wild-type *Fusarium oxysporum* strain NRRL 39464 (Wild Type) and *FUM15* deletion mutant strain tJML44.9 (Δ*FoFUM15*).

		**FB_1**	**FB_2**	**FB_3**	**FB_4**	**FC_1-OH**	**FC_1**	**FC_2**	**FC_3**	**FC_4**
**Wild Type**	Average	0.55	0.11	0.12	0.03	84.61	84.06	11.86	7.49	11.17
	Standard error	0.14	0.03	0.03	0.03	20.60	11.31	2.55	2.14	1.89
**Δ*FoFUM15***	Average	1.20	6.17	0.26	0.40	119.93	73.77	17.18	11.48	18.35
	Standard error	0.21	4.18	0.06	0.22	19.81	14.45	2.05	3.97	3.85

A

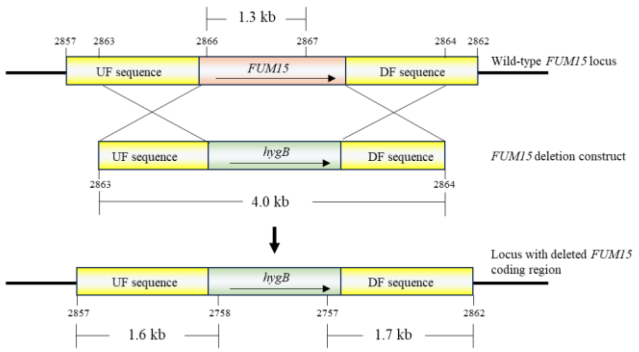

B

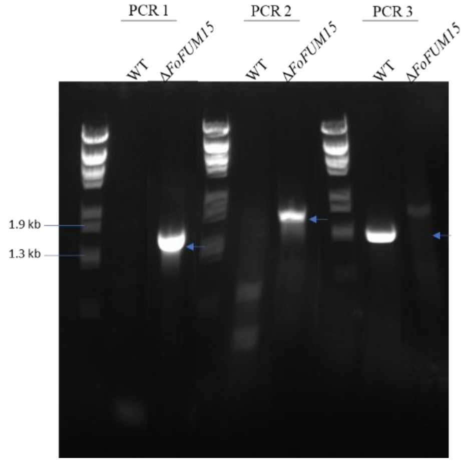

SFile 2, Figure S1: Construction the *FUM15* deletion construct and confirmation of *FUM15* deletion in a *Fusarium oxysporum* transformant strain tJML44.9. (A) Schematic representation of the *F. oxysporum FUM15* deletion construct. Abbreviations: UF – *FUM15* upstream flanking sequence; DF – *FUM15* downstream flanking sequence; Δ*FoFUM15* – *F. oxysporum FUM15* deletion mutant strain tJML44.9; *hygB* – hygromycin B resistance gene. (B) Diagnostic PCR analysis of Δ*FoFUM15* confirming successful replacement of the *FUM15* coding region with *hygB*. The image in this panel was modified to remove lanes that contained amplification products from transformants that were not *FUM15* deletion mutants. PCR 1 and PCR 2 were conducted using primer pairs 2857/2758 and 2862/2757 to yield 1.6 kb and 1.7 kb bands, respectively, with genomic data from Δ*FoFUM15*. The same PCR assays were expected to yield no band with DNA from the wild-type progenitor strain of *F. oxysporum* because it did not have *hygB*. Bands amplified from DNA of Δ*FoFUM15* indicate the successful replacement of the *FUM15* coding sequence with *hygB*. PCR 3 was done using primer pair 2866/2867, which bind within the wild-type *FUM15* coding region. Amplification of the 1.3-kb band from wild-type *F. oxysporum* DNA and no band from Δ*FoFUM15* DNA further support that the *FUM15* coding sequence is absent in Δ*FoFUM15*.

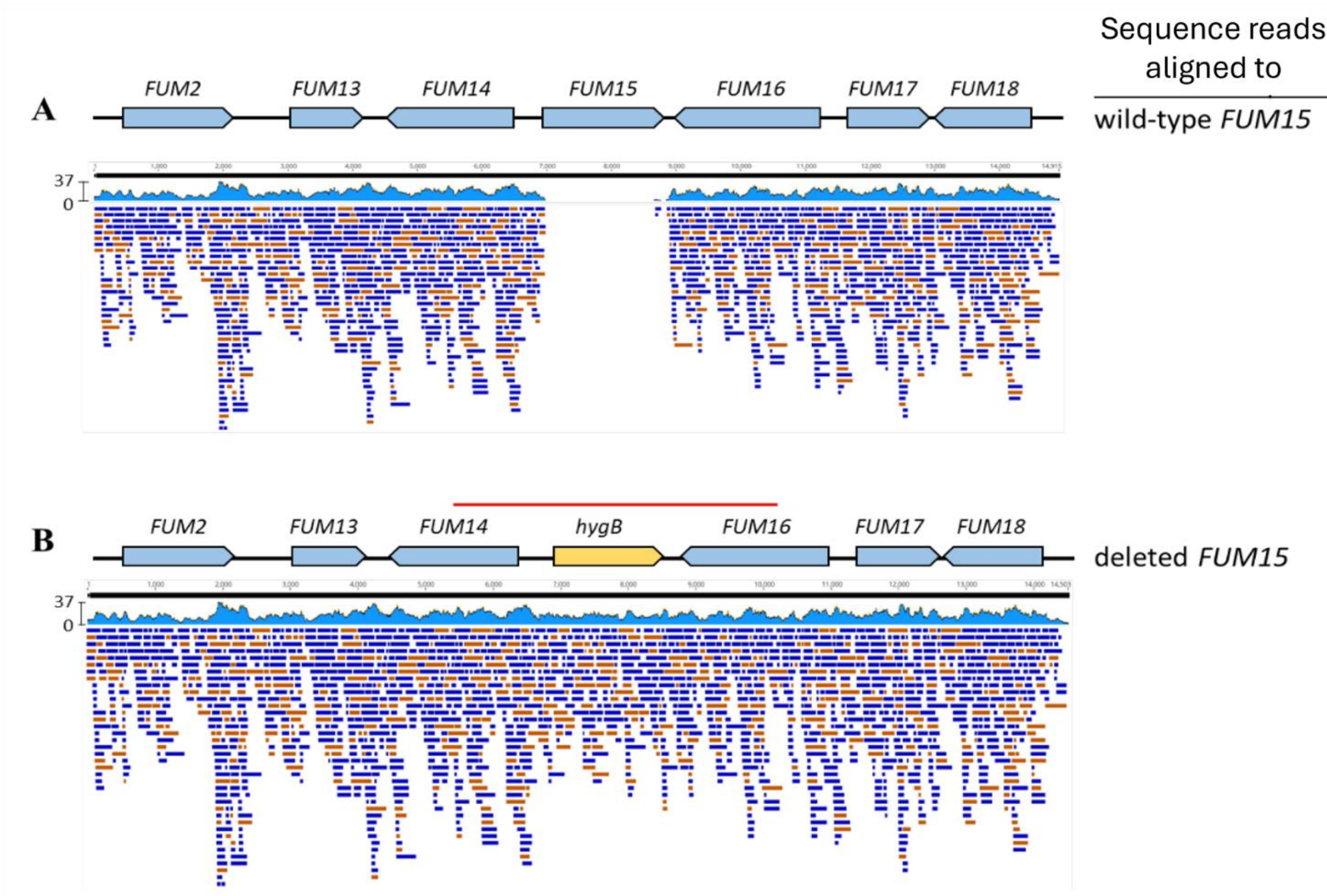

SFile 2, Figure S2: Confirmation of *FUM15* deletion in *F. oxysporum* strain tJML44.9 (Δ*FoFUM15*) using the 'Map Reads to Reference' function in CLC Genomics Workbench. In A and B, whole-genome sequence reads, generated by Illumina MiSeq, were mapped to different sequences. In **A**, reads were aligned to a 14.8 kb segment of the wild-type *F. oxysporum FUM* cluster that spans the *FUM2* and *FUM13* – *FUM18* genes, including the wildtype *FUM15* gene. In **B**, the reads were aligned to this same region of the *FUM* clusterin the mutant strain, in which the *FUM15* coding region has been replaced with the hygromycin B resistance gene (*hygB*). The red line above the sequence in B corresponds to the *FUM15* deletion construct. In A, the lack of sequence reads mapped to the *FUM15* coding region indicates that the coding region is absent in the genome of Δ*FoFUM15*. In B, mapping of sequence reads to *hygB* as well as reads that span *hygB* and flanking *FUM* cluster sequences is consistent with replacement of the *FUM15* coding region with *hygB* in the Δ*FoFUM15* genome. The orange and darker blue bars in A and B indicate the sequence reads that mapped to one or both reference sequences. The colors indicate directionality

of reads: blue indicates forward, and orange indicates reverse. The light blue chromatogram-like image indicates read coverage along the reference sequences. The scale shown to the left of the images indicates read coverage ranged from 0 to 37.

Supplementary File 3: Site-directed mutagenesis to generate the A-to-V (A580V) and the V-to-A (V580A) mutations at position 580 of the Fum8 homologs from *F. verticillioides* strain NRRL 20956 and *F. oxysporum* strain NRRL 39464, respectively. The mutations were made in the *FUM8* homologs that had been previously cloned into plasmids pFvF8-comp (*F. verticillioides FUM8*) and pFoFUM8-Gen (*F. oxysporum FUM8*) [1].

Gene	DNA and amino acid (AA) sequences	
F. verticillioides FUM8		
Wild-type	GGACTTGGACCCAGCTCT**GCC**AGGTGGTTCTATGGG	DNA
	**G L G P S S A R W F Y G**	AA
A580V mutation	GGACTTGGACCCAGCTCT**GTC**AGGTGGTTCTATGGG	DNA
	**G L G P S S V R W F Y G**	AA
F. oxysporum FUM8		
Wild-type	GGGCTCGGACCCAGTTCT**GTC**AGGTGGTTCTATGGA	DNA
	**G L G P S S V R W F Y G**	AA
V580A mutation	GGGCTCGGACCCAGTTCT**GCC**AGGTGGTTCTATGGA	DNA
	**G L G P S S A R W F Y G**	AA

Primers used in In-Fusion HD Cloning Plus protocol (Takara Bio USA, Inc.) showing the position of the codon that was mutated.

First Extension Reaction Primer: Primer 3594 5’-GACCCAGCTCTGTCAGGTGGTTCTATGG-3’

Second Extension Reaction Primer: Primer 3594 5’-CCATAGAACCACCTGACAGAGCTGGGTC-3’

Primers used to amplify and sequence *F. verticillioides* and *F. oxysporum FUM8* fragments to assess the presence of the *FvFUM8* A580V and *FoFUM8* V580A mutations.

Primer 679 5’-CGTAGTAGGAATGAGAAGGATG-3’

Primer 680 5’-GCAAGCTTTGTGGCTGATTGTC-3’

Detection of complementation plasmids in transformants of *fum8* mutant

The *F. verticillioides fum8* mutant (strain GfA3245) used as a host strain for complementation experiments was generated in a previous study [2]. The mutant was generated by additive gene disruption, which resulted in two truncated copies of *FUM8*. One copy was truncated at the 5’ end, and the other was truncated at the 3’ end [2]. The region of *FUM8* that included the codon corresponding to Fum8 position 580 was present in the *fum8* mutant. Therefore, complementation of the *fum8* mutant with a plasmid carrying wild-type *F. verticillioides FUM8* (*FvFUM8*), wild-type *F. oxysporum FUM8* (*FoFUM8*), *FvFUM8* A580V mutation, or *FoFUM8* V580A mutation resulted in the presence of two copies of the region of *FUM8* with the codon corresponding to Fum8 position 580. It was possible to detect the presence of the complementation plasmids in transformants of the *fum8* mutant in two ways. First, a ~2950-bp amplicon consisting of a full-length copy of *FvFUM8* or *FoFUM8* could be amplified from transformants of the mutant carrying any of the complementation plasmids but not from the mutant itself. Second, examination of chromatograms from Sanger sequence analysis of transformants revealed the presence of two peaks for some nucleotides in the *FUM8* coding region. In the case of transformants carrying a plasmid with the wild-type *FoFUM8*, there were two peaks for nucleotide positions that differ between *FoFUM8* and *FvFUM8*, including the nucleotide position that was mutated to generate the V580A mutation (indicated with green highlight in sequence below). In transformants carry the plasmid with the *FoFUM8* V580A mutation, there were two peaks for all positions that differ between *FoFUM8* and *FvFUM8*, except for the position that was changed to generate the V580A mutation.

fum8 mutant	GGACTTGGACCCAGCTCTGCCAGGTGGTTCTATGGG
Wild-type *FoFUM8*	GGGCTCGGACCCAGTTCTGTCAGGTGGTTCTATGGA
FoFUM8 V580A	GGGCTCGGACCCAGTTCTGCCAGGTGGTTCTATGGA

By contrast, in transformants of the *fum8* mutant complemented with the plasmid carrying the wild-type *FvFUM8*, there were single peaks for all nucleotide positions, because the region of *FUM8* amplified form the *fum8* mutant and the complementation plasmid were identical. In transformants carrying the plasmid with the *FvFUM8* A580V mutation, there were single peaks for all nucleotide positions of *FUM8* except for the nucleotide that was changed to generate the A580V mutation; there was a double (C/T) peak at this position (indicated with green highlight in sequence below).

fum8 mutant	GGACTTGGACCCAGCTCTGCCAGGTGGTTCTATGGG
Wild-type *FvFUM8*	GGACTTGGACCCAGCTCTGCCAGGTGGTTCTATGGG
FvFUM8 A580V	GGACTTGGACCCAGCTCTGTCAGGTGGTTCTATGGG

1. Proctor, R.H., et al., *A fumonisin biosynthetic gene cluster in Fusarium oxysporum strain O-1890 and the genetic basis for B versus C fumonisin production.* Fungal Genet.Biol., 2008. **45**: p. 1016-1026.

2. Seo, J.A., R.H. Proctor, and R.D. Plattner, *Characterization of four clustered and coregulated genes associated with fumonisin biosynthesis in Fusarium verticillioides.* Fungal Genet Biol, 2001. **34**(3): p. 155-65.
