# Supplementary material for: Distribution and shared evolutionary history of the Fumonisin and AAL toxin biosynthetic gene clusters
Source: BMC Genomics. 2026 Jan 21;27:71. doi: 10.1186/s12864-025-12037-3 (PMC12821873; doi:10.1186/s12864-025-12037-3)
Supplement: Supplementary file 2 — Supplementary Material 2. [file 12864_2025_12037_MOESM2_ESM.pdf]

STable 1 – List and GenBank Accession number of fungal genomes in which FUM cluster homologs were detected. Strains marked with an asterix (*) were sequenced for this study.

Species	Strain	GenBank Genome Sequence/Assembly Accession
Alternaria arborescens	EGS 39-128	AIIC00000000.1
Alternaria arborescens	NRRL 66744*	JAZHYV000000000
Alternaria arborescens	NRRL 20593*	JAACJH000000000.1
Aspergillus lacticoffeatus	CBS 101883	GCA_003184595.1
Aspergillus phoenicis	ATCC 13157	QQUR00000000.1
Aspergillus sclerotiicarbonarius	CBS 121057	PSSZ00000000.1
Aspergillus niger	ATCC 1015	ACJE01000004.1
Aspergillus welwitschiae	ITEM 11945	WWFU00000000.1
Bipolaris maydis	ATCC 48331	AIHU00000000.1
Bipolaris sorokiniana	ND90Pr	AEIN01000000.1
Bipolaris zeicola	26-R-13	AMCN01000000.1
Bipolaris zeicola	GZL10	JAEUBC000000000.1
Tolypocladium cylindrosporum	IBT 41712*	JAABOL000000000.1
Tolypocladium inflatum	NRRL 8044	AOHE00000000.1
Tolypocladium paradoxum	NRBC 100945	PKSG00000000.1
Fusarium fujikuroi	IMI 58289	GCA_900079805.1
Fusarium oxysporum	NRRL 39464 = FRC O-1879	JAAFOW000000000.1
Fusarium verticillioides	FGSC 7600	GCA_000149555.1

STable 2. Production of fumonisins in *Tolypocladium* strains and AAL toxin in *Alternaria arborescens* strains.

Fumonisin production (ng/mL)[a]					AAL Toxin production (ng/mL)[b]		
Strain	FB_1	FB_2	FB_3	FB_4	Strain	AAL-TA	AAL-TB
T. cylindrosporum (wild-type strains)					*A. arborescens* (wild-type strains)		
Control	0.0	0.0	0.0	0.0	Control	0.0	0.0
IBT 41711	0.0	3.6	0.0	2.3	NRRL 20593	104.3	22.2
IBT 41712	0.0	2.3	0.0	1.0	NRRL 66744	23.6	3.6
T. inflatum (wild-type strains)							
IBT 41581	0.0	4.1	0.0	1.4			
IBT 41582	0.0	0.1	0.0	0.1			
IBT 41583	0.0	0.0	0.0	0.1			

[a] In fumonisin production assays, *Tolypocladium* strains were grown in cracked maize kernel medium; and in AAL toxin production assays, *A. arborescens* strains were grown in rice kernel medium. All values are averages derived from 2 or 3 replicate cultures. Detection and quantification of the metabolites was determined by tandem liquid chromatography-mass spectrometry as described in the Methods.

Stable3: Functional annotation of *FUM* cluster associated genes based on sequence homology to a gene family and known functions in fumonisin biosynthesis based on previous genetic studies.

Designations			Gene Family	Functional role in fumonisin or AAL toxin biosynthesis
***FUM* Gene**	**Protein**	***ALT* Gene**		
FUM1	Fum1	*ALT1*	Polyketide Synthase	Synthesis of linear polyketide precursor of fumonisins and AAL toxins
FUM2	Fum2	nd	Cytochrome P450 Monooxygenase	Hydroxylation of fumonisin backbone at carbon atom 10 (C10) of FBs and C9 of FCs
FUM3	Fum3	*ALT11*	Dioxygenase	Hydroxylation of fumonisin backbone at C5 (FBs) or C4 FCs
FUM6	Fum6	*ALT*	NADPH Cytochrome P450 Monooxygenase	Hydroxylation of fumonisin backbone at C14 and C15 (FBs) or C13 and C14 (FCs)
FUM7	Fum7	*ALT*	Dehydrogenase	Reduction of carbon-carbon double bond in tricarboxylate precursor
FUM8	Fum8	*ALT4*	Class II Aminotransferase (= α-Oxoamine Synthase)	Condensation of linear polyketide and alanine (FBs) or glycine (FCs)
FUM10	Fum10	*ALT10*	Acyl-CoA Synthase	Activation of tricarboxylate with CoA
FUM11	Fum11	nd	Mitochondrial tricarboxylate transporter	Transport of tricarboxylate from mitochondrial lumen to cytoplasm
FUM13	Fum13	*ALT6*	Dehydrogenase	Reduction of carbonyl/keto group adjacent to amine
FUM14	Fum14	*ALT12*	Nonribosomal Peptide Synthetase-like Condensation Domain Protein	*O*-acetylation of tricarboxylate to hydroxyl on fumonisin backbone at C14 and C15 (FBs) or C13 and C14 (FCs)
FUM15	Fum15	*ALT8*	Cytochrome P450 Monooxygenase	Unknown
FUM16	Fum16	nd	Acyl-CoA Synthase	Unknown
FUM17	Fum17	nd	Ceramide Synthase	Unknown
FUM18	Fum18	nd	Ceramide Synthase	Self-protection
FUM19	Fum19	*ALT5*	ABC Transporter	Transport of fumonisins out of hyphae
FUM21	Fum21	*ALT13*	Zn(II)2Cys6 Transcription Factor	Transcriptional regulation of *FUM* genes
nd	Alt7	*ALT7*	Ceramide Synthase	Unknown
nd	Alt9	*ALT9*	Mitochondrial Tricarboxylate Transporter	Transport of tricarboxylate from mitochondrial lumen to cytoplasm (presumed)

nd	Sdr1	*SDR1/sdr1*	Dehydrogenase	Unknown

STable4: Results of Shimodaira-Hasegawa (SH) and Approximately Unbiased (AU) Tests of constrained trees. The tests compared the unconstrained tree to itself (=None) and to a constrained tree(s).

			P values[b]	
**Gene**	**Constraint[a]**	**Log Likelihood**	**SH**	**AU**
FUM1	None	-31885.976	1.0000	0.8284
	Tolypocladium-Fusarium clade	-32137.098	0.0000	0.0000
	Tolypocladium-Alternaria-Bipolaris clade	-31893.665	0.4685	0.1716
FUM3	None	-3256.773	1.0000	1.0000
	Tolypocladium-Fusarium clade	-3317.341	0.0001	0.0000
FUM6	None	-12623.219	1.0000	1.0000
	Tolypocladium-Fusarium clade	-12688.201	0.0000	0.0000
FUM7	None	-5170.231	1.0000	0.8723
	Tolypocladium-Fusarium clade	-5173.206	0.1288	0.1277
FUM8	None	-9866.184	1.0000	0.9991
	Tolypocladium-Fusarium clade	-9903.654	0.0003	0.0009
FUM10	None	-8776.482	1.0000	0.9999
	Tolypocladium-Trichoderma-Fusarium clade	-8937.111	0.0000	0.0001
FUM13	None	-4823.907	1.0000	0.9943
	Tolypocladium-Fusarium clade	-4840.518	0.0135	0.0057
FUM14	None	-9533.271	1.0000	0.9999
	Tolypocladium-Trichoderma-Fusarium clade	-9568.589	0.0007	0.0001
FUM15	None	-7983.616	1.0000	0.9984
	Tolypocladium-Fusarium clade	-8003.824	0.0056	0.0016
FUM19	None	-18580.782	1.0000	0.9996
	Tolypocladium-Fusarium clade	-18645.573	0.0000	0.0004
FUM21	None	-12176.461	1.0000	0.9976
	Tolypocladium-Fusarium clade	-12194.351	0.0073	0.0024

[a] In constrained trees, the Newick text file of the gene tree was modified to force the clade indicated. For example, in one constrained *FUM1* tree, a *Tolypocladium-Fusarium* clade was forced, and in the other *FUM1* tree, a *Tolypocladium-Alternaria-Bipolaris* clade was forced.

[b] A p value of <0.05 indicates the constrained tree was less well supported than the original unconstrained see.

STable 5. Housekeeping genes and phylogenetic information content (PIC) and substitution model

Gene	Protein	Alignment Length	PIC	Percent PIC/gene	Percent total PICs	Best Substitution Model
ACL1	ATP-Citrate Lyase	492	136	27.6	1.8	LG+G4
ACT1	Actin	502	299	59.6	4.0	LG+G4
DPA1	DNA Polymerase Alpha Subunit	1572	816	51.9	10.8	JTTDCMut+I+G4
DPD1	DNA Polymerase Delta Subunit	1143	456	39.9	6.0	LG+I+G4
DPE1	DNA Polymerase Epsilon Subunit	2276	1071	47.1	14.2	LG+I+G4
FAS1	Fatty Acid Synthase Alpha Subunit	1892	688	36.4	9.1	LG+G4
FAS2	Fatty Acid Synthase Beta Subunit	2120	849	40.0	11.3	LG+I+G4
MCM7	DNA Replication Licensing Factor	849	412	48.5	5.5	LG+I+G4
PGK1	Phosphoglycerate Kinase	431	133	30.9	1.8	WAG+G4
RPB1	RNA Polymerase Largest Subunit	1858	899	48.4	11.9	LG+F+I+G4
RPB2	RNA Polymerase Largest Subunit	1303	543	41.7	7.2	LG+I+G4
TEF1	Translation Elongation Factor 1-Alpha	472	85	18.0	1.1	LG+I+G4
TOP1	DNA Topoisomerase I	988	532	53.8	7.1	LG+F+I+G4
TSR1	Ribosome Biogenesis Protein	864	502	58.1	6.7	LG+G4
TUB1	Tubulin Alpha Subunit	452	64	14.2	0.8	LG+I+G4
TUB2	Tubulin Beta Subunit	453	56	12.4	0.7	JTT+G4
			Total = 7541		Total = 100	
