# Supplementary material for: Distribution and shared evolutionary history of the Fumonisin and AAL toxin biosynthetic gene clusters
Source: BMC Genomics. 2026 Jan 21;27:71. doi: 10.1186/s12864-025-12037-3 (PMC12821873; doi:10.1186/s12864-025-12037-3)
Supplement: Supplementary file 3 — Supplementary Material 3. [file 12864_2025_12037_MOESM3_ESM.pdf]

SFigure1. Phylogenetic analysis of top 50 BLASTP hits to *F. verticillioides* FUM cluster genes and proteins as query against NCBI nr database. Proteins and translated coding sequences were aligned with MAFFT linsi using default parameters. Maximum likelihood phylogeny was generated using RAXML with protein substitution model determined by modeltest (JTT+G4 and JTT+G4+F). Bootstrap values from automatically determined bootstrap replicates are show on branches. Red highlighted clades indicate monophyletic clades containing *FUM* gene homologs.

FUM1

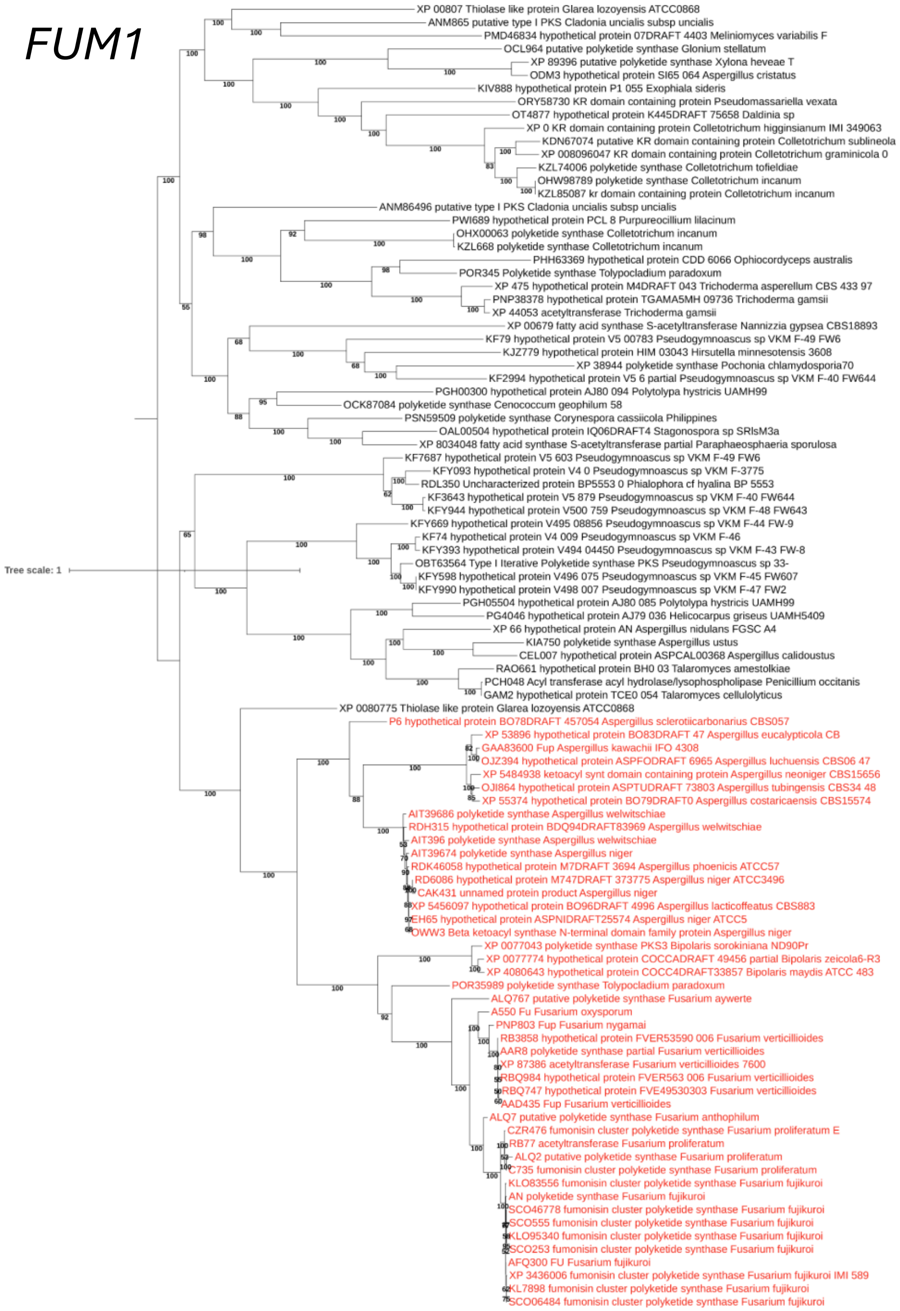

XP 00807 Thiolase like protein Glarea lozoyensis ATCC0868
ANM865 putative type I PKS Cladonia uncialis subsp uncialis
PMD46834 hypothetical protein 07DRAFT 4403 Meliniomyces variabilis F
OCL964 putative polyketide synthase Glonium stellatum
XP 89396 putative polyketide synthase Xylona heveae T
ODM3 hypothetical protein SI65 064 Aspergillus cristatus
KIV888 hypothetical protein P1 055 Exophiala sideris
ORY58730 KR domain containing protein Pseudomassariella vexata
OT4877 hypothetical protein K445DRAFT 75658 Daldinia sp
XP 0 KR domain containing protein Colletotrichum higginsianum IMI 349063
KDN67074 putative KR domain containing protein Colletotrichum sublineola
XP 008096047 KR domain containing protein Colletotrichum graminicola 0
KZL74006 polyketide synthase Colletotrichum tofieldiae
OHW98789 polyketide synthase Colletotrichum incanum
KZL85087 kr domain containing protein Colletotrichum incanum
ANM86496 putative type I PKS Cladonia uncialis subsp uncialis
PWI689 hypothetical protein PCL 8 Purpureocillium lilacinum
OHX00063 polyketide synthase Colletotrichum incanum
KZL668 polyketide synthase Colletotrichum incanum
PHH63369 hypothetical protein CDD 6066 Ophiocordyceps australis
POR345 Polyketide synthase Tolypocladium paradoxum
XP 475 hypothetical protein M4DRAFT 043 Trichoderma asperellum CBS 433 97
PNP38378 hypothetical protein TGAMA5MH 09736 Trichoderma gamsii
XP 44053 acetyltransferase Trichoderma gamsii
XP 00679 fatty acid synthase S-acetyltransferase Nannizzia gypsea CBS18893
KF79 hypothetical protein V5 00783 Pseudogymnoascus sp VKM F-49 FW6
KJZ779 hypothetical protein HIM 03043 Hirsutella minnesotensis 3608
XP 38944 polyketide synthase Pochonia chlamydosporia70
KF2994 hypothetical protein V5 6 partial Pseudogymnoascus sp VKM F-40 FW644
PGH00300 hypothetical protein AJ80 094 Polytolypa hystricis UAMH99
OCK87084 polyketide synthase Cenococcum geophilum 58
PSN59509 polyketide synthase Corynespora cassiicola Philippines
OAL00504 hypothetical protein IQ06DRAFT4 Stagonospora sp SRIsM3a
XP 8034048 fatty acid synthase S-acetyltransferase partial Paraphaeosphaeria sporulosa
KF7687 hypothetical protein V5 603 Pseudogymnoascus sp VKM F-49 FW6
KFY093 hypothetical protein V4 0 Pseudogymnoascus sp VKM F-3775
RDL350 Uncharacterized protein BP5553 0 Phialophora cf hyalina BP 5553
KF3643 hypothetical protein V5 879 Pseudogymnoascus sp VKM F-40 FW644
KFY944 hypothetical protein V500 759 Pseudogymnoascus sp VKM F-48 FW643
KFY669 hypothetical protein V495 08856 Pseudogymnoascus sp VKM F-44 FW-9
KF74 hypothetical protein V4 009 Pseudogymnoascus sp VKM F-46
KFY393 hypothetical protein V494 04450 Pseudogymnoascus sp VKM F-43 FW-8
OBT63564 Type I Iterative Polyketide synthase PKS Pseudogymnoascus sp 33-
KFY598 hypothetical protein V496 075 Pseudogymnoascus sp VKM F-45 FW607
KFY990 hypothetical protein V498 007 Pseudogymnoascus sp VKM F-47 FW2
PGH05504 hypothetical protein AJ80 085 Polytolypa hystricis UAMH99
PG4046 hypothetical protein AJ79 036 Helicocarpus griseus UAMH5409
XP 66 hypothetical protein AN Aspergillus nidulans FGSC A4
KIA750 polyketide synthase Aspergillus ustus
CEL007 hypothetical protein ASPCAL00368 Aspergillus calidoustus
RAO661 hypothetical protein BH0 03 Talaromyces amestolkiae
PCH048 Acyl transferase acyl hydrolase/lysophospholipase Penicillium occitanis
GAM2 hypothetical protein TCE0 054 Talaromyces cellulolyticus
XP 0080775 Thiolase like protein Glarea lozoyensis ATCC0868
P6 hypothetical protein BO78DRAFT 457054 Aspergillus sclerotiicarbonarius CBS057
XP 53896 hypothetical protein BO83DRAFT 47 Aspergillus eucalypticola CB
GAA83600 Fup Aspergillus kawachii IFO 4308
OJZ394 hypothetical protein ASPFODRAFT 6965 Aspergillus luchuensis CBS06 47
XP 5484938 ketoacyl synt domain containing protein Aspergillus neoniger CBS15656
OJI864 hypothetical protein ASPTUDRAFT 73803 Aspergillus tubingensis CBS34 48
XP 55374 hypothetical protein BO79DRAFT0 Aspergillus costaricaensis CBS15574
AIT39686 polyketide synthase Aspergillus welwitschiae
RDH315 hypothetical protein BDQ94DRAFT83969 Aspergillus welwitschiae
AIT396 polyketide synthase Aspergillus welwitschiae
AIT39674 polyketide synthase Aspergillus niger
RDK46058 hypothetical protein M7DRAFT 3694 Aspergillus phoenicis ATCC57
RD6086 hypothetical protein M747DRAFT 373775 Aspergillus niger ATCC3496
CAK431 unnamed protein product Aspergillus niger
XP 5456097 hypothetical protein BO96DRAFT 4996 Aspergillus lacticoffeatus CBS883
EH65 hypothetical protein ASPNIDRAFT25574 Aspergillus niger ATCC5
OWW3 Beta ketoacyl synthase N-terminal domain family protein Aspergillus niger
XP 0077043 polyketide synthase PKS3 Bipolaris sorokiniana ND90Pr
XP 0077774 hypothetical protein COCCADRAFT 49456 partial Bipolaris zeicola6-R3
XP 4080643 hypothetical protein COCC4DRAFT33857 Bipolaris maydis ATCC 483
POR35989 polyketide synthase Tolypocladium paradoxum
ALQ767 putative polyketide synthase Fusarium aywerte
A550 Fu Fusarium oxysporum
PNP803 Fup Fusarium nygamai
RB3858 hypothetical protein FVER53590 006 Fusarium verticillioides
AAR8 polyketide synthase partial Fusarium verticillioides
XP 87386 acetyltransferase Fusarium verticillioides 7600
RBQ984 hypothetical protein FVER563 006 Fusarium verticillioides
RBQ747 hypothetical protein FVE49530303 Fusarium verticillioides
AAD435 Fup Fusarium verticillioides
ALQ7 putative polyketide synthase Fusarium anthophilum
CZR476 fumonisin cluster polyketide synthase Fusarium proliferatum E
RB77 acetyltransferase Fusarium proliferatum
ALQ2 putative polyketide synthase Fusarium proliferatum
C735 fumonisin cluster polyketide synthase Fusarium proliferatum
KLO83556 fumonisin cluster polyketide synthase Fusarium fujikuroi
AN polyketide synthase Fusarium fujikuroi
SCO46778 fumonisin cluster polyketide synthase Fusarium fujikuroi
SCO555 fumonisin cluster polyketide synthase Fusarium fujikuroi
KLO95340 fumonisin cluster polyketide synthase Fusarium fujikuroi
SCO253 fumonisin cluster polyketide synthase Fusarium fujikuroi
AFQ300 FU Fusarium fujikuroi
XP 3436006 fumonisin cluster polyketide synthase Fusarium fujikuroi IMI 589
KL7898 fumonisin cluster polyketide synthase Fusarium fujikuroi
SCO06484 fumonisin cluster polyketide synthase Fusarium fujikuroi
Tree scale: 1

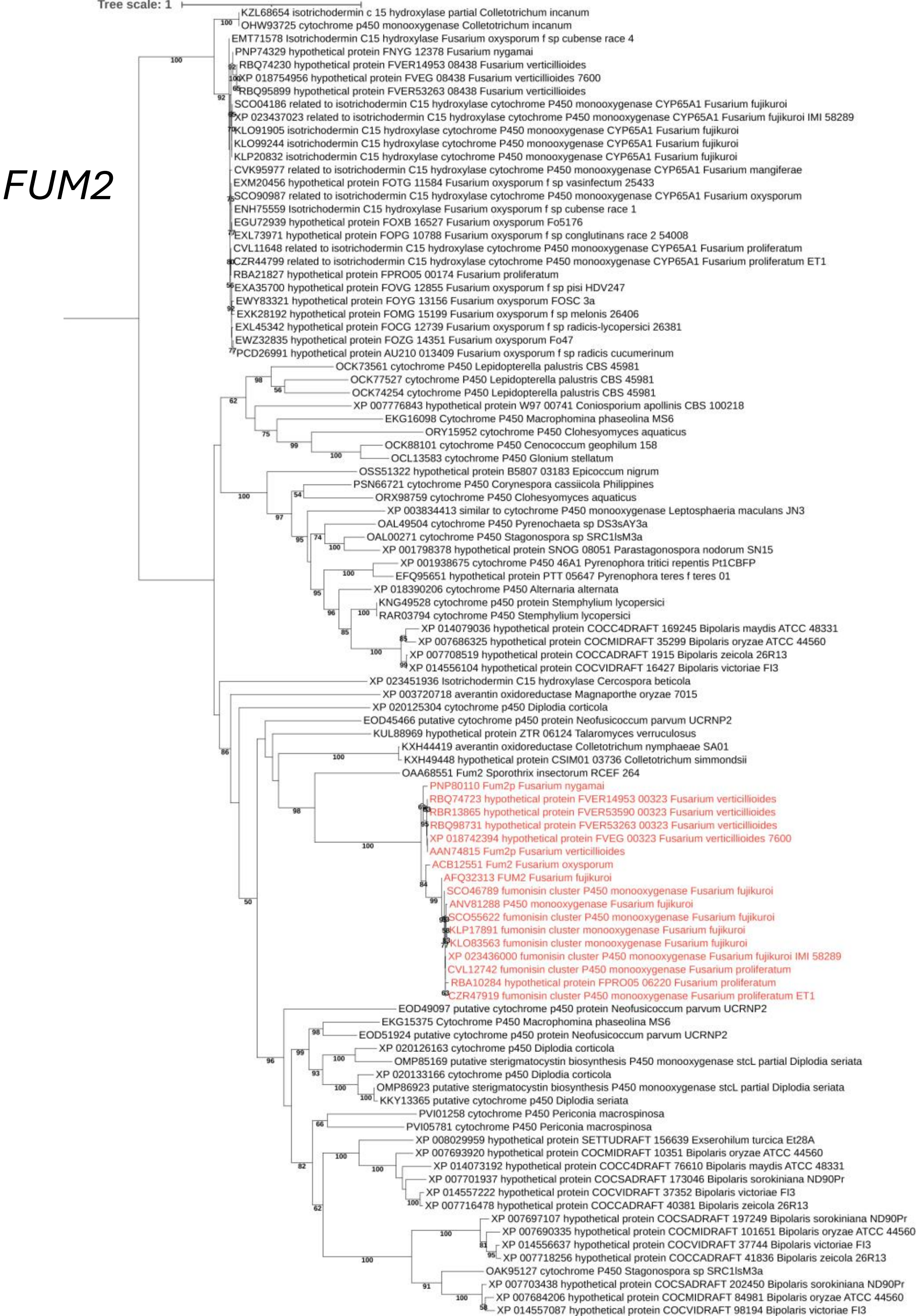

Tree scale: 1
FUM2
KZL68654 isotrichodermin c 15 hydroxylase partial Colletotrichum incanum
OHW93725 cytochrome p450 monooxygenase Colletotrichum incanum
EMT71578 Isotrichodermin C15 hydroxylase Fusarium oxysporum f sp cubense race 4
PNP74329 hypothetical protein FNYG 12378 Fusarium nygamai
RBQ74230 hypothetical protein FVER14953 08438 Fusarium verticillioides
XP 018754956 hypothetical protein FVEG 08438 Fusarium verticillioides 7600
RBQ95899 hypothetical protein FVER53263 08438 Fusarium verticillioides
SCO04186 related to isotrichodermin C15 hydroxylase cytochrome P450 monooxygenase CYP65A1 Fusarium fujikuroi
XP 023437023 related to isotrichodermin C15 hydroxylase cytochrome P450 monooxygenase CYP65A1 Fusarium fujikuroi IMI 58289
KLO91905 isotrichodermin C15 hydroxylase cytochrome P450 monooxygenase CYP65A1 Fusarium fujikuroi
KLO99244 isotrichodermin C15 hydroxylase cytochrome P450 monooxygenase CYP65A1 Fusarium fujikuroi
KLP20832 isotrichodermin C15 hydroxylase cytochrome P450 monooxygenase CYP65A1 Fusarium fujikuroi
CVK95977 related to isotrichodermin C15 hydroxylase cytochrome P450 monooxygenase CYP65A1 Fusarium mangiferae
EXM20456 hypothetical protein FOTG 11584 Fusarium oxysporum f sp vasinfectum 25433
SCO90987 related to isotrichodermin C15 hydroxylase cytochrome P450 monooxygenase CYP65A1 Fusarium oxysporum
ENH75559 Isotrichodermin C15 hydroxylase Fusarium oxysporum f sp cubense race 1
EGU72939 hypothetical protein FOXB 16527 Fusarium oxysporum Fo5176
EXL73971 hypothetical protein FOPG 10788 Fusarium oxysporum f sp conglutinans race 2 54008
CVL11648 related to isotrichodermin C15 hydroxylase cytochrome P450 monooxygenase CYP65A1 Fusarium proliferatum
CZR44799 related to isotrichodermin C15 hydroxylase cytochrome P450 monooxygenase CYP65A1 Fusarium proliferatum ET1
RBA21827 hypothetical protein FPRO05 00174 Fusarium proliferatum
EXA35700 hypothetical protein FOVG 12855 Fusarium oxysporum f sp pisi HDV247
EWY83321 hypothetical protein FOYG 13156 Fusarium oxysporum FOSC 3a
EXK28192 hypothetical protein FOMG 15199 Fusarium oxysporum f sp melonis 26406
EXL45342 hypothetical protein FOCG 12739 Fusarium oxysporum f sp radicis-lycopersici 26381
EWZ32835 hypothetical protein FOZG 14351 Fusarium oxysporum Fo47
PCD26991 hypothetical protein AU210 013409 Fusarium oxysporum f sp radicis cucumerinum
OCK73561 cytochrome P450 Lepidopterella palustris CBS 45981
OCK77527 cytochrome P450 Lepidopterella palustris CBS 45981
OCK74254 cytochrome P450 Lepidopterella palustris CBS 45981
XP 007776843 hypothetical protein W97 00741 Coniosporium apollinis CBS 100218
EKG16098 Cytochrome P450 Macrophomina phaseolina MS6
ORY15952 cytochrome P450 Clohesyomyces aquaticus
OCK88101 cytochrome P450 Cenococcum geophilum 158
OCL13583 cytochrome P450 Glonium stellatum
OSS51322 hypothetical protein B5807 03183 Epicoccum nigrum
PSN66721 cytochrome P450 Corynespora cassiicola Philippines
ORX98759 cytochrome P450 Clohesyomyces aquaticus
XP 003834413 similar to cytochrome P450 monooxygenase Leptosphaeria maculans JN3
OAL49504 cytochrome P450 Pyrenochaeta sp DS3sAY3a
OAL00271 cytochrome P450 Stagonospora sp SRC1lsM3a
XP 001798378 hypothetical protein SNOG 08051 Parastagonospora nodorum SN15
XP 001938675 cytochrome P450 46A1 Pyrenophora tritici repentis Pt1CBFP
EFQ95651 hypothetical protein PTT 05647 Pyrenophora teres f teres 01
XP 018390206 cytochrome P450 Alternaria alternata
KNG49528 cytochrome p450 protein Stemphylium lycopersici
RAR03794 cytochrome P450 Stemphylium lycopersici
XP 014079036 hypothetical protein COCC4DRAFT 169245 Bipolaris maydis ATCC 48331
XP 007686325 hypothetical protein COCMIDRAFT 35299 Bipolaris oryzae ATCC 44560
XP 007708519 hypothetical protein COCCADRAFT 1915 Bipolaris zeicola 26R13
XP 014556104 hypothetical protein COCVIDRAFT 16427 Bipolaris victoriae FI3
XP 023451936 Isotrichodermin C15 hydroxylase Cercospora beticola
XP 003720718 averantin oxidoreductase Magnaporthe oryzae 7015
XP 020125304 cytochrome p450 Diplodia corticola
EOD45466 putative cytochrome p450 protein Neofusicoccum parvum UCRNP2
KUL88969 hypothetical protein ZTR 06124 Talaromyces verruculosus
KXH44419 averantin oxidoreductase Colletotrichum nymphaeae SA01
KXH49448 hypothetical protein CSIM01 03736 Colletotrichum simmondsii
OAA68551 Fum2 Sporothrix insectorum RCEF 264
PNP80110 Fum2p Fusarium nygamai
RBQ74723 hypothetical protein FVER14953 00323 Fusarium verticillioides
RBR13865 hypothetical protein FVER53590 00323 Fusarium verticillioides
RBQ98731 hypothetical protein FVER53263 00323 Fusarium verticillioides
XP 018742394 hypothetical protein FVEG 00323 Fusarium verticillioides 7600
AAN74815 Fum2p Fusarium verticillioides
ACB12551 Fum2 Fusarium oxysporum
AFQ32313 FUM2 Fusarium fujikuroi
SCO46789 fumonisin cluster P450 monooxygenase Fusarium fujikuroi
ANV81288 P450 monooxygenase Fusarium fujikuroi
SCO55622 fumonisin cluster P450 monooxygenase Fusarium fujikuroi
KLP17891 fumonisin cluster monooxygenase Fusarium fujikuroi
KLO83563 fumonisin cluster monooxygenase Fusarium fujikuroi
XP 023436000 fumonisin cluster P450 monooxygenase Fusarium fujikuroi IMI 58289
CVL12742 fumonisin cluster P450 monooxygenase Fusarium proliferatum
RBA10284 hypothetical protein FPRO05 06220 Fusarium proliferatum
CZR47919 fumonisin cluster P450 monooxygenase Fusarium proliferatum ET1
EOD49097 putative cytochrome p450 protein Neofusicoccum parvum UCRNP2
EKG15375 Cytochrome P450 Macrophomina phaseolina MS6
EOD51924 putative cytochrome p450 protein Neofusicoccum parvum UCRNP2
XP 020126163 cytochrome p450 Diplodia corticola
OMP85169 putative sterigmatocystin biosynthesis P450 monooxygenase stcL partial Diplodia seriata
XP 020133166 cytochrome p450 Diplodia corticola
OMP86923 putative sterigmatocystin biosynthesis P450 monooxygenase stcL partial Diplodia seriata
KKY13365 putative cytochrome p450 Diplodia seriata
PVI01258 cytochrome P450 Periconia macrospinosa
PVI05781 cytochrome P450 Periconia macrospinosa
XP 008029959 hypothetical protein SETTUDRAFT 156639 Exserohilum turcica Et28A
XP 007693920 hypothetical protein COCMIDRAFT 10351 Bipolaris oryzae ATCC 44560
XP 014073192 hypothetical protein COCC4DRAFT 76610 Bipolaris maydis ATCC 48331
XP 007701937 hypothetical protein COCSADRAFT 173046 Bipolaris sorokiniana ND90Pr
XP 014557222 hypothetical protein COCVIDRAFT 37352 Bipolaris victoriae FI3
XP 007716478 hypothetical protein COCCADRAFT 40381 Bipolaris zeicola 26R13
XP 007697107 hypothetical protein COCSADRAFT 197249 Bipolaris sorokiniana ND90Pr
XP 007690335 hypothetical protein COCMIDRAFT 101651 Bipolaris oryzae ATCC 44560
XP 014556637 hypothetical protein COCVIDRAFT 37744 Bipolaris victoriae FI3
XP 007718256 hypothetical protein COCCADRAFT 41836 Bipolaris zeicola 26R13
OAK95127 cytochrome P450 Stagonospora sp SRC1lsM3a
XP 007703438 hypothetical protein COCSADRAFT 202450 Bipolaris sorokiniana ND90Pr
XP 007684206 hypothetical protein COCMIDRAFT 84981 Bipolaris oryzae ATCC 44560
XP 014557087 hypothetical protein COCVIDRAFT 98194 Bipolaris victoriae FI3

FUM3

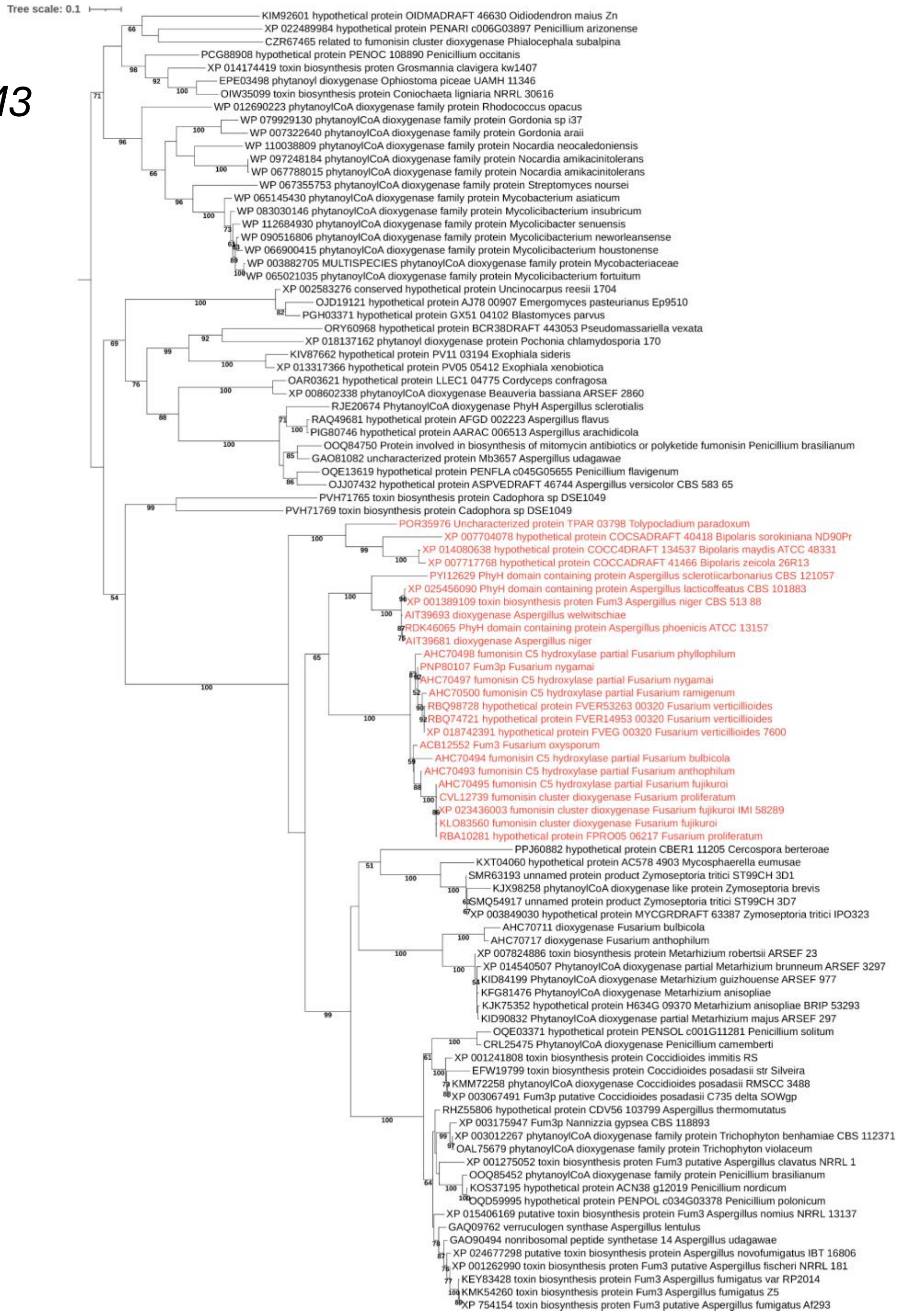
Tree scale: 0.1
KIM92601 hypothetical protein OIDMADRAFT 46630 Oidiodendron maius Zn
XP 022489984 hypothetical protein PENARI c006G03897 Penicillium arizonense
CZR67465 related to fumonisin cluster dioxygenase Phialocephala subalpina
PCG88908 hypothetical protein PENOC 108890 Penicillium occitanis
XP 014174419 toxin biosynthesis proten Grosmannia clavigera kw1407
EPE03498 phytanoyl dioxygenase Ophiostoma piceae UAMH 11346
OIW35099 toxin biosynthesis protein Coniochaeta ligniaria NRRL 30616
WP 012690223 phytanoylCoA dioxygenase family protein Rhodococcus opacus
WP 079929130 phytanoylCoA dioxygenase family protein Gordonia sp i37
WP 007322640 phytanoylCoA dioxygenase family protein Gordonia araii
WP 110038809 phytanoylCoA dioxygenase family protein Nocardia neocaledoniensis
WP 097248184 phytanoylCoA dioxygenase family protein Nocardia amikacinitolerans
WP 067788015 phytanoylCoA dioxygenase family protein Nocardia amikacinitolerans
WP 067355753 phytanoylCoA dioxygenase family protein Streptomyces noursei
WP 065145430 phytanoylCoA dioxygenase family protein Mycobacterium asiaticum
WP 083030146 phytanoylCoA dioxygenase family protein Mycolicibacterium insubricum
WP 112684930 phytanoylCoA dioxygenase family protein Mycolicibacter senuensis
WP 090516806 phytanoylCoA dioxygenase family protein Mycolicibacterium neworleansense
WP 066900415 phytanoylCoA dioxygenase family protein Mycolicibacterium houstonense
WP 003882705 MULTISPECIES phytanoylCoA dioxygenase family protein Mycobacteriaceae
WP 065021035 phytanoylCoA dioxygenase family protein Mycolicibacterium fortuitum
XP 002583276 conserved hypothetical protein Uncinocarpus reesii 1704
OJD19121 hypothetical protein AJ78 00907 Emergomyces pasteurianus Ep9510
PGH03371 hypothetical protein GX51 04102 Blastomyces parvus
ORY60968 hypothetical protein BCR38DRAFT 443053 Pseudomassariella vexata
XP 018137162 phytanoyl dioxygenase protein Pochonia chlamydosporia 170
KIV87662 hypothetical protein PV11 03194 Exophiala sideris
XP 013317366 hypothetical protein PV05 05412 Exophiala xenobiotica
OAR03621 hypothetical protein LLEC1 04775 Cordyceps confragosa
XP 008602338 phytanoylCoA dioxygenase Beauveria bassiana ARSEF 2860
RJE20674 PhytanoylCoA dioxygenase PhyH Aspergillus sclerotialis
RAQ49681 hypothetical protein AFGD 002223 Aspergillus flavus
PIG80746 hypothetical protein AARAC 006513 Aspergillus arachidicola
OOQ84750 Protein involved in biosynthesis of mitomycin antibiotics or polyketide fumonisin Penicillium brasilianum
GAO81082 uncharacterized protein Mb3657 Aspergillus udagawae
OQE13619 hypothetical protein PENFLA c045G05655 Penicillium flavigenum
OJJ07432 hypothetical protein ASPVEDRAFT 46744 Aspergillus versicolor CBS 583 65
PVH71765 toxin biosynthesis protein Cadophora sp DSE1049
PVH71769 toxin biosynthesis protein Cadophora sp DSE1049
POR35976 Uncharacterized protein TPAR 03798 Tolypocladium paradoxum
XP 007704078 hypothetical protein COCSADRAFT 40418 Bipolaris sorokiniana ND90Pr
XP 014080638 hypothetical protein COCC4DRAFT 134537 Bipolaris maydis ATCC 48331
XP 007717768 hypothetical protein COCCADRAFT 41466 Bipolaris zeicola 26R13
PYI12629 PhyH domain containing protein Aspergillus sclerotiicarbonarius CBS 121057
XP 025456090 PhyH domain containing protein Aspergillus lacticoffeatus CBS 101883
XP 001389109 toxin biosynthesis proten Fum3 Aspergillus niger CBS 513 88
AIT39693 dioxygenase Aspergillus welwitschiae
RDK46065 PhyH domain containing protein Aspergillus phoenicis ATCC 13157
AIT39681 dioxygenase Aspergillus niger
AHC70498 fumonisin C5 hydroxylase partial Fusarium phyllophilum
PNP80107 Fum3p Fusarium nygamai
AHC70497 fumonisin C5 hydroxylase partial Fusarium nygamai
AHC70500 fumonisin C5 hydroxylase partial Fusarium ramigenum
RBQ98728 hypothetical protein FVER53263 00320 Fusarium verticillioides
RBQ74721 hypothetical protein FVER14953 00320 Fusarium verticillioides
XP 018742391 hypothetical protein FVEG 00320 Fusarium verticillioides 7600
ACB12552 Fum3 Fusarium oxysporum
AHC70494 fumonisin C5 hydroxylase partial Fusarium bulbicola
AHC70493 fumonisin C5 hydroxylase partial Fusarium anthophilum
AHC70495 fumonisin C5 hydroxylase partial Fusarium fujikuroi
CVL12739 fumonisin cluster dioxygenase Fusarium proliferatum
XP 023436003 fumonisin cluster dioxygenase Fusarium fujikuroi IMI 58289
KLO83560 fumonisin cluster dioxygenase Fusarium fujikuroi
RBA10281 hypothetical protein FPRO05 06217 Fusarium proliferatum
PPJ60882 hypothetical protein CBER1 11205 Cercospora berteroae
KXT04060 hypothetical protein AC578 4903 Mycosphaerella eumusae
SMR63193 unnamed protein product Zymoseptoria tritici ST99CH 3D1
KJX98258 phytanoylCoA dioxygenase like protein Zymoseptoria brevis
SMQ54917 unnamed protein product Zymoseptoria tritici ST99CH 3D7
XP 003849030 hypothetical protein MYCGRDRAFT 63387 Zymoseptoria tritici IPO323
AHC70711 dioxygenase Fusarium bulbicola
AHC70717 dioxygenase Fusarium anthophilum
XP 007824886 toxin biosynthesis protein Metarhizium robertsii ARSEF 23
XP 014540507 PhytanoylCoA dioxygenase partial Metarhizium brunneum ARSEF 3297
KID84199 PhytanoylCoA dioxygenase Metarhizium guizhouense ARSEF 977
KFG81476 PhytanoylCoA dioxygenase Metarhizium anisopliae
KJK75352 hypothetical protein H634G 09370 Metarhizium anisopliae BRIP 53293
KID90832 PhytanoylCoA dioxygenase partial Metarhizium majus ARSEF 297
OQE03371 hypothetical protein PENSOL c001G11281 Penicillium solitum
CRL25475 PhytanoylCoA dioxygenase Penicillium camemberti
XP 001241808 toxin biosynthesis protein Coccidioides immitis RS
EFW19799 toxin biosynthesis protein Coccidioides posadasii str Silveira
KMM72258 phytanoylCoA dioxygenase Coccidioides posadasii RMSCC 3488
XP 003067491 Fum3p putative Coccidioides posadasii C735 delta SOWgp
RHZ55806 hypothetical protein CDV56 103799 Aspergillus thermomutatus
XP 003175947 Fum3p Nannizzia gypsea CBS 118893
XP 003012267 phytanoylCoA dioxygenase family protein Trichophyton benhamiae CBS 112371
OAL75679 phytanoylCoA dioxygenase family protein Trichophyton violaceum
XP 001275052 toxin biosynthesis proten Fum3 putative Aspergillus clavatus NRRL 1
OOQ85452 phytanoylCoA dioxygenase family protein Penicillium brasilianum
KOS37195 hypothetical protein ACN38 g12019 Penicillium nordicum
OQD59995 hypothetical protein PENPOL c034G03378 Penicillium polonicum
XP 015406169 putative toxin biosynthesis protein Fum3 Aspergillus nomius NRRL 13137
GAQ09762 verruculogen synthase Aspergillus lentulus
GAO90494 nonribosomal peptide synthetase 14 Aspergillus udagawae
XP 024677298 putative toxin biosynthesis protein Aspergillus novofumigatus IBT 16806
XP 001262990 toxin biosynthesis proten Fum3 putative Aspergillus fischeri NRRL 181
KEY83428 toxin biosynthesis protein Fum3 Aspergillus fumigatus var RP2014
KMK54260 toxin biosynthesis protein Fum3 Aspergillus fumigatus Z5
XP 754154 toxin biosynthesis proten Fum3 putative Aspergillus fumigatus Af293

FUM6

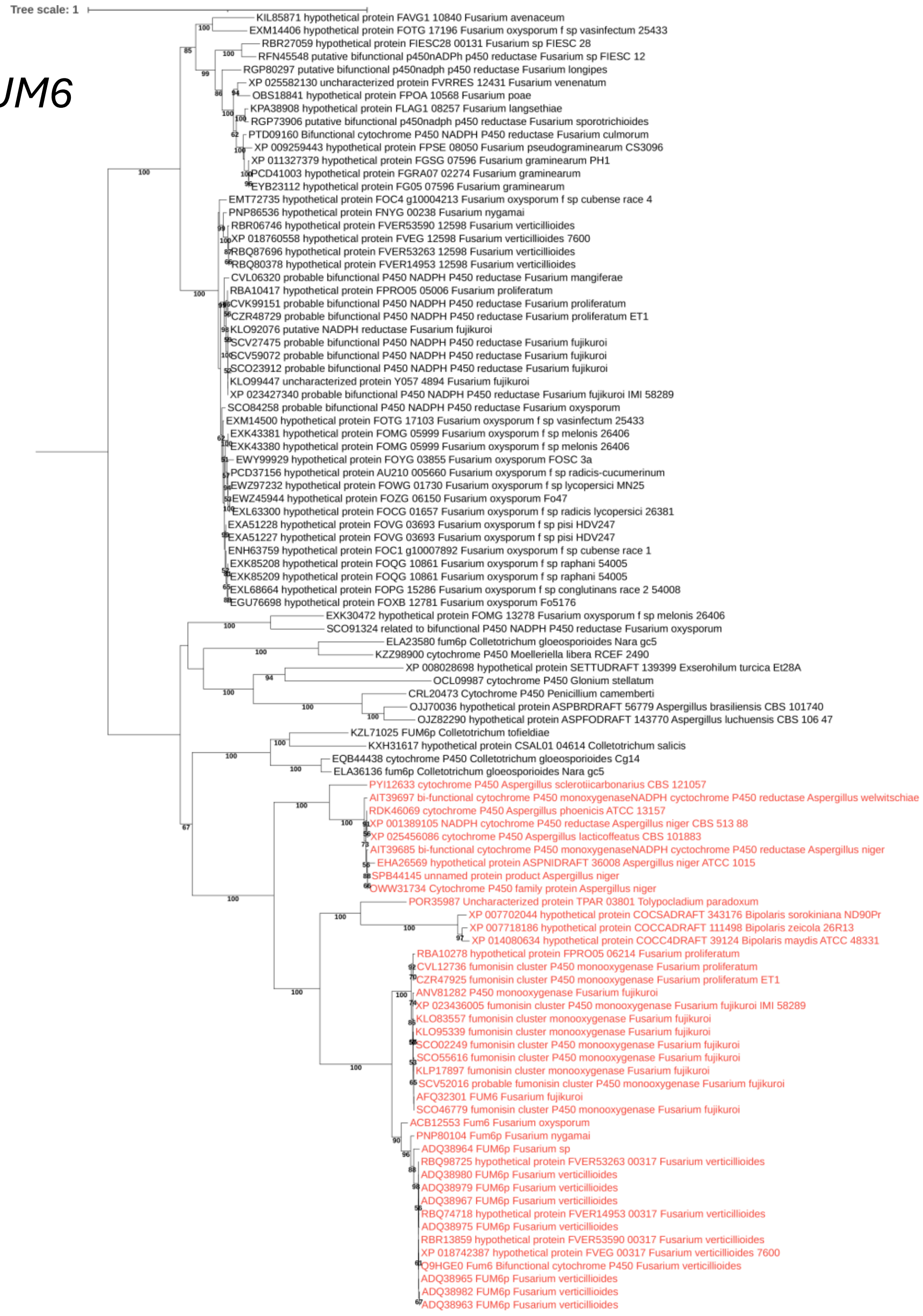
Tree scale: 1
KIL85871 hypothetical protein FAVG1 10840 Fusarium avenaceum
EXM14406 hypothetical protein FOTG 17196 Fusarium oxysporum f sp vasinfectum 25433
RBR27059 hypothetical protein FIESC28 00131 Fusarium sp FIESC 28
RFN45548 putative bifunctional p450nADPh p450 reductase Fusarium sp FIESC 12
RGP80297 putative bifunctional p450nadph p450 reductase Fusarium longipes
XP 025582130 uncharacterized protein FVRRES 12431 Fusarium venenatum
OBS18841 hypothetical protein FPOA 10568 Fusarium poae
KPA38908 hypothetical protein FLAG1 08257 Fusarium langsethiae
RGP73906 putative bifunctional p450nadph p450 reductase Fusarium sporotrichioides
PTD09160 Bifunctional cytochrome P450 NADPH P450 reductase Fusarium culmorum
XP 009259443 hypothetical protein FPSE 08050 Fusarium pseudograminearum CS3096
XP 011327379 hypothetical protein FGSG 07596 Fusarium graminearum PH1
PCD41003 hypothetical protein FGRA07 02274 Fusarium graminearum
EYB23112 hypothetical protein FG05 07596 Fusarium graminearum
EMT72735 hypothetical protein FOC4 g10004213 Fusarium oxysporum f sp cubense race 4
PNP86536 hypothetical protein FNYG 00238 Fusarium nygamai
RBR06746 hypothetical protein FVER53590 12598 Fusarium verticillioides
XP 018760558 hypothetical protein FVEG 12598 Fusarium verticillioides 7600
RBQ87696 hypothetical protein FVER53263 12598 Fusarium verticillioides
RBQ80378 hypothetical protein FVER14953 12598 Fusarium verticillioides
CVL06320 probable bifunctional P450 NADPH P450 reductase Fusarium mangiferae
RBA10417 hypothetical protein FPRO05 05006 Fusarium proliferatum
CVK99151 probable bifunctional P450 NADPH P450 reductase Fusarium proliferatum
CZR48729 probable bifunctional P450 NADPH P450 reductase Fusarium proliferatum ET1
KLO92076 putative NADPH reductase Fusarium fujikuroi
SCV27475 probable bifunctional P450 NADPH P450 reductase Fusarium fujikuroi
SCV59072 probable bifunctional P450 NADPH P450 reductase Fusarium fujikuroi
SCO23912 probable bifunctional P450 NADPH P450 reductase Fusarium fujikuroi
KLO99447 uncharacterized protein Y057 4894 Fusarium fujikuroi
XP 023427340 probable bifunctional P450 NADPH P450 reductase Fusarium fujikuroi IMI 58289
SCO84258 probable bifunctional P450 NADPH P450 reductase Fusarium oxysporum
EXM14500 hypothetical protein FOTG 17103 Fusarium oxysporum f sp vasinfectum 25433
EXK43381 hypothetical protein FOMG 05999 Fusarium oxysporum f sp melonis 26406
EXK43380 hypothetical protein FOMG 05999 Fusarium oxysporum f sp melonis 26406
EWY99929 hypothetical protein FOYG 03855 Fusarium oxysporum FOSC 3a
PCD37156 hypothetical protein AU210 005660 Fusarium oxysporum f sp radicis-cucumerinum
EWZ97232 hypothetical protein FOWG 01730 Fusarium oxysporum f sp lycopersici MN25
EWZ45944 hypothetical protein FOZG 06150 Fusarium oxysporum Fo47
EXL63300 hypothetical protein FOCG 01657 Fusarium oxysporum f sp radicis lycopersici 26381
EXA51228 hypothetical protein FOVG 03693 Fusarium oxysporum f sp pisi HDV247
EXA51227 hypothetical protein FOVG 03693 Fusarium oxysporum f sp pisi HDV247
ENH63759 hypothetical protein FOC1 g10007892 Fusarium oxysporum f sp cubense race 1
EXK85208 hypothetical protein FOQG 10861 Fusarium oxysporum f sp raphani 54005
EXK85209 hypothetical protein FOQG 10861 Fusarium oxysporum f sp raphani 54005
EXL68664 hypothetical protein FOPG 15286 Fusarium oxysporum f sp conglutinans race 2 54008
EGU76698 hypothetical protein FOXB 12781 Fusarium oxysporum Fo5176
EXK30472 hypothetical protein FOMG 13278 Fusarium oxysporum f sp melonis 26406
SCO91324 related to bifunctional P450 NADPH P450 reductase Fusarium oxysporum
ELA23580 fum6p Colletotrichum gloeosporioides Nara gc5
KZZ98900 cytochrome P450 Moelleriella libera RCEF 2490
XP 008028698 hypothetical protein SETTUDRAFT 139399 Exserohilum turcica Et28A
OCL09987 cytochrome P450 Glonium stellatum
CRL20473 Cytochrome P450 Penicillium camemberti
OJJ70036 hypothetical protein ASPBRDRAFT 56779 Aspergillus brasiliensis CBS 101740
OJZ82290 hypothetical protein ASPFODRAFT 143770 Aspergillus luchuensis CBS 106 47
KZL71025 FUM6p Colletotrichum tofieldiae
KXH31617 hypothetical protein CSAL01 04614 Colletotrichum salicis
EQB44438 cytochrome P450 Colletotrichum gloeosporioides Cg14
ELA36136 fum6p Colletotrichum gloeosporioides Nara gc5
PYI12633 cytochrome P450 Aspergillus sclerotiicarbonarius CBS 121057
AIT39697 bi-functional cytochrome P450 monoxygenaseNADPH cyctochrome P450 reductase Aspergillus welwitschiae
RDK46069 cytochrome P450 Aspergillus phoenicis ATCC 13157
XP 001389105 NADPH cytochrome P450 reductase Aspergillus niger CBS 513 88
XP 025456086 cytochrome P450 Aspergillus lacticoffeatus CBS 101883
AIT39685 bi-functional cytochrome P450 monoxygenaseNADPH cyctochrome P450 reductase Aspergillus niger
EHA26569 hypothetical protein ASPNIDRAFT 36008 Aspergillus niger ATCC 1015
SPB44145 unnamed protein product Aspergillus niger
OWW31734 Cytochrome P450 family protein Aspergillus niger
POR35987 Uncharacterized protein TPAR 03801 Tolypocladium paradoxum
XP 007702044 hypothetical protein COCSADRAFT 343176 Bipolaris sorokiniana ND90Pr
XP 007718186 hypothetical protein COCCADRAFT 111498 Bipolaris zeicola 26R13
XP 014080634 hypothetical protein COCC4DRAFT 39124 Bipolaris maydis ATCC 48331
RBA10278 hypothetical protein FPRO05 06214 Fusarium proliferatum
CVL12736 fumonisin cluster P450 monooxygenase Fusarium proliferatum
CZR47925 fumonisin cluster P450 monooxygenase Fusarium proliferatum ET1
ANV81282 P450 monooxygenase Fusarium fujikuroi
XP 023436005 fumonisin cluster P450 monooxygenase Fusarium fujikuroi IMI 58289
KLO83557 fumonisin cluster monooxygenase Fusarium fujikuroi
KLO95339 fumonisin cluster monooxygenase Fusarium fujikuroi
SCO02249 fumonisin cluster P450 monooxygenase Fusarium fujikuroi
SCO55616 fumonisin cluster P450 monooxygenase Fusarium fujikuroi
KLP17897 fumonisin cluster monooxygenase Fusarium fujikuroi
SCV52016 probable fumonisin cluster P450 monooxygenase Fusarium fujikuroi
AFQ32301 FUM6 Fusarium fujikuroi
SCO46779 fumonisin cluster P450 monooxygenase Fusarium fujikuroi
ACB12553 Fum6 Fusarium oxysporum
PNP80104 Fum6p Fusarium nygamai
ADQ38964 FUM6p Fusarium sp
RBQ98725 hypothetical protein FVER53263 00317 Fusarium verticillioides
ADQ38980 FUM6p Fusarium verticillioides
ADQ38979 FUM6p Fusarium verticillioides
ADQ38967 FUM6p Fusarium verticillioides
RBQ74718 hypothetical protein FVER14953 00317 Fusarium verticillioides
ADQ38975 FUM6p Fusarium verticillioides
RBR13859 hypothetical protein FVER53590 00317 Fusarium verticillioides
XP 018742387 hypothetical protein FVEG 00317 Fusarium verticillioides 7600
Q9HGE0 Fum6 Bifunctional cytochrome P450 Fusarium verticillioides
ADQ38965 FUM6p Fusarium verticillioides
ADQ38982 FUM6p Fusarium verticillioides
ADQ38963 FUM6p Fusarium verticillioides

FUM7

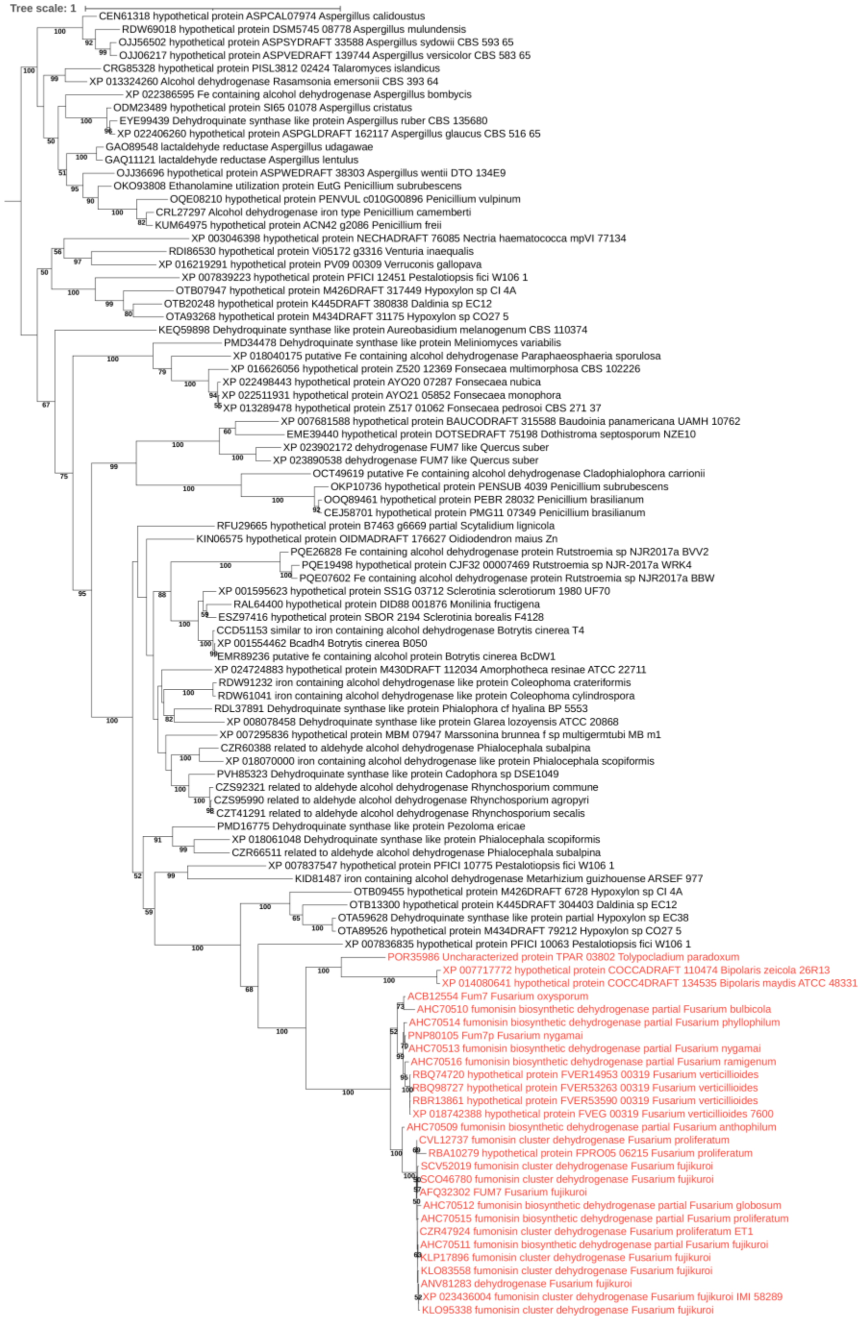

Tree scale: 1
CEN61318 hypothetical protein ASPCAL07974 Aspergillus calidoustus
RDW69018 hypothetical protein DSM5745 08778 Aspergillus mulundensis
OJJ56502 hypothetical protein ASPSYDRAFT 33588 Aspergillus sydowii CBS 593 65
OJJ06217 hypothetical protein ASPVEDRAFT 139744 Aspergillus versicolor CBS 583 65
CRG85328 hypothetical protein PISL3812 02424 Talaromyces islandicus
XP 013324260 Alcohol dehydrogenase Rasamsonia emersonii CBS 393 64
XP 022386595 Fe containing alcohol dehydrogenase Aspergillus bombycis
ODM23489 hypothetical protein SI65 01078 Aspergillus cristatus
EYE99439 Dehydroquinate synthase like protein Aspergillus ruber CBS 135680
XP 022406260 hypothetical protein ASPGLDRAFT 162117 Aspergillus glaucus CBS 516 65
GAO89548 lactaldehyde reductase Aspergillus udagawae
GAQ11121 lactaldehyde reductase Aspergillus lentulus
OJJ36696 hypothetical protein ASPWEDRAFT 38303 Aspergillus wentii DTO 134E9
OKO93808 Ethanolamine utilization protein EutG Penicillium subrubescens
OQE08210 hypothetical protein PENVUL c010G00896 Penicillium vulpinum
CRL27297 Alcohol dehydrogenase iron type Penicillium camemberti
KUM64975 hypothetical protein ACN42 g2086 Penicillium freii
XP 003046398 hypothetical protein NECHADRAFT 76085 Nectria haematococca mpVI 77134
RDI86530 hypothetical protein Vi05172 g3316 Venturia inaequalis
XP 016219291 hypothetical protein PV09 00309 Verruconis gallopava
XP 007839223 hypothetical protein PFICI 12451 Pestalotiopsis fici W106 1
OTB07947 hypothetical protein M426DRAFT 317449 Hypoxylon sp CI 4A
OTB20248 hypothetical protein K445DRAFT 380838 Daldinia sp EC12
OTA93268 hypothetical protein M434DRAFT 31175 Hypoxylon sp CO27 5
KEQ59898 Dehydroquinate synthase like protein Aureobasidium melanogenum CBS 110374
PMD34478 Dehydroquinate synthase like protein Meliniomyces variabilis
XP 018040175 putative Fe containing alcohol dehydrogenase Paraphaeosphaeria sporulosa
XP 016626056 hypothetical protein Z520 12369 Fonsecaea multimorphosa CBS 102226
XP 022498443 hypothetical protein AYO20 07287 Fonsecaea nubica
XP 022511931 hypothetical protein AYO21 05852 Fonsecaea monophora
XP 013289478 hypothetical protein Z517 01062 Fonsecaea pedrosoi CBS 271 37
XP 007681588 hypothetical protein BAUCODRAFT 315588 Baudoinia panamericana UAMH 10762
EME39440 hypothetical protein DOTSEDRAFT 75198 Dothistroma septosporum NZE10
XP 023902172 dehydrogenase FUM7 like Quercus suber
XP 023890538 dehydrogenase FUM7 like Quercus suber
OCT49619 putative Fe containing alcohol dehydrogenase Cladophialophora carrionii
OKP10736 hypothetical protein PENSUB 4039 Penicillium subrubescens
OOQ89461 hypothetical protein PEBR 28032 Penicillium brasilianum
CEJ58701 hypothetical protein PMG11 07349 Penicillium brasilianum
RFU29665 hypothetical protein B7463 g6669 partial Scytalidium lignicola
KIN06575 hypothetical protein OIDMADRAFT 176627 Oidiodendron maius Zn
PQE26828 Fe containing alcohol dehydrogenase protein Rutstroemia sp NJR2017a BVV2
PQE19498 hypothetical protein CJF32 00007469 Rutstroemia sp NJR-2017a WRK4
PQE07602 Fe containing alcohol dehydrogenase protein Rutstroemia sp NJR2017a BBW
XP 001595623 hypothetical protein SS1G 03712 Sclerotinia sclerotiorum 1980 UF70
RAL64400 hypothetical protein DID88 001876 Monilinia fructigena
ESZ97416 hypothetical protein SBOR 2194 Sclerotinia borealis F4128
CCD51153 similar to iron containing alcohol dehydrogenase Botrytis cinerea T4
XP 001554462 Bcadh4 Botrytis cinerea B050
EMR89236 putative fe containing alcohol protein Botrytis cinerea BcDW1
XP 024724883 hypothetical protein M430DRAFT 112034 Amorphotheca resinae ATCC 22711
RDW91232 iron containing alcohol dehydrogenase like protein Coleophoma crateriformis
RDW61041 iron containing alcohol dehydrogenase like protein Coleophoma cylindrospora
RDL37891 Dehydroquinate synthase like protein Phialophora cf hyalina BP 5553
XP 008078458 Dehydroquinate synthase like protein Glarea lozoyensis ATCC 20868
XP 007295836 hypothetical protein MBM 07947 Marssonina brunnea f sp multigermtubi MB m1
CZR60388 related to aldehyde alcohol dehydrogenase Phialocephala subalpina
XP 018070000 iron containing alcohol dehydrogenase like protein Phialocephala scopiformis
PVH85323 Dehydroquinate synthase like protein Cadophora sp DSE1049
CZS92321 related to aldehyde alcohol dehydrogenase Rhynchosporium commune
CZS95990 related to aldehyde alcohol dehydrogenase Rhynchosporium agropyri
CZT41291 related to aldehyde alcohol dehydrogenase Rhynchosporium secalis
PMD16775 Dehydroquinate synthase like protein Pezoloma ericae
XP 018061048 Dehydroquinate synthase like protein Phialocephala scopiformis
CZR66511 related to aldehyde alcohol dehydrogenase Phialocephala subalpina
XP 007837547 hypothetical protein PFICI 10775 Pestalotiopsis fici W106 1
KID81487 iron containing alcohol dehydrogenase Metarhizium guizhouense ARSEF 977
OTB09455 hypothetical protein M426DRAFT 6728 Hypoxylon sp CI 4A
OTB13300 hypothetical protein K445DRAFT 304403 Daldinia sp EC12
OTA59628 Dehydroquinate synthase like protein partial Hypoxylon sp EC38
OTA89526 hypothetical protein M434DRAFT 79212 Hypoxylon sp CO27 5
XP 007836835 hypothetical protein PFICI 10063 Pestalotiopsis fici W106 1
POR35986 Uncharacterized protein TPAR 03802 Tolypocladium paradoxum
XP 007717772 hypothetical protein COCCADRAFT 110474 Bipolaris zeicola 26R13
XP 014080641 hypothetical protein COCC4DRAFT 134535 Bipolaris maydis ATCC 48331
ACB12554 Fum7 Fusarium oxysporum
AHC70510 fumonisin biosynthetic dehydrogenase partial Fusarium bulbicola
AHC70514 fumonisin biosynthetic dehydrogenase partial Fusarium phyllophilum
PNP80105 Fum7p Fusarium nygamai
AHC70513 fumonisin biosynthetic dehydrogenase partial Fusarium nygamai
AHC70516 fumonisin biosynthetic dehydrogenase partial Fusarium ramigenum
RBQ74720 hypothetical protein FVER14953 00319 Fusarium verticillioides
RBQ98727 hypothetical protein FVER53263 00319 Fusarium verticillioides
RBR13861 hypothetical protein FVER53590 00319 Fusarium verticillioides
XP 018742388 hypothetical protein FVEG 00319 Fusarium verticillioides 7600
AHC70509 fumonisin biosynthetic dehydrogenase partial Fusarium anthophilum
CVL12737 fumonisin cluster dehydrogenase Fusarium proliferatum
RBA10279 hypothetical protein FPRO05 06215 Fusarium proliferatum
SCV52019 fumonisin cluster dehydrogenase Fusarium fujikuroi
SCO46780 fumonisin cluster dehydrogenase Fusarium fujikuroi
AFQ32302 FUM7 Fusarium fujikuroi
AHC70512 fumonisin biosynthetic dehydrogenase partial Fusarium globosum
AHC70515 fumonisin biosynthetic dehydrogenase partial Fusarium proliferatum
CZR47924 fumonisin cluster dehydrogenase Fusarium proliferatum ET1
AHC70511 fumonisin biosynthetic dehydrogenase partial Fusarium fujikuroi
KLP17896 fumonisin cluster dehydrogenase Fusarium fujikuroi
KLO83558 fumonisin cluster dehydrogenase Fusarium fujikuroi
ANV81283 dehydrogenase Fusarium fujikuroi
XP 023436004 fumonisin cluster dehydrogenase Fusarium fujikuroi IMI 58289
KLO95338 fumonisin cluster dehydrogenase Fusarium fujikuroi

FUM8

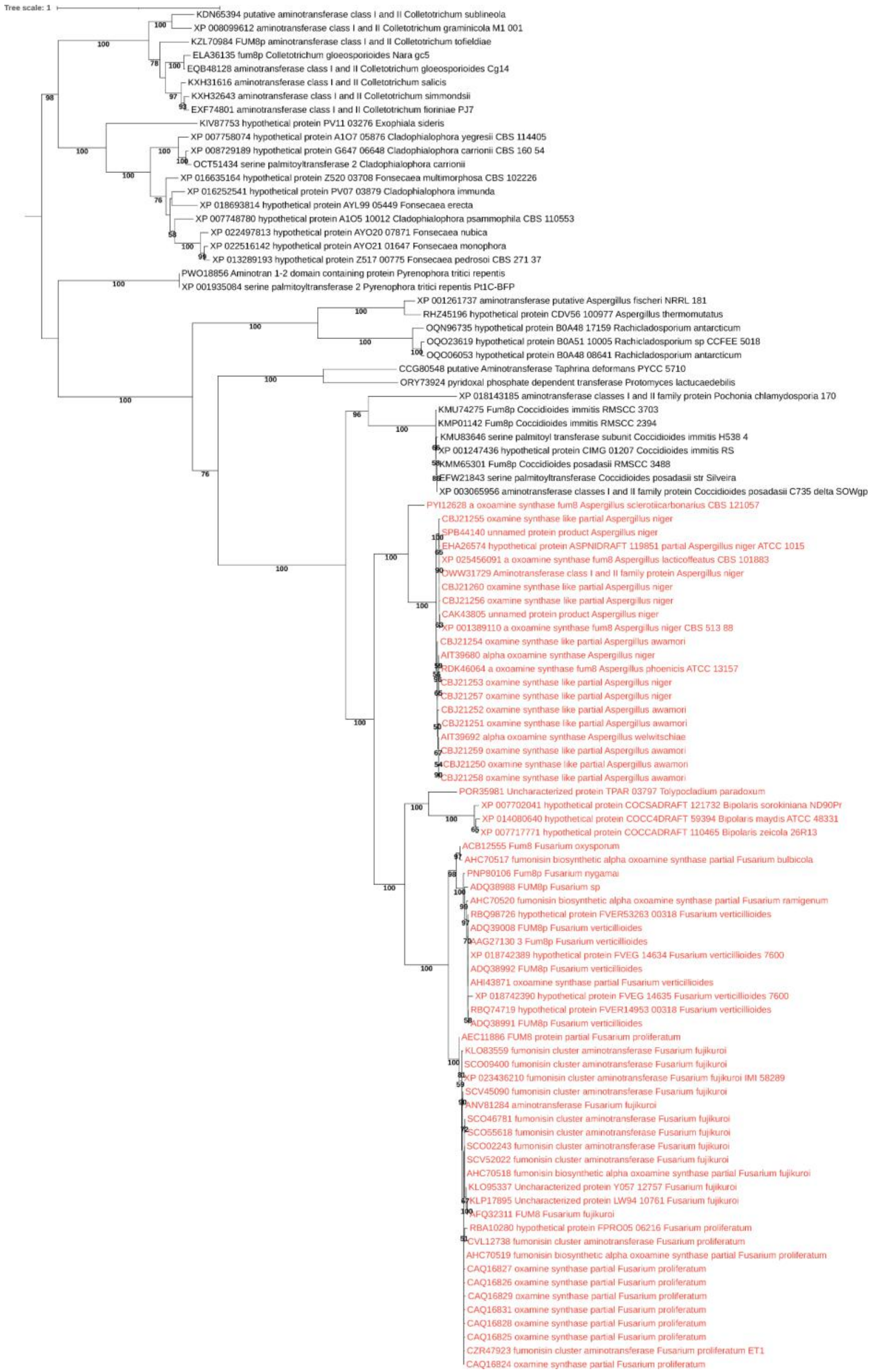

Tree scale: 1
KDN65394 putative aminotransferase class I and II Colletotrichum sublineola
XP 008099612 aminotransferase class I and II Colletotrichum graminicola M1 001
KZL70984 FUM8p aminotransferase class I and II Colletotrichum tofieldiae
ELA36135 fum8p Colletotrichum gloeosporioides Nara gc5
EQB48128 aminotransferase class I and II Colletotrichum gloeosporioides Cg14
KXH31616 aminotransferase class I and II Colletotrichum salicis
KXH32643 aminotransferase class I and II Colletotrichum simmondsii
EXF74801 aminotransferase class I and II Colletotrichum fioriniae PJ7
KIV87753 hypothetical protein PV11 03276 Exophiala sideris
XP 007758074 hypothetical protein A1O7 05876 Cladophialophora yegresii CBS 114405
XP 008729189 hypothetical protein G647 06648 Cladophialophora carrionii CBS 160 54
OCT51434 serine palmitoyltransferase 2 Cladophialophora carrionii
XP 016635164 hypothetical protein Z520 03708 Fonsecaea multimorphosa CBS 102226
XP 016252541 hypothetical protein PV07 03879 Cladophialophora immunda
XP 018693814 hypothetical protein AYL99 05449 Fonsecaea erecta
XP 007748780 hypothetical protein A1O5 10012 Cladophialophora psammophila CBS 110553
XP 022497813 hypothetical protein AYO20 07871 Fonsecaea nubica
XP 022516142 hypothetical protein AYO21 01647 Fonsecaea monophora
XP 013289193 hypothetical protein Z517 00775 Fonsecaea pedrosoi CBS 271 37
PWO18856 Aminotran 1-2 domain containing protein Pyrenophora tritici repentis
XP 001935084 serine palmitoyltransferase 2 Pyrenophora tritici repentis Pt1C-BFP
XP 001261737 aminotransferase putative Aspergillus fischeri NRRL 181
RHZ45196 hypothetical protein CDV56 100977 Aspergillus thermomutatus
OQN96735 hypothetical protein B0A48 17159 Rachicladosporium antarcticum
OQO23619 hypothetical protein B0A51 10005 Rachicladosporium sp CCFEE 5018
OQO06053 hypothetical protein B0A48 08641 Rachicladosporium antarcticum
CCG80548 putative Aminotransferase Taphrina deformans PYCC 5710
ORY73924 pyridoxal phosphate dependent transferase Protomyces lactucaedebilis
XP 018143185 aminotransferase classes I and II family protein Pochonia chlamydosporia 170
KMU74275 Fum8p Coccidioides immitis RMSCC 3703
KMP01142 Fum8p Coccidioides immitis RMSCC 2394
KMU83646 serine palmitoyl transferase subunit Coccidioides immitis H538 4
XP 001247436 hypothetical protein CIMG 01207 Coccidioides immitis RS
KMM65301 Fum8p Coccidioides posadasii RMSCC 3488
EFW21843 serine palmitoyltransferase Coccidioides posadasii str Silveira
XP 003065956 aminotransferase classes I and II family protein Coccidioides posadasii C735 delta SOWgp
PYI12628 a oxoamine synthase fum8 Aspergillus sclerotiicarbonarius CBS 121057
CBJ21255 oxamine synthase like partial Aspergillus niger
SPB44140 unnamed protein product Aspergillus niger
EHA26574 hypothetical protein ASPNIDRAFT 119851 partial Aspergillus niger ATCC 1015
XP 025456091 a oxoamine synthase fum8 Aspergillus lacticoffeatus CBS 101883
OWW31729 Aminotransferase class I and II family protein Aspergillus niger
CBJ21260 oxamine synthase like partial Aspergillus niger
CBJ21256 oxamine synthase like partial Aspergillus niger
CAK43805 unnamed protein product Aspergillus niger
XP 001389110 a oxoamine synthase fum8 Aspergillus niger CBS 513 88
CBJ21254 oxamine synthase like partial Aspergillus awamori
AIT39680 alpha oxoamine synthase Aspergillus niger
RDK46064 a oxoamine synthase fum8 Aspergillus phoenicis ATCC 13157
CBJ21253 oxamine synthase like partial Aspergillus niger
CBJ21257 oxamine synthase like partial Aspergillus niger
CBJ21252 oxamine synthase like partial Aspergillus awamori
CBJ21251 oxamine synthase like partial Aspergillus awamori
AIT39692 alpha oxoamine synthase Aspergillus welwitschiae
CBJ21259 oxamine synthase like partial Aspergillus awamori
CBJ21250 oxamine synthase like partial Aspergillus awamori
CBJ21258 oxamine synthase like partial Aspergillus awamori
POR35981 Uncharacterized protein TPAR 03797 Tolypocladium paradoxum
XP 007702041 hypothetical protein COCSADRAFT 121732 Bipolaris sorokiniana ND90Pr
XP 014080640 hypothetical protein COCC4DRAFT 59394 Bipolaris maydis ATCC 48331
XP 007717771 hypothetical protein COCCADRAFT 110465 Bipolaris zeicola 26R13
ACB12555 Fum8 Fusarium oxysporum
AHC70517 fumonisin biosynthetic alpha oxoamine synthase partial Fusarium bulbicola
PNP80106 Fum8p Fusarium nygamai
ADQ38988 FUM8p Fusarium sp
AHC70520 fumonisin biosynthetic alpha oxoamine synthase partial Fusarium ramigenum
RBQ98726 hypothetical protein FVER53263 00318 Fusarium verticillioides
ADQ39008 FUM8p Fusarium verticillioides
AAG27130 3 Fum8p Fusarium verticillioides
XP 018742389 hypothetical protein FVEG 14634 Fusarium verticillioides 7600
ADQ38992 FUM8p Fusarium verticillioides
AHI43871 oxoamine synthase partial Fusarium verticillioides
XP 018742390 hypothetical protein FVEG 14635 Fusarium verticillioides 7600
RBQ74719 hypothetical protein FVER14953 00318 Fusarium verticillioides
ADQ38991 FUM8p Fusarium verticillioides
AEC11886 FUM8 protein partial Fusarium proliferatum
KLO83559 fumonisin cluster aminotransferase Fusarium fujikuroi
SCO09400 fumonisin cluster aminotransferase Fusarium fujikuroi
XP 023436210 fumonisin cluster aminotransferase Fusarium fujikuroi IMI 58289
SCV45090 fumonisin cluster aminotransferase Fusarium fujikuroi
ANV81284 aminotransferase Fusarium fujikuroi
SCO46781 fumonisin cluster aminotransferase Fusarium fujikuroi
SCO55618 fumonisin cluster aminotransferase Fusarium fujikuroi
SCO02243 fumonisin cluster aminotransferase Fusarium fujikuroi
SCV52022 fumonisin cluster aminotransferase Fusarium fujikuroi
AHC70518 fumonisin biosynthetic alpha oxoamine synthase partial Fusarium fujikuroi
KLO95337 Uncharacterized protein Y057 12757 Fusarium fujikuroi
KLP17895 Uncharacterized protein LW94 10761 Fusarium fujikuroi
AFQ32311 FUM8 Fusarium fujikuroi
RBA10280 hypothetical protein FPRO05 06216 Fusarium proliferatum
CVL12738 fumonisin cluster aminotransferase Fusarium proliferatum
AHC70519 fumonisin biosynthetic alpha oxoamine synthase partial Fusarium proliferatum
CAQ16827 oxamine synthase partial Fusarium proliferatum
CAQ16826 oxamine synthase partial Fusarium proliferatum
CAQ16829 oxamine synthase partial Fusarium proliferatum
CAQ16831 oxamine synthase partial Fusarium proliferatum
CAQ16828 oxamine synthase partial Fusarium proliferatum
CAQ16825 oxamine synthase partial Fusarium proliferatum
CZR47923 fumonisin cluster aminotransferase Fusarium proliferatum ET1
CAQ16824 oxamine synthase partial Fusarium proliferatum

FUM10

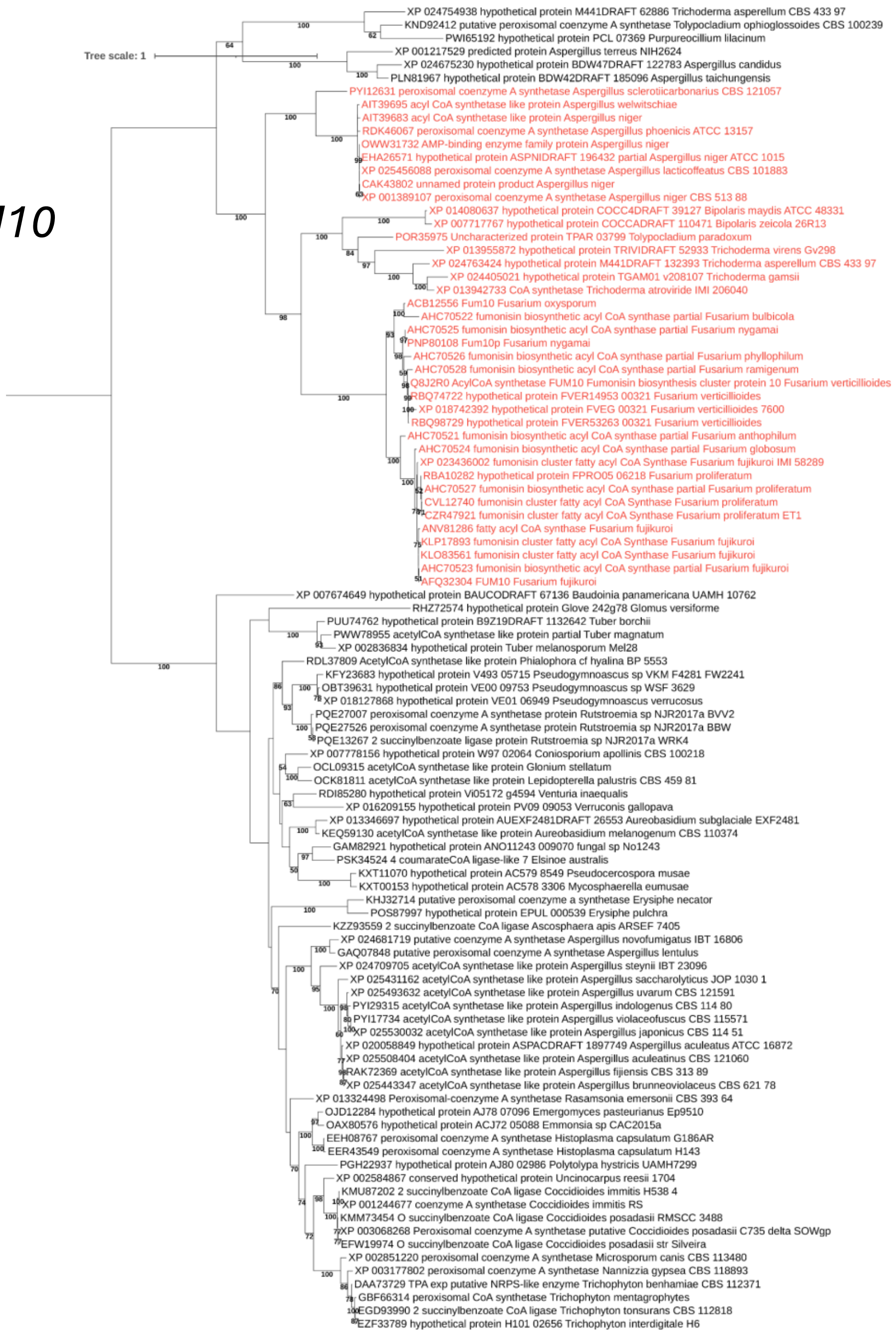
Tree scale: 1
XP 024754938 hypothetical protein M441DRAFT 62886 Trichoderma asperellum CBS 433 97
KND92412 putative peroxisomal coenzyme A synthetase Tolypocladium ophioglossoides CBS 100239
PWI65192 hypothetical protein PCL 07369 Purpureocillium lilacinum
XP 001217529 predicted protein Aspergillus terreus NIH2624
XP 024675230 hypothetical protein BDW47DRAFT 122783 Aspergillus candidus
PLN81967 hypothetical protein BDW42DRAFT 185096 Aspergillus taichungensis
PYI12631 peroxisomal coenzyme A synthetase Aspergillus sclerotiicarbonarius CBS 121057
AIT39695 acyl CoA synthetase like protein Aspergillus welwitschiae
AIT39683 acyl CoA synthetase like protein Aspergillus niger
RDK46067 peroxisomal coenzyme A synthetase Aspergillus phoenicis ATCC 13157
OWW31732 AMP-binding enzyme family protein Aspergillus niger
EHA26571 hypothetical protein ASPNIDRAFT 196432 partial Aspergillus niger ATCC 1015
XP 025456088 peroxisomal coenzyme A synthetase Aspergillus lacticoffeatus CBS 101883
CAK43802 unnamed protein product Aspergillus niger
XP 001389107 peroxisomal coenzyme A synthetase Aspergillus niger CBS 513 88
XP 014080637 hypothetical protein COCC4DRAFT 39127 Bipolaris maydis ATCC 48331
XP 007717767 hypothetical protein COCCADRAFT 110471 Bipolaris zeicola 26R13
POR35975 Uncharacterized protein TPAR 03799 Tolypocladium paradoxum
XP 013955872 hypothetical protein TRIVIDRAFT 52933 Trichoderma virens Gv298
XP 024763424 hypothetical protein M441DRAFT 132393 Trichoderma asperellum CBS 433 97
XP 024405021 hypothetical protein TGAM01 v208107 Trichoderma gamsii
XP 013942733 CoA synthetase Trichoderma atroviride IMI 206040
ACB12556 Fum10 Fusarium oxysporum
AHC70522 fumonisin biosynthetic acyl CoA synthase partial Fusarium bulbicola
AHC70525 fumonisin biosynthetic acyl CoA synthase partial Fusarium nygamai
PNP80108 Fum10p Fusarium nygamai
AHC70526 fumonisin biosynthetic acyl CoA synthase partial Fusarium phyllophilum
AHC70528 fumonisin biosynthetic acyl CoA synthase partial Fusarium ramigenum
Q8J2R0 AcylCoA synthetase FUM10 Fumonisin biosynthesis cluster protein 10 Fusarium verticillioides
RBQ74722 hypothetical protein FVER14953 00321 Fusarium verticillioides
XP 018742392 hypothetical protein FVEG 00321 Fusarium verticillioides 7600
RBQ98729 hypothetical protein FVER53263 00321 Fusarium verticillioides
AHC70521 fumonisin biosynthetic acyl CoA synthase partial Fusarium anthophilum
AHC70524 fumonisin biosynthetic acyl CoA synthase partial Fusarium globosum
XP 023436002 fumonisin cluster fatty acyl CoA Synthase Fusarium fujikuroi IMI 58289
RBA10282 hypothetical protein FPRO05 06218 Fusarium proliferatum
AHC70527 fumonisin biosynthetic acyl CoA synthase partial Fusarium proliferatum
CVL12740 fumonisin cluster fatty acyl CoA Synthase Fusarium proliferatum
CZR47921 fumonisin cluster fatty acyl CoA Synthase Fusarium proliferatum ET1
ANV81286 fatty acyl CoA synthase Fusarium fujikuroi
KLP17893 fumonisin cluster fatty acyl CoA Synthase Fusarium fujikuroi
KLO83561 fumonisin cluster fatty acyl CoA Synthase Fusarium fujikuroi
AHC70523 fumonisin biosynthetic acyl CoA synthase partial Fusarium fujikuroi
AFQ32304 FUM10 Fusarium fujikuroi
XP 007674649 hypothetical protein BAUCODRAFT 67136 Baudoinia panamericana UAMH 10762
RHZ72574 hypothetical protein Glove 242g78 Glomus versiforme
PUU74762 hypothetical protein B9Z19DRAFT 1132642 Tuber borchii
PWW78955 acetylCoA synthetase like protein partial Tuber magnatum
XP 002836834 hypothetical protein Tuber melanosporum Mel28
RDL37809 AcetylCoA synthetase like protein Phialophora cf hyalina BP 5553
KFY23683 hypothetical protein V493 05715 Pseudogymnoascus sp VKM F4281 FW2241
OBT39631 hypothetical protein VE00 09753 Pseudogymnoascus sp WSF 3629
XP 018127868 hypothetical protein VE01 06949 Pseudogymnoascus verrucosus
PQE27007 peroxisomal coenzyme A synthetase protein Rutstroemia sp NJR2017a BVV2
PQE27526 peroxisomal coenzyme A synthetase protein Rutstroemia sp NJR2017a BBW
PQE13267 2 succinylbenzoate ligase protein Rutstroemia sp NJR2017a WRK4
XP 007778156 hypothetical protein W97 02064 Coniosporium apollinis CBS 100218
OCL09315 acetylCoA synthetase like protein Glonium stellatum
OCK81811 acetylCoA synthetase like protein Lepidopterella palustris CBS 459 81
RDI85280 hypothetical protein Vi05172 g4594 Venturia inaequalis
XP 016209155 hypothetical protein PV09 09053 Verruconis gallopava
XP 013346697 hypothetical protein AUEXF2481DRAFT 26553 Aureobasidium subglaciale EXF2481
KEQ59130 acetylCoA synthetase like protein Aureobasidium melanogenum CBS 110374
GAM82921 hypothetical protein ANO11243 009070 fungal sp No1243
PSK34524 4 coumarateCoA ligase-like 7 Elsinoe australis
KXT11070 hypothetical protein AC579 8549 Pseudocercospora musae
KXT00153 hypothetical protein AC578 3306 Mycosphaerella eumusae
KHJ32714 putative peroxisomal coenzyme a synthetase Erysiphe necator
POS87997 hypothetical protein EPUL 000539 Erysiphe pulchra
KZZ93559 2 succinylbenzoate CoA ligase Ascosphaera apis ARSEF 7405
XP 024681719 putative coenzyme A synthetase Aspergillus novofumigatus IBT 16806
GAQ07848 putative peroxisomal coenzyme A synthetase Aspergillus lentulus
XP 024709705 acetylCoA synthetase like protein Aspergillus steynii IBT 23096
XP 025431162 acetylCoA synthetase like protein Aspergillus saccharolyticus JOP 1030 1
XP 025493632 acetylCoA synthetase like protein Aspergillus uvarum CBS 121591
PYI29315 acetylCoA synthetase like protein Aspergillus indologenus CBS 114 80
PYI17734 acetylCoA synthetase like protein Aspergillus violaceofuscus CBS 115571
XP 025530032 acetylCoA synthetase like protein Aspergillus japonicus CBS 114 51
XP 020058849 hypothetical protein ASPACDRAFT 1897749 Aspergillus aculeatus ATCC 16872
XP 025508404 acetylCoA synthetase like protein Aspergillus aculeatinus CBS 121060
RAK72369 acetylCoA synthetase like protein Aspergillus fijiensis CBS 313 89
XP 025443347 acetylCoA synthetase like protein Aspergillus brunneoviolaceus CBS 621 78
XP 013324498 Peroxisomal-coenzyme A synthetase Rasamsonia emersonii CBS 393 64
OJD12284 hypothetical protein AJ78 07096 Emergomyces pasteurianus Ep9510
OAX80576 hypothetical protein ACJ72 05088 Emmonsia sp CAC2015a
EEH08767 peroxisomal coenzyme A synthetase Histoplasma capsulatum G186AR
EER43549 peroxisomal coenzyme A synthetase Histoplasma capsulatum H143
PGH22937 hypothetical protein AJ80 02986 Polytolypa hystricis UAMH7299
XP 002584867 conserved hypothetical protein Uncinocarpus reesii 1704
KMU87202 2 succinylbenzoate CoA ligase Coccidioides immitis H538 4
XP 001244677 coenzyme A synthetase Coccidioides immitis RS
KMM73454 O succinylbenzoate CoA ligase Coccidioides posadasii RMSCC 3488
XP 003068268 Peroxisomal coenzyme A synthetase putative Coccidioides posadasii C735 delta SOWgp
EFW19974 O succinylbenzoate CoA ligase Coccidioides posadasii str Silveira
XP 002851220 peroxisomal coenzyme A synthetase Microsporum canis CBS 113480
XP 003177802 peroxisomal coenzyme A synthetase Nannizzia gypsea CBS 118893
DAA73729 TPA exp putative NRPS-like enzyme Trichophyton benhamiae CBS 112371
GBF66314 peroxisomal CoA synthetase Trichophyton mentagrophytes
EGD93990 2 succinylbenzoate CoA ligase Trichophyton tonsurans CBS 112818
EZF33789 hypothetical protein H101 02656 Trichophyton interdigitale H6

FUM11

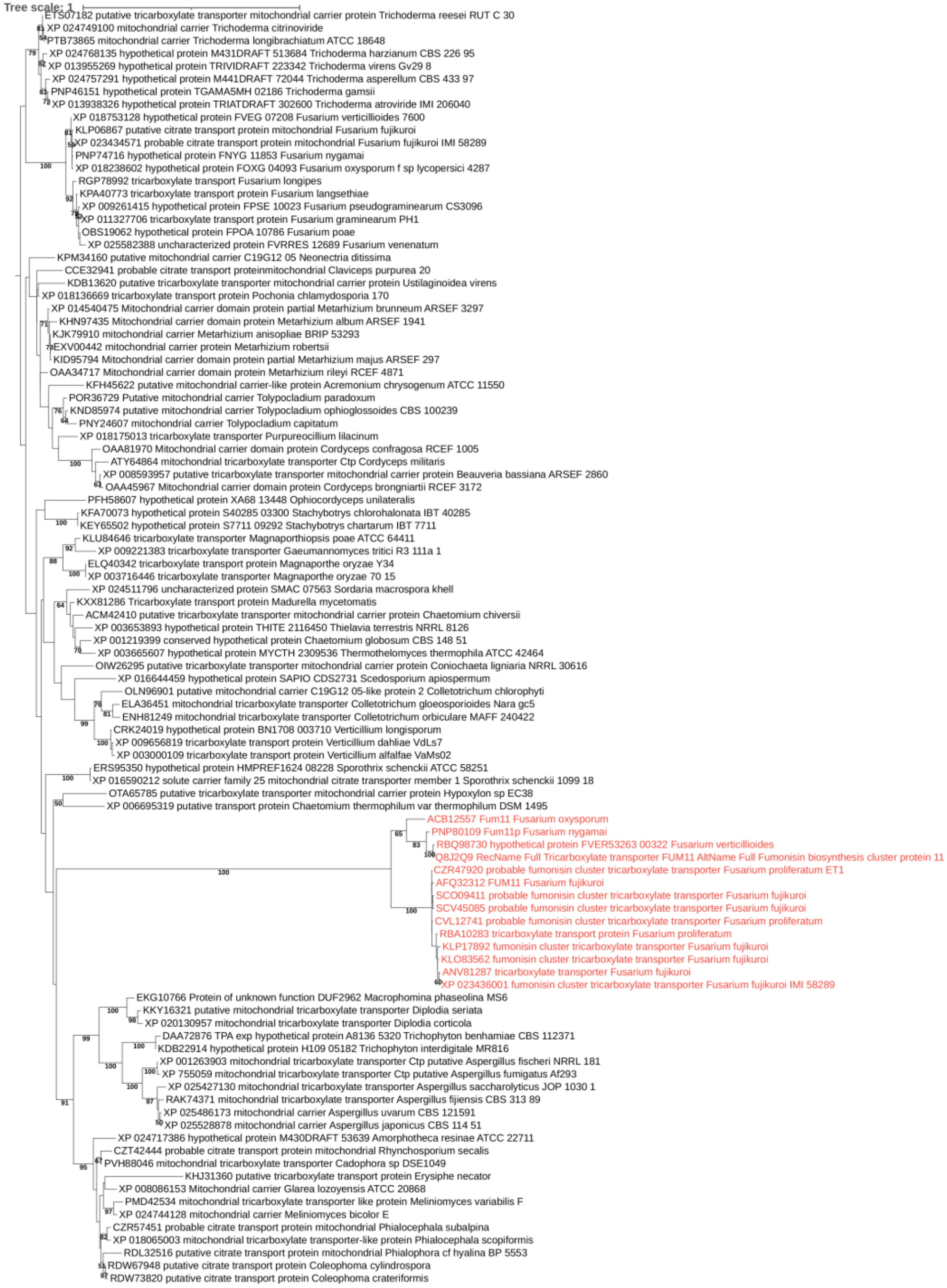

Tree scale: 1
ETS07182 putative tricarboxylate transporter mitochondrial carrier protein Trichoderma reesei RUT C 30
XP 024749100 mitochondrial carrier Trichoderma citrinoviride
PTB73865 mitochondrial carrier Trichoderma longibrachiatum ATCC 18648
XP 024768135 hypothetical protein M431DRAFT 513684 Trichoderma harzianum CBS 226 95
XP 013955269 hypothetical protein TRIVIDRAFT 223342 Trichoderma virens Gv29 8
XP 024757291 hypothetical protein M441DRAFT 72044 Trichoderma asperellum CBS 433 97
PNP46151 hypothetical protein TGAMA5MH 02186 Trichoderma gamsii
XP 013938326 hypothetical protein TRIATDRAFT 302600 Trichoderma atroviride IMI 206040
XP 018753128 hypothetical protein FVEG 07208 Fusarium verticillioides 7600
KLP06867 putative citrate transport protein mitochondrial Fusarium fujikuroi
XP 023434571 probable citrate transport protein mitochondrial Fusarium fujikuroi IMI 58289
PNP74716 hypothetical protein FNYG 11853 Fusarium nygamai
XP 018238602 hypothetical protein FOXG 04093 Fusarium oxysporum f sp lycopersici 4287
RGP78992 tricarboxylate transport Fusarium longipes
KPA40773 tricarboxylate transport protein Fusarium langsethiae
XP 009261415 hypothetical protein FPSE 10023 Fusarium pseudograminearum CS3096
XP 011327706 tricarboxylate transport protein Fusarium graminearum PH1
OBS19062 hypothetical protein FPOA 10786 Fusarium poae
XP 025582388 uncharacterized protein FVRRES 12689 Fusarium venenatum
KPM34160 putative mitochondrial carrier C19G12 05 Neonectria ditissima
CCE32941 probable citrate transport proteinmitochondrial Claviceps purpurea 20
KDB13620 putative tricarboxylate transporter mitochondrial carrier protein Ustilaginoidea virens
XP 018136669 tricarboxylate transport protein Pochonia chlamydosporia 170
XP 014540475 Mitochondrial carrier domain protein partial Metarhizium brunneum ARSEF 3297
KHN97435 Mitochondrial carrier domain protein Metarhizium album ARSEF 1941
KJK79910 mitochondrial carrier Metarhizium anisopliae BRIP 53293
EXV00442 mitochondrial carrier protein Metarhizium robertsii
KID95794 Mitochondrial carrier domain protein partial Metarhizium majus ARSEF 297
OAA34717 Mitochondrial carrier domain protein Metarhizium rileyi RCEF 4871
KFH45622 putative mitochondrial carrier-like protein Acremonium chrysogenum ATCC 11550
POR36729 Putative mitochondrial carrier Tolypocladium paradoxum
KND85974 putative mitochondrial carrier Tolypocladium ophioglossoides CBS 100239
PNY24607 mitochondrial carrier Tolypocladium capitatum
XP 018175013 tricarboxylate transporter Purpureocillium lilacinum
OAA81970 Mitochondrial carrier domain protein Cordyceps confragosa RCEF 1005
ATY64864 mitochondrial tricarboxylate transporter Ctp Cordyceps militaris
XP 008593957 putative tricarboxylate transporter mitochondrial carrier protein Beauveria bassiana ARSEF 2860
OAA45967 Mitochondrial carrier domain protein Cordyceps brongniartii RCEF 3172
PFH58607 hypothetical protein XA68 13448 Ophiocordyceps unilateralis
KFA70073 hypothetical protein S40285 03300 Stachybotrys chlorohalonata IBT 40285
KEY65502 hypothetical protein S7711 09292 Stachybotrys chartarum IBT 7711
KLU84646 tricarboxylate transporter Magnaporthiopsis poae ATCC 64411
XP 009221383 tricarboxylate transporter Gaeumannomyces tritici R3 111a 1
ELQ40342 tricarboxylate transport protein Magnaporthe oryzae Y34
XP 003716446 tricarboxylate transporter Magnaporthe oryzae 70 15
XP 024511796 uncharacterized protein SMAC 07563 Sordaria macrospora khell
KXX81286 Tricarboxylate transport protein Madurella mycetomatis
ACM42410 putative tricarboxylate transporter mitochondrial carrier protein Chaetomium chiversii
XP 003653893 hypothetical protein THITE 2116450 Thielavia terrestris NRRL 8126
XP 001219399 conserved hypothetical protein Chaetomium globosum CBS 148 51
XP 003665607 hypothetical protein MYCTH 2309536 Thermothelomyces thermophila ATCC 42464
OIW26295 putative tricarboxylate transporter mitochondrial carrier protein Coniochaeta ligniaria NRRL 30616
XP 016644459 hypothetical protein SAPIO CDS2731 Scedosporium apiospermum
OLN96901 putative mitochondrial carrier C19G12 05-like protein 2 Colletotrichum chlorophyti
ELA36451 mitochondrial tricarboxylate transporter Colletotrichum gloeosporioides Nara gc5
ENH81249 mitochondrial tricarboxylate transporter Colletotrichum orbiculare MAFF 240422
CRK24019 hypothetical protein BN1708 003710 Verticillium longisporum
XP 009656819 tricarboxylate transport protein Verticillium dahliae VdLs7
XP 003000109 tricarboxylate transport protein Verticillium alfalfae VaMs02
ERS95350 hypothetical protein HMPREF1624 08228 Sporothrix schenckii ATCC 58251
XP 016590212 solute carrier family 25 mitochondrial citrate transporter member 1 Sporothrix schenckii 1099 18
OTA65785 putative tricarboxylate transporter mitochondrial carrier protein Hypoxylon sp EC38
XP 006695319 putative transport protein Chaetomium thermophilum var thermophilum DSM 1495
ACB12557 Fum11 Fusarium oxysporum
PNP80109 Fum11p Fusarium nygamai
RBQ98730 hypothetical protein FVER53263 00322 Fusarium verticillioides
Q8J2Q9 RecName Full Tricarboxylate transporter FUM11 AltName Full Fumonisin biosynthesis cluster protein 11
CZR47920 probable fumonisin cluster tricarboxylate transporter Fusarium proliferatum ET1
AFQ32312 FUM11 Fusarium fujikuroi
SCO09411 probable fumonisin cluster tricarboxylate transporter Fusarium fujikuroi
SCV45085 probable fumonisin cluster tricarboxylate transporter Fusarium fujikuroi
CVL12741 probable fumonisin cluster tricarboxylate transporter Fusarium proliferatum
RBA10283 tricarboxylate transport protein Fusarium proliferatum
KLP17892 fumonisin cluster tricarboxylate transporter Fusarium fujikuroi
KLO83562 fumonisin cluster tricarboxylate transporter Fusarium fujikuroi
ANV81287 tricarboxylate transporter Fusarium fujikuroi
XP 023436001 fumonisin cluster tricarboxylate transporter Fusarium fujikuroi IMI 58289
EKG10766 Protein of unknown function DUF2962 Macrophomina phaseolina MS6
KKY16321 putative mitochondrial tricarboxylate transporter Diplodia seriata
XP 020130957 mitochondrial tricarboxylate transporter Diplodia corticola
DAA72876 TPA exp hypothetical protein A8136 5320 Trichophyton benhamiae CBS 112371
KDB22914 hypothetical protein H109 05182 Trichophyton interdigitale MR816
XP 001263903 mitochondrial tricarboxylate transporter Ctp putative Aspergillus fischeri NRRL 181
XP 755059 mitochondrial tricarboxylate transporter Ctp putative Aspergillus fumigatus Af293
XP 025427130 mitochondrial tricarboxylate transporter Aspergillus saccharolyticus JOP 1030 1
RAK74371 mitochondrial tricarboxylate transporter Aspergillus fijiensis CBS 313 89
XP 025486173 mitochondrial carrier Aspergillus uvarum CBS 121591
XP 025528878 mitochondrial carrier Aspergillus japonicus CBS 114 51
XP 024717386 hypothetical protein M430DRAFT 53639 Amorphotheca resinae ATCC 22711
CZT42444 probable citrate transport protein mitochondrial Rhynchosporium secalis
PVH88046 mitochondrial tricarboxylate transporter Cadophora sp DSE1049
KHJ31360 putative tricarboxylate transport protein Erysiphe necator
XP 008086153 Mitochondrial carrier Glarea lozoyensis ATCC 20868
PMD42534 mitochondrial tricarboxylate transporter like protein Meliniomyces variabilis F
XP 024744128 mitochondrial carrier Meliniomyces bicolor E
CZR57451 probable citrate transport protein mitochondrial Phialocephala subalpina
XP 018065003 mitochondrial tricarboxylate transporter-like protein Phialocephala scopiformis
RDL32516 putative citrate transport protein mitochondrial Phialophora cf hyalina BP 5553
RDW67948 putative citrate transport protein Coleophoma cylindrospora
RDW73820 putative citrate transport protein Coleophoma crateriformis

FUM13

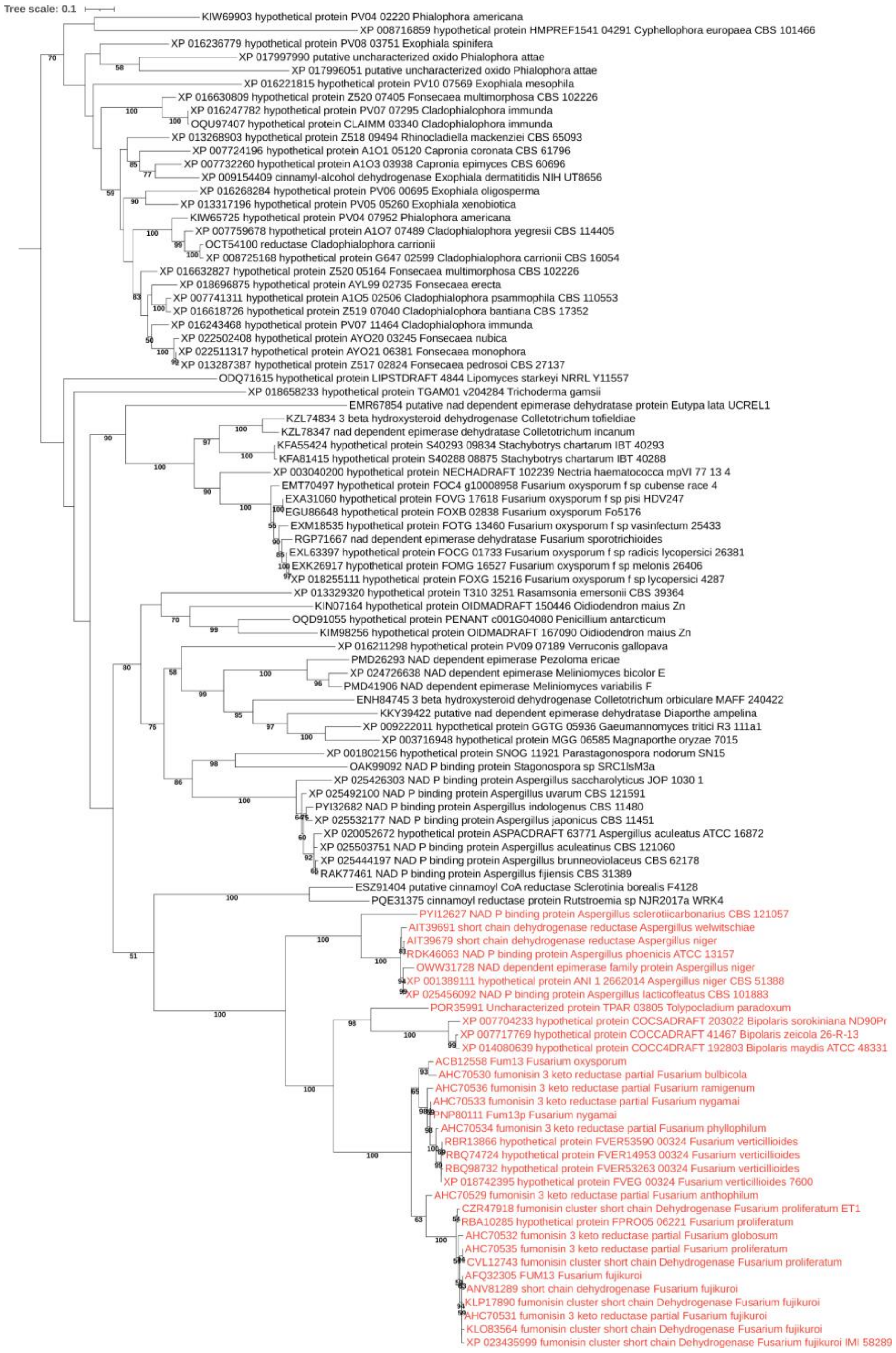

Tree scale: 0.1
KIW69903 hypothetical protein PV04 02220 Phialophora americana
XP 008716859 hypothetical protein HMPREF1541 04291 Cyphellophora europaea CBS 101466
XP 016236779 hypothetical protein PV08 03751 Exophiala spinifera
XP 017997990 putative uncharacterized oxido Phialophora attae
XP 017996051 putative uncharacterized oxido Phialophora attae
XP 016221815 hypothetical protein PV10 07569 Exophiala mesophila
XP 016630809 hypothetical protein Z520 07405 Fonsecaea multimorphosa CBS 102226
XP 016247782 hypothetical protein PV07 07295 Cladophialophora immunda
OQU97407 hypothetical protein CLAIMM 03340 Cladophialophora immunda
XP 013268903 hypothetical protein Z518 09494 Rhinocladiella mackenziei CBS 65093
XP 007724196 hypothetical protein A1O1 05120 Capronia coronata CBS 61796
XP 007732260 hypothetical protein A1O3 03938 Capronia epimyces CBS 60696
XP 009154409 cinnamyl-alcohol dehydrogenase Exophiala dermatitidis NIH UT8656
XP 016268284 hypothetical protein PV06 00695 Exophiala oligosperma
XP 013317196 hypothetical protein PV05 05260 Exophiala xenobiotica
KIW65725 hypothetical protein PV04 07952 Phialophora americana
XP 007759678 hypothetical protein A1O7 07489 Cladophialophora yegresii CBS 114405
OCT54100 reductase Cladophialophora carrionii
XP 008725168 hypothetical protein G647 02599 Cladophialophora carrionii CBS 16054
XP 016632827 hypothetical protein Z520 05164 Fonsecaea multimorphosa CBS 102226
XP 018696875 hypothetical protein AYL99 02735 Fonsecaea erecta
XP 007741311 hypothetical protein A1O5 02506 Cladophialophora psammophila CBS 110553
XP 016618726 hypothetical protein Z519 07040 Cladophialophora bantiana CBS 17352
XP 016243468 hypothetical protein PV07 11464 Cladophialophora immunda
XP 022502408 hypothetical protein AYO20 03245 Fonsecaea nubica
XP 022511317 hypothetical protein AYO21 06381 Fonsecaea monophora
XP 013287387 hypothetical protein Z517 02824 Fonsecaea pedrosoi CBS 27137
ODQ71615 hypothetical protein LIPSTDRAFT 4844 Lipomyces starkeyi NRRL Y11557
XP 018658233 hypothetical protein TGAM01 v204284 Trichoderma gamsii
EMR67854 putative nad dependent epimerase dehydratase protein Eutypa lata UCREL1
KZL74834 3 beta hydroxysteroid dehydrogenase Colletotrichum tofieldiae
KZL78347 nad dependent epimerase dehydratase Colletotrichum incanum
KFA55424 hypothetical protein S40293 09834 Stachybotrys chartarum IBT 40293
KFA81415 hypothetical protein S40288 08875 Stachybotrys chartarum IBT 40288
XP 003040200 hypothetical protein NECHADRAFT 102239 Nectria haematococca mpVI 77 13 4
EMT70497 hypothetical protein FOC4 g10008958 Fusarium oxysporum f sp cubense race 4
EXA31060 hypothetical protein FOVG 17618 Fusarium oxysporum f sp pisi HDV247
EGU86648 hypothetical protein FOXB 02838 Fusarium oxysporum Fo5176
EXM18535 hypothetical protein FOTG 13460 Fusarium oxysporum f sp vasinfectum 25433
RGP71667 nad dependent epimerase dehydratase Fusarium sporotrichioides
EXL63397 hypothetical protein FOCG 01733 Fusarium oxysporum f sp radicis lycopersici 26381
EXK26917 hypothetical protein FOMG 16527 Fusarium oxysporum f sp melonis 26406
XP 018255111 hypothetical protein FOXG 15216 Fusarium oxysporum f sp lycopersici 4287
XP 013329320 hypothetical protein T310 3251 Rasamsonia emersonii CBS 39364
KIN07164 hypothetical protein OIDMADRAFT 150446 Oidiodendron maius Zn
OQD91055 hypothetical protein PENANT c001G04080 Penicillium antarcticum
KIM98256 hypothetical protein OIDMADRAFT 167090 Oidiodendron maius Zn
XP 016211298 hypothetical protein PV09 07189 Verruconis gallopava
PMD26293 NAD dependent epimerase Pezoloma ericae
XP 024726638 NAD dependent epimerase Meliniomyces bicolor E
PMD41906 NAD dependent epimerase Meliniomyces variabilis F
ENH84745 3 beta hydroxysteroid dehydrogenase Colletotrichum orbiculare MAFF 240422
KKY39422 putative nad dependent epimerase dehydratase Diaporthe ampelina
XP 009222011 hypothetical protein GGTG 05936 Gaeumannomyces tritici R3 111a1
XP 003716948 hypothetical protein MGG 06585 Magnaporthe oryzae 7015
XP 001802156 hypothetical protein SNOG 11921 Parastagonospora nodorum SN15
OAK99092 NAD P binding protein Stagonospora sp SRC1lsM3a
XP 025426303 NAD P binding protein Aspergillus saccharolyticus JOP 1030 1
XP 025492100 NAD P binding protein Aspergillus uvarum CBS 121591
PYI32682 NAD P binding protein Aspergillus indologenus CBS 11480
XP 025532177 NAD P binding protein Aspergillus japonicus CBS 11451
XP 020052672 hypothetical protein ASPACDRAFT 63771 Aspergillus aculeatus ATCC 16872
XP 025503751 NAD P binding protein Aspergillus aculeatinus CBS 121060
XP 025444197 NAD P binding protein Aspergillus brunneoviolaceus CBS 62178
RAK77461 NAD P binding protein Aspergillus fijiensis CBS 31389
ESZ91404 putative cinnamoyl CoA reductase Sclerotinia borealis F4128
PQE31375 cinnamoyl reductase protein Rutstroemia sp NJR2017a WRK4
PYI12627 NAD P binding protein Aspergillus sclerotiicarbonarius CBS 121057
AIT39691 short chain dehydrogenase reductase Aspergillus welwitschiae
AIT39679 short chain dehydrogenase reductase Aspergillus niger
RDK46063 NAD P binding protein Aspergillus phoenicis ATCC 13157
OWW31728 NAD dependent epimerase family protein Aspergillus niger
XP 001389111 hypothetical protein ANI 1 2662014 Aspergillus niger CBS 51388
XP 025456092 NAD P binding protein Aspergillus lacticoffeatus CBS 101883
POR35991 Uncharacterized protein TPAR 03805 Tolypocladium paradoxum
XP 007704233 hypothetical protein COCSADRAFT 203022 Bipolaris sorokiniana ND90Pr
XP 007717769 hypothetical protein COCCADRAFT 41467 Bipolaris zeicola 26-R-13
XP 014080639 hypothetical protein COCC4DRAFT 192803 Bipolaris maydis ATCC 48331
ACB12558 Fum13 Fusarium oxysporum
AHC70530 fumonisin 3 keto reductase partial Fusarium bulbicola
AHC70536 fumonisin 3 keto reductase partial Fusarium ramigenum
AHC70533 fumonisin 3 keto reductase partial Fusarium nygamai
PNP80111 Fum13p Fusarium nygamai
AHC70534 fumonisin 3 keto reductase partial Fusarium phyllophilum
RBR13866 hypothetical protein FVER53590 00324 Fusarium verticillioides
RBQ74724 hypothetical protein FVER14953 00324 Fusarium verticillioides
RBQ98732 hypothetical protein FVER53263 00324 Fusarium verticillioides
XP 018742395 hypothetical protein FVEG 00324 Fusarium verticillioides 7600
AHC70529 fumonisin 3 keto reductase partial Fusarium anthophilum
CZR47918 fumonisin cluster short chain Dehydrogenase Fusarium proliferatum ET1
RBA10285 hypothetical protein FPRO05 06221 Fusarium proliferatum
AHC70532 fumonisin 3 keto reductase partial Fusarium globosum
AHC70535 fumonisin 3 keto reductase partial Fusarium proliferatum
CVL12743 fumonisin cluster short chain Dehydrogenase Fusarium proliferatum
AFQ32305 FUM13 Fusarium fujikuroi
ANV81289 short chain dehydrogenase Fusarium fujikuroi
KLP17890 fumonisin cluster short chain Dehydrogenase Fusarium fujikuroi
AHC70531 fumonisin 3 keto reductase partial Fusarium fujikuroi
KLO83564 fumonisin cluster short chain Dehydrogenase Fusarium fujikuroi
XP 023435999 fumonisin cluster short chain Dehydrogenase Fusarium fujikuroi IMI 58289

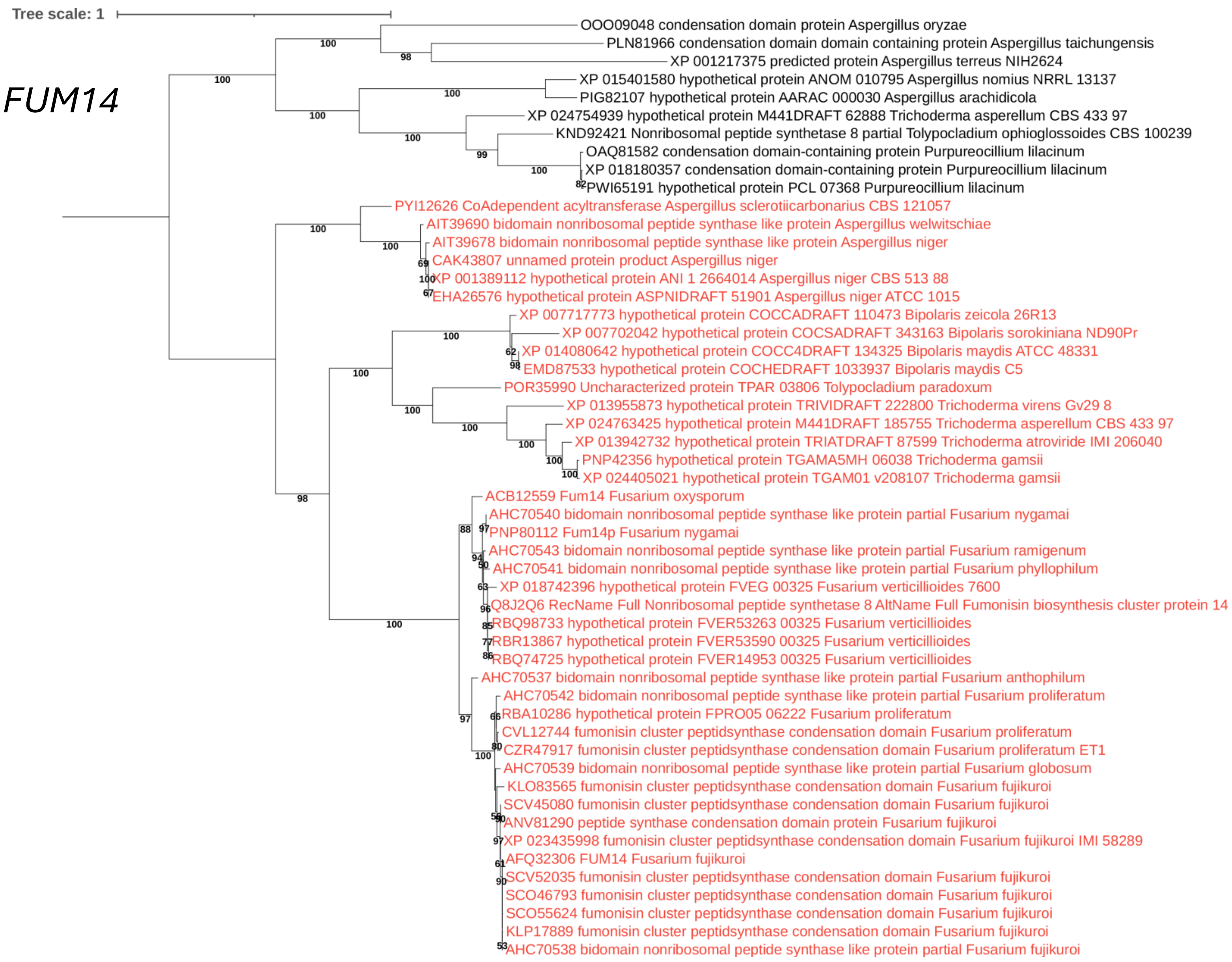
Tree scale: 1
FUM14
OOO09048 condensation domain protein Aspergillus oryzae
PLN81966 condensation domain domain containing protein Aspergillus taichungensis
XP 001217375 predicted protein Aspergillus terreus NIH2624
XP 015401580 hypothetical protein ANOM 010795 Aspergillus nomius NRRL 13137
PIG82107 hypothetical protein AARAC 000030 Aspergillus arachidicola
XP 024754939 hypothetical protein M441DRAFT 62888 Trichoderma asperellum CBS 433 97
KND92421 Nonribosomal peptide synthetase 8 partial Tolypocladium ophioglossoides CBS 100239
OAQ81582 condensation domain-containing protein Purpureocillium lilacinum
XP 018180357 condensation domain-containing protein Purpureocillium lilacinum
PWI65191 hypothetical protein PCL 07368 Purpureocillium lilacinum
PYI12626 CoAdependent acyltransferase Aspergillus sclerotiicarbonarius CBS 121057
AIT39690 bidomain nonribosomal peptide synthase like protein Aspergillus welwitschiae
AIT39678 bidomain nonribosomal peptide synthase like protein Aspergillus niger
CAK43807 unnamed protein product Aspergillus niger
XP 001389112 hypothetical protein ANI 1 2664014 Aspergillus niger CBS 513 88
EHA26576 hypothetical protein ASPNIDRAFT 51901 Aspergillus niger ATCC 1015
XP 007717773 hypothetical protein COCCADRAFT 110473 Bipolaris zeicola 26R13
XP 007702042 hypothetical protein COCSADRAFT 343163 Bipolaris sorokiniana ND90Pr
XP 014080642 hypothetical protein COCC4DRAFT 134325 Bipolaris maydis ATCC 48331
EMD87533 hypothetical protein COCHEDRAFT 1033937 Bipolaris maydis C5
POR35990 Uncharacterized protein TPAR 03806 Tolypocladium paradoxum
XP 013955873 hypothetical protein TRIVIDRAFT 222800 Trichoderma virens Gv29 8
XP 024763425 hypothetical protein M441DRAFT 185755 Trichoderma asperellum CBS 433 97
XP 013942732 hypothetical protein TRIATDRAFT 87599 Trichoderma atroviride IMI 206040
PNP42356 hypothetical protein TGAMA5MH 06038 Trichoderma gamsii
XP 024405021 hypothetical protein TGAM01 v208107 Trichoderma gamsii
ACB12559 Fum14 Fusarium oxysporum
AHC70540 bidomain nonribosomal peptide synthase like protein partial Fusarium nygamai
PNP80112 Fum14p Fusarium nygamai
AHC70543 bidomain nonribosomal peptide synthase like protein partial Fusarium ramigenum
AHC70541 bidomain nonribosomal peptide synthase like protein partial Fusarium phyllophilum
XP 018742396 hypothetical protein FVEG 00325 Fusarium verticillioides 7600
Q8J2Q6 RecName Full Nonribosomal peptide synthetase 8 AltName Full Fumonisin biosynthesis cluster protein 14
RBQ98733 hypothetical protein FVER53263 00325 Fusarium verticillioides
RBR13867 hypothetical protein FVER53590 00325 Fusarium verticillioides
RBQ74725 hypothetical protein FVER14953 00325 Fusarium verticillioides
AHC70537 bidomain nonribosomal peptide synthase like protein partial Fusarium anthophilum
AHC70542 bidomain nonribosomal peptide synthase like protein partial Fusarium proliferatum
RBA10286 hypothetical protein FPRO05 06222 Fusarium proliferatum
CVL12744 fumonisin cluster peptidsynthase condensation domain Fusarium proliferatum
CZR47917 fumonisin cluster peptidsynthase condensation domain Fusarium proliferatum ET1
AHC70539 bidomain nonribosomal peptide synthase like protein partial Fusarium globosum
KLO83565 fumonisin cluster peptidsynthase condensation domain Fusarium fujikuroi
SCV45080 fumonisin cluster peptidsynthase condensation domain Fusarium fujikuroi
ANV81290 peptide synthase condensation domain protein Fusarium fujikuroi
XP 023435998 fumonisin cluster peptidsynthase condensation domain Fusarium fujikuroi IMI 58289
AFQ32306 FUM14 Fusarium fujikuroi
SCV52035 fumonisin cluster peptidsynthase condensation domain Fusarium fujikuroi
SCO46793 fumonisin cluster peptidsynthase condensation domain Fusarium fujikuroi
SCO55624 fumonisin cluster peptidsynthase condensation domain Fusarium fujikuroi
KLP17889 fumonisin cluster peptidsynthase condensation domain Fusarium fujikuroi
AHC70538 bidomain nonribosomal peptide synthase like protein partial Fusarium fujikuroi

FUM15

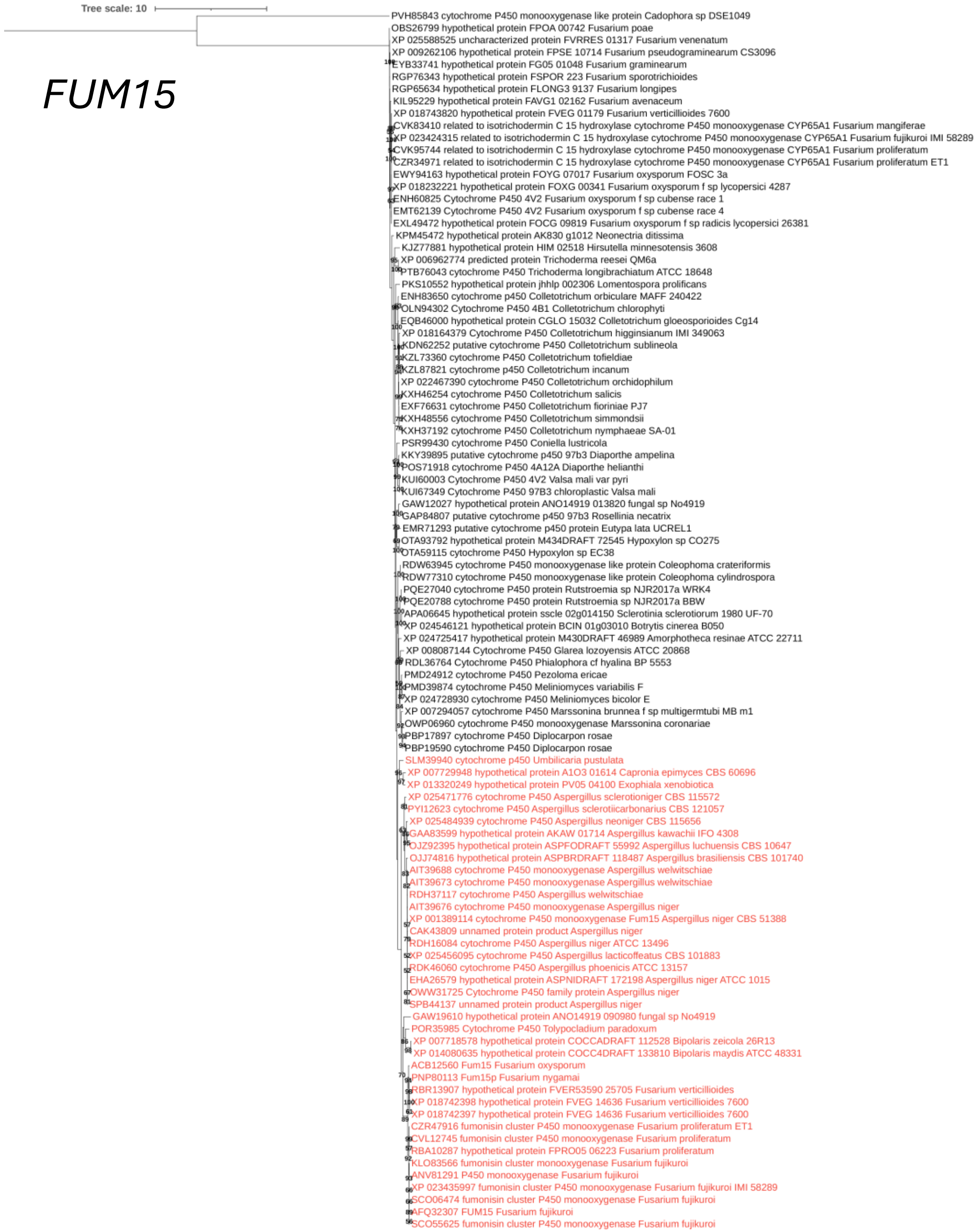

Tree scale: 10
PVH85843 cytochrome P450 monooxygenase like protein Cadophora sp DSE1049
OBS26799 hypothetical protein FPOA 00742 Fusarium poae
XP 025588525 uncharacterized protein FVRRES 01317 Fusarium venenatum
XP 009262106 hypothetical protein FPSE 10714 Fusarium pseudograminearum CS3096
EYB33741 hypothetical protein FG05 01048 Fusarium graminearum
RGP76343 hypothetical protein FSPOR 223 Fusarium sporotrichioides
RGP65634 hypothetical protein FLONG3 9137 Fusarium longipes
KIL95229 hypothetical protein FAVG1 02162 Fusarium avenaceum
XP 018743820 hypothetical protein FVEG 01179 Fusarium verticillioides 7600
CVK83410 related to isotrichodermin C 15 hydroxylase cytochrome P450 monooxygenase CYP65A1 Fusarium mangiferae
XP 023424315 related to isotrichodermin C 15 hydroxylase cytochrome P450 monooxygenase CYP65A1 Fusarium fujikuroi IMI 58289
CVK95744 related to isotrichodermin C 15 hydroxylase cytochrome P450 monooxygenase CYP65A1 Fusarium proliferatum
CZR34971 related to isotrichodermin C 15 hydroxylase cytochrome P450 monooxygenase CYP65A1 Fusarium proliferatum ET1
EWY94163 hypothetical protein FOYG 07017 Fusarium oxysporum FOSC 3a
XP 018232221 hypothetical protein FOXG 00341 Fusarium oxysporum f sp lycopersici 4287
ENH60825 Cytochrome P450 4V2 Fusarium oxysporum f sp cubense race 1
EMT62139 Cytochrome P450 4V2 Fusarium oxysporum f sp cubense race 4
EXL49472 hypothetical protein FOCG 09819 Fusarium oxysporum f sp radicis lycopersici 26381
KPM45472 hypothetical protein AK830 g1012 Neonectria ditissima
KJZ77881 hypothetical protein HIM 02518 Hirsutella minnesotensis 3608
XP 006962774 predicted protein Trichoderma reesei QM6a
PTB76043 cytochrome P450 Trichoderma longibrachiatum ATCC 18648
PKS10552 hypothetical protein jhhlp 002306 Lomentospora prolificans
ENH83650 cytochrome p450 Colletotrichum orbiculare MAFF 240422
OLN94302 Cytochrome P450 4B1 Colletotrichum chlorophyti
EQB46000 hypothetical protein CGLO 15032 Colletotrichum gloeosporioides Cg14
XP 018164379 Cytochrome P450 Colletotrichum higginsianum IMI 349063
KDN62252 putative cytochrome P450 Colletotrichum sublineola
KZL73360 cytochrome P450 Colletotrichum tofieldiae
KZL87821 cytochrome p450 Colletotrichum incanum
XP 022467390 cytochrome P450 Colletotrichum orchidophilum
KXH46254 cytochrome P450 Colletotrichum salicis
EXF76631 cytochrome P450 Colletotrichum fioriniae PJ7
KXH48556 cytochrome P450 Colletotrichum simmondsii
KXH37192 cytochrome P450 Colletotrichum nymphaeae SA-01
PSR99430 cytochrome P450 Coniella lustricola
KKY39895 putative cytochrome p450 97b3 Diaporthe ampelina
POS71918 cytochrome P450 4A12A Diaporthe helianthi
KUI60003 Cytochrome P450 4V2 Valsa mali var pyri
KUI67349 Cytochrome P450 97B3 chloroplastic Valsa mali
GAW12027 hypothetical protein ANO14919 013820 fungal sp No4919
GAP84807 putative cytochrome p450 97b3 Rosellinia necatrix
EMR71293 putative cytochrome p450 protein Eutypa lata UCREL1
OTA93792 hypothetical protein M434DRAFT 72545 Hypoxylon sp CO275
OTA59115 cytochrome P450 Hypoxylon sp EC38
RDW63945 cytochrome P450 monooxygenase like protein Coleophoma crateriformis
RDW77310 cytochrome P450 monooxygenase like protein Coleophoma cylindrospora
PQE27040 cytochrome P450 protein Rutstroemia sp NJR2017a WRK4
PQE20788 cytochrome P450 protein Rutstroemia sp NJR2017a BBW
APA06645 hypothetical protein sscle 02g014150 Sclerotinia sclerotiorum 1980 UF-70
XP 024546121 hypothetical protein BCIN 01g03010 Botrytis cinerea B050
XP 024725417 hypothetical protein M430DRAFT 46989 Amorphotheca resinae ATCC 22711
XP 008087144 Cytochrome P450 Glarea lozoyensis ATCC 20868
RDL36764 Cytochrome P450 Phialophora cf hyalina BP 5553
PMD24912 cytochrome P450 Pezoloma ericae
PMD39874 cytochrome P450 Meliniomyces variabilis F
XP 024728930 cytochrome P450 Meliniomyces bicolor E
XP 007294057 cytochrome P450 Marssonina brunnea f sp multigermtubi MB m1
OWP06960 cytochrome P450 monooxygenase Marssonina coronariae
PBP17897 cytochrome P450 Diplocarpon rosae
PBP19590 cytochrome P450 Diplocarpon rosae
SLM39940 cytochrome p450 Umbilicaria pustulata
XP 007729948 hypothetical protein A1O3 01614 Capronia epimyces CBS 60696
XP 013320249 hypothetical protein PV05 04100 Exophiala xenobiotica
XP 025471776 cytochrome P450 Aspergillus sclerotioniger CBS 115572
PYI12623 cytochrome P450 Aspergillus sclerotiicarbonarius CBS 121057
XP 025484939 cytochrome P450 Aspergillus neoniger CBS 115656
GAA83599 hypothetical protein AKAW 01714 Aspergillus kawachii IFO 4308
OJZ92395 hypothetical protein ASPFODRAFT 55992 Aspergillus luchuensis CBS 10647
OJJ74816 hypothetical protein ASPBRDRAFT 118487 Aspergillus brasiliensis CBS 101740
AIT39688 cytochrome P450 monooxygenase Aspergillus welwitschiae
AIT39673 cytochrome P450 monooxygenase Aspergillus welwitschiae
RDH37117 cytochrome P450 Aspergillus welwitschiae
AIT39676 cytochrome P450 monooxygenase Aspergillus niger
XP 001389114 cytochrome P450 monooxygenase Fum15 Aspergillus niger CBS 51388
CAK43809 unnamed protein product Aspergillus niger
RDH16084 cytochrome P450 Aspergillus niger ATCC 13496
XP 025456095 cytochrome P450 Aspergillus lacticoffeatus CBS 101883
RDK46060 cytochrome P450 Aspergillus phoenicis ATCC 13157
EHA26579 hypothetical protein ASPNIDRAFT 172198 Aspergillus niger ATCC 1015
OWW31725 Cytochrome P450 family protein Aspergillus niger
SPB44137 unnamed protein product Aspergillus niger
GAW19610 hypothetical protein ANO14919 090980 fungal sp No4919
POR35985 Cytochrome P450 Tolypocladium paradoxum
XP 007718578 hypothetical protein COCCADRAFT 112528 Bipolaris zeicola 26R13
XP 014080635 hypothetical protein COCC4DRAFT 133810 Bipolaris maydis ATCC 48331
ACB12560 Fum15 Fusarium oxysporum
PNP80113 Fum15p Fusarium nygamai
RBR13907 hypothetical protein FVER53590 25705 Fusarium verticillioides
XP 018742398 hypothetical protein FVEG 14636 Fusarium verticillioides 7600
XP 018742397 hypothetical protein FVEG 14636 Fusarium verticillioides 7600
CZR47916 fumonisin cluster P450 monooxygenase Fusarium proliferatum ET1
CVL12745 fumonisin cluster P450 monooxygenase Fusarium proliferatum
RBA10287 hypothetical protein FPRO05 06223 Fusarium proliferatum
KLO83566 fumonisin cluster monooxygenase Fusarium fujikuroi
ANV81291 P450 monooxygenase Fusarium fujikuroi
XP 023435997 fumonisin cluster P450 monooxygenase Fusarium fujikuroi IMI 58289
SCO06474 fumonisin cluster P450 monooxygenase Fusarium fujikuroi
AFQ32307 FUM15 Fusarium fujikuroi
SCO55625 fumonisin cluster P450 monooxygenase Fusarium fujikuroi

FUM16

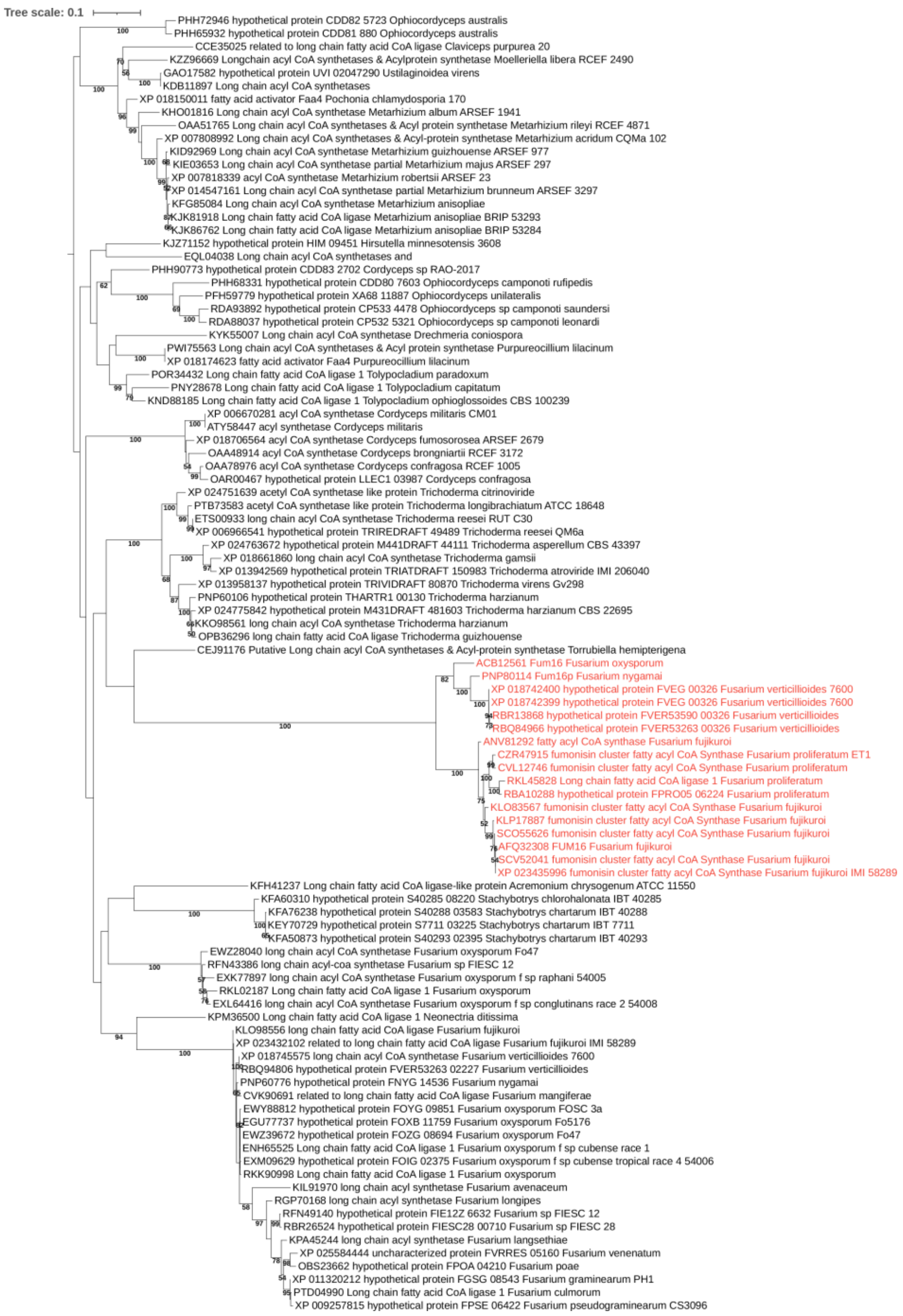
Tree scale: 0.1
PHH72946 hypothetical protein CDD82 5723 Ophiocordyceps australis
PHH65932 hypothetical protein CDD81 880 Ophiocordyceps australis
CCE35025 related to long chain fatty acid CoA ligase Claviceps purpurea 20
KZZ96669 Longchain acyl CoA synthetases & Acylprotein synthetase Moelleriella libera RCEF 2490
GAO17582 hypothetical protein UVI 02047290 Ustilaginoidea virens
KDB11897 Long chain acyl CoA synthetases
XP 018150011 fatty acid activator Faa4 Pochonia chlamydosporia 170
KHO01816 Long chain acyl CoA synthetase Metarhizium album ARSEF 1941
OAA51765 Long chain acyl CoA synthetases & Acyl protein synthetase Metarhizium rileyi RCEF 4871
XP 007808992 Long chain acyl CoA synthetases & Acyl-protein synthetase Metarhizium acridum CQMa 102
KID92969 Long chain acyl CoA synthetase Metarhizium guizhouense ARSEF 977
KIE03653 Long chain acyl CoA synthetase partial Metarhizium majus ARSEF 297
XP 007818339 acyl CoA synthetase Metarhizium robertsii ARSEF 23
XP 014547161 Long chain acyl CoA synthetase partial Metarhizium brunneum ARSEF 3297
KFG85084 Long chain acyl CoA synthetase Metarhizium anisopliae
KJK81918 Long chain fatty acid CoA ligase Metarhizium anisopliae BRIP 53293
KJK86762 Long chain fatty acid CoA ligase Metarhizium anisopliae BRIP 53284
KJZ71152 hypothetical protein HIM 09451 Hirsutella minnesotensis 3608
EQL04038 Long chain acyl CoA synthetases and
PHH90773 hypothetical protein CDD83 2702 Cordyceps sp RAO-2017
PHH68331 hypothetical protein CDD80 7603 Ophiocordyceps camponoti rufipedis
PFH59779 hypothetical protein XA68 11887 Ophiocordyceps unilateralis
RDA93892 hypothetical protein CP533 4478 Ophiocordyceps sp camponoti saundersi
RDA88037 hypothetical protein CP532 5321 Ophiocordyceps sp camponoti leonardi
KYK55007 Long chain acyl CoA synthetase Drechmeria coniospora
PWI75563 Long chain acyl CoA synthetases & Acyl protein synthetase Purpureocillium lilacinum
XP 018174623 fatty acid activator Faa4 Purpureocillium lilacinum
POR34432 Long chain fatty acid CoA ligase 1 Tolypocladium paradoxum
PNY28678 Long chain fatty acid CoA ligase 1 Tolypocladium capitatum
KND88185 Long chain fatty acid CoA ligase 1 Tolypocladium ophioglossoides CBS 100239
XP 006670281 acyl CoA synthetase Cordyceps militaris CM01
ATY58447 acyl synthetase Cordyceps militaris
XP 018706564 acyl CoA synthetase Cordyceps fumosorosea ARSEF 2679
OAA48914 acyl CoA synthetase Cordyceps brongniartii RCEF 3172
OAA78976 acyl CoA synthetase Cordyceps confragosa RCEF 1005
OAR00467 hypothetical protein LLEC1 03987 Cordyceps confragosa
XP 024751639 acetyl CoA synthetase like protein Trichoderma citrinoviride
PTB73583 acetyl CoA synthetase like protein Trichoderma longibrachiatum ATCC 18648
ETS00933 long chain acyl CoA synthetase Trichoderma reesei RUT C30
XP 006966541 hypothetical protein TRIREDRAFT 49489 Trichoderma reesei QM6a
XP 024763672 hypothetical protein M441DRAFT 44111 Trichoderma asperellum CBS 43397
XP 018661860 long chain acyl CoA synthetase Trichoderma gamsii
XP 013942569 hypothetical protein TRIATDRAFT 150983 Trichoderma atroviride IMI 206040
XP 013958137 hypothetical protein TRIVIDRAFT 80870 Trichoderma virens Gv298
PNP60106 hypothetical protein THARTR1 00130 Trichoderma harzianum
XP 024775842 hypothetical protein M431DRAFT 481603 Trichoderma harzianum CBS 22695
KKO98561 long chain acyl CoA synthetase Trichoderma harzianum
OPB36296 long chain fatty acid CoA ligase Trichoderma guizhouense
CEJ91176 Putative Long chain acyl CoA synthetases & Acyl-protein synthetase Torrubiella hemipterigena
ACB12561 Fum16 Fusarium oxysporum
PNP80114 Fum16p Fusarium nygamai
XP 018742400 hypothetical protein FVEG 00326 Fusarium verticillioides 7600
XP 018742399 hypothetical protein FVEG 00326 Fusarium verticillioides 7600
RBR13868 hypothetical protein FVER53590 00326 Fusarium verticillioides
RBQ84966 hypothetical protein FVER53263 00326 Fusarium verticillioides
ANV81292 fatty acyl CoA synthase Fusarium fujikuroi
CZR47915 fumonisin cluster fatty acyl CoA Synthase Fusarium proliferatum ET1
CVL12746 fumonisin cluster fatty acyl CoA Synthase Fusarium proliferatum
RKL45828 Long chain fatty acid CoA ligase 1 Fusarium proliferatum
RBA10288 hypothetical protein FPRO05 06224 Fusarium proliferatum
KLO83567 fumonisin cluster fatty acyl CoA Synthase Fusarium fujikuroi
KLP17887 fumonisin cluster fatty acyl CoA Synthase Fusarium fujikuroi
SCO55626 fumonisin cluster fatty acyl CoA Synthase Fusarium fujikuroi
AFQ32308 FUM16 Fusarium fujikuroi
SCV52041 fumonisin cluster fatty acyl CoA Synthase Fusarium fujikuroi
XP 023435996 fumonisin cluster fatty acyl CoA Synthase Fusarium fujikuroi IMI 58289
KFH41237 Long chain fatty acid CoA ligase-like protein Acremonium chrysogenum ATCC 11550
KFA60310 hypothetical protein S40285 08220 Stachybotrys chlorohalonata IBT 40285
KFA76238 hypothetical protein S40288 03583 Stachybotrys chartarum IBT 40288
KEY70729 hypothetical protein S7711 03225 Stachybotrys chartarum IBT 7711
KFA50873 hypothetical protein S40293 02395 Stachybotrys chartarum IBT 40293
EWZ28040 long chain acyl CoA synthetase Fusarium oxysporum Fo47
RFN43386 long chain acyl-coa synthetase Fusarium sp FIESC 12
EXK77897 long chain acyl CoA synthetase Fusarium oxysporum f sp raphani 54005
RKL02187 Long chain fatty acid CoA ligase 1 Fusarium oxysporum
EXL64416 long chain acyl CoA synthetase Fusarium oxysporum f sp conglutinans race 2 54008
KPM36500 Long chain fatty acid CoA ligase 1 Neonectria ditissima
KLO98556 long chain fatty acid CoA ligase Fusarium fujikuroi
XP 023432102 related to long chain fatty acid CoA ligase Fusarium fujikuroi IMI 58289
XP 018745575 long chain acyl CoA synthetase Fusarium verticillioides 7600
RBQ94806 hypothetical protein FVER53263 02227 Fusarium verticillioides
PNP60776 hypothetical protein FNYG 14536 Fusarium nygamai
CVK90691 related to long chain fatty acid CoA ligase Fusarium mangiferae
EWY88812 hypothetical protein FOYG 09851 Fusarium oxysporum FOSC 3a
EGU77737 hypothetical protein FOXB 11759 Fusarium oxysporum Fo5176
EWZ39672 hypothetical protein FOZG 08694 Fusarium oxysporum Fo47
ENH65525 Long chain fatty acid CoA ligase 1 Fusarium oxysporum f sp cubense race 1
EXM09629 hypothetical protein FOIG 02375 Fusarium oxysporum f sp cubense tropical race 4 54006
RKK90998 Long chain fatty acid CoA ligase 1 Fusarium oxysporum
KIL91970 long chain acyl synthetase Fusarium avenaceum
RGP70168 long chain acyl synthetase Fusarium longipes
RFN49140 hypothetical protein FIE12Z 6632 Fusarium sp FIESC 12
RBR26524 hypothetical protein FIESC28 00710 Fusarium sp FIESC 28
KPA45244 long chain acyl synthetase Fusarium langsethiae
XP 025584444 uncharacterized protein FVRRES 05160 Fusarium venenatum
OBS23662 hypothetical protein FPOA 04210 Fusarium poae
XP 011320212 hypothetical protein FGSG 08543 Fusarium graminearum PH1
PTD04990 Long chain fatty acid CoA ligase 1 Fusarium culmorum
XP 009257815 hypothetical protein FPSE 06422 Fusarium pseudograminearum CS3096

FUM17

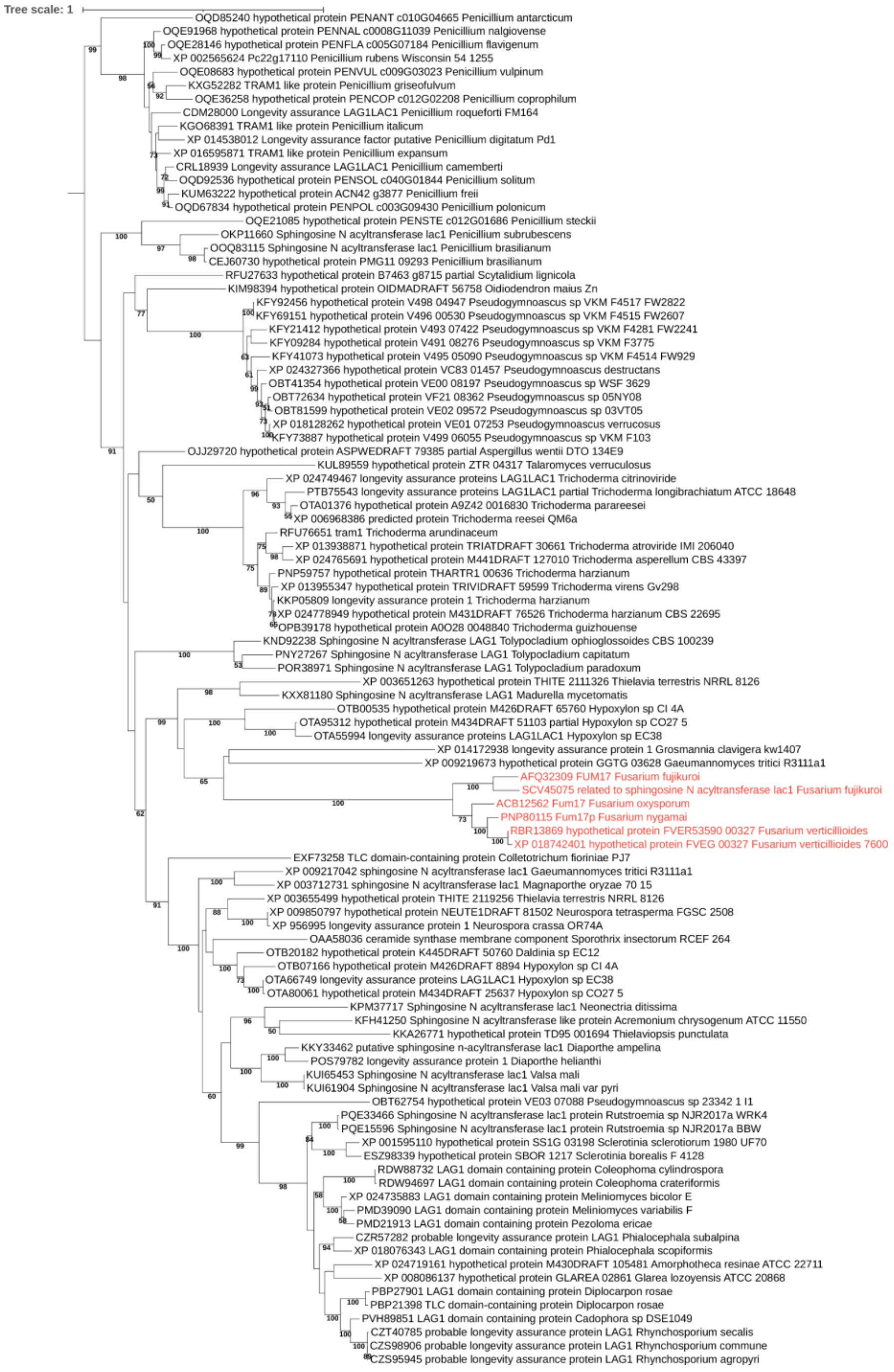
Tree scale: 1
OQD85240 hypothetical protein PENANT c010G04665 Penicillium antarcticum
OQE91968 hypothetical protein PENNAL c0008G11039 Penicillium nalgiovense
OQE28146 hypothetical protein PENFLA c005G07184 Penicillium flavigenum
XP 002565624 Pc22g17110 Penicillium rubens Wisconsin 54 1255
OQE08683 hypothetical protein PENVUL c009G03023 Penicillium vulpinum
KXG52282 TRAM1 like protein Penicillium griseofulvum
OQE36258 hypothetical protein PENCOP c012G02208 Penicillium coprophilum
CDM28000 Longevity assurance LAG1LAC1 Penicillium roqueforti FM164
KGO68391 TRAM1 like protein Penicillium italicum
XP 014538012 Longevity assurance factor putative Penicillium digitatum Pd1
XP 016595871 TRAM1 like protein Penicillium expansum
CRL18939 Longevity assurance LAG1LAC1 Penicillium camemberti
OQD92536 hypothetical protein PENSOL c040G01844 Penicillium solitum
KUM63222 hypothetical protein ACN42 g3877 Penicillium freii
OQD67834 hypothetical protein PENPOL c003G09430 Penicillium polonicum
OQE21085 hypothetical protein PENSTE c012G01686 Penicillium steckii
OKP11660 Sphingosine N acyltransferase lac1 Penicillium subrubescens
OOQ83115 Sphingosine N acyltransferase lac1 Penicillium brasilianum
CEJ60730 hypothetical protein PMG11 09293 Penicillium brasilianum
RFU27633 hypothetical protein B7463 g8715 partial Scytalidium lignicola
KIM98394 hypothetical protein OIDMADRAFT 56758 Oidiodendron maius Zn
KFY92456 hypothetical protein V498 04947 Pseudogymnoascus sp VKM F4517 FW2822
KFY69151 hypothetical protein V496 00530 Pseudogymnoascus sp VKM F4515 FW2607
KFY21412 hypothetical protein V493 07422 Pseudogymnoascus sp VKM F4281 FW2241
KFY09284 hypothetical protein V491 08276 Pseudogymnoascus sp VKM F3775
KFY41073 hypothetical protein V495 05090 Pseudogymnoascus sp VKM F4514 FW929
XP 024327366 hypothetical protein VC83 01457 Pseudogymnoascus destructans
OBT41354 hypothetical protein VE00 08197 Pseudogymnoascus sp WSF 3629
OBT72634 hypothetical protein VF21 08362 Pseudogymnoascus sp 05NY08
OBT81599 hypothetical protein VE02 09572 Pseudogymnoascus sp 03VT05
XP 018128262 hypothetical protein VE01 07253 Pseudogymnoascus verrucosus
KFY73887 hypothetical protein V499 06055 Pseudogymnoascus sp VKM F103
OJJ29720 hypothetical protein ASPWEDRAFT 79385 partial Aspergillus wentii DTO 134E9
KUL89559 hypothetical protein ZTR 04317 Talaromyces verruculosus
XP 024749467 longevity assurance proteins LAG1LAC1 Trichoderma citrinoviride
PTB75543 longevity assurance proteins LAG1LAC1 partial Trichoderma longibrachiatum ATCC 18648
OTA01376 hypothetical protein A9Z42 0016830 Trichoderma parareesei
XP 006968386 predicted protein Trichoderma reesei QM6a
RFU76651 tram1 Trichoderma arundinaceum
XP 013938871 hypothetical protein TRIATDRAFT 30661 Trichoderma atroviride IMI 206040
XP 024765691 hypothetical protein M441DRAFT 127010 Trichoderma asperellum CBS 43397
PNP59757 hypothetical protein THARTR1 00636 Trichoderma harzianum
XP 013955347 hypothetical protein TRIVIDRAFT 59599 Trichoderma virens Gv298
KKP05809 longevity assurance protein 1 Trichoderma harzianum
XP 024778949 hypothetical protein M431DRAFT 76526 Trichoderma harzianum CBS 22695
OPB39178 hypothetical protein A0O28 0048840 Trichoderma guizhouense
KND92238 Sphingosine N acyltransferase LAG1 Tolypocladium ophioglossoides CBS 100239
PNY27267 Sphingosine N acyltransferase LAG1 Tolypocladium capitatum
POR38971 Sphingosine N acyltransferase LAG1 Tolypocladium paradoxum
XP 003651263 hypothetical protein THITE 2111326 Thielavia terrestris NRRL 8126
KXX81180 Sphingosine N acyltransferase LAG1 Madurella mycetomatis
OTB00535 hypothetical protein M426DRAFT 65760 Hypoxylon sp CI 4A
OTA95312 hypothetical protein M434DRAFT 51103 partial Hypoxylon sp CO27 5
OTA55994 longevity assurance proteins LAG1LAC1 Hypoxylon sp EC38
XP 014172938 longevity assurance protein 1 Grosmannia clavigera kw1407
XP 009219673 hypothetical protein GGTG 03628 Gaeumannomyces tritici R3111a1
AFQ32309 FUM17 Fusarium fujikuroi
SCV45075 related to sphingosine N acyltransferase lac1 Fusarium fujikuroi
ACB12562 Fum17 Fusarium oxysporum
PNP80115 Fum17p Fusarium nygamai
RBR13869 hypothetical protein FVER53590 00327 Fusarium verticillioides
XP 018742401 hypothetical protein FVEG 00327 Fusarium verticillioides 7600
EXF73258 TLC domain-containing protein Colletotrichum fioriniae PJ7
XP 009217042 sphingosine N acyltransferase lac1 Gaeumannomyces tritici R3111a1
XP 003712731 sphingosine N acyltransferase lac1 Magnaporthe oryzae 70 15
XP 003655499 hypothetical protein THITE 2119256 Thielavia terrestris NRRL 8126
XP 009850797 hypothetical protein NEUTE1DRAFT 81502 Neurospora tetrasperma FGSC 2508
XP 956995 longevity assurance protein 1 Neurospora crassa OR74A
OAA58036 ceramide synthase membrane component Sporothrix insectorum RCEF 264
OTB20182 hypothetical protein K445DRAFT 50760 Daldinia sp EC12
OTB07166 hypothetical protein M426DRAFT 8894 Hypoxylon sp CI 4A
OTA66749 longevity assurance proteins LAG1LAC1 Hypoxylon sp EC38
OTA80061 hypothetical protein M434DRAFT 25637 Hypoxylon sp CO27 5
KPM37717 Sphingosine N acyltransferase lac1 Neonectria ditissima
KFH41250 Sphingosine N acyltransferase like protein Acremonium chrysogenum ATCC 11550
KKA26771 hypothetical protein TD95 001694 Thielaviopsis punctulata
KKY33462 putative sphingosine n-acyltransferase lac1 Diaporthe ampelina
POS79782 longevity assurance protein 1 Diaporthe helianthi
KUI65453 Sphingosine N acyltransferase lac1 Valsa mali
KUI61904 Sphingosine N acyltransferase lac1 Valsa mali var pyri
OBT62754 hypothetical protein VE03 07088 Pseudogymnoascus sp 23342 1 I1
PQE33466 Sphingosine N acyltransferase lac1 protein Rutstroemia sp NJR2017a WRK4
PQE15596 Sphingosine N acyltransferase lac1 protein Rutstroemia sp NJR2017a BBW
XP 001595110 hypothetical protein SS1G 03198 Sclerotinia sclerotiorum 1980 UF70
ESZ98339 hypothetical protein SBOR 1217 Sclerotinia borealis F 4128
RDW88732 LAG1 domain containing protein Coleophoma cylindrospora
RDW94697 LAG1 domain containing protein Coleophoma crateriformis
XP 024735883 LAG1 domain containing protein Meliniomyces bicolor E
PMD39090 LAG1 domain containing protein Meliniomyces variabilis F
PMD21913 LAG1 domain containing protein Pezoloma ericae
CZR57282 probable longevity assurance protein LAG1 Phialocephala subalpina
XP 018076343 LAG1 domain containing protein Phialocephala scopiformis
XP 024719161 hypothetical protein M430DRAFT 105481 Amorphotheca resinae ATCC 22711
XP 008086137 hypothetical protein GLAREA 02861 Glarea lozoyensis ATCC 20868
PBP27901 LAG1 domain containing protein Diplocarpon rosae
PBP21398 TLC domain-containing protein Diplocarpon rosae
PVH89851 LAG1 domain containing protein Cadophora sp DSE1049
CZT40785 probable longevity assurance protein LAG1 Rhynchosporium secalis
CZS98906 probable longevity assurance protein LAG1 Rhynchosporium commune
CZS95945 probable longevity assurance protein LAG1 Rhynchosporium agropyri

FUM18

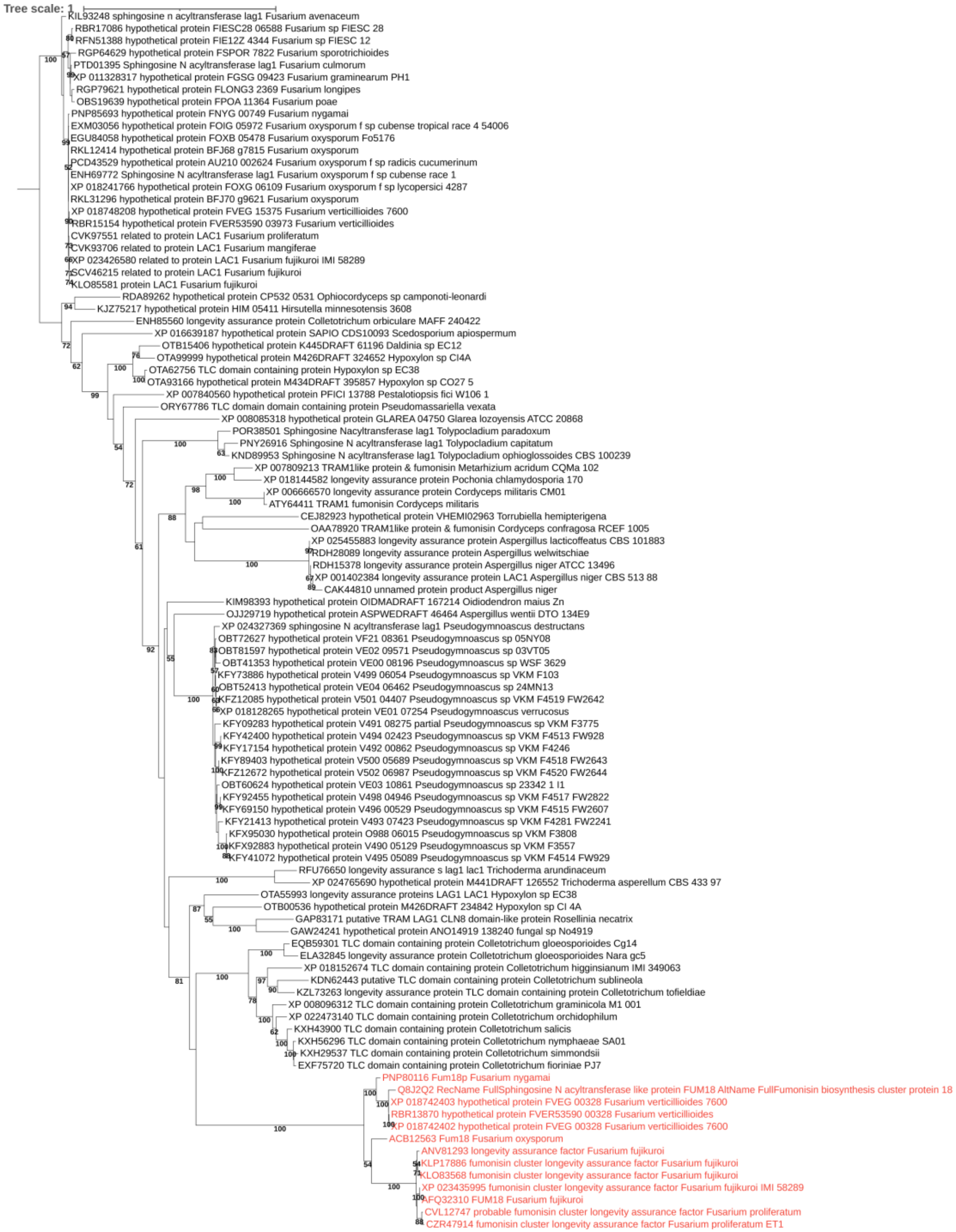

Tree scale: 1
KIL93248 sphingosine n acyltransferase lag1 Fusarium avenaceum
RBR17086 hypothetical protein FIESC28 06588 Fusarium sp FIESC 28
RFN51388 hypothetical protein FIE12Z 4344 Fusarium sp FIESC 12
RGP64629 hypothetical protein FSPOR 7822 Fusarium sporotrichioides
PTD01395 Sphingosine N acyltransferase lag1 Fusarium culmorum
XP 011328317 hypothetical protein FGSG 09423 Fusarium graminearum PH1
RGP79621 hypothetical protein FLONG3 2369 Fusarium longipes
OBS19639 hypothetical protein FPOA 11364 Fusarium poae
PNP85693 hypothetical protein FNYG 00749 Fusarium nygamai
EXM03056 hypothetical protein FOIG 05972 Fusarium oxysporum f sp cubense tropical race 4 54006
EGU84058 hypothetical protein FOXB 05478 Fusarium oxysporum Fo5176
RKL12414 hypothetical protein BFJ68 g7815 Fusarium oxysporum
PCD43529 hypothetical protein AU210 002624 Fusarium oxysporum f sp radicis cucumerinum
ENH69772 Sphingosine N acyltransferase lag1 Fusarium oxysporum f sp cubense race 1
XP 018241766 hypothetical protein FOXG 06109 Fusarium oxysporum f sp lycopersici 4287
RKL31296 hypothetical protein BFJ70 g9621 Fusarium oxysporum
XP 018748208 hypothetical protein FVEG 15375 Fusarium verticillioides 7600
RBR15154 hypothetical protein FVER53590 03973 Fusarium verticillioides
CVK97551 related to protein LAC1 Fusarium proliferatum
CVK93706 related to protein LAC1 Fusarium mangiferae
XP 023426580 related to protein LAC1 Fusarium fujikuroi IMI 58289
SCV46215 related to protein LAC1 Fusarium fujikuroi
KLO85581 protein LAC1 Fusarium fujikuroi
RDA89262 hypothetical protein CP532 0531 Ophiocordyceps sp camponoti-leonardi
KJZ75217 hypothetical protein HIM 05411 Hirsutella minnesotensis 3608
ENH85560 longevity assurance protein Colletotrichum orbiculare MAFF 240422
XP 016639187 hypothetical protein SAPIO CDS10093 Scedosporium apiospermum
OTB15406 hypothetical protein K445DRAFT 61196 Daldinia sp EC12
OTA99999 hypothetical protein M426DRAFT 324652 Hypoxylon sp CI4A
OTA62756 TLC domain containing protein Hypoxylon sp EC38
OTA93166 hypothetical protein M434DRAFT 395857 Hypoxylon sp CO27 5
XP 007840560 hypothetical protein PFICI 13788 Pestalotiopsis fici W106 1
ORY67786 TLC domain domain containing protein Pseudomassariella vexata
XP 008085318 hypothetical protein GLAREA 04750 Glarea lozoyensis ATCC 20868
POR38501 Sphingosine Nacyltransferase lag1 Tolypocladium paradoxum
PNY26916 Sphingosine N acyltransferase lag1 Tolypocladium capitatum
KND89953 Sphingosine N acyltransferase lag1 Tolypocladium ophioglossoides CBS 100239
XP 007809213 TRAM1like protein & fumonisin Metarhizium acridum CQMa 102
XP 018144582 longevity assurance protein Pochonia chlamydosporia 170
XP 006666570 longevity assurance protein Cordyceps militaris CM01
ATY64411 TRAM1 fumonisin Cordyceps militaris
CEJ82923 hypothetical protein VHEMI02963 Torrubiella hemipterigena
OAA78920 TRAM1like protein & fumonisin Cordyceps confragosa RCEF 1005
XP 025455883 longevity assurance protein Aspergillus lacticoffeatus CBS 101883
RDH28089 longevity assurance protein Aspergillus welwitschiae
RDH15378 longevity assurance protein Aspergillus niger ATCC 13496
XP 001402384 longevity assurance protein LAC1 Aspergillus niger CBS 513 88
CAK44810 unnamed protein product Aspergillus niger
KIM98393 hypothetical protein OIDMADRAFT 167214 Oidiodendron maius Zn
OJJ29719 hypothetical protein ASPWEDRAFT 46464 Aspergillus wentii DTO 134E9
XP 024327369 sphingosine N acyltransferase lag1 Pseudogymnoascus destructans
OBT72627 hypothetical protein VF21 08361 Pseudogymnoascus sp 05NY08
OBT81597 hypothetical protein VE02 09571 Pseudogymnoascus sp 03VT05
OBT41353 hypothetical protein VE00 08196 Pseudogymnoascus sp WSF 3629
KFY73886 hypothetical protein V499 06054 Pseudogymnoascus sp VKM F103
OBT52413 hypothetical protein VE04 06462 Pseudogymnoascus sp 24MN13
KFZ12085 hypothetical protein V501 04407 Pseudogymnoascus sp VKM F4519 FW2642
XP 018128265 hypothetical protein VE01 07254 Pseudogymnoascus verrucosus
KFY09283 hypothetical protein V491 08275 partial Pseudogymnoascus sp VKM F3775
KFY42400 hypothetical protein V494 02423 Pseudogymnoascus sp VKM F4513 FW928
KFY17154 hypothetical protein V492 00862 Pseudogymnoascus sp VKM F4246
KFY89403 hypothetical protein V500 05689 Pseudogymnoascus sp VKM F4518 FW2643
KFZ12672 hypothetical protein V502 06987 Pseudogymnoascus sp VKM F4520 FW2644
OBT60624 hypothetical protein VE03 10861 Pseudogymnoascus sp 23342 1 I1
KFY92455 hypothetical protein V498 04946 Pseudogymnoascus sp VKM F4517 FW2822
KFY69150 hypothetical protein V496 00529 Pseudogymnoascus sp VKM F4515 FW2607
KFY21413 hypothetical protein V493 07423 Pseudogymnoascus sp VKM F4281 FW2241
KFX95030 hypothetical protein O988 06015 Pseudogymnoascus sp VKM F3808
KFX92883 hypothetical protein V490 05129 Pseudogymnoascus sp VKM F3557
KFY41072 hypothetical protein V495 05089 Pseudogymnoascus sp VKM F4514 FW929
RFU76650 longevity assurance s lag1 lac1 Trichoderma arundinaceum
XP 024765690 hypothetical protein M441DRAFT 126552 Trichoderma asperellum CBS 433 97
OTA55993 longevity assurance proteins LAG1 LAC1 Hypoxylon sp EC38
OTB00536 hypothetical protein M426DRAFT 234842 Hypoxylon sp CI 4A
GAP83171 putative TRAM LAG1 CLN8 domain-like protein Rosellinia necatrix
GAW24241 hypothetical protein ANO14919 138240 fungal sp No4919
EQB59301 TLC domain containing protein Colletotrichum gloeosporioides Cg14
ELA32845 longevity assurance protein Colletotrichum gloeosporioides Nara gc5
XP 018152674 TLC domain containing protein Colletotrichum higginsianum IMI 349063
KDN62443 putative TLC domain containing protein Colletotrichum sublineola
KZL73263 longevity assurance protein TLC domain containing protein Colletotrichum tofieldiae
XP 008096312 TLC domain containing protein Colletotrichum graminicola M1 001
XP 022473140 TLC domain containing protein Colletotrichum orchidophilum
KXH43900 TLC domain containing protein Colletotrichum salicis
KXH56296 TLC domain containing protein Colletotrichum nymphaeae SA01
KXH29537 TLC domain containing protein Colletotrichum simmondsii
EXF75720 TLC domain containing protein Colletotrichum fioriniae PJ7
PNP80116 Fum18p Fusarium nygamai
Q8J2Q2 RecName FullSphingosine N acyltransferase like protein FUM18 AltName FullFumonisin biosynthesis cluster protein 18
XP 018742403 hypothetical protein FVEG 00328 Fusarium verticillioides 7600
RBR13870 hypothetical protein FVER53590 00328 Fusarium verticillioides
XP 018742402 hypothetical protein FVEG 00328 Fusarium verticillioides 7600
ACB12563 Fum18 Fusarium oxysporum
ANV81293 longevity assurance factor Fusarium fujikuroi
KLP17886 fumonisin cluster longevity assurance factor Fusarium fujikuroi
KLO83568 fumonisin cluster longevity assurance factor Fusarium fujikuroi
XP 023435995 fumonisin cluster longevity assurance factor Fusarium fujikuroi IMI 58289
AFQ32310 FUM18 Fusarium fujikuroi
CVL12747 probable fumonisin cluster longevity assurance factor Fusarium proliferatum
CZR47914 fumonisin cluster longevity assurance factor Fusarium proliferatum ET1

FUM19

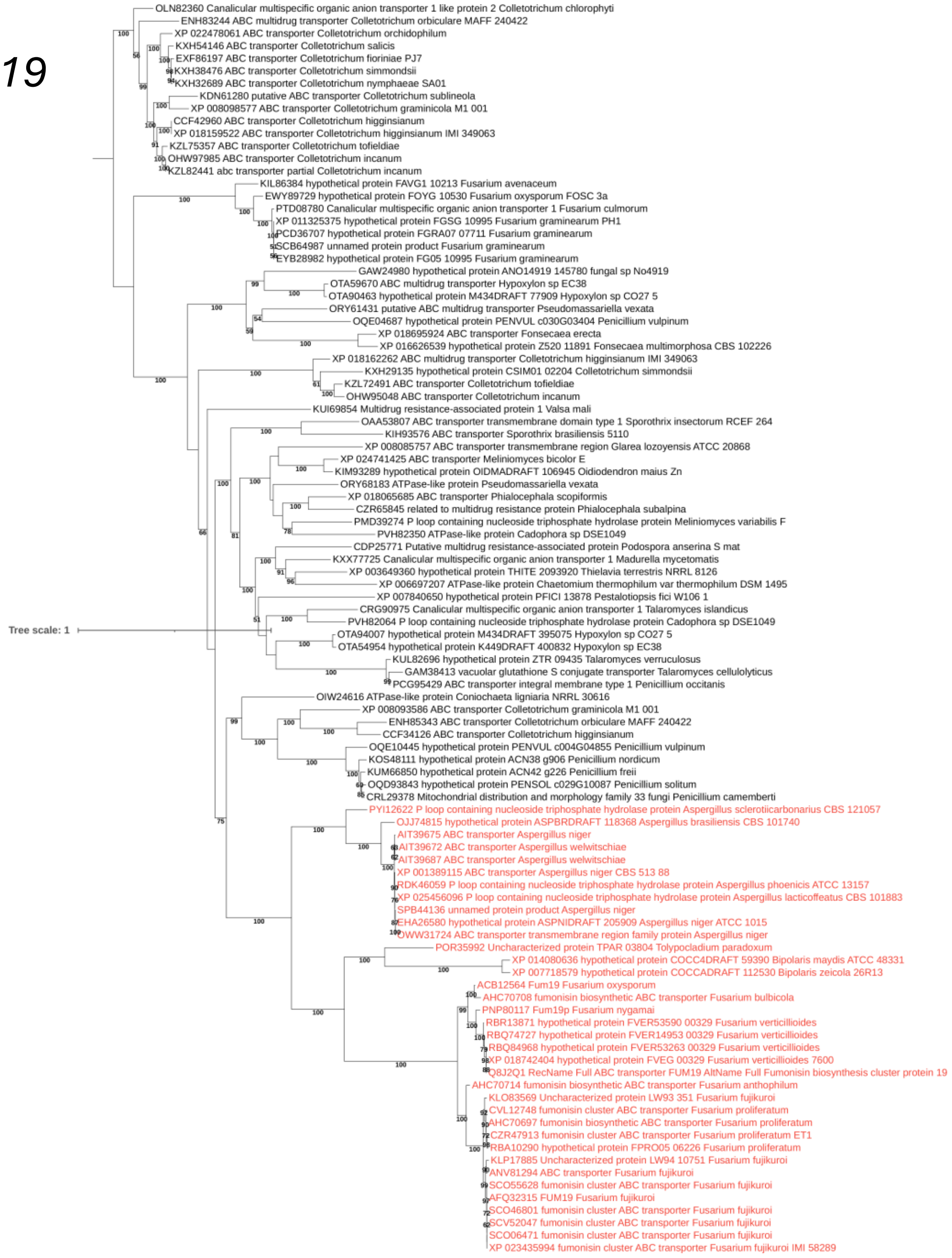
OLN82360 Canalicular multispecific organic anion transporter 1 like protein 2 Colletotrichum chlorophyti
ENH83244 ABC multidrug transporter Colletotrichum orbiculare MAFF 240422
XP 022478061 ABC transporter Colletotrichum orchidophilum
KXH54146 ABC transporter Colletotrichum salicis
EXF86197 ABC transporter Colletotrichum fioriniae PJ7
KXH38476 ABC transporter Colletotrichum simmondsii
KXH32689 ABC transporter Colletotrichum nymphaeae SA01
KDN61280 putative ABC transporter Colletotrichum sublineola
XP 008098577 ABC transporter Colletotrichum graminicola M1 001
CCF42960 ABC transporter Colletotrichum higginsianum
XP 018159522 ABC transporter Colletotrichum higginsianum IMI 349063
KZL75357 ABC transporter Colletotrichum tofieldiae
OHW97985 ABC transporter Colletotrichum incanum
KZL82441 abc transporter partial Colletotrichum incanum
KIL86384 hypothetical protein FAVG1 10213 Fusarium avenaceum
EWY89729 hypothetical protein FOYG 10530 Fusarium oxysporum FOSC 3a
PTD08780 Canalicular multispecific organic anion transporter 1 Fusarium culmorum
XP 011325375 hypothetical protein FGSG 10995 Fusarium graminearum PH1
PCD36707 hypothetical protein FGRA07 07711 Fusarium graminearum
SCB64987 unnamed protein product Fusarium graminearum
EYB28982 hypothetical protein FG05 10995 Fusarium graminearum
GAW24980 hypothetical protein ANO14919 145780 fungal sp No4919
OTA59670 ABC multidrug transporter Hypoxylon sp EC38
OTA90463 hypothetical protein M434DRAFT 77909 Hypoxylon sp CO27 5
ORY61431 putative ABC multidrug transporter Pseudomassariella vexata
OQE04687 hypothetical protein PENVUL c030G03404 Penicillium vulpinum
XP 018695924 ABC transporter Fonsecaea erecta
XP 016626539 hypothetical protein Z520 11891 Fonsecaea multimorphosa CBS 102226
XP 018162262 ABC multidrug transporter Colletotrichum higginsianum IMI 349063
KXH29135 hypothetical protein CSIM01 02204 Colletotrichum simmondsii
KZL72491 ABC transporter Colletotrichum tofieldiae
OHW95048 ABC transporter Colletotrichum incanum
KUI69854 Multidrug resistance-associated protein 1 Valsa mali
OAA53807 ABC transporter transmembrane domain type 1 Sporothrix insectorum RCEF 264
KIH93576 ABC transporter Sporothrix brasiliensis 5110
XP 008085757 ABC transporter transmembrane region Glarea lozoyensis ATCC 20868
XP 024741425 ABC transporter Meliniomyces bicolor E
KIM93289 hypothetical protein OIDMADRAFT 106945 Oidiodendron maius Zn
ORY68183 ATPase-like protein Pseudomassariella vexata
XP 018065685 ABC transporter Phialocephala scopiformis
CZR65845 related to multidrug resistance protein Phialocephala subalpina
PMD39274 P loop containing nucleoside triphosphate hydrolase protein Meliniomyces variabilis F
PVH82350 ATPase-like protein Cadophora sp DSE1049
CDP25771 Putative multidrug resistance-associated protein Podospora anserina S mat
KXX77725 Canalicular multispecific organic anion transporter 1 Madurella mycetomatis
XP 003649360 hypothetical protein THITE 2093920 Thielavia terrestris NRRL 8126
XP 006697207 ATPase-like protein Chaetomium thermophilum var thermophilum DSM 1495
XP 007840650 hypothetical protein PFICI 13878 Pestalotiopsis fici W106 1
CRG90975 Canalicular multispecific organic anion transporter 1 Talaromyces islandicus
PVH82064 P loop containing nucleoside triphosphate hydrolase protein Cadophora sp DSE1049
OTA94007 hypothetical protein M434DRAFT 395075 Hypoxylon sp CO27 5
OTA54954 hypothetical protein K449DRAFT 400832 Hypoxylon sp EC38
KUL82696 hypothetical protein ZTR 09435 Talaromyces verruculosus
GAM38413 vacuolar glutathione S conjugate transporter Talaromyces cellulolyticus
PCG95429 ABC transporter integral membrane type 1 Penicillium occitanis
OIW24616 ATPase-like protein Coniochaeta ligniaria NRRL 30616
XP 008093586 ABC transporter Colletotrichum graminicola M1 001
ENH85343 ABC transporter Colletotrichum orbiculare MAFF 240422
CCF34126 ABC transporter Colletotrichum higginsianum
OQE10445 hypothetical protein PENVUL c004G04855 Penicillium vulpinum
KOS48111 hypothetical protein ACN38 g906 Penicillium nordicum
KUM66850 hypothetical protein ACN42 g226 Penicillium freii
OQD93843 hypothetical protein PENSOL c029G10087 Penicillium solitum
CRL29378 Mitochondrial distribution and morphology family 33 fungi Penicillium camemberti
PYI12622 P loop containing nucleoside triphosphate hydrolase protein Aspergillus sclerotiicarbonarius CBS 121057
OJJ74815 hypothetical protein ASPBRDRAFT 118368 Aspergillus brasiliensis CBS 101740
AIT39675 ABC transporter Aspergillus niger
AIT39672 ABC transporter Aspergillus welwitschiae
AIT39687 ABC transporter Aspergillus welwitschiae
XP 001389115 ABC transporter Aspergillus niger CBS 513 88
RDK46059 P loop containing nucleoside triphosphate hydrolase protein Aspergillus phoenicis ATCC 13157
XP 025456096 P loop containing nucleoside triphosphate hydrolase protein Aspergillus lacticoffeatus CBS 101883
SPB44136 unnamed protein product Aspergillus niger
EHA26580 hypothetical protein ASPNIDRAFT 205909 Aspergillus niger ATCC 1015
OWW31724 ABC transporter transmembrane region family protein Aspergillus niger
POR35992 Uncharacterized protein TPAR 03804 Tolypocladium paradoxum
XP 014080636 hypothetical protein COCC4DRAFT 59390 Bipolaris maydis ATCC 48331
XP 007718579 hypothetical protein COCCADRAFT 112530 Bipolaris zeicola 26R13
ACB12564 Fum19 Fusarium oxysporum
AHC70708 fumonisin biosynthetic ABC transporter Fusarium bulbicola
PNP80117 Fum19p Fusarium nygamai
RBR13871 hypothetical protein FVER53590 00329 Fusarium verticillioides
RBQ74727 hypothetical protein FVER14953 00329 Fusarium verticillioides
RBQ84968 hypothetical protein FVER53263 00329 Fusarium verticillioides
XP 018742404 hypothetical protein FVEG 00329 Fusarium verticillioides 7600
Q8J2Q1 RecName Full ABC transporter FUM19 AltName Full Fumonisin biosynthesis cluster protein 19
AHC70714 fumonisin biosynthetic ABC transporter Fusarium anthophilum
KLO83569 Uncharacterized protein LW93 351 Fusarium fujikuroi
CVL12748 fumonisin cluster ABC transporter Fusarium proliferatum
AHC70697 fumonisin biosynthetic ABC transporter Fusarium proliferatum
CZR47913 fumonisin cluster ABC transporter Fusarium proliferatum ET1
RBA10290 hypothetical protein FPRO05 06226 Fusarium proliferatum
KLP17885 Uncharacterized protein LW94 10751 Fusarium fujikuroi
ANV81294 ABC transporter Fusarium fujikuroi
SCO55628 fumonisin cluster ABC transporter Fusarium fujikuroi
AFQ32315 FUM19 Fusarium fujikuroi
SCO46801 fumonisin cluster ABC transporter Fusarium fujikuroi
SCV52047 fumonisin cluster ABC transporter Fusarium fujikuroi
SCO06471 fumonisin cluster ABC transporter Fusarium fujikuroi
XP 023435994 fumonisin cluster ABC transporter Fusarium fujikuroi IMI 58289
Tree scale: 1

FUM21

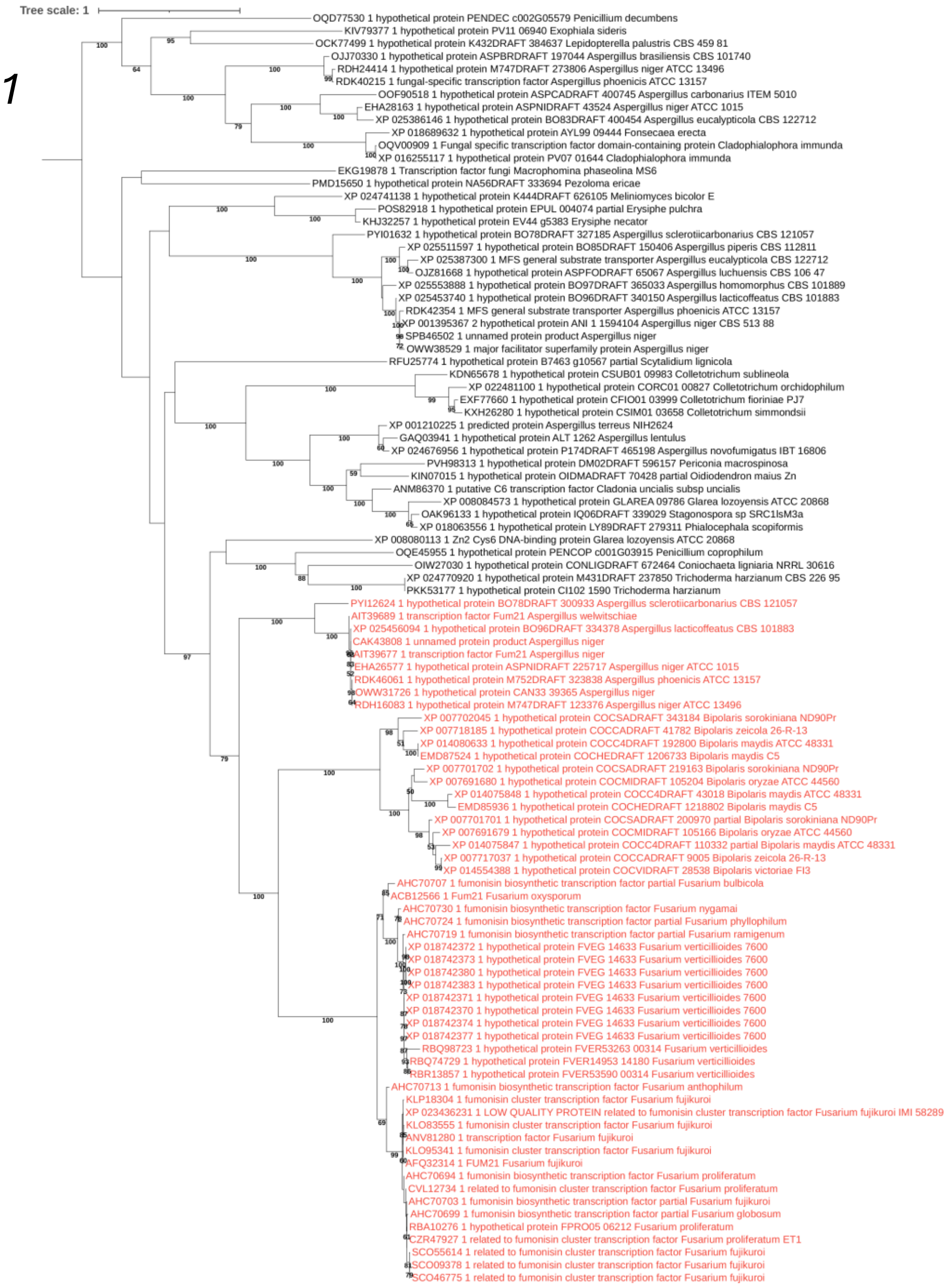
Tree scale: 1
OQD77530 1 hypothetical protein PENDEC c002G05579 Penicillium decumbens
KIV79377 1 hypothetical protein PV11 06940 Exophiala sideris
OCK77499 1 hypothetical protein K432DRAFT 384637 Lepidopterella palustris CBS 459 81
OJJ70330 1 hypothetical protein ASPBRDRAFT 197044 Aspergillus brasiliensis CBS 101740
RDH24414 1 hypothetical protein M747DRAFT 273806 Aspergillus niger ATCC 13496
RDK40215 1 fungal-specific transcription factor Aspergillus phoenicis ATCC 13157
OOF90518 1 hypothetical protein ASPCADRAFT 400745 Aspergillus carbonarius ITEM 5010
EHA28163 1 hypothetical protein ASPNIDRAFT 43524 Aspergillus niger ATCC 1015
XP 025386146 1 hypothetical protein BO83DRAFT 400454 Aspergillus eucalypticola CBS 122712
XP 018689632 1 hypothetical protein AYL99 09444 Fonsecaea erecta
OQV00909 1 Fungal specific transcription factor domain-containing protein Cladophialophora immunda
XP 016255117 1 hypothetical protein PV07 01644 Cladophialophora immunda
EKG19878 1 Transcription factor fungi Macrophomina phaseolina MS6
PMD15650 1 hypothetical protein NA56DRAFT 333694 Pezoloma ericae
XP 024741138 1 hypothetical protein K444DRAFT 626105 Meliniomyces bicolor E
POS82918 1 hypothetical protein EPUL 004074 partial Erysiphe pulchra
KHJ32257 1 hypothetical protein EV44 g5383 Erysiphe necator
PYI01632 1 hypothetical protein BO78DRAFT 327185 Aspergillus sclerotiicarbonarius CBS 121057
XP 025511597 1 hypothetical protein BO85DRAFT 150406 Aspergillus piperis CBS 112811
XP 025387300 1 MFS general substrate transporter Aspergillus eucalypticola CBS 122712
OJZ81668 1 hypothetical protein ASPFODRAFT 65067 Aspergillus luchuensis CBS 106 47
XP 025553888 1 hypothetical protein BO97DRAFT 365033 Aspergillus homomorphus CBS 101889
XP 025453740 1 hypothetical protein BO96DRAFT 340150 Aspergillus lacticoffeatus CBS 101883
RDK42354 1 MFS general substrate transporter Aspergillus phoenicis ATCC 13157
XP 001395367 2 hypothetical protein ANI 1 1594104 Aspergillus niger CBS 513 88
SPB46502 1 unnamed protein product Aspergillus niger
OWW38529 1 major facilitator superfamily protein Aspergillus niger
RFU25774 1 hypothetical protein B7463 g10567 partial Scytalidium lignicola
KDN65678 1 hypothetical protein CSUB01 09983 Colletotrichum sublineola
XP 022481100 1 hypothetical protein CORC01 00827 Colletotrichum orchidophilum
EXF77660 1 hypothetical protein CFIO01 03999 Colletotrichum fioriniae PJ7
KXH26280 1 hypothetical protein CSIM01 03658 Colletotrichum simmondsii
XP 001210225 1 predicted protein Aspergillus terreus NIH2624
GAQ03941 1 hypothetical protein ALT 1262 Aspergillus lentulus
XP 024676956 1 hypothetical protein P174DRAFT 465198 Aspergillus novofumigatus IBT 16806
PVH98313 1 hypothetical protein DM02DRAFT 596157 Periconia macrospinosa
KIN07015 1 hypothetical protein OIDMADRAFT 70428 partial Oidiodendron maius Zn
ANM86370 1 putative C6 transcription factor Cladonia uncialis subsp uncialis
XP 008084573 1 hypothetical protein GLAREA 09786 Glarea lozoyensis ATCC 20868
OAK96133 1 hypothetical protein IQ06DRAFT 339029 Stagonospora sp SRC1lsM3a
XP 018063556 1 hypothetical protein LY89DRAFT 279311 Phialocephala scopiformis
XP 008080113 1 Zn2 Cys6 DNA-binding protein Glarea lozoyensis ATCC 20868
OQE45955 1 hypothetical protein PENCOP c001G03915 Penicillium coprophilum
OIW27030 1 hypothetical protein CONLIGDRAFT 672464 Coniochaeta ligniaria NRRL 30616
XP 024770920 1 hypothetical protein M431DRAFT 237850 Trichoderma harzianum CBS 226 95
PKK53177 1 hypothetical protein CI102 1590 Trichoderma harzianum
PYI12624 1 hypothetical protein BO78DRAFT 300933 Aspergillus sclerotiicarbonarius CBS 121057
AIT39689 1 transcription factor Fum21 Aspergillus welwitschiae
XP 025456094 1 hypothetical protein BO96DRAFT 334378 Aspergillus lacticoffeatus CBS 101883
CAK43808 1 unnamed protein product Aspergillus niger
AIT39677 1 transcription factor Fum21 Aspergillus niger
EHA26577 1 hypothetical protein ASPNIDRAFT 225717 Aspergillus niger ATCC 1015
RDK46061 1 hypothetical protein M752DRAFT 323838 Aspergillus phoenicis ATCC 13157
OWW31726 1 hypothetical protein CAN33 39365 Aspergillus niger
RDH16083 1 hypothetical protein M747DRAFT 123376 Aspergillus niger ATCC 13496
XP 007702045 1 hypothetical protein COCSADRAFT 343184 Bipolaris sorokiniana ND90Pr
XP 007718185 1 hypothetical protein COCCADRAFT 41782 Bipolaris zeicola 26-R-13
XP 014080633 1 hypothetical protein COCC4DRAFT 192800 Bipolaris maydis ATCC 48331
EMD87524 1 hypothetical protein COCHEDRAFT 1206733 Bipolaris maydis C5
XP 007701702 1 hypothetical protein COCSADRAFT 219163 Bipolaris sorokiniana ND90Pr
XP 007691680 1 hypothetical protein COCMIDRAFT 105204 Bipolaris oryzae ATCC 44560
XP 014075848 1 hypothetical protein COCC4DRAFT 43018 Bipolaris maydis ATCC 48331
EMD85936 1 hypothetical protein COCHEDRAFT 1218802 Bipolaris maydis C5
XP 007701701 1 hypothetical protein COCSADRAFT 200970 partial Bipolaris sorokiniana ND90Pr
XP 007691679 1 hypothetical protein COCMIDRAFT 105166 Bipolaris oryzae ATCC 44560
XP 014075847 1 hypothetical protein COCC4DRAFT 110332 partial Bipolaris maydis ATCC 48331
XP 007717037 1 hypothetical protein COCCADRAFT 9005 Bipolaris zeicola 26-R-13
XP 014554388 1 hypothetical protein COCVIDRAFT 28538 Bipolaris victoriae FI3
AHC70707 1 fumonisin biosynthetic transcription factor partial Fusarium bulbicola
ACB12566 1 Fum21 Fusarium oxysporum
AHC70730 1 fumonisin biosynthetic transcription factor Fusarium nygamai
AHC70724 1 fumonisin biosynthetic transcription factor partial Fusarium phyllophilum
AHC70719 1 fumonisin biosynthetic transcription factor partial Fusarium ramigenum
XP 018742372 1 hypothetical protein FVEG 14633 Fusarium verticillioides 7600
XP 018742373 1 hypothetical protein FVEG 14633 Fusarium verticillioides 7600
XP 018742380 1 hypothetical protein FVEG 14633 Fusarium verticillioides 7600
XP 018742383 1 hypothetical protein FVEG 14633 Fusarium verticillioides 7600
XP 018742371 1 hypothetical protein FVEG 14633 Fusarium verticillioides 7600
XP 018742370 1 hypothetical protein FVEG 14633 Fusarium verticillioides 7600
XP 018742374 1 hypothetical protein FVEG 14633 Fusarium verticillioides 7600
XP 018742377 1 hypothetical protein FVEG 14633 Fusarium verticillioides 7600
RBQ98723 1 hypothetical protein FVER53263 00314 Fusarium verticillioides
RBQ74729 1 hypothetical protein FVER14953 14180 Fusarium verticillioides
RBR13857 1 hypothetical protein FVER53590 00314 Fusarium verticillioides
AHC70713 1 fumonisin biosynthetic transcription factor Fusarium anthophilum
KLP18304 1 fumonisin cluster transcription factor Fusarium fujikuroi
XP 023436231 1 LOW QUALITY PROTEIN related to fumonisin cluster transcription factor Fusarium fujikuroi IMI 58289
KLO83555 1 fumonisin cluster transcription factor Fusarium fujikuroi
ANV81280 1 transcription factor Fusarium fujikuroi
KLO95341 1 fumonisin cluster transcription factor Fusarium fujikuroi
AFQ32314 1 FUM21 Fusarium fujikuroi
AHC70694 1 fumonisin biosynthetic transcription factor Fusarium proliferatum
CVL12734 1 related to fumonisin cluster transcription factor Fusarium proliferatum
AHC70703 1 fumonisin biosynthetic transcription factor partial Fusarium fujikuroi
AHC70699 1 fumonisin biosynthetic transcription factor partial Fusarium globosum
RBA10276 1 hypothetical protein FPRO05 06212 Fusarium proliferatum
CZR47927 1 related to fumonisin cluster transcription factor Fusarium proliferatum ET1
SCO55614 1 related to fumonisin cluster transcription factor Fusarium fujikuroi
SCO09378 1 related to fumonisin cluster transcription factor Fusarium fujikuroi
SCO46775 1 related to fumonisin cluster transcription factor Fusarium fujikuroi

Supplementary SFigure 2: Phylogenetic tree of fungal ceramide synthase genes, showing the relatively distant relationship of the *Fusarium FUM* cluster gene *FUM18* and the *Alternaria* and *Bipolaris FUM* cluster gene *ALT7*. The tree was inferred by maximum likelihood analysis of the predicted amino acid sequences of selected fungal ceramide synthase genes. Numbers near branches are bootstrap values based on 1000 pseudoreplicates. *FUM17* is not included in this tree, because it is more distantly related to *ALT7* and *FUM18* than the *Schizosaccharomyces pombe* gene used as the outgroup in this tree. In the tree, letter/number combinations placed after species names are protein accessions from the Joint Genome Institute (JGI) or NCBI/GenBank. JGI accession are preceded by JGI; all other accession are from NCBI/GenBank.

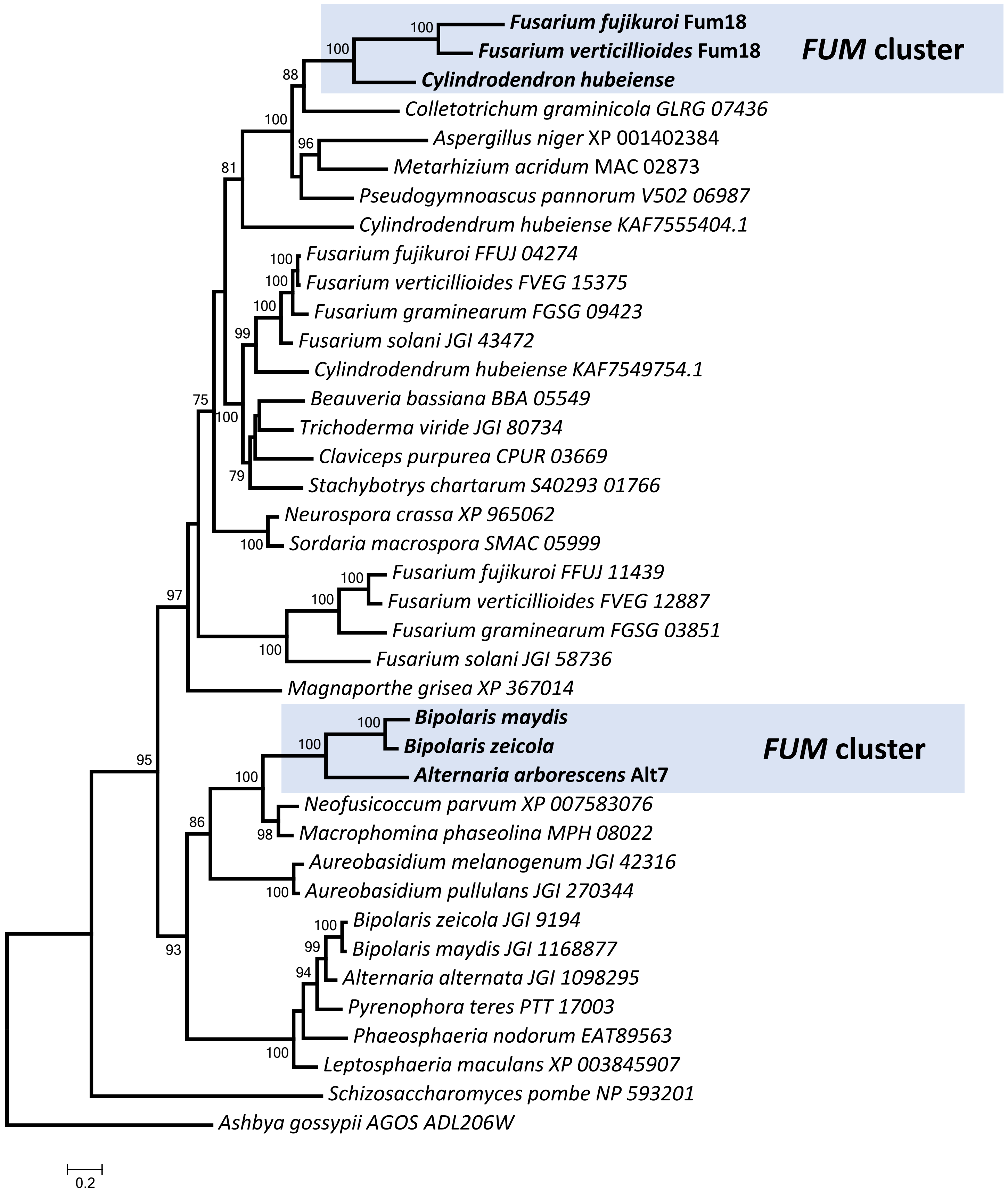

Supplementary Figure 3: Phylogenetic tree inferred from putative mitochondrial transporter genes. The tree shows the relatively distant relationship of the *Fusarium FUM* cluster gene *FUM11* and the *Alternaria arborescens FUM* cluster gene *ALT9*. The tree was inferred by maximum likelihood analysis of the predicted amino acid sequences of selected fungal mitochondrial transporter genes. Numbers near branches are bootstrap values based on 1000 pseudoreplicates. In the tree, the designations placed after the name *Alternaria arborescens* are strain designations. Letter/number combinations after all other species names are NCBI/GenBank accession numbers.

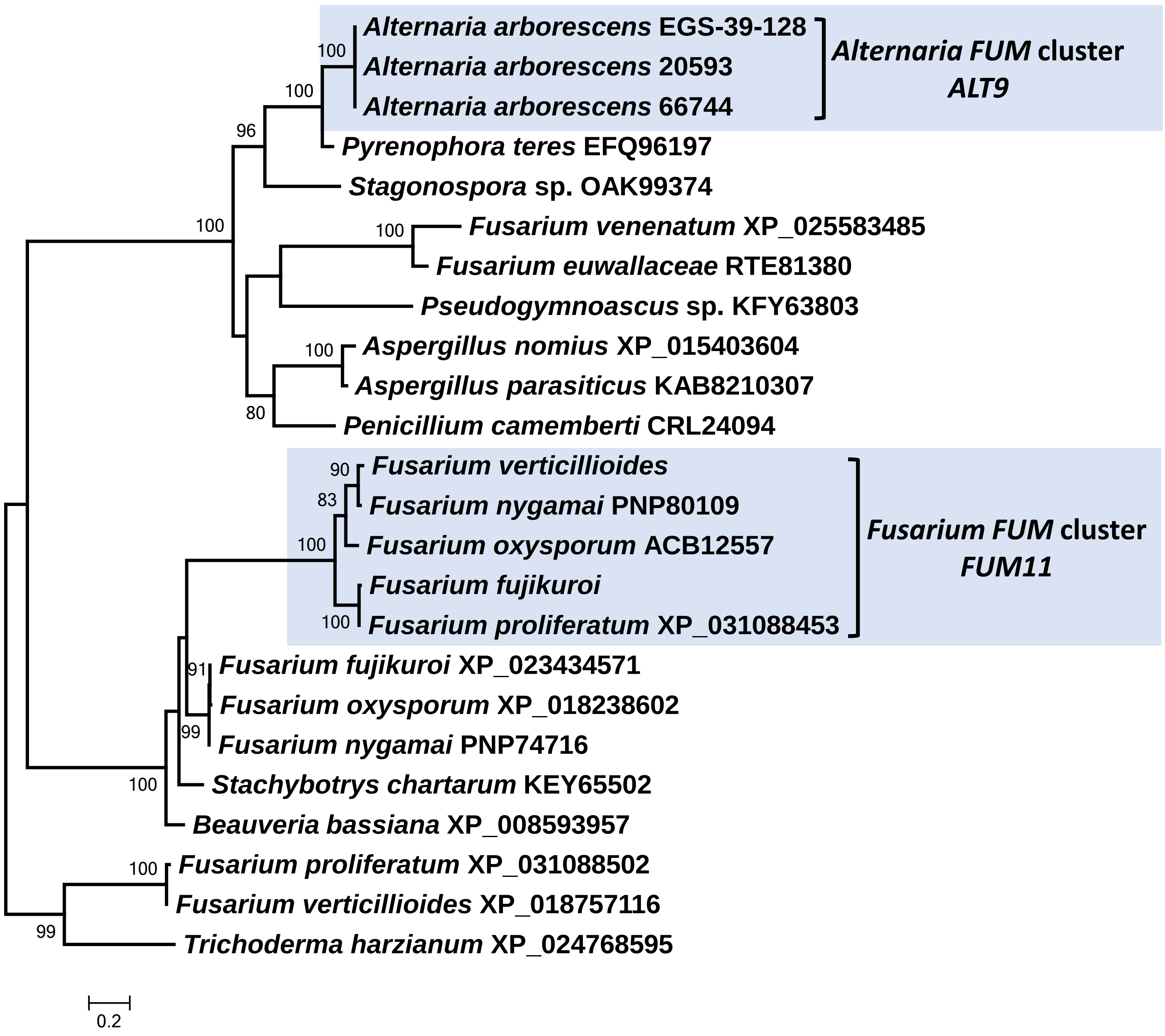

SFigure 4. Closure of sequence gaps between contigs from *FUM* cluster region of *Alternaria arborescens* strains NRRL 20593 and NRRL 66744. The ‘Map Reads to Reference’ function in CLC Genomics Workbench was used to map Illumina MiSeq sequence reads from NRRL 20593 or NRRL 66744 to the sequence of the *FUM* cluster region from strain As-27 (GenBank Accession AB969680.1). Manual inspection of the resulting mapped sequences indicated multiple 1 or 2-base indels and nucleotide substitutions in the NRRL 20593 and NRRL 66744 sequences relative to the As-27 sequence, but no large indels, nucleotide substitutions, or inversions. However, the mapped reads indicated a six-base gap near the 3’ end of the *FUM1* sequence of NRRL 20593. **A –** arrows indicate genes with arrowhead pointing in direction of transcription. *FUM* or *ALT* designations of genes are indicated. For non-*FUM* and non-*ALT* genes, the corresponding protein name from BLASTx hit is indicated. Multicolored bar at bottom of **A** is the nucleotide scale for the mapped region. **B –** Sequence coverage from Map Reads to Reference analysis. For NRRL 20593, the maximum coverage was 599 reads and the lowest was 0 reads for a six-base region near the 3’ end of *FUM1*. For NRRL 66744, the maximum coverage was 349 reads and the lowest was 1 read for the same region with 0 had 0 reads in same region that had 0 coverage in NRRL 20593. The gap in sequences was not present in the As-27, the EGS 39-128 Illumina GA II, or NRRL 66744 PacBio sequence data. **C –** MiSeq sequence reads mapped to GenBank accession AB969680.1 for the 124,657-base *FUM* region of As-27. Each horizontal line indicates a sequence read. A blue line indicates read is a product of paired end sequencing (i.e., sequence is based on sense and antisense sequence data); green line indicates read is from sense sequence only; red line indicates read is from antisense sequence only; and yellow indicates reads are from duplicated sequence in reference sequence. Only mapped reads to a depth of ~100X coverage are shown for each strain.

NRRL 20593 reads mapped to AsS-27 *FUM* region sequence

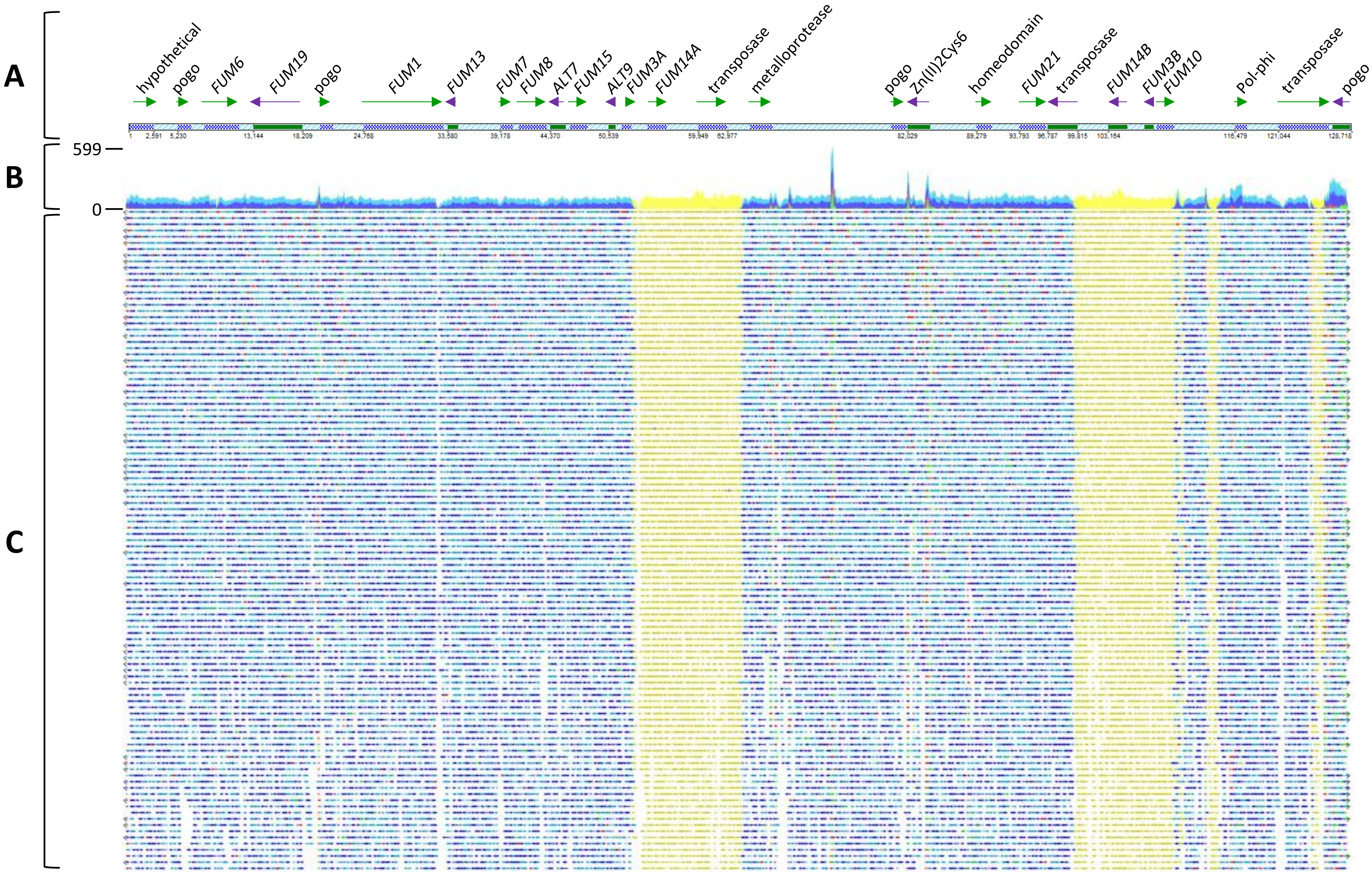

NRRL 66744 reads mapped to AsS-27 *FUM* region sequence

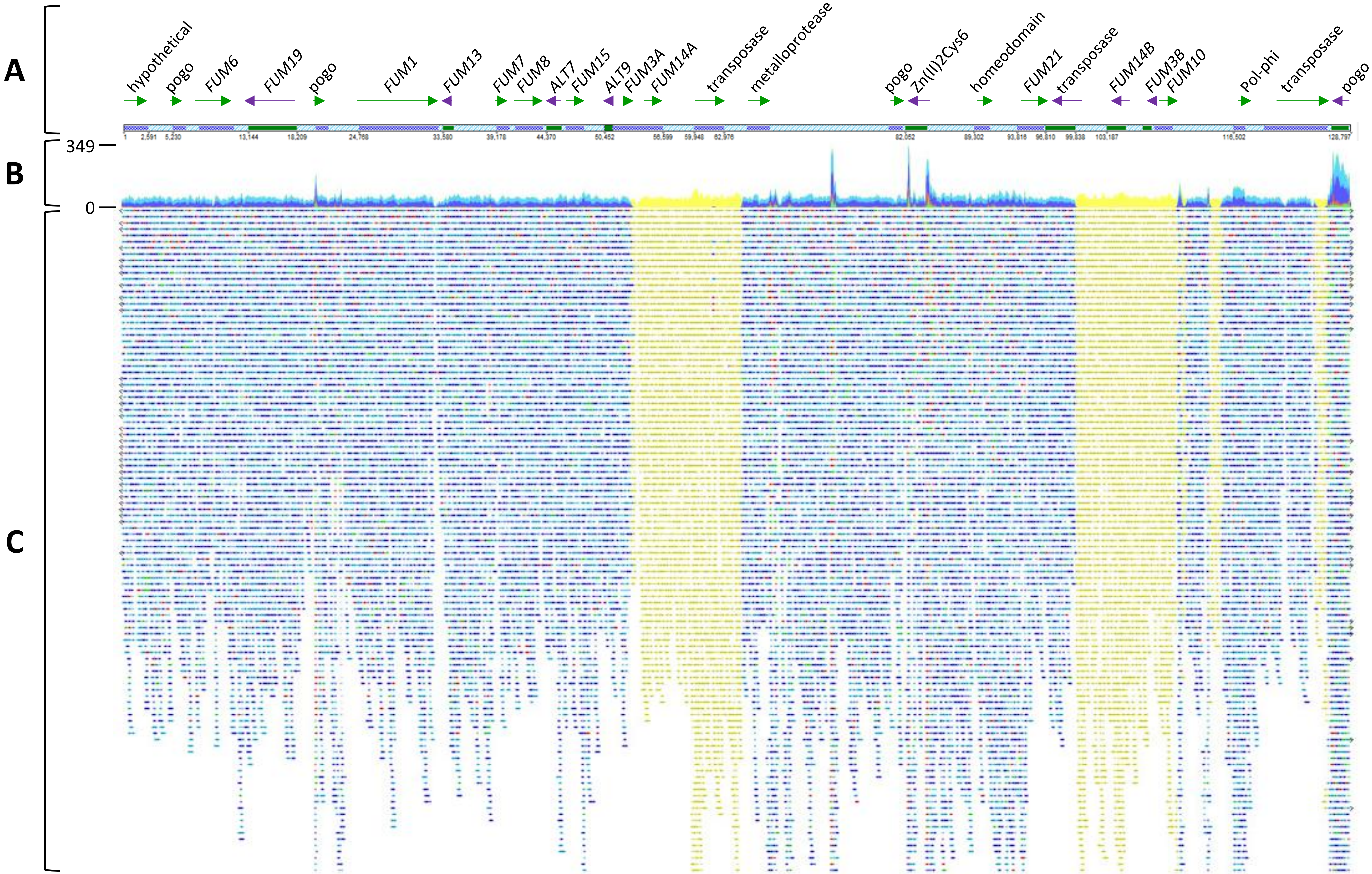

SFigure 5: A) Individual housekeeping gene trees. The alignment of the predicted amino acid sequence of each gene was subjected to maximum likelihood analysis using IQ-Tree. Numbers near branches are bootstrap values based on 1000 replications. Alignments used to infer the trees shown here were concatenated to infer the species tree shown in **Figure 2A**. **B)** Extended Majority Rule Analysis Inferred from 16 ML trees (each tree inferred from a different housekeeping gene).

A) 16 ML trees for individual housekeeping genes

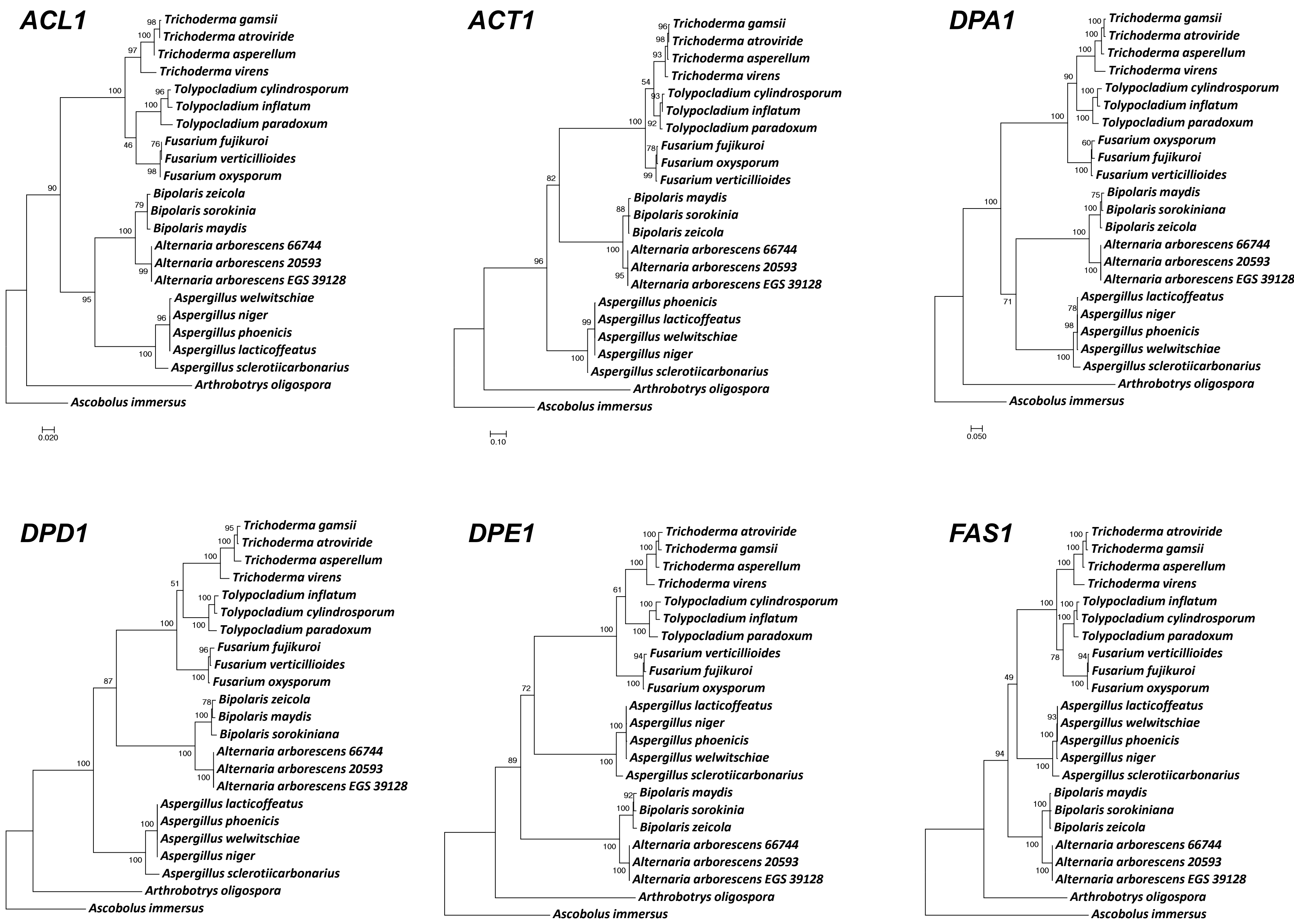

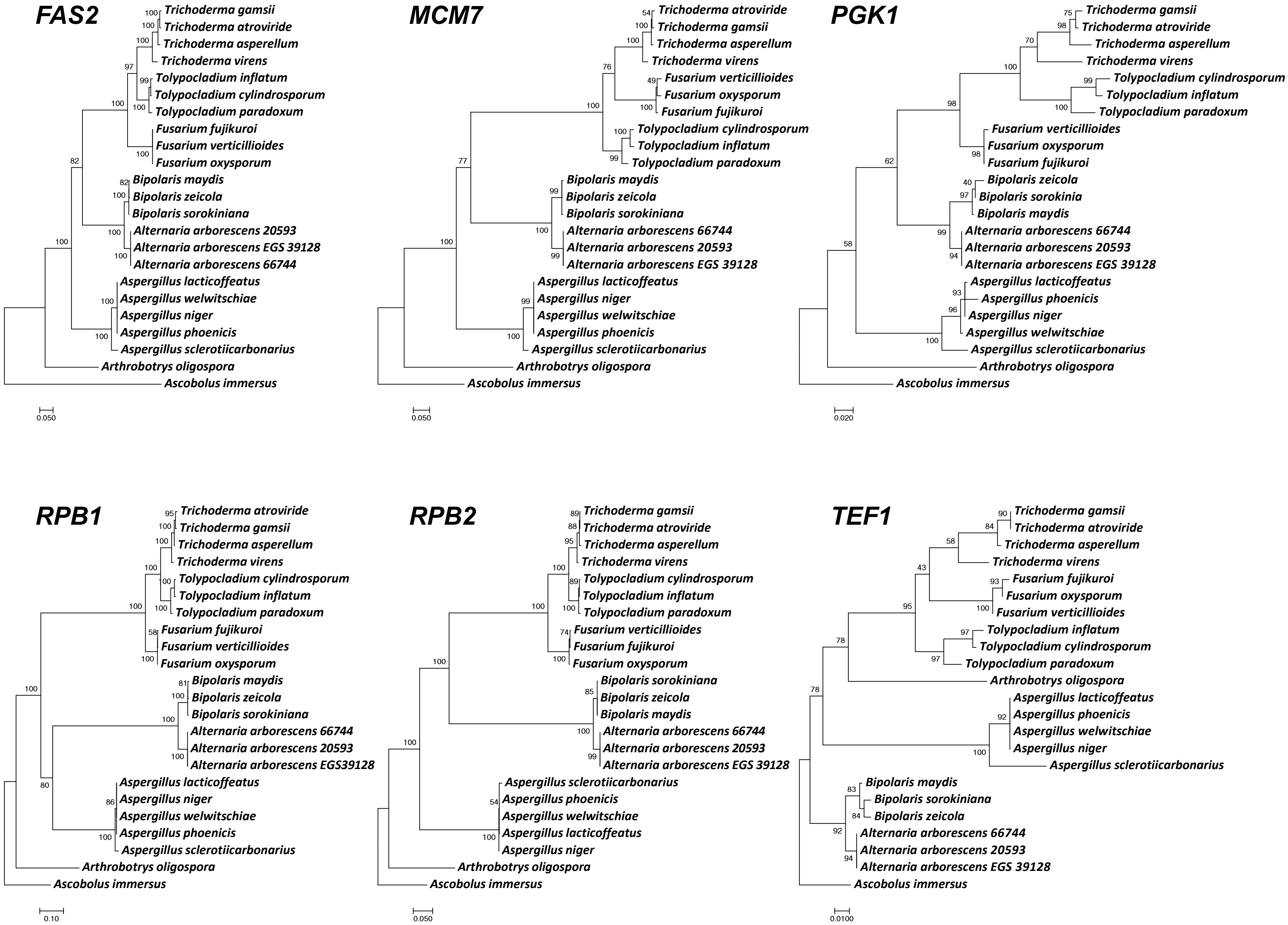
FAS2
Trichoderma gamsii
Trichoderma atroviride
Trichoderma asperellum
Trichoderma virens
Tolypocladium inflatum
Tolypocladium cylindrosporum
Tolypocladium paradoxum
Fusarium fujikuroi
Fusarium verticillioides
Fusarium oxysporum
Bipolaris maydis
Bipolaris zeicola
Bipolaris sorokiniana
Alternaria arborescens 20593
Alternaria arborescens EGS 39128
Alternaria arborescens 66744
Aspergillus lacticoffeatus
Aspergillus welwitschiae
Aspergillus niger
Aspergillus phoenicis
Aspergillus sclerotiicarbonarius
Arthrobotrys oligospora
Ascobolus immersus
0.050
MCM7
Trichoderma atroviride
Trichoderma gamsii
Trichoderma asperellum
Trichoderma virens
Fusarium verticillioides
Fusarium oxysporum
Fusarium fujikuroi
Tolypocladium cylindrosporum
Tolypocladium inflatum
Tolypocladium paradoxum
Bipolaris maydis
Bipolaris zeicola
Bipolaris sorokiniana
Alternaria arborescens 66744
Alternaria arborescens 20593
Alternaria arborescens EGS 39128
Aspergillus lacticoffeatus
Aspergillus niger
Aspergillus welwitschiae
Aspergillus phoenicis
Aspergillus sclerotiicarbonarius
Arthrobotrys oligospora
Ascobolus immersus
0.050
PGK1
Trichoderma gamsii
Trichoderma atroviride
Trichoderma asperellum
Trichoderma virens
Tolypocladium cylindrosporum
Tolypocladium inflatum
Tolypocladium paradoxum
Fusarium verticillioides
Fusarium oxysporum
Fusarium fujikuroi
Bipolaris zeicola
Bipolaris sorokinia
Bipolaris maydis
Alternaria arborescens 66744
Alternaria arborescens 20593
Alternaria arborescens EGS 39128
Aspergillus lacticoffeatus
Aspergillus phoenicis
Aspergillus niger
Aspergillus welwitschiae
Aspergillus sclerotiicarbonarius
Arthrobotrys oligospora
Ascobolus immersus
0.020
RPB1
Trichoderma atroviride
Trichoderma gamsii
Trichoderma asperellum
Trichoderma virens
Tolypocladium cylindrosporum
Tolypocladium inflatum
Tolypocladium paradoxum
Fusarium fujikuroi
Fusarium verticillioides
Fusarium oxysporum
Bipolaris maydis
Bipolaris zeicola
Bipolaris sorokiniana
Alternaria arborescens 66744
Alternaria arborescens 20593
Alternaria arborescens EGS39128
Aspergillus lacticoffeatus
Aspergillus niger
Aspergillus welwitschiae
Aspergillus phoenicis
Aspergillus sclerotiicarbonarius
Arthrobotrys oligospora
Ascobolus immersus
0.10
RPB2
Trichoderma gamsii
Trichoderma atroviride
Trichoderma asperellum
Trichoderma virens
Tolypocladium cylindrosporum
Tolypocladium inflatum
Tolypocladium paradoxum
Fusarium verticillioides
Fusarium fujikuroi
Fusarium oxysporum
Bipolaris sorokiniana
Bipolaris zeicola
Bipolaris maydis
Alternaria arborescens 66744
Alternaria arborescens 20593
Alternaria arborescens EGS 39128
Aspergillus sclerotiicarbonarius
Aspergillus phoenicis
Aspergillus welwitschiae
Aspergillus lacticoffeatus
Aspergillus niger
Arthrobotrys oligospora
Ascobolus immersus
0.050
TEF1
Trichoderma gamsii
Trichoderma atroviride
Trichoderma asperellum
Trichoderma virens
Fusarium fujikuroi
Fusarium oxysporum
Fusarium verticillioides
Tolypocladium inflatum
Tolypocladium cylindrosporum
Tolypocladium paradoxum
Arthrobotrys oligospora
Aspergillus lacticoffeatus
Aspergillus phoenicis
Aspergillus welwitschiae
Aspergillus niger
Aspergillus sclerotiicarbonarius
Bipolaris maydis
Bipolaris sorokiniana
Bipolaris zeicola
Alternaria arborescens 66744
Alternaria arborescens 20593
Alternaria arborescens EGS 39128
Ascobolus immersus
0.0100

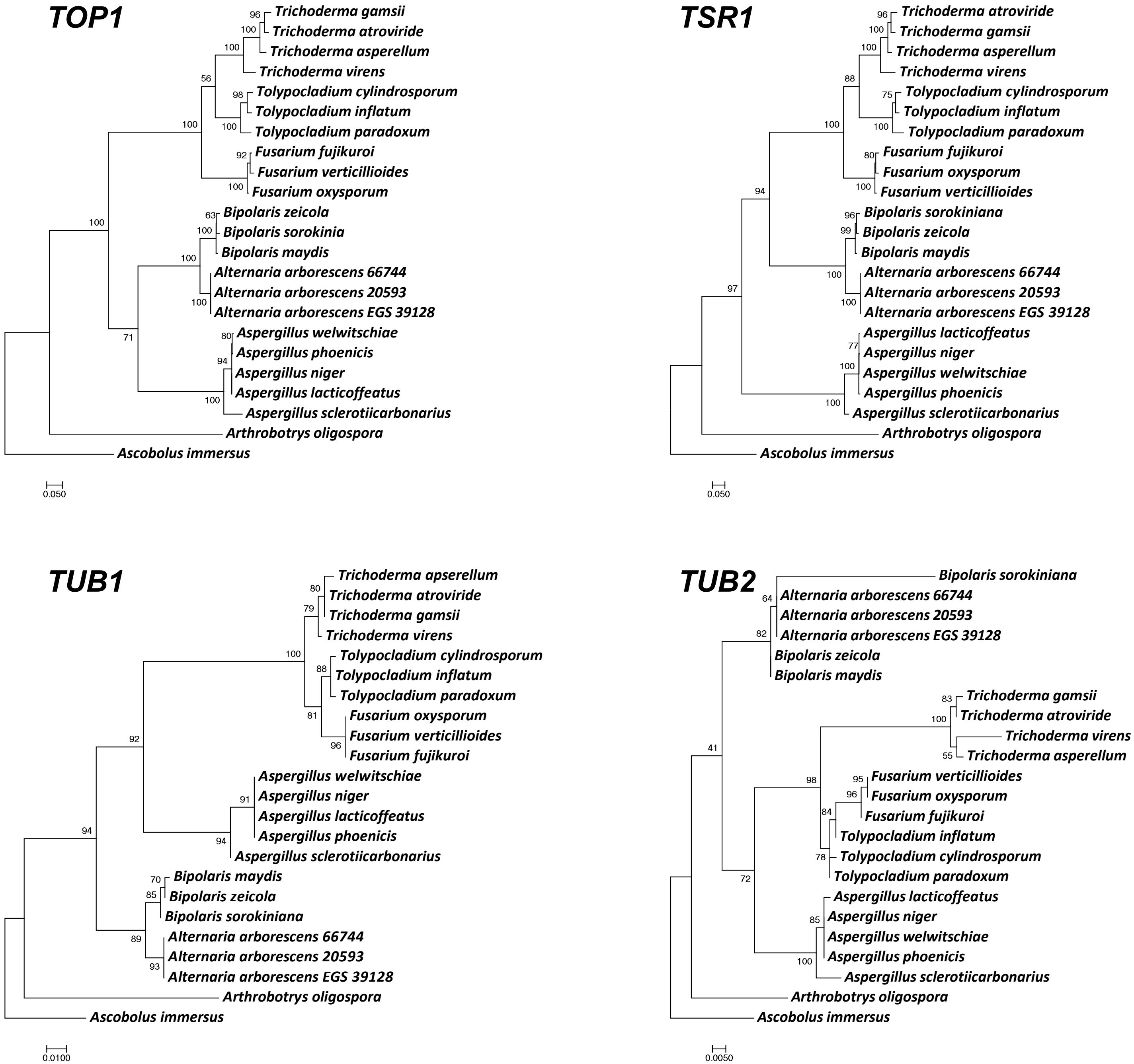

TOP1
Trichoderma gamsii
Trichoderma atroviride
Trichoderma asperellum
Trichoderma virens
Tolypocladium cylindrosporum
Tolypocladium inflatum
Tolypocladium paradoxum
Fusarium fujikuroi
Fusarium verticillioides
Fusarium oxysporum
Bipolaris zeicola
Bipolaris sorokinia
Bipolaris maydis
Alternaria arborescens 66744
Alternaria arborescens 20593
Alternaria arborescens EGS 39128
Aspergillus welwitschiae
Aspergillus phoenicis
Aspergillus niger
Aspergillus lacticoffeatus
Aspergillus sclerotiicarbonarius
Arthrobotrys oligospora
Ascobolus immersus
0.050
TSR1
Trichoderma atroviride
Trichoderma gamsii
Trichoderma asperellum
Trichoderma virens
Tolypocladium cylindrosporum
Tolypocladium inflatum
Tolypocladium paradoxum
Fusarium fujikuroi
Fusarium oxysporum
Fusarium verticillioides
Bipolaris sorokiniana
Bipolaris zeicola
Bipolaris maydis
Alternaria arborescens 66744
Alternaria arborescens 20593
Alternaria arborescens EGS 39128
Aspergillus lacticoffeatus
Aspergillus niger
Aspergillus welwitschiae
Aspergillus phoenicis
Aspergillus sclerotiicarbonarius
Arthrobotrys oligospora
Ascobolus immersus
0.050
TUB1
Trichoderma apserellum
Trichoderma atroviride
Trichoderma gamsii
Trichoderma virens
Tolypocladium cylindrosporum
Tolypocladium inflatum
Tolypocladium paradoxum
Fusarium oxysporum
Fusarium verticillioides
Fusarium fujikuroi
Aspergillus welwitschiae
Aspergillus niger
Aspergillus lacticoffeatus
Aspergillus phoenicis
Aspergillus sclerotiicarbonarius
Bipolaris maydis
Bipolaris zeicola
Bipolaris sorokiniana
Alternaria arborescens 66744
Alternaria arborescens 20593
Alternaria arborescens EGS 39128
Arthrobotrys oligospora
Ascobolus immersus
0.0100
TUB2
Bipolaris sorokiniana
Alternaria arborescens 66744
Alternaria arborescens 20593
Alternaria arborescens EGS 39128
Bipolaris zeicola
Bipolaris maydis
Trichoderma gamsii
Trichoderma atroviride
Trichoderma virens
Trichoderma asperellum
Fusarium verticillioides
Fusarium oxysporum
Fusarium fujikuroi
Tolypocladium inflatum
Tolypocladium cylindrosporum
Tolypocladium paradoxum
Aspergillus lacticoffeatus
Aspergillus niger
Aspergillus welwitschiae
Aspergillus phoenicis
Aspergillus sclerotiicarbonarius
Arthrobotrys oligospora
Ascobolus immersus
0.0050

B) Extended Majority Rule Analysis

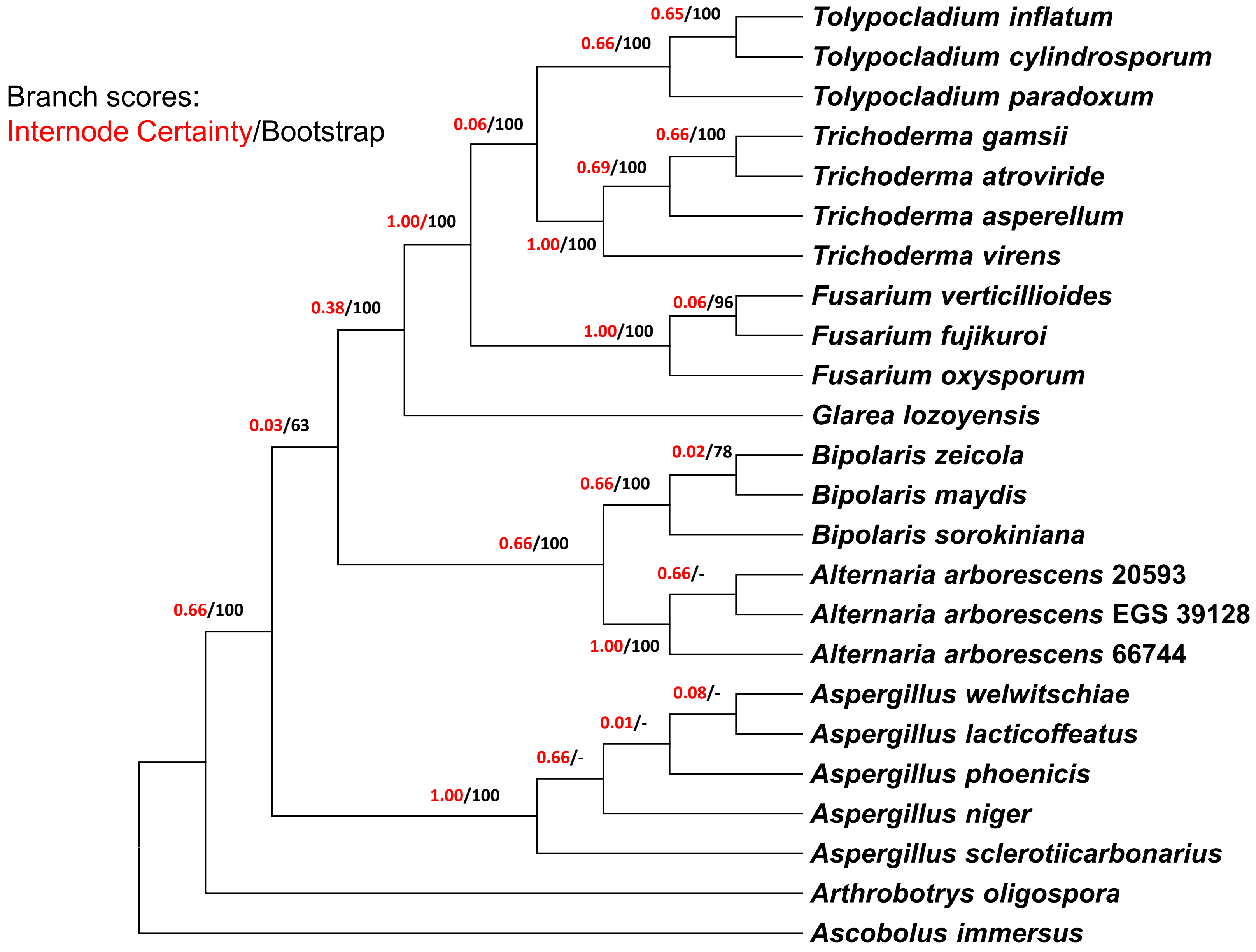

SFigure 6. *FUM* gene trees. Deduced amino acid sequences were aligned using the alignment tool Muscle in MEGA7. The resulting alignments were subjected to maximum likelihood analysis using IQ-Tree (ver. 1.6.9). Substitution models were determined during analysis in IQ-Tree. Values near branches are bootstrap values based on 1000 replications.

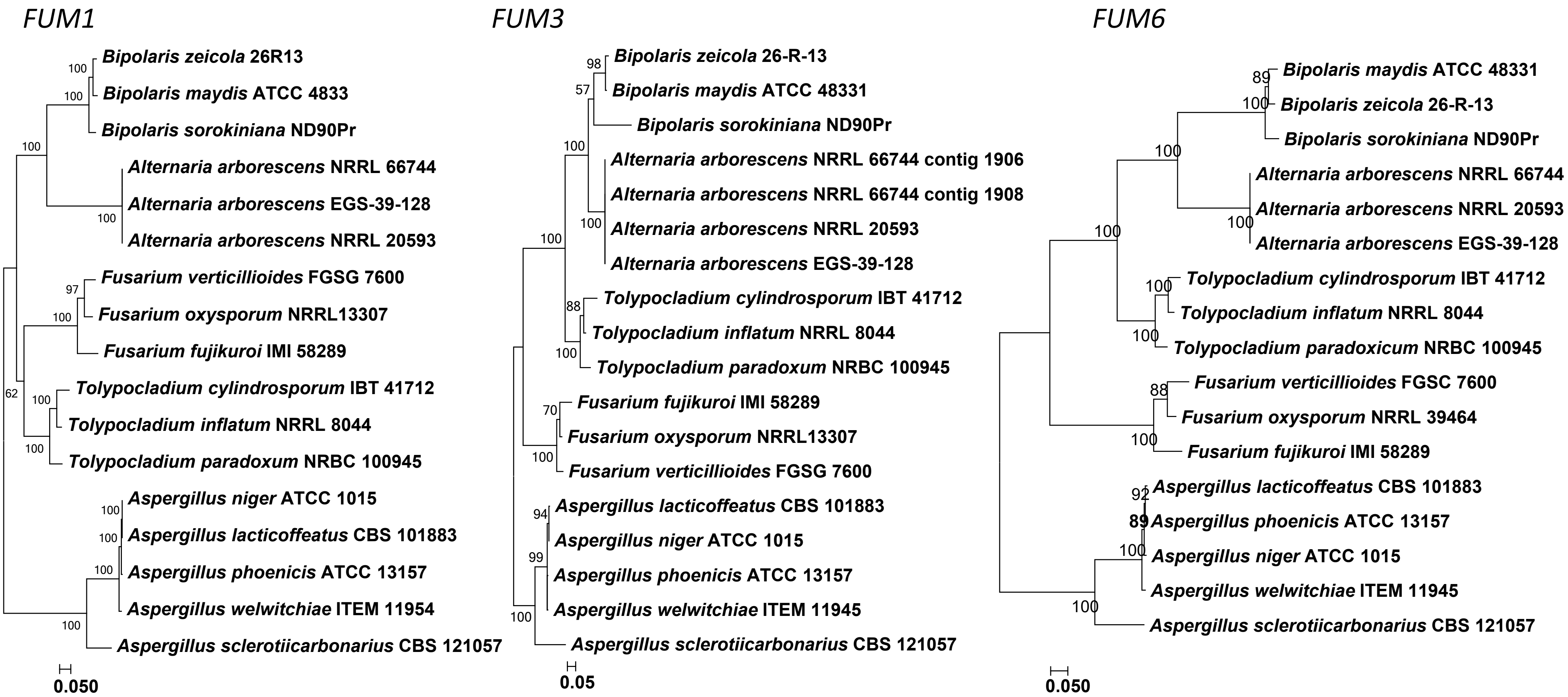

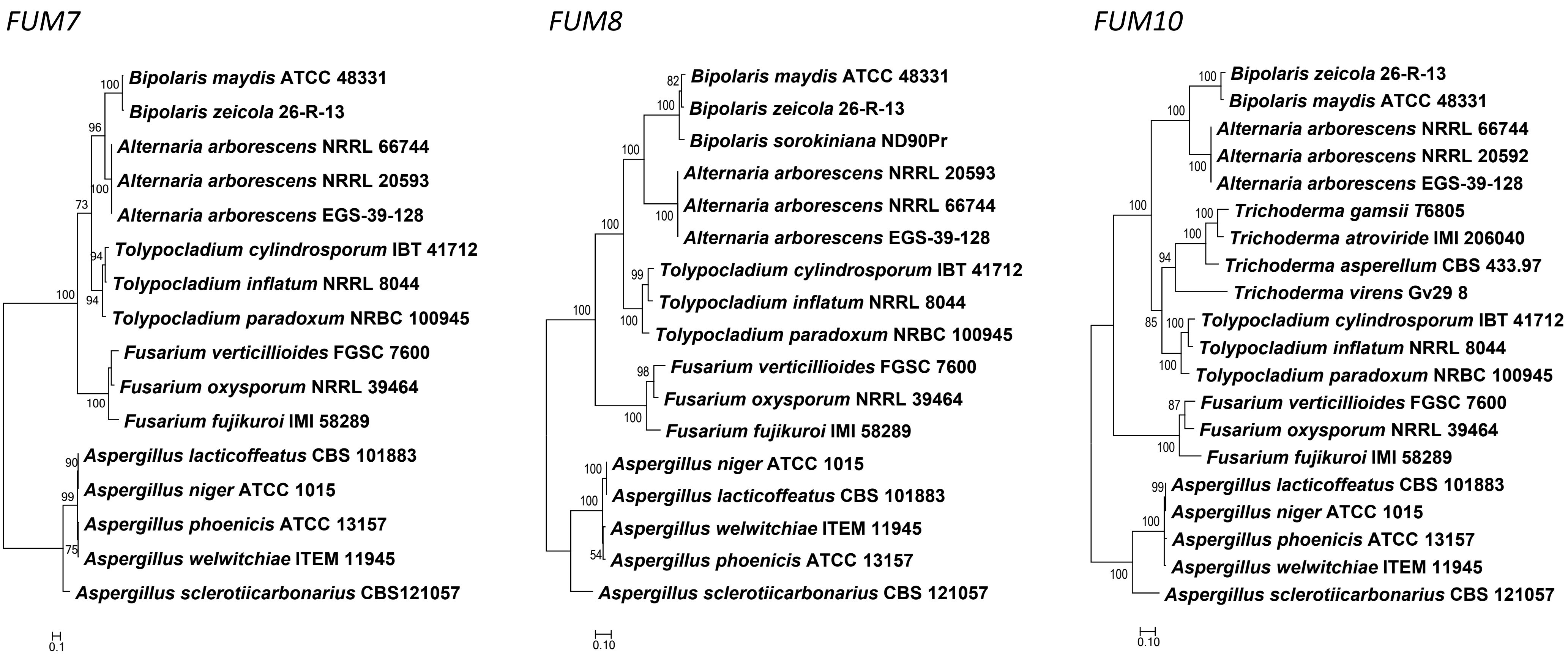
FUM7
Bipolaris maydis ATCC 48331
Bipolaris zeicola 26-R-13
Alternaria arborescens NRRL 66744
Alternaria arborescens NRRL 20593
Alternaria arborescens EGS-39-128
Tolypocladium cylindrosporum IBT 41712
Tolypocladium inflatum NRRL 8044
Tolypocladium paradoxum NRBC 100945
Fusarium verticillioides FGSC 7600
Fusarium oxysporum NRRL 39464
Fusarium fujikuroi IMI 58289
Aspergillus lacticoffeatus CBS 101883
Aspergillus niger ATCC 1015
Aspergillus phoenicis ATCC 13157
Aspergillus welwitchiae ITEM 11945
Aspergillus sclerotiicarbonarius CBS121057
0.1
FUM8
Bipolaris maydis ATCC 48331
Bipolaris zeicola 26-R-13
Bipolaris sorokiniana ND90Pr
Alternaria arborescens NRRL 20593
Alternaria arborescens NRRL 66744
Alternaria arborescens EGS-39-128
Tolypocladium cylindrosporum IBT 41712
Tolypocladium inflatum NRRL 8044
Tolypocladium paradoxum NRBC 100945
Fusarium verticillioides FGSC 7600
Fusarium oxysporum NRRL 39464
Fusarium fujikuroi IMI 58289
Aspergillus niger ATCC 1015
Aspergillus lacticoffeatus CBS 101883
Aspergillus welwitchiae ITEM 11945
Aspergillus phoenicis ATCC 13157
Aspergillus sclerotiicarbonarius CBS 121057
0.10
FUM10
Bipolaris zeicola 26-R-13
Bipolaris maydis ATCC 48331
Alternaria arborescens NRRL 66744
Alternaria arborescens NRRL 20592
Alternaria arborescens EGS-39-128
Trichoderma gamsii T6805
Trichoderma atroviride IMI 206040
Trichoderma asperellum CBS 433.97
Trichoderma virens Gv29 8
Tolypocladium cylindrosporum IBT 41712
Tolypocladium inflatum NRRL 8044
Tolypocladium paradoxum NRBC 100945
Fusarium verticillioides FGSC 7600
Fusarium oxysporum NRRL 39464
Fusarium fujikuroi IMI 58289
Aspergillus lacticoffeatus CBS 101883
Aspergillus niger ATCC 1015
Aspergillus phoenicis ATCC 13157
Aspergillus welwitchiae ITEM 11945
Aspergillus sclerotiicarbonarius CBS 121057
0.10

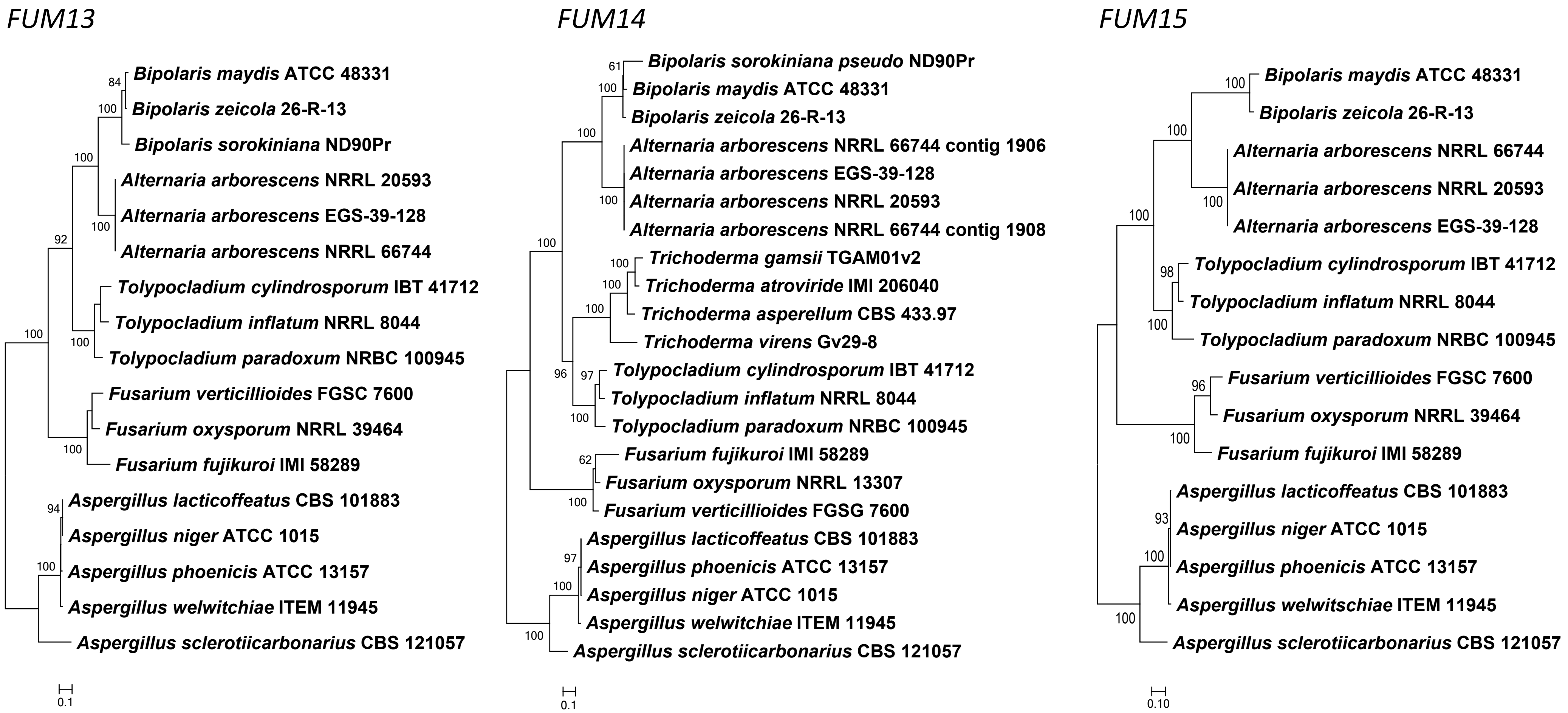
FUM13
Bipolaris maydis ATCC 48331
Bipolaris zeicola 26-R-13
Bipolaris sorokiniana ND90Pr
Alternaria arborescens NRRL 20593
Alternaria arborescens EGS-39-128
Alternaria arborescens NRRL 66744
Tolypocladium cylindrosporum IBT 41712
Tolypocladium inflatum NRRL 8044
Tolypocladium paradoxum NRBC 100945
Fusarium verticillioides FGSC 7600
Fusarium oxysporum NRRL 39464
Fusarium fujikuroi IMI 58289
Aspergillus lacticoffeatus CBS 101883
Aspergillus niger ATCC 1015
Aspergillus phoenicis ATCC 13157
Aspergillus welwitchiae ITEM 11945
Aspergillus sclerotiicarbonarius CBS 121057
0.1
FUM14
Bipolaris sorokiniana pseudo ND90Pr
Bipolaris maydis ATCC 48331
Bipolaris zeicola 26-R-13
Alternaria arborescens NRRL 66744 contig 1906
Alternaria arborescens EGS-39-128
Alternaria arborescens NRRL 20593
Alternaria arborescens NRRL 66744 contig 1908
Trichoderma gamsii TGAM01v2
Trichoderma atroviride IMI 206040
Trichoderma asperellum CBS 433.97
Trichoderma virens Gv29-8
Tolypocladium cylindrosporum IBT 41712
Tolypocladium inflatum NRRL 8044
Tolypocladium paradoxum NRBC 100945
Fusarium fujikuroi IMI 58289
Fusarium oxysporum NRRL 13307
Fusarium verticillioides FGSG 7600
Aspergillus lacticoffeatus CBS 101883
Aspergillus phoenicis ATCC 13157
Aspergillus niger ATCC 1015
Aspergillus welwitchiae ITEM 11945
Aspergillus sclerotiicarbonarius CBS 121057
0.1
FUM15
Bipolaris maydis ATCC 48331
Bipolaris zeicola 26-R-13
Alternaria arborescens NRRL 66744
Alternaria arborescens NRRL 20593
Alternaria arborescens EGS-39-128
Tolypocladium cylindrosporum IBT 41712
Tolypocladium inflatum NRRL 8044
Tolypocladium paradoxum NRBC 100945
Fusarium verticillioides FGSC 7600
Fusarium oxysporum NRRL 39464
Fusarium fujikuroi IMI 58289
Aspergillus lacticoffeatus CBS 101883
Aspergillus niger ATCC 1015
Aspergillus phoenicis ATCC 13157
Aspergillus welwitschiae ITEM 11945
Aspergillus sclerotiicarbonarius CBS 121057
0.10

FUM19

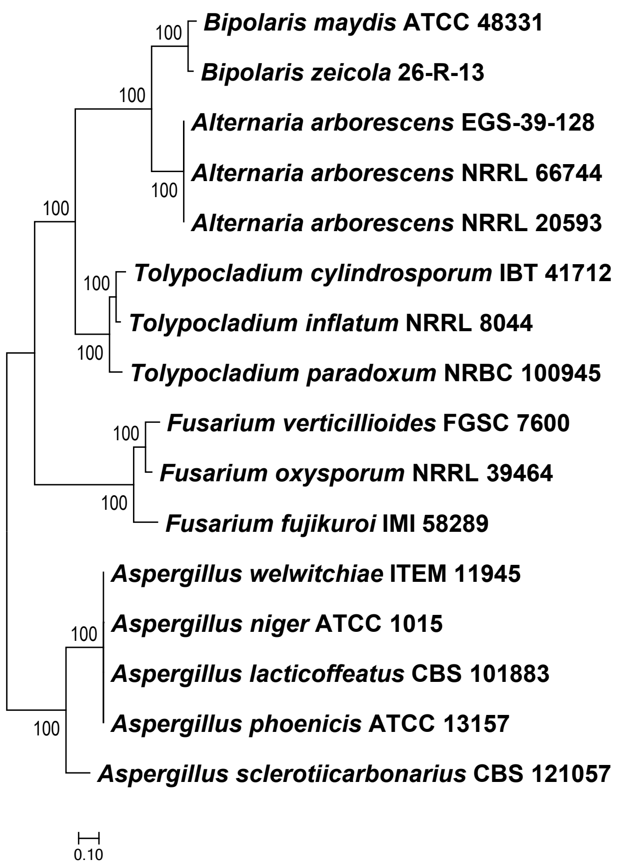

Bipolaris maydis ATCC 48331
Bipolaris zeicola 26-R-13
Alternaria arborescens EGS-39-128
Alternaria arborescens NRRL 66744
Alternaria arborescens NRRL 20593
Tolypocladium cylindrosporum IBT 41712
Tolypocladium inflatum NRRL 8044
Tolypocladium paradoxum NRBC 100945
Fusarium verticillioides FGSC 7600
Fusarium oxysporum NRRL 39464
Fusarium fujikuroi IMI 58289
Aspergillus welwitchiae ITEM 11945
Aspergillus niger ATCC 1015
Aspergillus lacticoffeatus CBS 101883
Aspergillus phoenicis ATCC 13157
Aspergillus sclerotiicarbonarius CBS 121057
100
0.10

FUM21

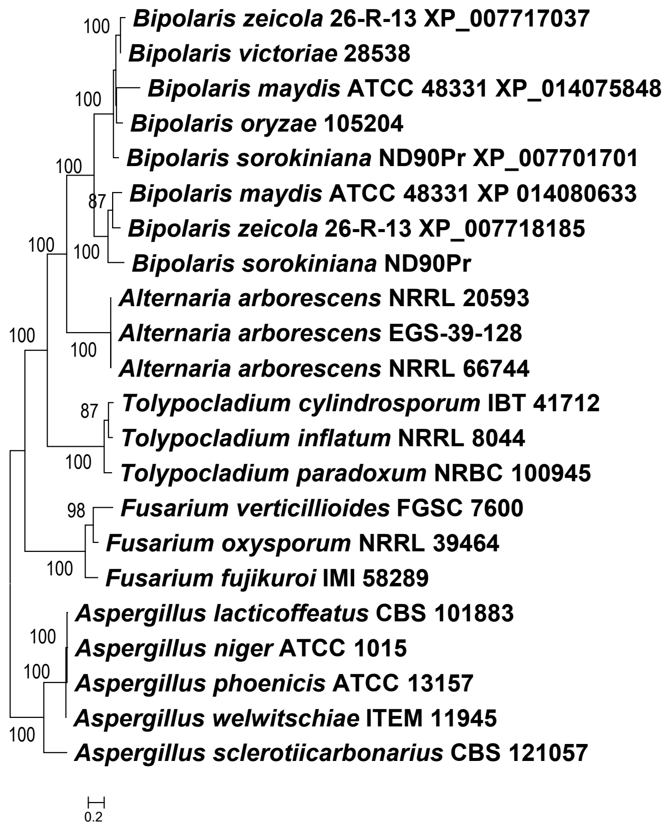

Bipolaris zeicola 26-R-13 XP_007717037
Bipolaris victoriae 28538
Bipolaris maydis ATCC 48331 XP_014075848
Bipolaris oryzae 105204
Bipolaris sorokiniana ND90Pr XP_007701701
Bipolaris maydis ATCC 48331 XP 014080633
Bipolaris zeicola 26-R-13 XP_007718185
Bipolaris sorokiniana ND90Pr
Alternaria arborescens NRRL 20593
Alternaria arborescens EGS-39-128
Alternaria arborescens NRRL 66744
Tolypocladium cylindrosporum IBT 41712
Tolypocladium inflatum NRRL 8044
Tolypocladium paradoxum NRBC 100945
Fusarium verticillioides FGSC 7600
Fusarium oxysporum NRRL 39464
Fusarium fujikuroi IMI 58289
Aspergillus lacticoffeatus CBS 101883
Aspergillus niger ATCC 1015
Aspergillus phoenicis ATCC 13157
Aspergillus welwitschiae ITEM 11945
Aspergillus sclerotiicarbonarius CBS 121057
100
87
98
0.2

SFigure7. NOTUNG reconciliation analysis of individual *FUM* gene trees with the species tree inferred from 16 concatenated housekeeping genes. Horizontal transfer events (yellow arrows) and duplication events at specific nodes (green circles) and loss events (grey nodes/taxa). All trees were inferred with alignments of predicted amino acid sequences

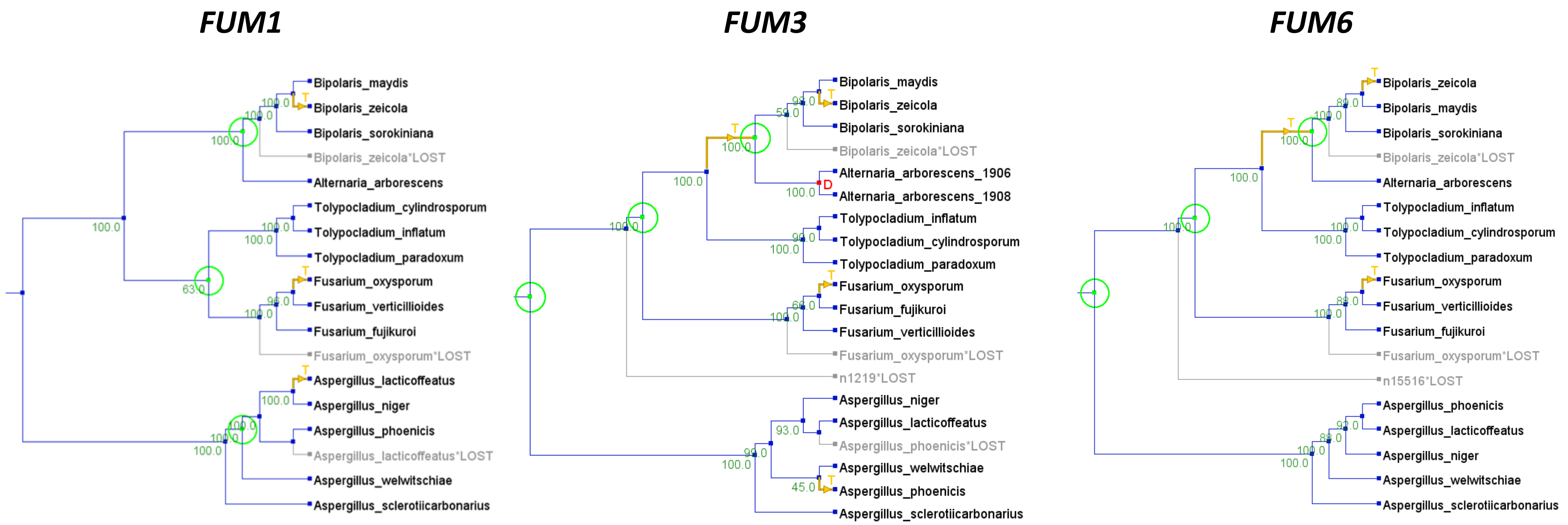

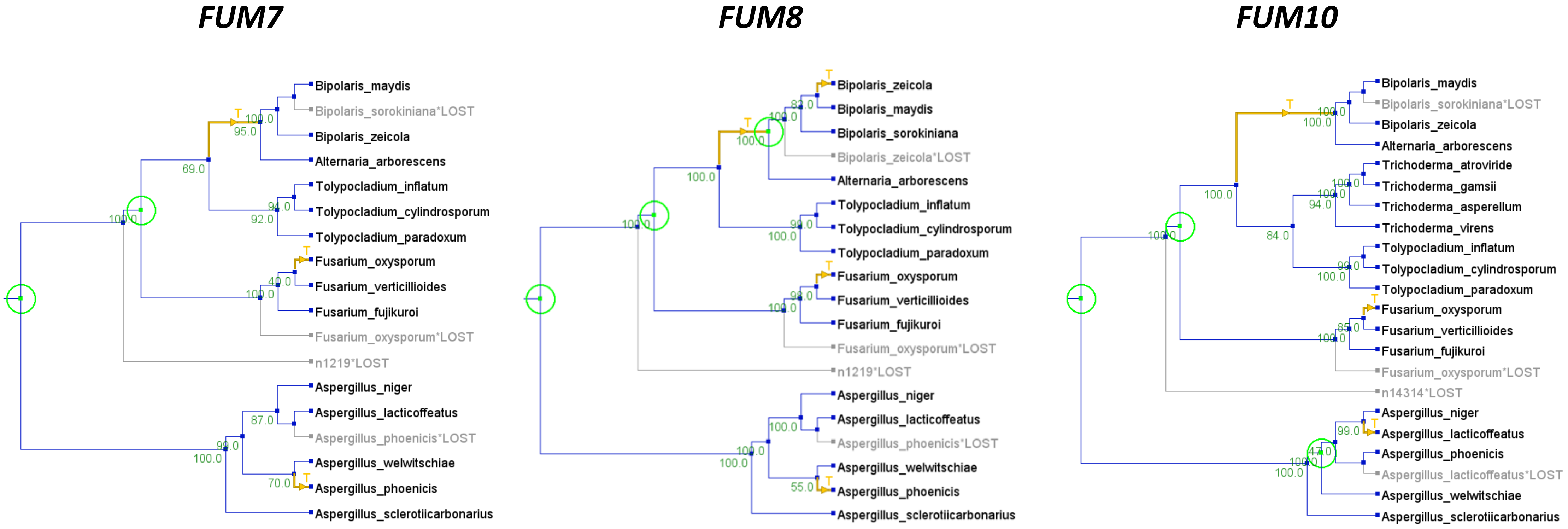

FUM7
FUM8
FUM10
Bipolaris_maydis
Bipolaris_sorokiniana*LOST
Bipolaris_zeicola
Alternaria_arborescens
Tolypocladium_inflatum
Tolypocladium_cylindrosporum
Tolypocladium_paradoxum
Fusarium_oxysporum
Fusarium_verticillioides
Fusarium_fujikuroi
Fusarium_oxysporum*LOST
n1219*LOST
Aspergillus_niger
Aspergillus_lacticoffeatus
Aspergillus_phoenicis*LOST
Aspergillus_welwitschiae
Aspergillus_phoenicis
Aspergillus_sclerotiicarbonarius
Bipolaris_zeicola
Bipolaris_maydis
Bipolaris_sorokiniana
Bipolaris_zeicola*LOST
Alternaria_arborescens
Tolypocladium_inflatum
Tolypocladium_cylindrosporum
Tolypocladium_paradoxum
Fusarium_oxysporum
Fusarium_verticillioides
Fusarium_fujikuroi
Fusarium_oxysporum*LOST
n1219*LOST
Aspergillus_niger
Aspergillus_lacticoffeatus
Aspergillus_phoenicis*LOST
Aspergillus_welwitschiae
Aspergillus_phoenicis
Aspergillus_sclerotiicarbonarius
Bipolaris_maydis
Bipolaris_sorokiniana*LOST
Bipolaris_zeicola
Alternaria_arborescens
Trichoderma_atroviride
Trichoderma_gamsii
Trichoderma_asperellum
Trichoderma_virens
Tolypocladium_inflatum
Tolypocladium_cylindrosporum
Tolypocladium_paradoxum
Fusarium_oxysporum
Fusarium_verticillioides
Fusarium_fujikuroi
Fusarium_oxysporum*LOST
n14314*LOST
Aspergillus_niger
Aspergillus_lacticoffeatus
Aspergillus_phoenicis
Aspergillus_lacticoffeatus*LOST
Aspergillus_welwitschiae
Aspergillus_sclerotiicarbonarius

FUM13

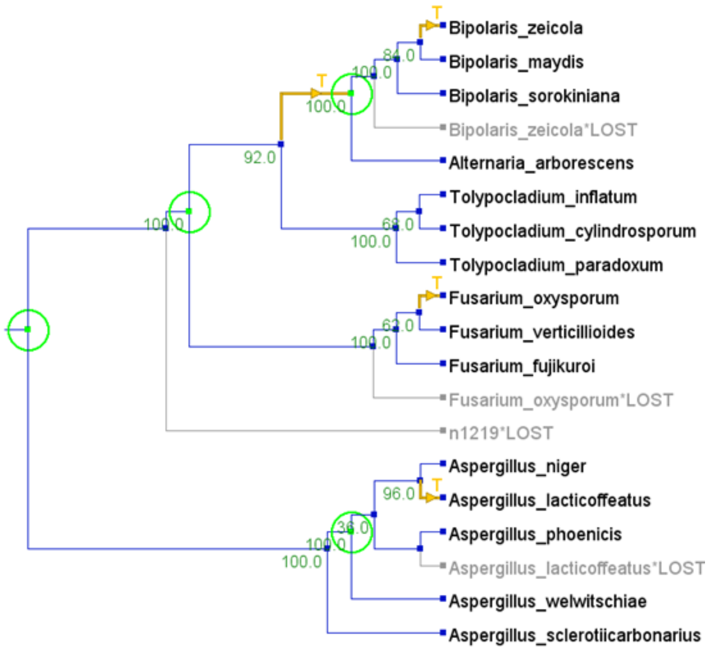

FUM14

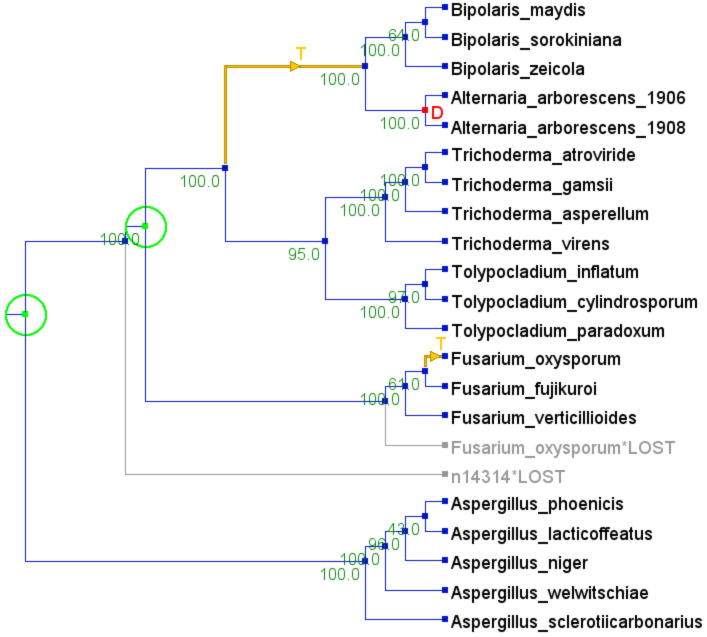

FUM15

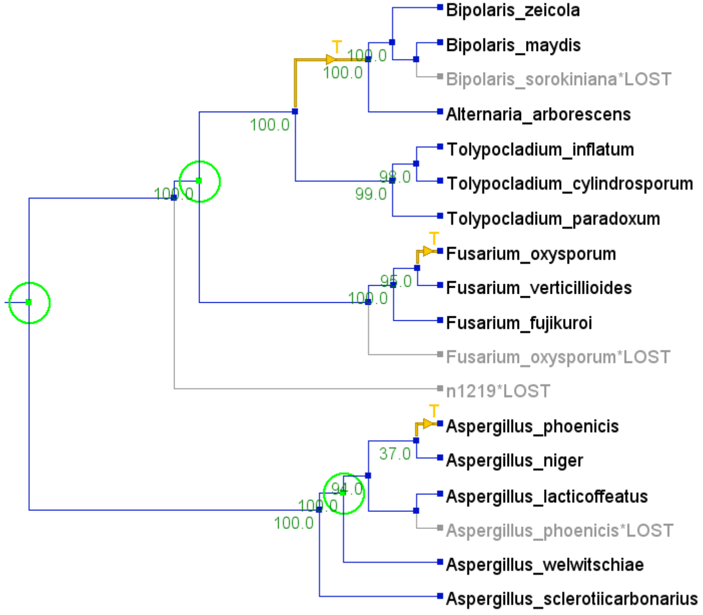

FUM19

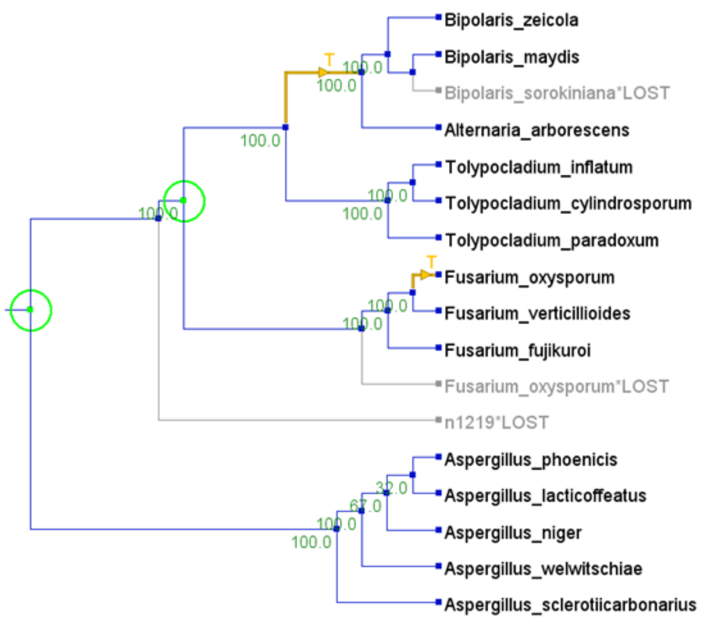
Bipolaris_zeicola
Bipolaris_maydis
Bipolaris_sorokiniana*LOST
Alternaria_arborescens
Tolypocladium_inflatum
Tolypocladium_cylindrosporum
Tolypocladium_paradoxum
Fusarium_oxysporum
Fusarium_verticillioides
Fusarium_fujikuroi
Fusarium_oxysporum*LOST
n1219*LOST
Aspergillus_phoenicis
Aspergillus_lacticoffeatus
Aspergillus_niger
Aspergillus_welwitschiae
Aspergillus_sclerotiicarbonarius

FUM21

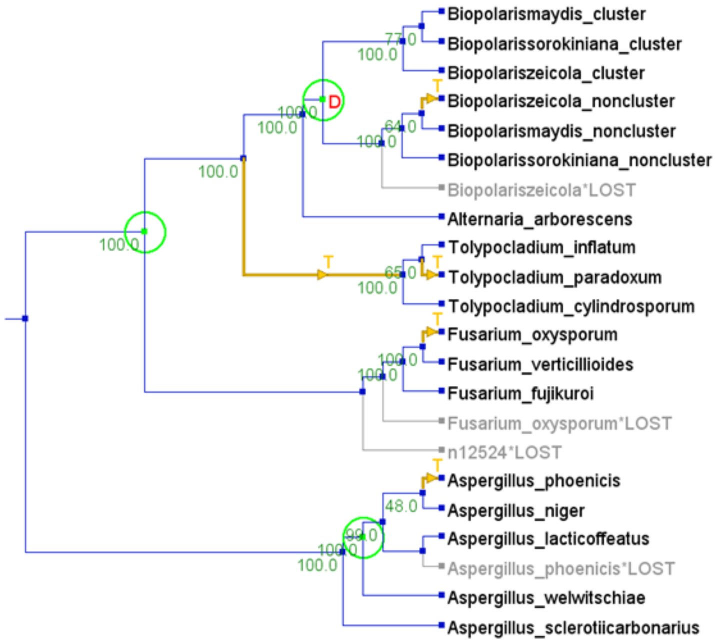
Biopolarismaydis_cluster
Biopolarissorokiniana_cluster
Biopolariszeicola_cluster
Biopolariszeicola_noncluster
Biopolarismaydis_noncluster
Biopolarissorokiniana_noncluster
Biopolariszeicola*LOST
Alternaria_arborescens
Tolypocladium_inflatum
Tolypocladium_paradoxum
Tolypocladium_cylindrosporum
Fusarium_oxysporum
Fusarium_verticillioides
Fusarium_fujikuroi
Fusarium_oxysporum*LOST
n12524*LOST
Aspergillus_phoenicis
Aspergillus_niger
Aspergillus_lacticoffeatus
Aspergillus_phoenicis*LOST
Aspergillus_welwitschiae
Aspergillus_sclerotiicarbonarius

SFigure8. FUM gene trees with closely related proteins from outgroup taxa showing *Aspergillus* lineage as the earliest diverging lineage. Deduced amino acid sequences were aligned using the alignment tool Muscle in MEGA7. The resulting alignments were subjected to maximum likelihood analysis using IQ-Tree (ver. 1.6.9). Substitution models were determined during analysis in IQ-Tree. Values near branches are bootstrap values based on 1000 replications.

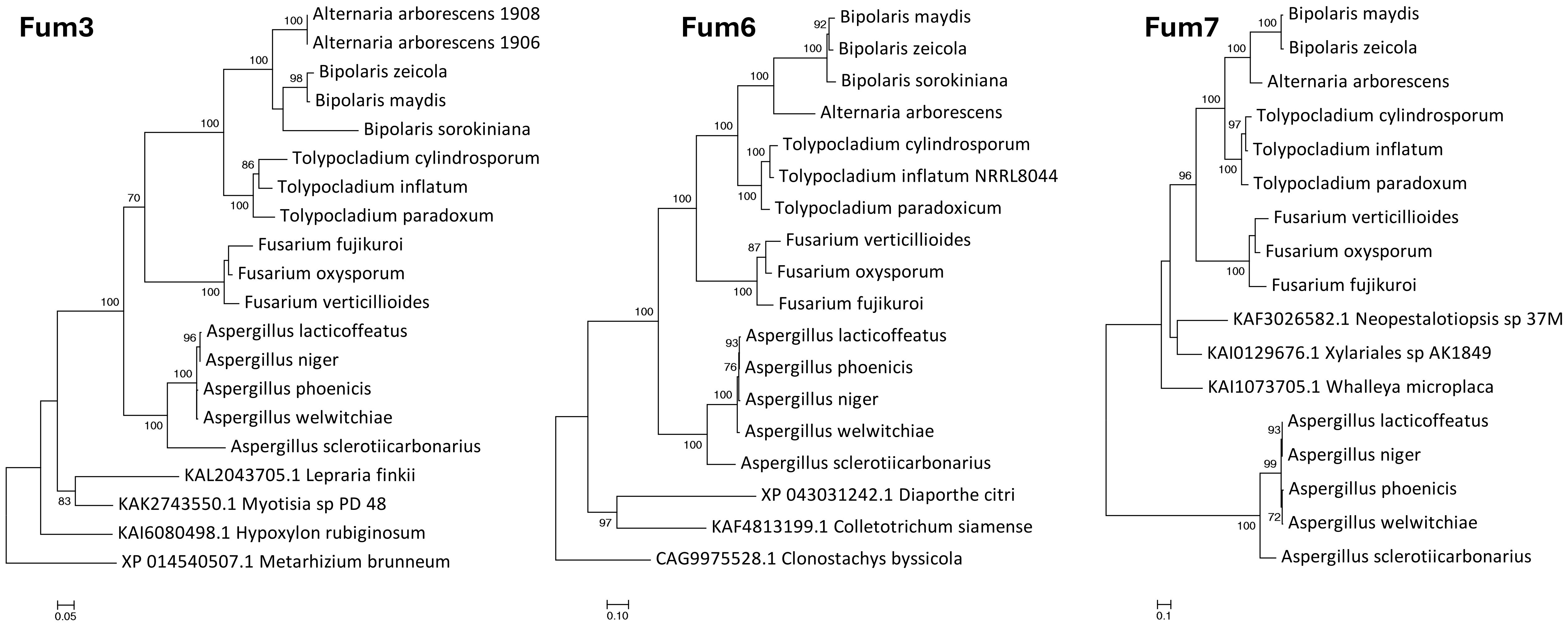

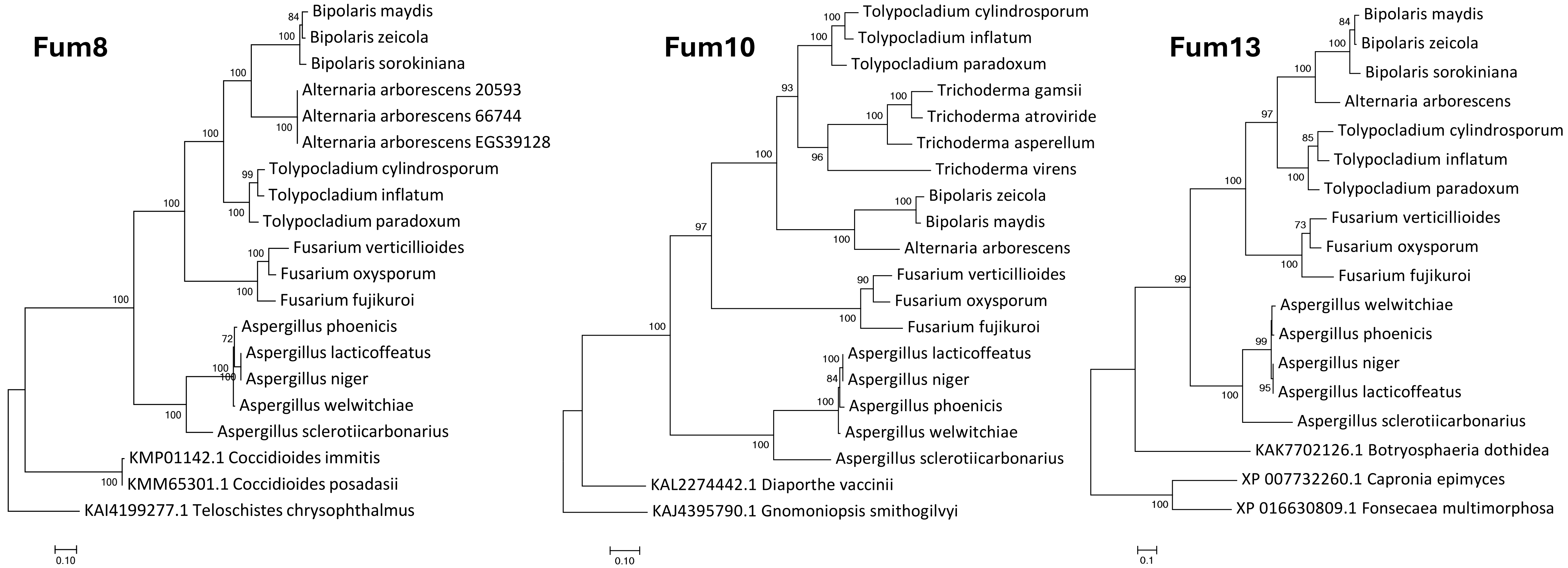
Fum8
Bipolaris maydis
Bipolaris zeicola
Bipolaris sorokiniana
Alternaria arborescens 20593
Alternaria arborescens 66744
Alternaria arborescens EGS39128
Tolypocladium cylindrosporum
Tolypocladium inflatum
Tolypocladium paradoxum
Fusarium verticillioides
Fusarium oxysporum
Fusarium fujikuroi
Aspergillus phoenicis
Aspergillus lacticoffeatus
Aspergillus niger
Aspergillus welwitchiae
Aspergillus sclerotiicarbonarius
KMP01142.1 Coccidioides immitis
KMM65301.1 Coccidioides posadasii
KAI4199277.1 Teloschistes chrysophthalmus
0.10
Fum10
Tolypocladium cylindrosporum
Tolypocladium inflatum
Tolypocladium paradoxum
Trichoderma gamsii
Trichoderma atroviride
Trichoderma asperellum
Trichoderma virens
Bipolaris zeicola
Bipolaris maydis
Alternaria arborescens
Fusarium verticillioides
Fusarium oxysporum
Fusarium fujikuroi
Aspergillus lacticoffeatus
Aspergillus niger
Aspergillus phoenicis
Aspergillus welwitchiae
Aspergillus sclerotiicarbonarius
KAL2274442.1 Diaporthe vaccinii
KAJ4395790.1 Gnomoniopsis smithogilvyi
0.10
Fum13
Bipolaris maydis
Bipolaris zeicola
Bipolaris sorokiniana
Alternaria arborescens
Tolypocladium cylindrosporum
Tolypocladium inflatum
Tolypocladium paradoxum
Fusarium verticillioides
Fusarium oxysporum
Fusarium fujikuroi
Aspergillus welwitchiae
Aspergillus phoenicis
Aspergillus niger
Aspergillus lacticoffeatus
Aspergillus sclerotiicarbonarius
KAK7702126.1 Botryosphaeria dothidea
XP 007732260.1 Capronia epimyces
XP 016630809.1 Fonsecaea multimorphosa
0.1

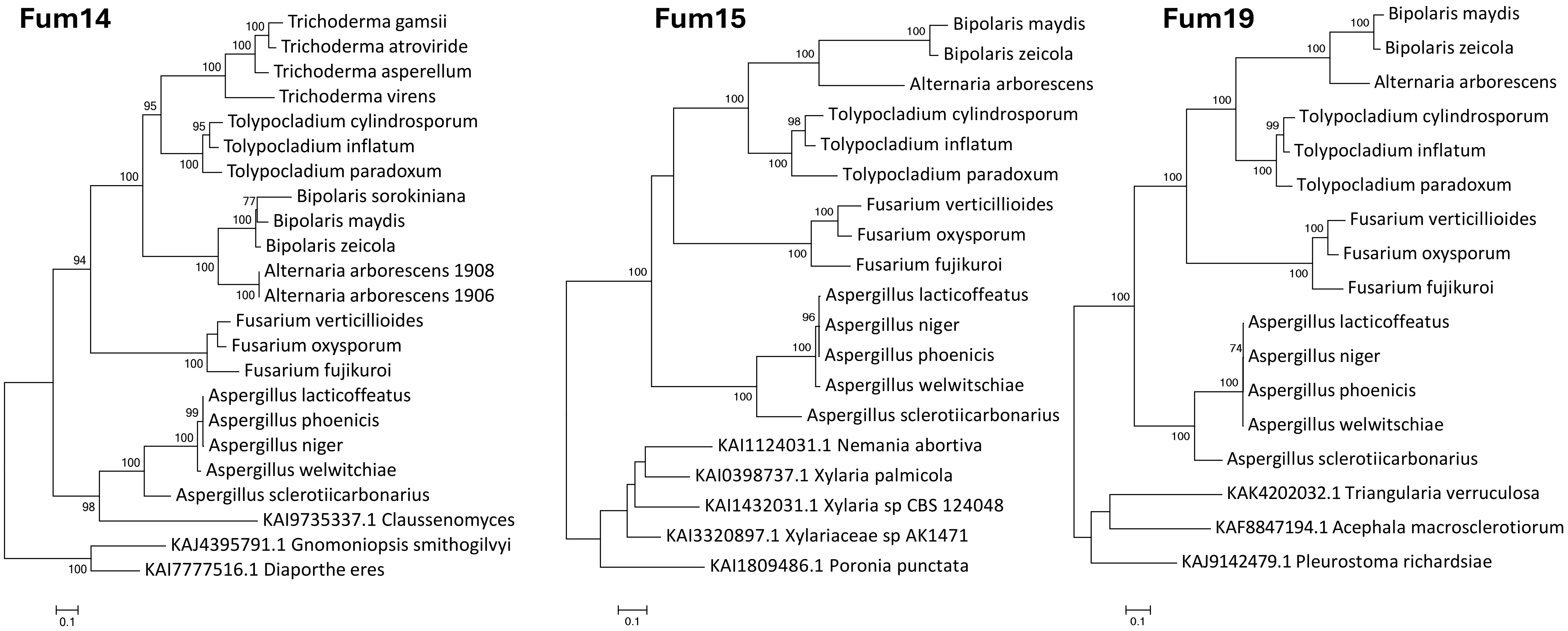
Fum14
Trichoderma gamsii
Trichoderma atroviride
Trichoderma asperellum
Trichoderma virens
Tolypocladium cylindrosporum
Tolypocladium inflatum
Tolypocladium paradoxum
Bipolaris sorokiniana
Bipolaris maydis
Bipolaris zeicola
Alternaria arborescens 1908
Alternaria arborescens 1906
Fusarium verticillioides
Fusarium oxysporum
Fusarium fujikuroi
Aspergillus lacticoffeatus
Aspergillus phoenicis
Aspergillus niger
Aspergillus welwitchiae
Aspergillus sclerotiicarbonarius
KAI9735337.1 Claussenomyces
KAJ4395791.1 Gnomoniopsis smithogilvyi
KAI7777516.1 Diaporthe eres
0.1
Fum15
Bipolaris maydis
Bipolaris zeicola
Alternaria arborescens
Tolypocladium cylindrosporum
Tolypocladium inflatum
Tolypocladium paradoxum
Fusarium verticillioides
Fusarium oxysporum
Fusarium fujikuroi
Aspergillus lacticoffeatus
Aspergillus niger
Aspergillus phoenicis
Aspergillus welwitschiae
Aspergillus sclerotiicarbonarius
KAI1124031.1 Nemania abortiva
KAI0398737.1 Xylaria palmicola
KAI1432031.1 Xylaria sp CBS 124048
KAI3320897.1 Xylariaceae sp AK1471
KAI1809486.1 Poronia punctata
0.1
Fum19
Bipolaris maydis
Bipolaris zeicola
Alternaria arborescens
Tolypocladium cylindrosporum
Tolypocladium inflatum
Tolypocladium paradoxum
Fusarium verticillioides
Fusarium oxysporum
Fusarium fujikuroi
Aspergillus lacticoffeatus
Aspergillus niger
Aspergillus phoenicis
Aspergillus welwitschiae
Aspergillus sclerotiicarbonarius
KAK4202032.1 Triangularia verruculosa
KAF8847194.1 Acephala macrosclerotiorum
KAJ9142479.1 Pleurostoma richardsiae
0.1
